# ChromaSig: A Probabilistic Approach to Finding Common Chromatin Signatures in the Human Genome

**DOI:** 10.1371/journal.pcbi.1000201

**Published:** 2008-10-17

**Authors:** Gary Hon, Bing Ren, Wei Wang

**Affiliations:** 1 Bioinformatics Program, University of California San Diego, La Jolla, California, United States of America; 2 Ludwig Institute for Cancer Research, University of California San Diego, La Jolla, California, United States of America; 3 Department of Cellular and Molecular Medicine and Moores Cancer Center, UCSD School of Medicine, University of California San Diego, La Jolla, California, United States of America; 4 Center for Theoretical Biological Physics, Department of Chemistry and Biochemistry, University of California San Diego, La Jolla, California, United States of America; Duke University, United States of America

## Abstract

Computational methods to identify functional genomic elements using genetic information have been very successful in determining gene structure and in identifying a handful of *cis*-regulatory elements. But the vast majority of regulatory elements have yet to be discovered, and it has become increasingly apparent that their discovery will not come from using genetic information alone. Recently, high-throughput technologies have enabled the creation of information-rich epigenetic maps, most notably for histone modifications. However, tools that search for functional elements using this epigenetic information have been lacking. Here, we describe an unsupervised learning method called ChromaSig to find, in an unbiased fashion, commonly occurring chromatin signatures in both tiling microarray and sequencing data. Applying this algorithm to nine chromatin marks across a 1% sampling of the human genome in HeLa cells, we recover eight clusters of distinct chromatin signatures, five of which correspond to known patterns associated with transcriptional promoters and enhancers. Interestingly, we observe that the distinct chromatin signatures found at enhancers mark distinct functional classes of enhancers in terms of transcription factor and coactivator binding. In addition, we identify three clusters of novel chromatin signatures that contain evolutionarily conserved sequences and potential *cis*-regulatory elements. Applying ChromaSig to a panel of 21 chromatin marks mapped genomewide by ChIP-Seq reveals 16 classes of genomic elements marked by distinct chromatin signatures. Interestingly, four classes containing enrichment for repressive histone modifications appear to be locally heterochromatic sites and are enriched in quickly evolving regions of the genome. The utility of this approach in uncovering novel, functionally significant genomic elements will aid future efforts of genome annotation via chromatin modifications.

## Author Summary

The DNA in eukaryotes is packaged by histones. Interestingly, histones can be marked by a variety of posttranslational modifications, and it has been hypothesized that distinct combinations of histone modifications mark at distinct functional regions of the genome. The study of histone modifications has been aided by the development of high-throughput techniques to map a wide assortment of histone modifications on a global scale. However, because much of our current understanding of the human genome is concentrated on promoters, most studies have only examined histone modifications at these well-defined sites, ignoring the vast majority of the genome. To aid in the discovery of functional elements outside of these well-annotated loci, we develop an unbiased method that searches for commonly occurring histone modification patterns on a global scale without using any annotation information. This method recovers known patterns associated with transcriptional enhancers and promoters. Supporting the histone code hypothesis, we discover that the different functional activities of enhancers are closely associated with the presence of different histone modification patterns. We also discover several novel patterns that likely contain other potential regulatory elements. As the availability of large-scale histone modification data increases, the ability of methods such as the one presented here to concisely describe commonly occurring chromatin signatures, thereby abstracting away irrelevant or redundant data, will become increasingly more critical.

## Introduction

In eukaryotes, DNA is packaged into nucleosomes, each consisting of an octamer of histone proteins [1]–[3]. Histones are subject to an assortment of post-translational modifications including phosphorylation, acetylation, and methylation [4]–[6]. Many of these modifications have been linked to transcriptional activation, silencing, heterochromatin formation [1], [3], [7]–[9], DNA damage sensing and repair [10], and chromosomal segregation [11]. Evidence is accumulating to support the hypothesis that different combinations of histone modifications confer different functional specificities [12]. For example, in *Saccharomyces cerevisiae*, the nucleosomes near active promoters are marked by H3K9ac and H3K4me3, while inactive promoters generally lack these marks [1],[13],[14]. In human, active promoters are associated with H3K4me3, and enhancers are associated with H3K4me1 but lack H3K4me3 [15]. With dozens of covalent modifications already detected on histones, it is conceivable that additional patterns of chromatin modifications exist, and may reveal novel functional elements of the genome.

High-throughput experimental techniques, such as chromatin immunoprecipitation on a microarray (ChIP–chip) [16],[17] and its sequencing-based variant ChIP-Seq [18], have been used to map the enrichment of modified histones on a large scale [15]. This data has revealed that the profiles of chromatin modifications over large genomic regions define functional domains. In principle, analysis of the chromatin modification patterns should allow identification of different classes of functional elements associated with the different histone modifications. However, tools for finding chromatin modification patterns have been lacking [1],[13],[14].

Previously, supervised classification methods have been used to identify chromatin modification patterns at known functional sites [13], [15], [19]–[21]. For example, many studies focus entirely on well-defined transcriptional promoters [3],[8],[9],[13],[15]. But this supervised approach of focusing only on annotated loci trivializes the problem of finding commonly occurring histone modification patterns on a global scale. One of the main motivations for developing an unsupervised learning method is that we do not know *a priori* what functional elements are associated with specific histone modification patterns.

Here, we develop a novel, unbiased method for identification of histone modification patterns occurring repeatedly in the genome. We assume that a consistent repertoire of chromatin modification patterns exists, and that a pattern search algorithm should identify such patterns in an unbiased fashion without using any annotations. We treat this problem as a variant of the standard motif finding problem: given a sequence over an alphabet, find subsequences that are repeated more often than would be expected by chance. Here, rather than working with a sequence over a discrete alphabet such as nucleotides or amino acids, we have a sequence of real-valued enrichment of chromatin modifications over a genomic region. To perform motif finding over chromatin modifications, we develop a probabilistic method called ChromaSig. Applying ChromaSig to a panel of chromatin maps from ChIP–chip experiments performed in HeLa cells on ENCODE arrays, we recover eight distinct clusters of chromatin signatures. We recover known patterns observed at putative active promoters and enhancers [15], as well as several previously uncharacterized patterns. Furthermore, the distinct chromatin signatures found at enhancers mark distinct functional classes of enhancers in terms of transcription factor and co-activator binding. Finally, we also apply ChromaSig genomewide to 21 chromatin marks mapped using ChIP-Seq in CD4+ T cells, recovering 16 distinct and frequently occurring chromatin signatures. ChromaSig reveals frequent and redundant cross-talk between different histone modifications at a previously unappreciated level, and reveals a unique class of quickly-evolving genome elements consistently marked by repressive histone modifications. These results support the utility of ChromaSig in discovering of novel chromatin signatures.

## Methods

### Overview of ChromaSig

We represent large-scale chromatin modifications maps as enrichment over consecutively tiled 100-bp bins. To find frequently-occurring chromatin signatures, ChromaSig is divided into two parts. In the first part, we find all loci of width 2-kb that are highly enriched in chromatin modifications, and therefore likely to contain chromatin signatures. But as known chromatin signatures at promoters and enhancers are typically larger than 2-kb [15], these enriched loci are likely part of a larger chromatin signature, which may be found in the vicinity of the enriched locus and oriented on either strand of DNA. Thus, we define a search region of 7-kb around each enriched locus where we search for a chromatin signature motif of size 4-kb. This choice of search region and motif sizes ensures that at least 75% of the enriched locus is covered by the motif. In the second part, ChromaSig clusters, aligns, and orients these enriched loci on the basis of chromatin modifications, using a Euclidean distance measure. A given locus *i* can either align to the motif *M*, the background *B*, or some other motif *M′*. For a given histone mark *h*, the likelihood of accepting locus *i* at location offset *l* and orientation *p* into *M* is given by:
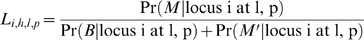
We then employ a greedy algorithm to align and orient each locus *i* to *M* by choosing the *l* and *p* that maximize the following objective function over all members of the motif: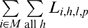.

**Figure 1:**
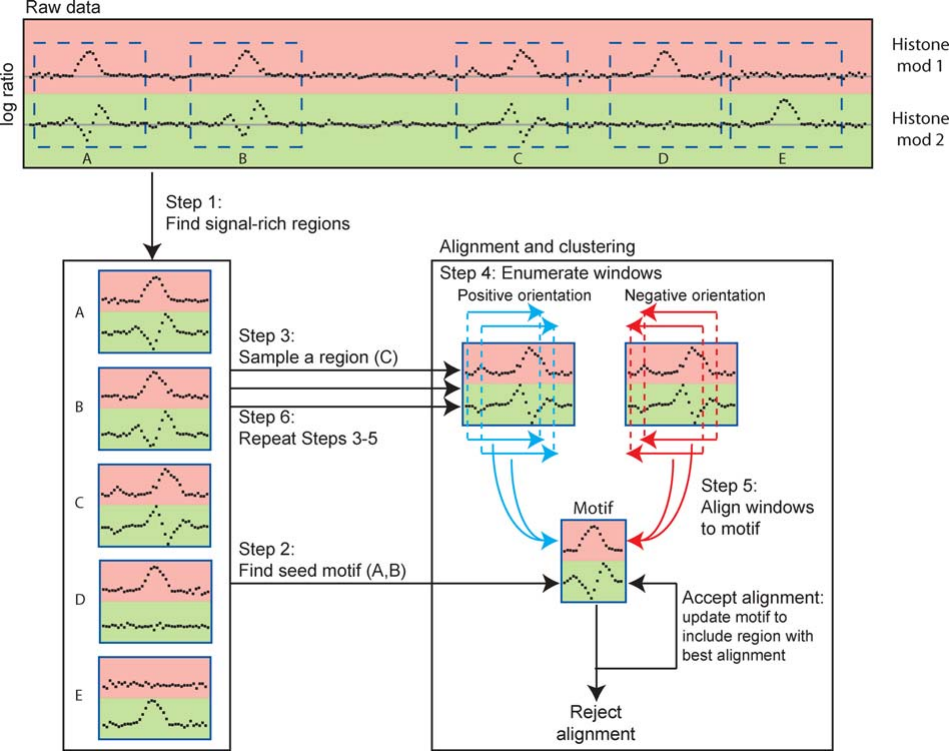
Schematic overview of ChromaSig. In Step 1, we scan genome-scale histone modification maps to find signal-rich loci that potentially contain chromatin signatures. In Step 2, we generate a seed pattern to initialize ChromaSig. In Steps 3 through 5, we visit each enriched locus in turn, enumerate all possible 4-kb windows spanning at least 75% of the locus, and align each window to the seed. This is repeated until each locus has been visited 5 times. Loci that align well to the seed are added to the seed.

Algorithmically, we first define the seed motif by finding a small group of loci sharing a common chromatin signature. We then expand this seed to include other loci, simultaneously refining the motif being searched. Let *D* represent the set of loci already assigned to a motif, initially empty. We sequentially visit each locus not in *D* a total of 5 times. All aligned loci having the motif are output and added to *D*, to be excluded for future rounds of pattern searching. This procedure is repeated with a new seed until no more seeds are found. An overview of the algorithm is given in Scheme 1 and Figure 1.

### Scheme 1: Overview of the ChromaSig

*N* = number of loci

*D* = the set of all assigned loci, initially empty

Repeat while (N≠|D|)

Find a seed motif *M* of loci∉*D* sharing a chromatin signature (described below)

Repeat 5 times

   For each locus *i*∉*D*

       Compute the likelihood of adding *i* into *M*

       Choose to exclude *i* from *M*, or add *i* to *M* in a specific

          location and orientation

   Update *M*

D = D∪M

Print M

### Chromatin Modification Data for ChIP–chip

We use published histone profiles for H4ac, H3ac, H3K4me1, H3K4me2, H3K4me3, and core histone H3 [15] (GEO accession GSE6273), as well as H3K9ac, H3K18ac, and H3K27ac (Heintzman et al., submitted) (GEO accession GSE7118). These data were obtained from ChIP–chip experiments performed in HeLa cells using oligonucleotide tiling arrays spanning the ENCODE regions, a set of 44 genomic regions with a total length of 30 Mbp. We bin the data into 100-bp bins, averaging the probes falling into each bin.

### Finding Loci near Chromatin Signatures

To reduce the search space for finding chromatin signatures, we first focus on enriched loci of width of *w* = 2-kb containing ChIP–chip signals significantly deviating from background. For each histone modification *h*∈1…*H*, let *x_h,i_* be the average log-ratio of bin *i*. After array normalization, *x_h,i_* approximately follows a Gaussian distribution *N*(*μ_h_*, *σ_h_*). To find both histone modification rich and poor loci, we assign a *χ* ^2^ statistic to each locus of size *w* starting at the *j*th bin:
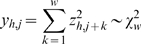
where *z_h,j+k_* = (*x_h,j+k_−μ_h_*)/*σ_h_* is a standard normal variate. We perform the above separately for each histone modification and use a p-value cutoff of 1.0E-5 to assess significant loci. To create a non-redundant list of significant loci over all histone modifications, we represent the score of a locus *j* as the sum of all significant *y_h,j_*. Also, as it is likely that loci adjacent to significant loci will also be significant, we keep a statistically significant high-scoring locus only if all other loci ≤2.5-kb away have a lower score. Finally, we remove all loci poorly represented on the tiling microarray, here defined as containing fewer than 75% of the total number of possible probes in the locus.

### Finding Distinct Chromatin Signatures

The enriched loci above are not grouped by chromatin signature, may not be aligned, and, in the case of asymmetric patterns, may not be in the same orientation. Our goal is to reverse these statements. But first, we begin with some notation. We are given a set of enriched loci from above and a seed motif of width *w_M_* = 4-kb from initialization (described below). For a given locus, we want to determine if it contains the seed motif. But since the loci is not aligned *a priori*, we expand our search to all width *w_M_* windows containing at least 75% of the locus, in both forward and reverse orientations. Thus, we are searching for a 4-kb motif in a 7-kb search region. For simplicity, we allow each locus to contain at most one motif.

ChromaSig refines one motif at a time. The chromatin signature of each motif is defined by the elements belonging to the motif. More specifically, it is defined as: a set of loci {*i*_1_,…*i_j_*,…*i_n_*} that contain the motif, a set of relative locations {*l*_1_…*l_j_*…*l_n_*} where *l_j_* indicates the location offset of the motif in locus *i_j_*, and a set of polarities {*p*_1_…*p_j_*…*p_n_*} where *p_j_* indicates the orientation of the motif in locus *i_j_*. Here, *n* is the total number of loci containing the motif, which can range from 1 to *N* (*N* is the number of loci, which is 1558 here), and *p_j_* can be either “+” indicating the forward orientation or “−” indicating the reverse orientation. Let 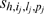 (denoted by *s_h,j_*) be the real-valued sequence of the length *w_M_* window corresponding to locus *i_j_* at location *l_j_* and orientation *p_j_* for histone modification *h*. Let 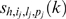 (denoted by *s_h,j_(k)*) be the value of the *k* ^th^ bin in this sequence. Given a seed pattern and a locus *i_j_*, we search over all possible *s_h_*_,*j*_ around *i_j_* for an optimal match to the motif.

We define a seed motif as *m* = {*m*_1_,…,*m_H_*}, where *H* is the number of histone modifications, *h* ranges from 1 to *H,* 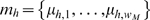, 
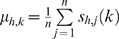
, and *n* is the number of aligned windows. In words, each histone modification *h* has its own length *w_M_* pattern, which is the average of all aligned windows. We define the motif standard deviation similarly:
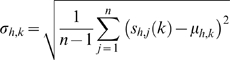



During the sampling step, we choose a locus *i* and attempt to align every length *w_M_* window, at all possible locations *l* and orientations *p*, to the current seed motif. We compute the probability of observing a window's sequence under the motif model as
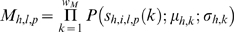
where *P*(*x*;*μ*;*σ*) is a probability defined by dividing the Gaussian probability density function by its maximum value: *P*(*x*;*μ*;*σ*) = exp(−(*x*−*μ*)^2^/(2*σ* ^2^)).

Given a locus to be aligned to the seed, we consider two possibilities: 1) the locus aligns well to the seed and is accepted into the seed, or 2) the locus does not align well and is rejected. In the latter case, the locus may not align well because 2A) the locus matches better to a null background or 2B) the locus matches better to another motif.

To decide between these possibilities, we consider two background models. To consider 2A, we define the null background model by the mean of all bins in the entire ENCODE regions for each histone modification *h* (*μ_h_*) and the mean of the motif standard deviations 
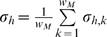
. The probability of observing a window under the null background model is then:
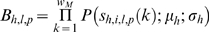



Ideally, we would consider 2B by aligning a locus to all other possible motifs. But since we do not know *a priori* what motifs exist, we model the probability that a window belongs to another motif by:
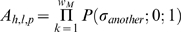
where *σ_another_* is a user-specified parameter (here set to an empirical value of 1.75) that represents the expected quality of the match with another motif, represented as the number of standard deviations from the mean. Larger values of *σ_another_* indicate a looser background model and higher values indicate a more stringent background model.

The *M_h,l,p_* represent the probabilities to add the locus to the seed at a specific location and orientation for a given histone modification, while the *B_h,l,p_* and *A_h,l,p_* represent the probabilities to exclude the locus. To determine which window aligns best to the motif model, we form the likelihood:
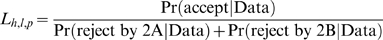



Applying Bayes rule,
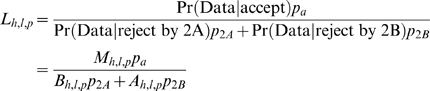
where *p_a_*, *p_2A_*, and *p_2B_* are priors that sum to 1. Here, we let *p_a_* = *p_2B_* and *p_2A_* = 0.01. When *L_h_*_,*l*,*p*_<1, the chance of rejecting a window is greater than accepting it into the motif. If this is true for all *l* and *p* for a given *h*, then there can be no favorable alignment of any window from the given locus to the motif that involves the histone modification *h*. In such a case, we unilaterally reject the locus, regardless of how well other histone modifications align. Otherwise, we find the *l* and *p* that maximizes 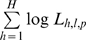, and add this aligned locus into the seed motif.

A cycle is defined to be the process of aligning each locus to the seed motif. At the end of a cycle, we construct a new seed motif containing all accepted windows in their aligned locations and orientations. At the end of 5 cycles, we output the motif and aligned loci belonging to it. To ensure generality of the chromatin signatures, we reject clusters with fewer than 20 elements or clusters having a maximum absolute log-ratio signal less than 0.5.

### Initialization

While most of the loci input to ChromaSig will not be aligned, we do expect that a small number of them will be nearly aligned. To determine the seed motif, we attempt to create seeds starting from 100 randomly chosen enriched loci. For each such locus *i*, we compute the Euclidean distance to all other loci and then use a fast approximate sorting method to find the closest ∼20 loci to *i*, which forms a potential seed. Specifically, we define the leaves of a tree as the loci distances in random order and then construct a tournament tree until there are ≤20 parent nodes. A good seed contains both regions of high signal and low signal, with the members of the seed sharing a very similar chromatin signature. Notably, a seed saturated with signal is uninformative, as it will be difficult to align. We distinguish good seeds by using the following score:
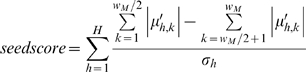
where 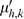 is |*μ_h_*| in descending order. A high seed score indicates a motif with balanced amounts of high and low signal, together with a small standard deviation. We use the seed with the highest score to initialize ChromaSig.

### Application of ChromaSig to Genomewide ChIP-Seq Data

To ensure that ChromaSig is sufficiently general, we also apply it to genomewide distributions of 21 histone marks mapped by ChIP-Seq in CD4+ T cells [18] (Table S2).

- **Data normalization**: We consider only those reads that map uniquely to the genome (hg18) with a maximum of 2 mismatches, and count polyclonal reads once to reduce sequencing bias. We partition the genome into 100-bp bins and count the number of reads in each bin. The number of unique monoclonal reads may be highly variable between different histone marks. For example, there are 15.4 million reads spanning H3K4me3 but only 1.9 million spanning H3K79me2. This vast difference in coverage makes it difficult to compare ChIP enrichment for different histone marks by comparing tag counts. Even for a single mark, sites of true ChIP enrichment can have a large difference in ChIP-Seq tag density [22]. To address these concerns, we normalize the number of reads in each bin *x_h,i_* with a sigmoid function:
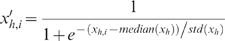
Where median(*x_h_*) and std(*x_h_*) are the median and standard deviation of the number of tags in the 100-bp bins for histone mark *h*, excluding spurious bins containing exactly 0 and 1 reads. By definition, *x′_h,i_* will be 0.5 for bins containing the median number of tags, falls to 0 as tag counts decrease, and saturates to 1 as tag counts increase.
- **Finding ChIP-Seq signal-rich loci**: As we cannot assume a Gaussian distribution of normalized enrichment, we model the background empirically using all 2-kb windows in the ENCODE regions. Furthermore, there are twice as many chromatin marks in the ChIP-Seq dataset compared to the ChIP–chip dataset, and being genomewide the coverage is 100 times higher. To focus on the highest quality loci, we keep a statistically significant high-scoring locus only if all other loci less than 5.0-kb away have a lower score, rather than the 2.5 kb used for ChIP–chip. Furthermore, several chromatin marks including H3K9me3 and H3K36me3 are known to be enriched over large domains. To focus on chromatin signatures smaller than 10-kb, when creating a non-redundant list of significant loci, we only consider those loci *y_h,j_* with p-value smaller than 1E-5 and that are more than 2.5-kb away from any other significant locus in *h*.
- **Motif with pseudocounts**: As ChIP-Seq provides a digital readout of ChIP enrichment, many bins are empty, and it is possible that the motif mean *μ_h,k_* = 0 for some *h* and *k*, which results in *σ_h_*_,*k*_ = 0. To relieve this prohibitive constraint, we add a pseudocount of 0.5 to each position of the motif:
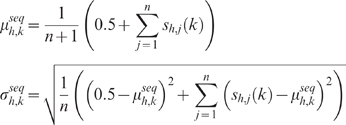
As the number of elements in the motif increases, the contribution of the pseudocount decreases.
- **Parameters**: We run ChromaSig on ChIP-Seq data with the same parameters as for ChIP–chip data. But to focus only on the most frequently-occurring chromatin signatures, we consider only those clusters with an average normalized enrichment greater than 0.25 and with at least 500 loci.

### Software Availability

ChromaSig is open source and freely available at http://bioinformatics-renlab.ucsd.edu/rentrac/wiki/ChromaSig.

## Results

### ChromaSig Identifies Distinct Chromatin Signatures

**Figure 2:**
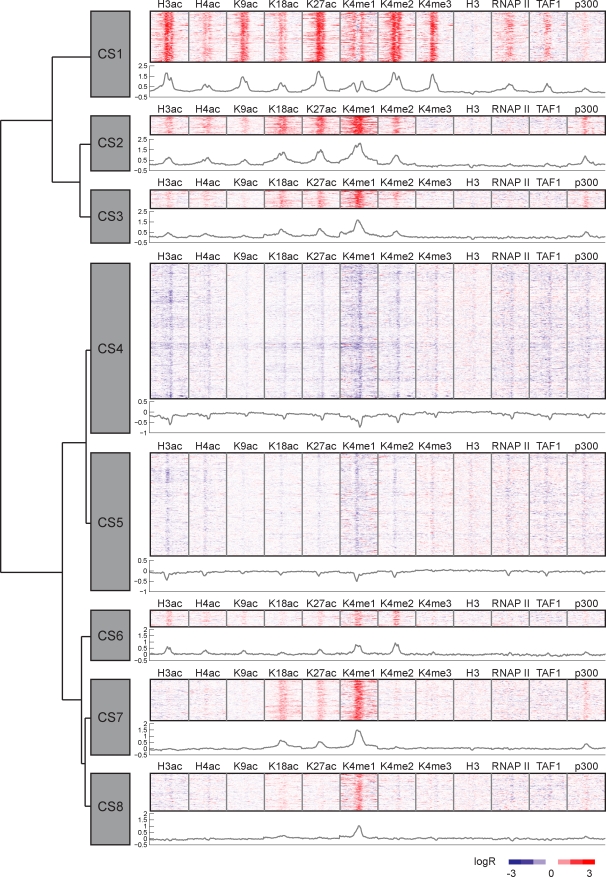
ChromaSig clusters recovered from 9 chromatin marks mapped by ChIP–chip in HeLa cells on ENCODE arrays. Heatmaps (top) and average histone modification profiles (bottom) for each cluster output by ChromaSig. Each horizontal line in the heatmap represents chromatin marks for a single locus. The window size for each mark is 10-kb. Nine histone marks (H4ac, H3ac, H3K9ac, H3K18ac, H3K27ac, H3K4me1, H3K4me2, H3K4me3, and H3) used by ChromaSig and three independent functional marks (RNAPII, TAF250, p300) are presented. To organize these clusters visually, we use hierarchical clustering with a Euclidean distance metric (left).

Starting with ChIP–chip data for H4ac, H3ac, H3K9ac, H3K18ac, H3K27ac, H3K4me1, H3K4me2, H3K4me3, and core histone H3 spanning the ENCODE regions, we first use a sliding window approach to identify signal-rich loci likely to contain histone modification patterns (see Methods, Figure 1). Our goal is to find commonly-occurring patterns in this set of loci. But because this sliding-window approach is quite crude, it is unlikely that the loci will be aligned. Furthermore, a chromatin profile can be observed in two possible orientations corresponding to the two DNA strands running in opposite directions, and the sliding window approach does not account for these orientations. As such, it is unlikely that the collection of signal-rich loci is oriented optimally to preserve asymmetric chromatin signatures, such as those found at promoters [15]. We employ ChromaSig to align and orient these loci into clusters with similar chromatin signatures. Different chromatin signatures can be distinguished by different enrichment of one or more histone modifications, or they may share similar enrichment for all modifications but contain a different enrichment profile for one or more modifications. We find eight clusters spanning 1118 loci (Figure 2 and Table S1).

Loci in the same cluster share the same chromatin signature, and each cluster has a distinct chromatin signature (Figure 2), indicating that the method is functioning as designed. To highlight the similarities and differences of each cluster, we perform hierarchical clustering on the average profiles of each cluster (Figure 1). This reveals that, while some clusters are strikingly distinct from one another, others are only subtly different. On the more distinct side, CS1 is the only cluster to have strong enrichment of H3K4me3, while cluster CS8 is the only cluster to be enriched solely in H3K4me1. More subtly, the chromatin marks present at CS2 and CS3 are the same, but are consistently weaker in CS3 than CS2. Along the same lines, CS6 has narrower and weaker enrichment of H3K4me1 that distinguishes it from the other clusters bearing the H3K4me1 mark. The smallest cluster CS6 contains 44 aligned loci, suggesting that the patterns occur frequently, and may likely be found outside of the ENCODE regions. At the same time, loci in the same cluster also share similar profiles for functional marks (RNAPII, TAF250, p300), which were not the criteria used by ChromaSig. This enrichment of functional marks implies that the clusters group together functionally related genomic loci.

### Comparing ChromaSig Clusters to Clusters from a Supervised Learning Method

To assess the performance of ChromaSig in finding distinct chromatin signatures, we compare ChromaSig signatures to those recovered by a supervised learning approach. Using a training set of chromatin signatures at promoters and enhancers, Heintzman et al. predicted 198 promoters and 389 enhancers [15]. Because their method relied on a sliding window approach that considers aligning chromatin signatures from both strands, each set of predictions should be aligned and oriented. To find distinct clusters of histone modifications on the basis of the nine chromatin marks studied here, we perform k-means clustering on the chromatin modifications near each of these two sets of predictions, giving promoter clusters SP1–4 and enhancer clusters SE1–4 (Figure S1A and S1B).

**Figure 3:**
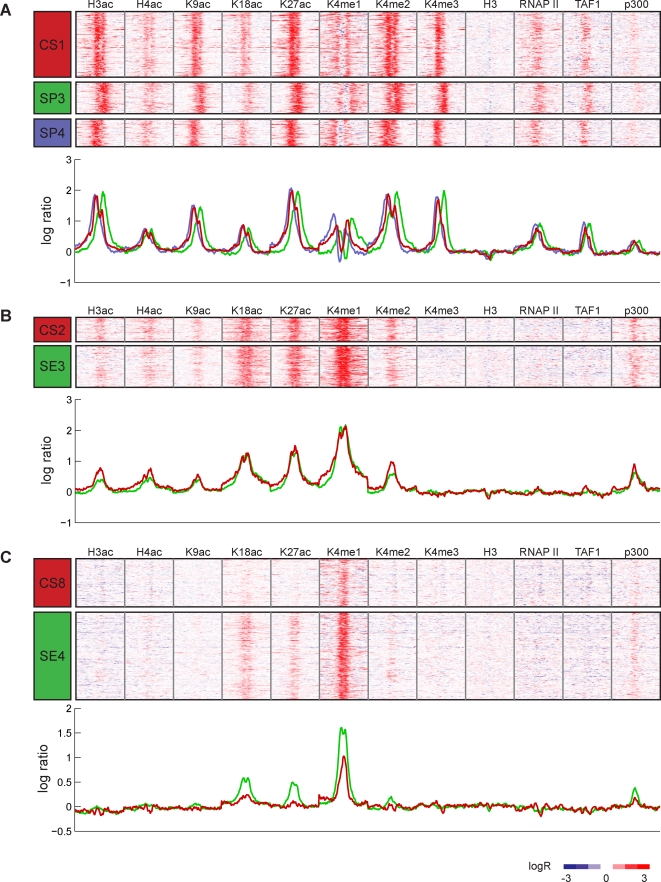
Comparison of ChromaSig to a supervised clustering method from Heintzman et al. [15]. (A) Heatmaps (top) and average histone modification profiles (bottom) for cluster CS1, together with those for SP3 and SP4, which recover CS1 (33.3% recovery by SP3 and 31.1% recovery by SP4). (B) Heatmaps (top) and average histone modification profiles (bottom) for cluster CS2, together with those for SE3, which recovers CS2 (61.2% recovery by SE3). (C) Heatmaps (top) and average histone modification profiles (bottom) for cluster CS8, together with those for SE4, which recovers CS8 (26.5% recovery by SE4). The color of each curve is indicated by the color of the cluster label.

To assess the quality of ChromaSig clustering and alignment, we compare the clusters of predicted enhancers and promoters that recover at least 25% of the loci from each ChromaSig cluster (Figure 3 and Figure S2). The two ChromaSig clusters CS2 and CS7 show striking similarity with clusters SE3 and SE4, respectively (Figure 3B and Figure S2B). Remarkably, even without a training set, ChromaSig employing an unsupervised learning method recovers chromatin signatures found by a supervised learning technique.

This picture changes with ChromaSig cluster CS1, which is recovered by SP3 and SP4. All three of these clusters are enriched with the same chromatin modifications, indicating that the two methods perform similarly, at least at a coarse scale. But interestingly, while the asymmetric patterns SP3 and SP4 are distinct, they appear to be mirror images of each other, and are likely the same pattern observed in opposite directions. Since ChromaSig considers strand orientation in its alignment, cluster CS1 is essentially a merge of these two mirrored clusters, forming a single distinct, consistent, and asymmetric pattern (Figure 3A). Thus, patterns recovered by ChromaSig are less redundant. Also, cluster CS8 contains only H3K4me1 enrichment, and the only cluster that recovers it also contains numerous loci enriched in H3K18ac and H3K27ac (Figure 3C). This, together with the fact that clusters CS4–6 are not recovered by any of clusters SP1–4 and SE1–4, indicate that ChromaSig can find distinct patterns not found by this supervised learning method.

ChromaSig clusters preserve pattern asymmetry, are better aligned, are less redundant, contain loci with more consistent patterns, and contain unique patterns that are not found by the supervised learning method. Most importantly, ChromaSig does not require the construction of training sets, nor does it require the specification of arbitrary parameters such as the number of clusters to find. Instead, ChromaSig finds the natural groupings of the data, creating new clusters as necessary.

### ChromaSig Identifies Known Patterns at Promoters and Enhancers

To date only a handful of distinct histone modification patterns have been broadly associated with specific functions. These include active promoters that are generally marked by the presence of H3K4me3 but absence of H3K4me1, and enhancers marked by the presence of H3K4me1 but absence of H3K4me3 [3],[13],[15]. To assess whether ChromaSig clusters of chromatin signatures correspond to specific biological functions, we first compare them to existing genome annotation.

#### Transcription start sites (TSS)

**Figure 4:**
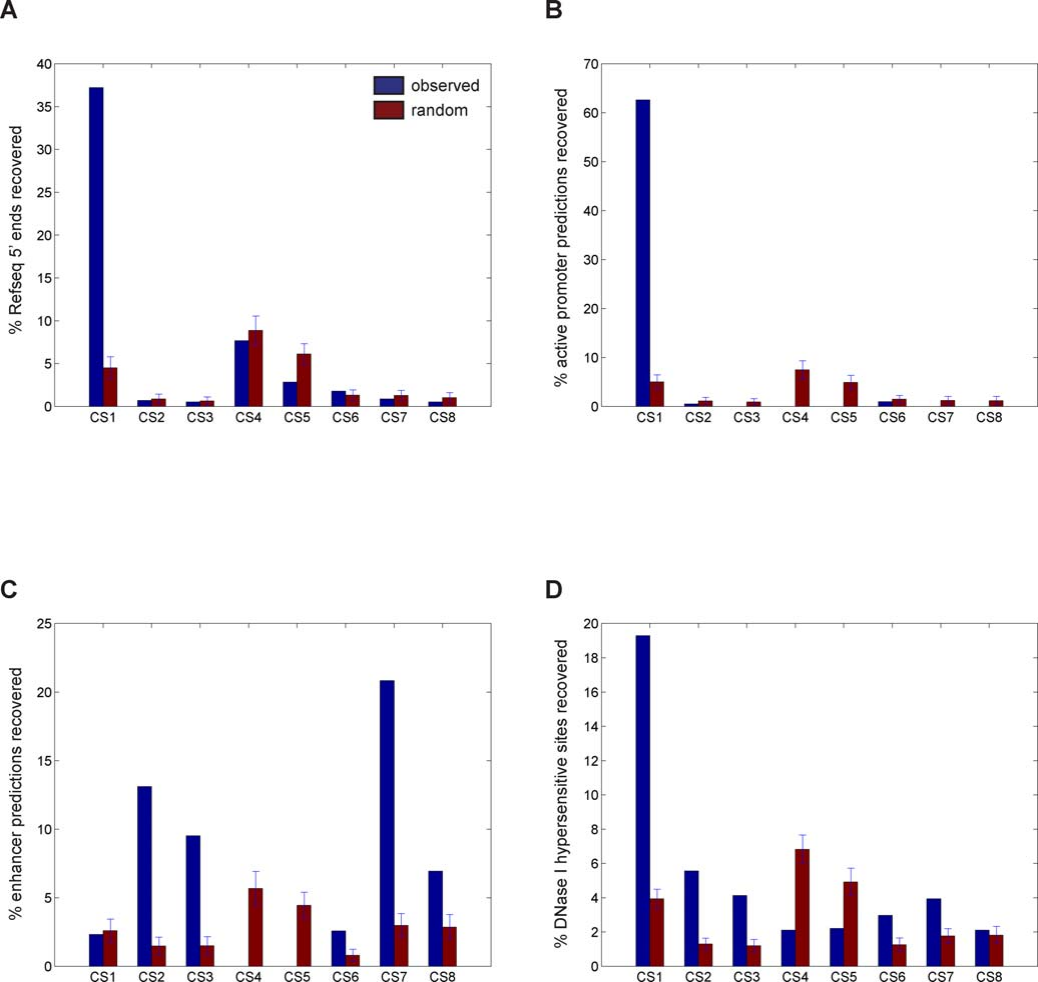
Overlap of ChromaSig clusters with known functional sites in the human ENCODE regions. Percentage of (A) 559 unique Refseq TSSs [23], (B) 198 putative active promoters [15], (C) 389 putative enhancers [15], and (D) 1042 hypersensitive sites [24] that are found within 2.5-kb to the aligned loci, as compared to 100 sets of random sites.

Catalogs of transcription start sites (TSSs) are one of the most abundant and nearly complete annotations for human genomic elements. Of the 559 unique Refseq TSSs [23] in the ENCODE regions, 208 (37.2%) are proximal (hereafter defined as within 2.5-kb) to cluster CS1, far more than any other cluster (Figure 4A). To assess the significance of this overlap, we compare with 100 random sets of clusters of the same size, sampled from regions on the ChIP–chip array to avoid biases from probe-poor regions, giving a *p*-value of 3.2E-141 assuming a Gaussian distribution. The majority of Refseq TSSs are not recovered, as roughly half of them do not contain enrichment of these histone modifications [15].

#### Promoter and enhancer predictions

Heintzman et al. use the same dataset but with a supervised learning approach to predict active promoters and enhancers [15]. A majority (62.6%, *p*<1.0E-300) of the predicted active promoters are proximal to cluster CS1 (Figure 4B). In addition, the enhancer predictions generally fall into clusters CS2–3 and CS6–8 (Figure 4C). These results indicate that cluster CS1 is highly enriched in promoters containing the active chromatin marks, while clusters CS2–3 and CS6–8 are enriched in HeLa-marked enhancers.

#### DNase I hypersensitivity (HS) sites

DNase I hypersensitivity is a hallmark for many types of *cis*-regulatory elements. Using a list of putative HS sites found from high-throughput, high resolution DNase-chip experiments [24], we find significant enrichment of HS sites at clusters CS1 (*p* = 6.7E-165), CS2–3 (*p_CS2_* = 8.4E-36, *p_CS3_* = 7.3E-16), and CS6–7 (*p_CS6_* = 7.1E-6, *p_CS7_* = 2.5E-7) (Figure 4D), consistent with their proposed function as promoters and enhancers. On the other hand, clusters CS4–5 shows marked depletion of HS sites (*p_CS4_* = 9.7E-9, *p_CS5_* = 3.7E-4).

### Distinct Chromatin Signatures Associated with Distinct Functions

We recover several distinct chromatin signatures associated with predicted HeLa enhancers. CS8 is only enriched in H3K4me1, while CS7 also contains H3K18ac and H3K27ac enrichment. In addition to these marks, clusters CS2–3 also have H3K4me2 enrichment, with CS2 being more acetylated than CS3. As the remaining cluster CS6 is the only one to have less than 25% of its loci recovered by predicted enhancers and also has the weakest enrichment of the enhancer hallmark H3K4me1, it may contain loci other than enhancers and we exclude CS6 from this analysis.

**Figure 5:**
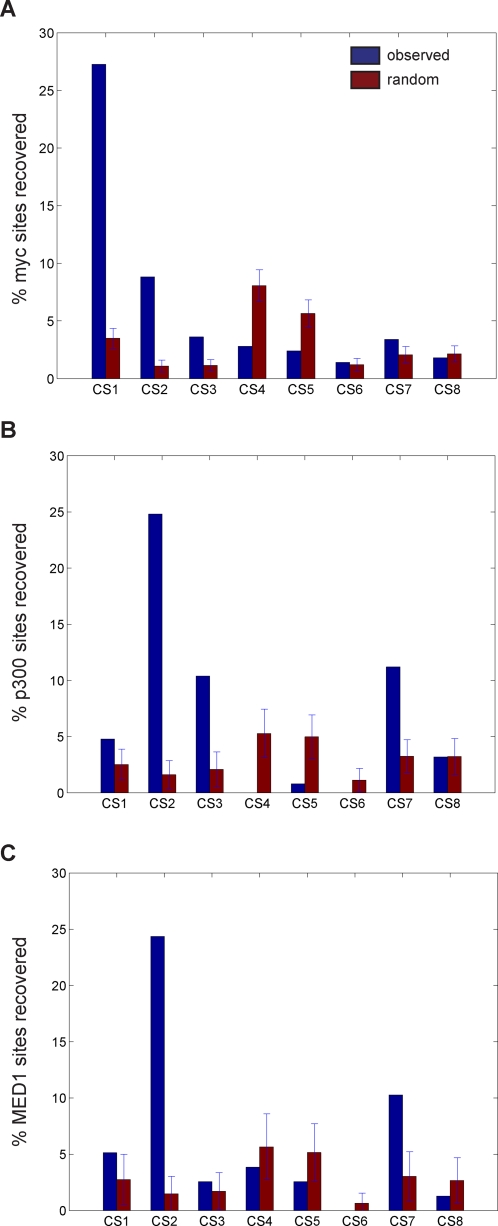
Overlap of ChromaSig clusters with transcription factors and coactivators mapped in HeLa cells in the ENCODE regions. Percentage of (A) 499 c-Myc [35], (B) 125 p300 [15], and (C) 78 MED1 [15] binding sites found within 2.5-kb to aligned clusters, as compared to 100 sets of random sites.

If the histone code hypothesis is true, we would expect functional differences between enhancers marked by different signatures. To assess if the different enhancer-like clusters also have distinct functional roles, we examine enrichment in binding sites for a variety of transcription factors and co-activators mapped in HeLa. We notice that binding sites for the transcription factor c-Myc is significantly enriched at clusters CS2 and CS3 (*p_CS2_* = 4.6E-50, *p_CS3_* = 3.6E-7) (Figure 5A). Visually comparing the chromatin modifications at these clusters which have c-Myc enrichment to clusters CS7–8 that lack c-Myc enrichment, we observe that CS2–3 have enrichment of H3ac, H4ac, and H3K4me2, while these chromatin marks are absent in E3–4. Thus, one of these marks may be important to c-Myc function. In contrast, the co-activator p300 is highly enriched at clusters CS2, CS3, and CS7 (*p_CS2_* = 1.5E-75, *p_CS3_* = 4.1E-8, *p_CS7_* = 3.3E-8) (Figure 5B). Strikingly, the only cluster lacking p300 enrichment, CS8, is also the only cluster to lack enrichment of H3K18ac and H3K27ac, suggesting a connection between these chromatin marks and p300 activity. Finally, binding sites for a different co-activator MED1 are only enriched at clusters CS2 and CS7 (*p_CS2_* = 5.4E-50, *p_CS7_* = 4.9E-4) (Figure 5C), distinct from binding of p300 and c-Myc. These results suggest that enhancers marked by different chromatin signatures have unique functional roles dictated by distinct protein complexes.

### ChromaSig Identifies Other Potential Regulatory Sequences

**Figure 6:**
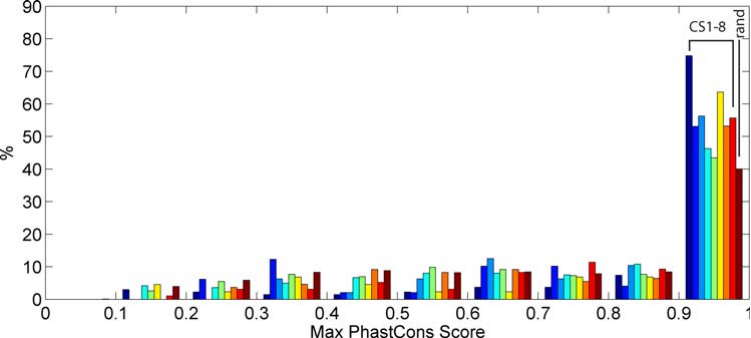
ChromaSig clusters are evolutionarily conserved. Distribution of maximum PhastCons conservation scores [25] over a 1-kb window centered at the aligned loci, as compared to 10000 random sites.

Outside of promoters and enhancers, current knowledge on common histone modification patterns is sparse. ChromaSig identifies two novel signatures CS4–5 marking sites of unknown function, as well as CS6 which is only slightly recovered by enhancer predictions. To assess the possible functional significance of these genomic sites, we first analyze sequence conservation. Here, we use PhastCons scores from multiple alignments of 7 vertebrate genomes (chimp, mouse, rat, dog, chicken, fugu, and zebrafish) and human [25] to determine the amount of between-species conservation of each cluster (Figure 6). Conservation scores for clusters CS4–6 are generally significantly greater than that expected at random (*p*_CS4_ = 9.6E-5, *p*_CS5_ = 7.8E-2, *p*_CS6_ = 1.6E-3, as assessed by the Wilcoxon signed rank test compared to 10000 random sites). Turning to RegPot, which scores the regulatory potential of regions in the human genome, we find that these clusters also have greater regulatory potential than that expected at random (*p*_CS4_ = 3.5E-11, *p*_CS5_ = 2.1E-2, *p*_CS6_ = 1.6E-7). Together, these results suggest clusters CS4–6 contain biologically functional loci.

**Figure 7:**
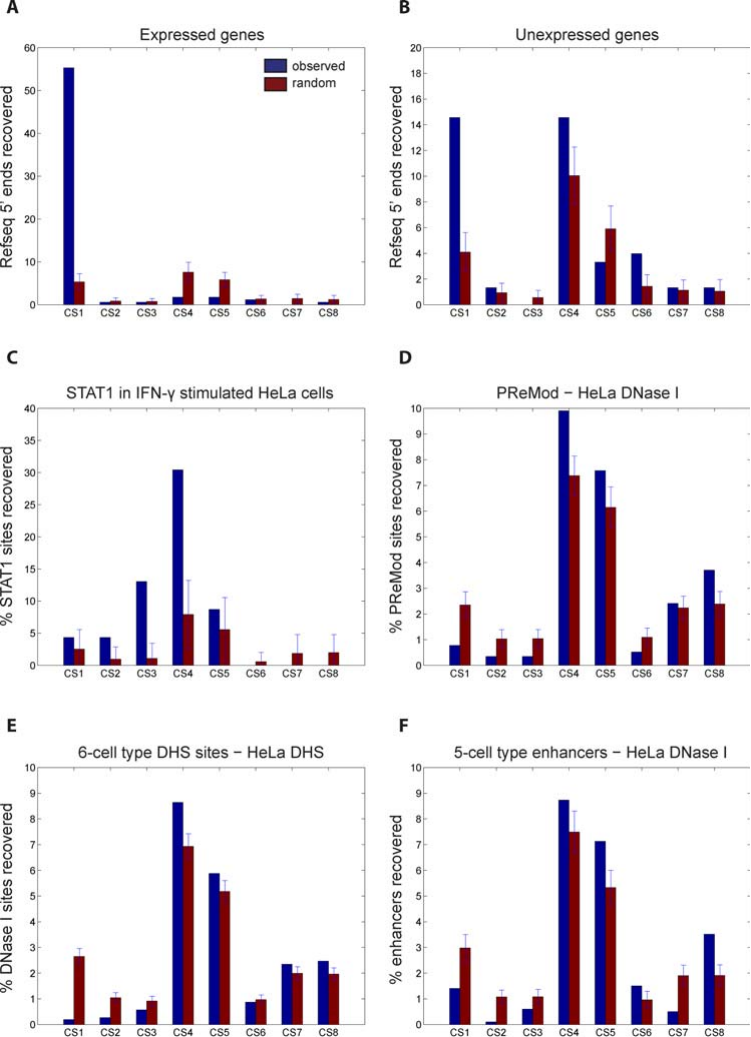
Clusters CS4–5 contain regulatory elements. Percentage of the (A) promoters from expressed genes, (B) promoters from unexpressed genes, and (C) STAT1 binding sites in IFN-γ treated HeLa cells that are within 2.5-kb of the aligned loci. Percentage of (D) PReMod sites [26], (E) combined 6-cell type HS sites [24],[27], and (F) combined 5-cell type enhancer predictions distal to HeLa HS sites that are within 2.5-kb of aligned loci. All overlaps are compared to 100 sets of random sites.

Clusters CS4–5 are generally depleted of all histone modifications, as well as the functional marks RNAP II, TAF1, and p300 (Figure 2). The overlap of cluster CS4 at Refseq TSSs (Figure 4A) and the lack of overlap at active promoters (Figure 4B) suggest that some CS4 sites may contain inactive TSSs. To assess this, we examine enrichment of clusters at promoters of expressed and unexpressed genes (Figure 7A and 7B). We observe depletion of clusters CS4–5 at the 5′ ends of expressed genes (p_CS4_ = 7.5E-4, p_CS4_ = 1.6E-2), and CS4 is actually enriched at promoters of unexpressed genes (p_CS4_ = 2.4E-2). Thus, some members of CS4 may be inactive promoters. We also observe significant enrichment of cluster CS6 at promoters of unexpressed genes (p = 1.7E-3) (Figure 7B). This suggests that, in addition to containing enhancers, this cluster of evolutionarily conserved sequences that are marked by HS in HeLa cells may also contain inactive promoters.

As the majority of clusters CS4–5 are not explained by promoters, we next ask if these clusters recover other distal regulatory elements. The depletion of HeLa HS sites in CS4–5 (Figure 4D) suggests that these clusters should also be depleted of transcription factor binding sites (TFBSs). But when we examine the overlap with STAT1 binding sites in HeLa cells treated with IFN-γ (Heintzman et al., in submission), we observe striking enrichment with cluster CS4 (*p* = 5.4E-5) (Figure 7C). Interestingly, while ChromaSig clusters are derived from HeLa chromatin profiles, the STAT1 overlap occurs in a different cellular context, suggesting that cluster CS4 may harbor TFBSs not bound in HeLa cells.

**Figure 8:**
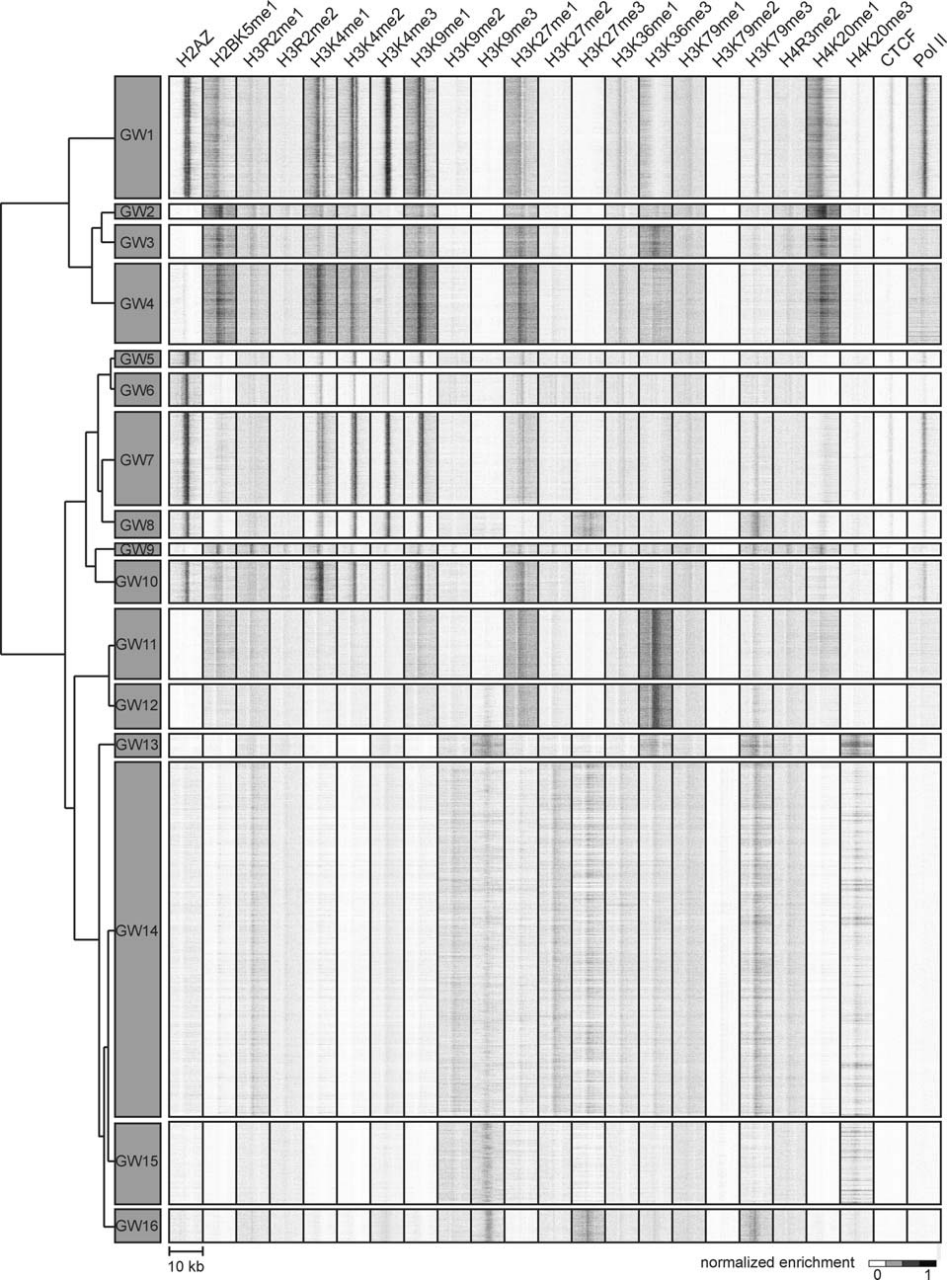
ChromaSig clusters recovered from 21 histone marks mapped by ChIP-Seq in CD4+ T cells genomewide. ChromaSig recovers 16 clusters spanning 49340 genomic loci. Each cluster is represented by a heatmap summarizing ChIP-Seq enrichment for all loci in the cluster. The window size for each mark is 10-kb. To organize these clusters visually, we use hierarchical clustering with a Euclidean distance metric (left).

The PreMod database [26] contains 1655 putative conserved TF modules in the ENCODE regions. As PreMod is determined by static sequence data, its sites represent TFBSs under various cellular conditions. To test the hypothesis that clusters CS4–5 mark TFBSs not bound in HeLa cells, we test the enrichment of these clusters at PreMod sites distal to HeLa HS sites. Interestingly, we find that CS4 members are enriched in these sites (*p*_CS4_ = 7.6E-5), suggesting that this cluster contains sites that potentially bind TFs, but not in HeLa cells (Figure 7D). As an independent method to support this result, we combine HS sites previously mapped in six non-HeLa cell lines [24],[27]. Removing those sites near HeLa HS sites, we find significant enrichment with clusters CS4 and CS5 (*p*_CS4_ = 1.4E-4, *p*_CS4_ = 3.0E-2) (Figure 8E). Finally, we compare clusters CS4–5 with enhancers predicted in four cell types (Heintzman et al., in submission), using our previously published chromatin signature-based method [15]. Of those enhancers not marked by HS in HeLa cells, we observe significant enrichment at clusters CS4–5 (*p*_CS4_ = 3.7E-2, *p*_CS5_ = 7.7E-3) (Figure 7F). Together, these results suggest that ChromaSig clusters having novel chromatin signatures also contain regulatory sequences.

### ChromaSig Identifies Distinct Chromatin Signatures in Genomewide ChIP-Seq Data

So far, we have shown that ChromaSig can find distinct chromatin signatures using ChIP–chip data spanning the ENCODE regions. But the question remains as to whether ChromaSig is applicable on a genomewide level or on ChIP-Seq data from next-generation sequencing. To address this, we focus on a recently published study by Barski et al. which used ChIP-Seq to map the genomewide distributions of 21 chromatin marks in CD4+ T cells [18]. We identify 16 clusters containing distinct chromatin signatures spanning 49340 genomic loci (Figure 8). Using hierarchical clustering with a Euclidean distance measure to categorize the average profiles of each cluster reveals that there are essentially two main categories of genomic elements. One class, GW1–10, contains combination of the activating marks H3K4me1/2/3 and H2BK5me1. Another class, GW11–16, are more prevalently marked by the repressive marks H3K9me3, H3K27me3, and H3K36me3, and H3K79me3.

**Figure 9:**
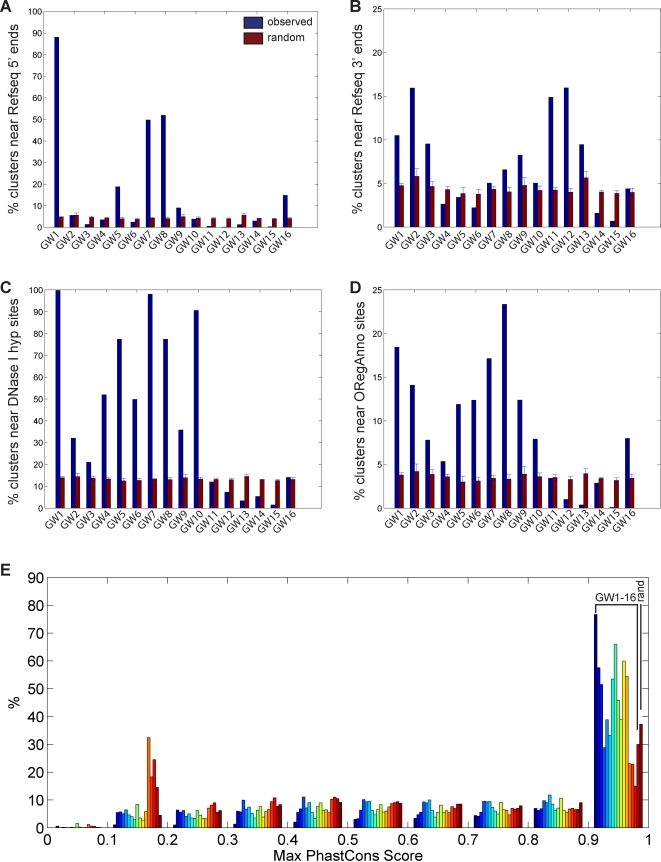
Overlap of genomewide clusters with known annotations. Percentage of each cluster within 2.5-kb of (A) 21211 Refseq 5′ ends [23], (B) 20754 Refseq 3′ ends [23], (C) 95709 DNase I hypersensitive sites mapped in CD4+ T cells [29], and (D) 21959 regulatory sites from the ORegAnno database [30], as compared to 100 sets of random sites. (E) Distribution of maximum PhastCons scores [25] over a 1-kb window centered at ChromaSig aligned sites, as compared to 10000 random sites.

There are 5 clusters significantly enriched for promoters (Figure 9A), each with a distinct combination of chromatin marks. To assess significance, we compare with 100 random sets of clusters of the same size, sampled from non-repeat masked regions of the genome. In addition to being the only promoter cluster enriched in H4K20me1, GW1 contains the strongest enrichment of H3K4me3 with a corresponding wide valley of H3K4me1 enrichment, in contrast to GW7 which has weaker H3K4me3 enrichment followed by a narrower H3K4me1 enrichment profile and GW5 which contains even weaker enrichment of these marks. Of the remaining promoter-associated clusters, GW8 contains “bivalent” promoters enriched in active H3K4me3 and repressive H3K27me3 marks [28], while GW16 is mainly enriched in the repressive marks H3K9me3, H3K27me3, and H3K79me3.

Enrichment of H3K36me3 has been associated with the 3′ ends of highly expressed genes [18]. Consistent with this, we observe that GW11–12, which contain the strongest enrichment of H3K36me3, are also enriched at Refseq 3′-ends (Figure 9B). While the vast majority of histone modifications at these two clusters are similar, it is also clear that GW11 is more enriched in H3K9me1 and H4K20me1 than GW12.

Recently, Boyle et al. mapped DNase I hypersensitive sites genomewide in CD4+ T cells [29]. Here, we observe that clusters GW1–10, which generally contain active marks, are all enriched in DHS sites. In contrast, the remaining clusters GW11–16 marked by repressive marks all lack DHS enrichment (Figure 9C). Thus, GW1–10 likely contain regulatory elements functioning in CD4+ T cells. Mirroring this observation, clusters GW1–10 are also generally enriched in known regulatory elements as annotated by ORegAnno [30] (Figure 9D).

This analysis reveals possible functional roles for GW1–12 and GW16. Like these clusters, each remaining cluster contains loci that share a consistent chromatin signature, suggesting that each cluster contains loci that may function similarly. Interestingly, GW13–16 are all consistently marked by repressive chromatin marks, and in particular the heterochromatin mark H3K9me3. But unlike large domains of heterochromatin, GW13–16 appear to be localized to small heterochromatic loci spanning less than 5 kb. To assess possible functionality for GW13–15, we next turn to sequence conservation. Surprisingly, these clusters and GW16 are actually less conserved than expected at random (*p*<1e-15) (Figure 9E). Thus, GW13–16 contain quickly evolving but consistently marked, locally heterochromatic regions of the genome, though their specific functions remain unknown.

## Discussion

Large-scale maps of histone modifications provide a global view of epigenetic status and allow us to investigate the influence of epigenetics in development and disease. Thanks to the development of large scale experimental approaches including ChIP–chip and ChIP-seq [16],[31], datasets of histone modification profiles are rapidly accumulating. However, while numerous methods have been developed to identify the binding locations of transcription factors (TFs) from these data [19]–[21],[32], methods for analysis of histone modification profiles are still lacking due to unique challenges that have not been encountered with TF data. Binding sites for TFs are generally discrete peaks and are sparsely scattered throughout the genome [19], whereas histone modifications are often repeated over many consecutive nucleosomes [1],[3]. As such, finding regions of interest in a histone modification landscape is quite different from finding TF hits. While using standard peak-finding on histone modifications is possible, this approach suffers from several drawbacks. First, peak-finding ignores loci depleted of binding signal, which can be important in mapping nucleosome-depleted regions [15]. Second, analysis of histone modification data is focused on identifying a specific pattern in regions often spanning thousands of base pairs (bps) while peak finding for TFs is generally focused on much smaller regions. Third, peak finding ignores the binding profile's orientation, but the orientation of asymmetric histone patterns can be quite functionally revealing [13],[15]. Finally, peak-finding treats different proteins independently, ignoring the correlation of different histone modifications, and thereby reducing the likelihood of discovering novel biological insights from the combinatorial presence of multiple histone modifications [13],[15].

In this study, we introduce a strategy called ChromaSig to find commonly occurring chromatin signatures given a landscape of histone modification profiles. Using an unsupervised learning approach, ChromaSig simultaneously clusters, aligns, and orients chromatin signatures without using any training sets or external annotations. Using histone modification data alone, ChromaSig is able to distinguish subtle differences in chromatin signatures, allowing it to find natural groupings of the data without relying explicitly on heavily constraining parameters such as the number of expected clusters, which can severely hamper pattern discovery. Interestingly, even with this limited input, ChromaSig recovers chromatin signatures similar to a previously published supervised learning method that used high-quality curated training sets. In addition to discovering new chromatin signatures, the ChromaSig clusters preserve pattern asymmetry, are better aligned, and are less redundant.

ChromaSig is sensitive enough to recover known histone modification patterns for active promoters and enhancers. This recovery of known patterns further suggests that the novel patterns are real. Our method is also able to clearly distinguish between different classes of enhancers based on chromatin modifications. Interestingly, we find that different functional activities of associated with enhancers, such as binding of specific co-activators and transcription factors, are linked to specific histone modifications present at the enhancers. While the mechanism for this phenomenon is unclear and will require further study, it is tempting to speculate that additional maps of chromatin marks and transcription factors in HeLa cells may uncover more specific classes of enhancer chromatin signatures associated with more specific functions, lending further support to the histone code hypothesis. This phenomenon may also occur at other genomic elements such as promoters and insulators.

ChromaSig also recovers several novel clusters CS4–5, which are simultaneously depleted of 9 chromatin modification marks and 3 general transcription factors. Such depletion may correspond to special chromatin structures that are generally resistant to immunoprecipitation. Indeed, depletion of ChIP/Input signals at these loci is also observed in independent ChIP–chip experiments against STAT1, c-Myc and other transcription factors using HeLa S3 cells [15],^33^. However, we find that these sites contain evolutionarily conserved sequences and are enriched in inactive promoters and TFBSs. These observations suggest that clusters CS4–5 contain potential regulatory elements.

Application of ChromaSig genomewide recovers only 16 distinct chromatin signatures. With the 21 different histone modifications studied here, the number of different possible combinations is 2ˆ21. Strikingly, ChromaSig reveals that the number of frequently-occurring histone modifications is actually quite small in humans, a result mirrored in yeast [13], and some chromatin signatures occur much more frequently than others. Notably, GW1–10 are all enriched in DNase I hypersensitive sites, indicating they are likely to mark function genomic elements in CD4+ T cells. Of these, GW1/5/7/8 are highly enriched in H3K4me3, and consistent with this, are also enriched in promoters. The remaining hypersensitive clusters are enriched in known regulatory elements, some of which may be enhancers. Consistent with this, many of these clusters contain stronger enrichment of H3K4me1 than H3K4me3. Extending from our results focused on the ENCODE regions, we hypothesize that these different clusters are bound by a different combination of transcription factors and co-activators.

In recent years, numerous studies have used the genome sequence, along with high-throughput expression and transcription factor ChIP data, to characterize regulatory elements [21],[34]. As the epigenetic code offers an abstraction over the genetic code, using it alone may be viable in the study of some functional genomic elements – including genes, enhancers, repressors, insulators, and other regulatory elements. As the availability of large-scale data for chromatin marks increases, the ability of methods such as the one presented here to concisely describe the underlying chromatin signatures, thereby abstracting away irrelevant or redundant data, will become increasingly more critical. Future efforts to unify both epigenetic and genetic content will be quite powerful in further identifying and characterizing regulatory elements that have eluded current methods.

## Supporting Information

Figure S1 Heatmaps of promoter and enhancer predictions from Heintzman et al. [15]. Heatmaps of chromatin modifications and functional marks found at (A) promoter and (B) enhancer predictions, after performing *k*-means clustering on the nine chromatin marks (*k* = 4).
(6.16 MB TIF)

Figure S2 Comparison of ChromaSig clusters to clusters from Heintzman et al. [15]. Heatmaps (top) and average histone modification profiles (bottom) for ChromaSig clusters (A) CS3 and (B) CS7, together with those clusters in Heintzman et al. which recover the ChromaSig clusters. Comparisons for CS1–2 and CS8 can be found in Figure 3. Clusters CS4–6 are not recovered by Heintzman et al. clusters. The color of each curve is indicated by the color of the cluster label.
(6.36 MB TIF)

Table S1 ENCODE clusters in HeLa cells. Locations and orientations of each predicted element (hg17), after applying ChromaSig to 9 histone marks mapped by ChIP–chip in HeLa cells on ENCODE arrays.
(0.03 MB DOC)

Table S2 Genomewide clusters in CD4+ T cells. Locations and orientations of each predicted element (hg18), after applying ChromaSig to 21 histone marks mapped by ChIP-Seq in CD4+ T cells genomewide.
(1.52 MB RTF)

## References

[1] Millar CB, Grunstein M (2006). Genome-wide patterns of histone modifications in yeast.. Nat Rev Mol Cell Biol.

[2] Segal E, Fondufe-Mittendorf Y, Chen L, Thastrom A, Field Y (2006). A genomic code for nucleosome positioning.. Nature.

[3] Roh TY, Cuddapah S, Cui K, Zhao K (2006). The genomic landscape of histone modifications in human T cells.. Proc Natl Acad Sci U S A.

[4] Grant PA (2001). A tale of histone modifications.. Genome Biol.

[5] Wang H, Wang L, Erdjument-Bromage H, Vidal M, Tempst P (2004). Role of histone H2A ubiquitination in Polycomb silencing.. Nature.

[6] Nathan D, Ingvarsdottir K, Sterner DE, Bylebyl GR, Dokmanovic M (2006). Histone sumoylation is a negative regulator in Saccharomyces cerevisiae and shows dynamic interplay with positive-acting histone modifications.. Genes Dev.

[7] Sims RJ, Reinberg D (2006). Histone H3 Lys 4 methylation: caught in a bind?. Genes Dev.

[8] Kim TH, Barrera LO, Qu C, Van Calcar S, Trinklein ND (2005). Direct isolation and identification of promoters in the human genome.. Genome Res.

[9] Kim TH, Barrera LO, Zheng M, Qu C, Singer MA (2005). A high-resolution map of active promoters in the human genome.. Nature.

[10] Bergink S, Salomons FA, Hoogstraten D, Groothuis TA, de Waard H (2006). DNA damage triggers nucleotide excision repair-dependent monoubiquitylation of histone H2A.. Genes Dev.

[11] Cimini D, Mattiuzzo M, Torosantucci L, Degrassi F (2003). Histone hyperacetylation in mitosis prevents sister chromatid separation and produces chromosome segregation defects.. Mol Biol Cell.

[12] Jenuwein T, Allis CD (2001). Translating the histone code.. Science.

[13] Liu CL, Kaplan T, Kim M, Buratowski S, Schreiber SL (2005). Single-nucleosome mapping of histone modifications in S. cerevisiae.. PLoS Biol.

[14] Pokholok DK, Harbison CT, Levine S, Cole M, Hannett NM (2005). Genome-wide map of nucleosome acetylation and methylation in yeast.. Cell.

[15] Heintzman ND, Stuart RK, Hon G, Fu Y, Ching CW (2007). Distinct and predictive chromatin signatures of transcriptional promoters and enhancers in the human genome.. Nat Genet.

[16] Ren B, Robert F, Wyrick JJ, Aparicio O, Jennings EG (2000). Genome-wide location and function of DNA binding proteins.. Science.

[17] Iyer VR, Horak CE, Scafe CS, Botstein D, Snyder M (2001). Genomic binding sites of the yeast cell-cycle transcription factors SBF and MBF.. Nature.

[18] Barski A, Cuddapah S, Cui K, Roh TY, Schones DE (2007). High-resolution profiling of histone methylations in the human genome.. Cell.

[19] Zheng M, Barrera LO, Ren B, Wu YN (2005). ChIP–chip: Data, Model, and Analysis.

[20] Johnson WE, Li W, Meyer CA, Gottardo R, Carroll JS (2006). Model-based analysis of tiling-arrays for ChIP–chip.. Proc Natl Acad Sci U S A.

[21] Benner CFGA, Subramaniam S, Glass C (2006). HOMER: An Algorithm for the De Novo Discovery of cis-Regulatory Elements from High Throughput Data.

[22] Johnson DS, Mortazavi A, Myers RM, Wold B (2007). Genome-wide mapping of in vivo protein-DNA interactions.. Science.

[23] Pruitt KD, Tatusova T, Maglott DR (2005). NCBI Reference Sequence (RefSeq): a curated non-redundant sequence database of genomes, transcripts and proteins.. Nucleic Acids Res.

[24] Crawford GE, Davis S, Scacheri PC, Renaud G, Halawi MJ (2006). DNase-chip: a high-resolution method to identify DNase I hypersensitive sites using tiled microarrays.. Nat Methods.

[25] Siepel A, Bejerano G, Pedersen JS, Hinrichs AS, Hou M (2005). Evolutionarily conserved elements in vertebrate, insect, worm, and yeast genomes.. Genome Res.

[26] Blanchette M, Bataille AR, Chen X, Poitras C, Laganiere J (2006). Genome-wide computational prediction of transcriptional regulatory modules reveals new insights into human gene expression.. Genome Res.

[27] Xi H, Shulha HP, Lin JM, Vales TR, Fu Y (2007). Identification and characterization of cell type-specific and ubiquitous chromatin regulatory structures in the human genome.. PLoS Genet.

[28] Bernstein BE, Mikkelsen TS, Xie X, Kamal M, Huebert DJ (2006). A bivalent chromatin structure marks key developmental genes in embryonic stem cells.. Cell.

[29] Boyle AP, Davis S, Shulha HP, Meltzer P, Margulies EH (2008). High-resolution mapping and characterization of open chromatin across the genome.. Cell.

[30] Montgomery SB, Griffith OL, Sleumer MC, Bergman CM, Bilenky M (2006). ORegAnno: an open access database and curation system for literature-derived promoters, transcription factor binding sites and regulatory variation.. Bioinformatics.

[31] Wei CL, Wu Q, Vega VB, Chiu KP, Ng P (2006). A global map of p53 transcription-factor binding sites in the human genome.. Cell.

[32] Qi Y, Rolfe A, MacIsaac KD, Gerber GK, Pokholok D (2006). High-resolution computational models of genome binding events.. Nat Biotechnol.

[33] ENCODE_Consortium (2004). The ENCODE (ENCyclopedia Of DNA Elements) Project.. Science.

[34] Wang W, Cherry JM, Nochomovitz Y, Jolly E, Botstein D (2005). Inference of combinatorial regulation in yeast transcriptional networks: a case study of sporulation.. Proc Natl Acad Sci U S A.

[35] Kim J, Bhinge AA, Morgan XC, Iyer VR (2005). Mapping DNA-protein interactions in large genomes by sequence tag analysis of genomic enrichment.. Nat Methods.

