# Supplementary figures and images for: ChromaSig: A Probabilistic Approach to Finding Common Chromatin Signatures in the Human Genome
Source: PLoS Comput Biol. 2008 Oct 17;4(10):e1000201. doi: 10.1371/journal.pcbi.1000201 (PMC2556089; doi:10.1371/journal.pcbi.1000201)

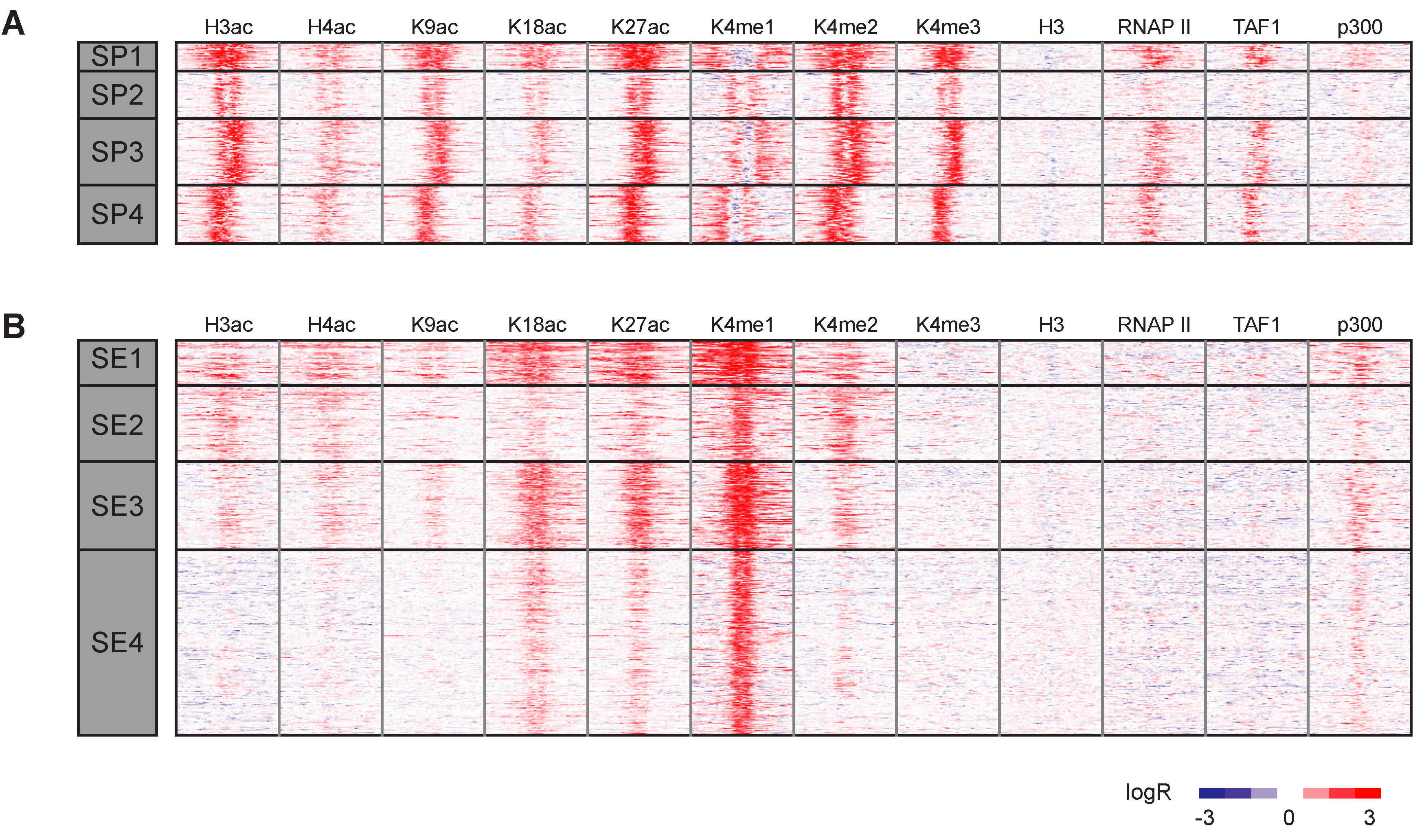

Supplement: Figure S1 — Heatmaps of promoter and enhancer predictions from Heintzman et al. [15]. Heatmaps of chromatin modifications and functional marks found at (A) promoter and (B) enhancer predictions, after performing k-means clustering on the nine chromatin marks (k = 4). (6.16 MB TIF) [file pcbi.1000201.s001.tif]

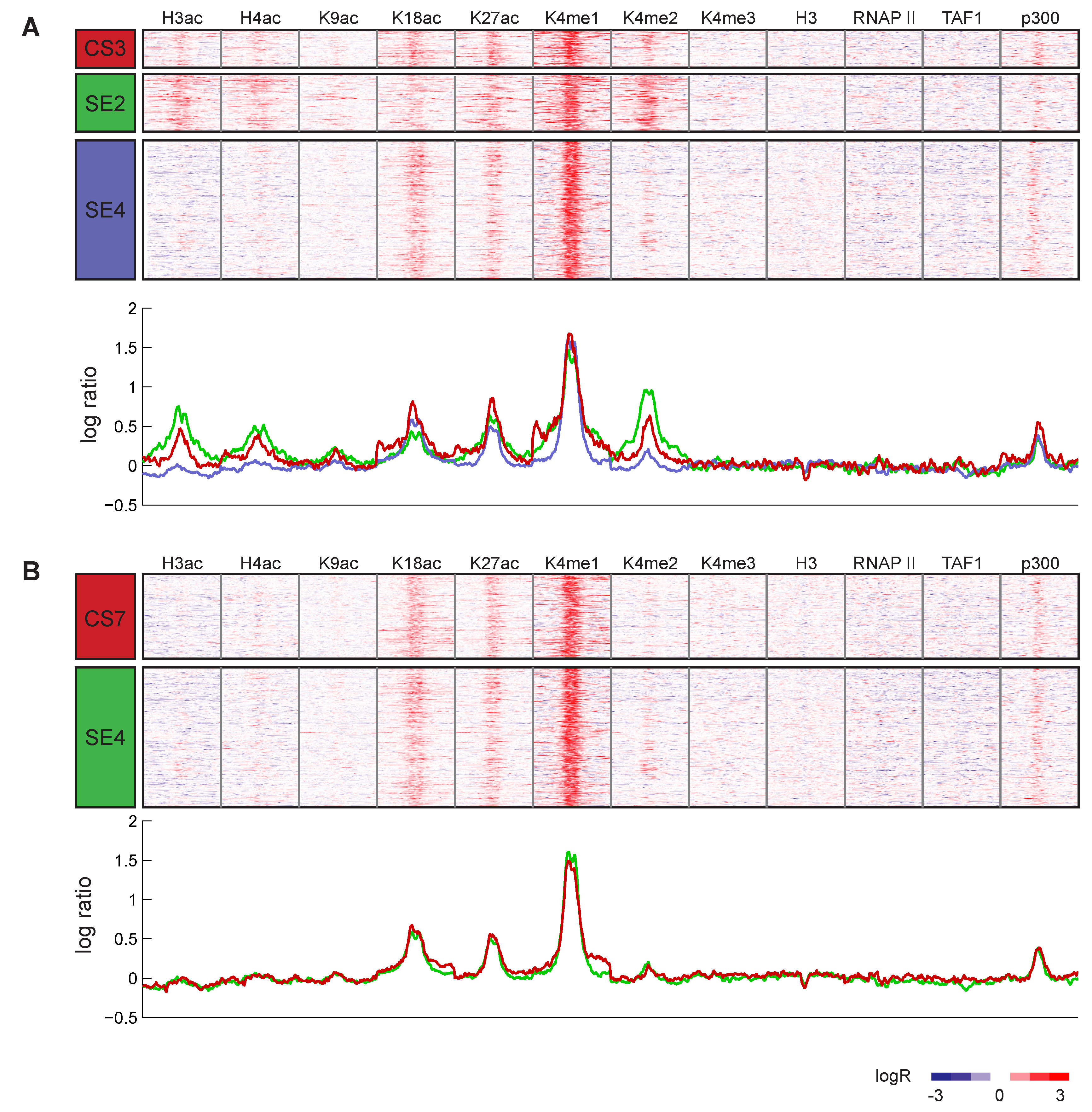

Supplement: Figure S2 — Comparison of ChromaSig clusters to clusters from Heintzman et al. [15]. Heatmaps (top) and average histone modification profiles (bottom) for ChromaSig clusters (A) CS3 and (B) CS7, together with those clusters in Heintzman et al. which recover the ChromaSig clusters. Comparisons for CS1–2 and CS8 can be found in Figure 3. Clusters CS4–6 are not recovered by Heintzman et al. clusters. The color of each curve is indicated by the color of the cluster label. (6.36 MB TIF) [file pcbi.1000201.s002.tif]
