# Supplementary material for: ChromaSig: A Probabilistic Approach to Finding Common Chromatin Signatures in the Human Genome
Source: PLoS Comput Biol. 2008 Oct 17;4(10):e1000201. doi: 10.1371/journal.pcbi.1000201 (PMC2556089; doi:10.1371/journal.pcbi.1000201)
Supplement: Table S1 — ENCODE clusters in HeLa cells. Locations and orientations of each predicted element (hg17), after applying ChromaSig to 9 histone marks mapped by ChIP–chip in HeLa cells on ENCODE arrays. (0.03 MB DOC) [file pcbi.1000201.s003.doc]

Table S1: Clusters recovered by ChromaSig

cluster CS1 (in hg17)
chrX	153864695
chr6	41862845
chr7	89871190
chr7	115734822
chrX	153278595
chr15	41907539
chr16	371751
chr1	148067784
chr7	26928111
chr22	30666758
chr21	39607817
chr6	132312590
chr5	131853964
chr5	131659164
chr7	89677490
chr9	128868806
chr7	26996811
chrX	153678595
chr21	39477517
chr7	126626457
chr15	41769639
chr15	41669739
chrX	153177895
chr7	27012711
chr20	33593079
chr20	33667979
chr11	64327239
chr5	56148757
chr20	33328279
chrX	152721195
chr5	132230264
chrX	152714595
chr13	29894366
chr21	39739617
chrX	153282695
chrX	153819195
chr6	108502347
chr19	59383935
chr7	116186422
chr1	148397884
chr16	414451
chr6	108722847
chr1	148185084
chr15	41880839
chr2	220188901
chr13	112393115
chr6	41621545
chr5	131735164
chr19	59665435
chr5	131591464
chrX	153123495
chr21	33560987
chr2	220288201
chr11	64334539
chr21	33021887
chr20	33335979
chr11	1741642
chr22	30474558
chr22	30211758
chr21	33938587
chr5	131861464
chr6	108384847
chr21	39641317
chr7	26909511
chrX	153271195
chr7	113648934
chr22	30382658
chr21	33524387
chr22	30470458
chr1	148113084
chr21	39674217
chr15	41871939
chr16	341951
chr1	148132784
chr20	33751079
chr21	39682217
chr9	129016406
chr11	5661746
chr11	116163866
chr2	220262301
chrX	153308195
chrX	152756895
chr6	108689147
chr21	34209887
chr11	64401239
chr9	128789406
chrX	153158595
chr5	131920764
chr5	56241357
chrX	122719600
chr20	33506179
chr7	115444722
chr15	41589339
chr22	31195658
chr15	41572739
chr13	29779266
chr18	59788951
chr11	1925942
chr22	30216958
chrX	152806095
chr21	33698887
chrX	153091395
chr19	59175135
chr7	89620490
chr6	41855845
chr20	33715679
chr21	33882687
chr6	74029603
chr7	114157134
chr15	41826239
chr2	118487854
chr21	32687187
chr16	391851
chrX	152670995
chr7	126822757
chr16	68151
chr5	131774564
chr9	128878506
chr7	116097222
chr21	32906687
chr13	29867766
chrX	153129595
chrX	153139295
chr16	219051
chr2	234543875
chr1	148243184
chr5	56283557
chr19	59653735
chrX	152883695
chr15	41857339
chr5	132194064
chrX	153337695
chr7	26963011
chr9	128953006
chr21	33067587

cluster CS2 (in hg17)
chr14	98919674
chr8	119160971
chr1	148333884
chr2	234390175
chr6	41799445
chr5	131629264
chr11	116449666
chr5	142362101
chr20	33356879
chr21	32815687
chr13	29813766
chr7	116044122
chr6	132317690
chr21	39274617
chr21	39287217
chr11	1805142
chr1	148157684
chr11	64379639
chrX	153392395
chr7	115950822
chr6	41788545
chr5	131618164
chr7	113766234
chr8	119093071
chr7	115551122
chr7	115865522
chr7	116037522
chr21	34242287
chr13	112414415
chr8	119106371
chr21	39431617
chr22	31253158
chr21	32758487
chr7	115464022
chr7	115454222
chr11	64367539
chr6	41774645
chr13	29615066
chr7	115637422
chr7	115746222
chr18	59501151
chr7	115505822
chr7	115940922
chr7	114164734
chr5	141960601
chr13	29810366
chr6	41809445
chrX	152688395
chr11	1750942

cluster CS3 (in hg17)
chr13	29785466
chr5	142234901
chr2	220379701
chr8	119059771
chr11	1761342
chr7	115711122
chr21	39309217
chr7	116358722
chr5	55947657
chr7	116392122
chr8	119086671
chr6	132340190
chr7	116894022
chr7	116619622
chr20	33378179
chr7	116032122
chr7	89897690
chr13	112786515
chr7	115493822
chr7	116647622
chr7	113789434
chr7	116822422
chr7	115825122
chr7	114168934
chr13	29804666
chr13	29775266
chr11	116360466
chr22	30367658
chr6	41504245
chr21	33949887
chr7	113736734
chr2	220212401
chr1	148382584
chr6	132344490
chr7	116366122
chr7	115657722
chr19	59186535
chr7	115629522
chr22	31482258
chr13	29903366
chr21	34093287
chr13	29881166
chr7	116504722
chr21	39300417
chr5	55956957
chr7	114086034
chr7	116012322
chr8	119181071

cluster CS7 (in hg17)
chr7	116917022
chr7	116937922
chr6	132448190
chr16	103551
chr7	114465634
chr7	90091090
chr2	220151401
chr7	116880022
chr12	38827627
chr6	132226790
chr21	32745287
chr6	132561990
chr2	234365775
chr5	56048857
chr1	148360584
chr18	23853182
chr6	73797503
chr7	115689722
chr7	115726322
chr6	132553490
chr15	41596039
chr11	115978066
chr21	33977687
chr18	23909982
chr18	23958482
chr7	125923057
chr2	234362375
chr7	90098990
chr7	117010522
chr12	38655627
chr12	38890027
chr7	115935822
chr2	234507575
chr2	234694675
chr14	53252226
chr14	98493174
chr7	115850922
chr7	114098034
chr12	38669627
chr18	24017382
chr21	39341817
chr8	119130571
chr8	118944471
chr18	59593151
chr21	39408817
chr4	118678909
chr11	5583846
chr5	142282501
chr21	39419417
chr7	115699622
chr18	23874382
chr5	142200001
chr7	125935757
chr22	30770958
chr14	53327326
chr5	142369001
chr6	132427190
chr2	234454975
chr5	55876057
chr7	125808957
chr18	23847482
chr5	131458464
chr2	51951706
chr6	132696990
chr7	90439190
chr7	89453390
chr7	115597722
chr7	114321434
chr18	23981882
chr18	24199382
chr18	23949482
chr7	126171757
chr11	5715146
chr6	132633590
chr18	59685551
chr8	118913271
chr22	31416658
chr2	234370775
chr22	31260658
chr14	52994926
chr7	115667822
chr6	73846403
chr7	115822222
chr7	115564822
chr7	113817934
chrX	153522695
chr11	130805848
chr8	119201971
chr7	126116757
chr7	90516690
chr6	132599490
chr14	53079326
chr18	59545051
chr14	53388826
chr2	234375475
chr11	116387166
chr7	89882090
chr7	126052557
chr7	115810922
chr18	59459551
chr8	119038371
chr8	119045171
chr18	59477151
chr7	114025534
chr18	23921482
chr7	115682022
chr8	119291271
chr21	34262987
chrX	153591695

cluster CS8 (in hg17)
chr14	53219026
chr8	118967971
chr7	116554322
chr6	41520045
chr7	90457690
chr4	119072509
chr18	23996082
chr7	126121657
chr8	119076471
chr4	119056809
chr5	131636964
chr7	116841222
chr2	234738875
chr5	56007657
chr14	53279926
chr5	55981657
chr6	73829203
chr18	59482851
chr14	53144926
chr7	117180322
chr7	126111057
chr7	115931222
chr6	41565145
chr22	31364558
chr5	142239301
chr6	41843945
chr5	142285801
chr6	132622590
chr7	113808734
chr6	41593545
chr11	1728542
chr14	98481974
chr6	132594690
chr11	5346946
chr11	116398266
chr14	98635874
chr7	114178534
chr7	126058257
chr13	112428315
chr14	53344226
chr18	23905682
chr21	33668587
chr7	117062522
chr14	53430326
chr2	220419201
chr5	132040864
chr5	142170201
chr7	113894034
chr2	51922106
chr6	132300790
chr8	119283771
chr7	115816422
chr2	234667975
chr8	119153171
chr8	119135371
chr11	116439866
chr12	38666627
chr7	125729257
chr5	56065157
chr6	41542145
chr7	116766322
chr7	126672457
chr22	31371458
chr15	42012639
chr11	5321346
chr7	113937734
chr7	114370134
chr18	23962182
chr8	119035171
chr8	119149971
chr7	115835222
chr7	126038157
chr5	55939457
chr12	38918027
chr14	53416126
chr8	119065971
chr13	29597766
chr2	234672575
chr7	125961857
chr18	59700851
chr7	113700934
chr6	132495090
chr18	23913582
chr7	115574522
chr7	116834822
chr21	33946387
chr7	116493922
chr5	131437364
chr7	116206122
chr5	131449864
chr21	39470217
chr7	116948522
chr7	90523190
chr5	142139401
chr11	5325646
chr14	53330726
chr14	53053426
chr2	118431954

cluster CS6 (in hg17)
chr2	220269501
chr5	56170657
chr21	33873387
chr15	41831439
chr7	27159711
chr7	126581857
chr11	116453766
chr5	132110864
chr9	129100306
chr7	27048211
chr11	64284039
chr1	148405284
chr16	360551
chr13	112596515
chr6	41712945
chr21	33713487
chr1	148230284
chr7	26885311
chr11	116211566
chr7	114213734
chr13	112696915
chrX	153597595
chr6	108432747
chr12	38734527
chr7	116854322
chr7	115522722
chr11	116411266
chr11	64389339
chr9	128865406
chr11	2116242
chr11	63973039
chr13	112588915
chr8	119175771
chrX	152868895
chr14	98810574
chrX	153324095
chr11	2247042
chr6	108561947
chr13	112645915
chr11	64412139
chr7	117107422
chrX	152797295
chr21	34218487
chr18	59767251

cluster CS4 (in hg17)
chr16	25938478
chr8	119359371
chr11	130674048
chr14	53398526
chr11	5466546
chr22	31770258
chr16	150751
chr15	41808939
chr7	116999122
chr13	112610615
chr22	30626858
chr6	73922403
chr19	59955735
chr21	39707717
chr5	56104057
chr1	148084484
chr11	116114966
chr21	33445587
chr14	98557574
chr11	116088266
chr19	60007435
chr16	26214978
chr1	148213384
chr11	116233966
chr16	25852378
chr22	31594658
chr11	64128939
chr22	31077158
chr7	116018822
chr11	4804446
chr20	33610379
chr16	26050778
chr7	90487290
chr9	128842506
chr11	5408446
chr7	26937111
chr11	64153239
chr21	39687717
chr5	142272301
chr7	27171711
chr5	131355964
chr2	220360001
chr13	29645666
chr13	112520915
chr11	4785746
chr19	59488935
chrX	153567095
chr7	90316790
chr20	33693879
chrX	153576695
chr2	118138854
chr7	126652557
chr22	30864158
chr22	31575858
chr13	29651166
chr8	119303671
chr8	119335671
chr11	5400446
chr13	29432266
chr15	41698539
chr5	55909457
chr5	142035001
chr14	98939474
chr15	41614039
chr15	41968439
chr2	118363154
chr7	125746057
chr2	118085854
chr7	27136411
chr5	131387464
chr5	131415964
chr11	4923946
chr15	41761539
chr19	59962135
chr16	25978378
chr5	131383764
chr7	116462522
chr5	132151764
chr15	41743639
chr11	64137639
chr5	131760464
chr7	116963722
chr15	41678839
chr19	59725035
chr11	116109966
chr16	25833978
chr19	59552635
chr5	132119364
chr7	126440457
chr22	30434158
chr7	90505090
chr21	33340087
chr11	4782246
chr16	25901678
chr14	98762174
chr15	41973239
chr22	31792558
chr22	30879358
chr22	31689358
chrX	153021295
chr7	125767957
chr7	89686590
chr20	33574279
chr5	131335764
chr5	142157601
chr21	32915587
chr7	116981522
chr20	33415379
chr11	5414746
chr16	22651
chr22	31454558
chr15	41953139
chr21	33089187
chr2	220513201
chr11	64170539
chr22	31777358
chr7	27120611
chr21	32881487
chr14	98682874
chr19	59977435
chr11	115966166
chr19	59945635
chr2	234661575
chr11	4896546
chr11	5221846
chr11	4854946
chr11	5361146
chr11	116251866
chr22	30908458
chr20	33678379
chr22	31084658
chr9	128835506
chr16	25974278
chr13	112433315
chr6	41850945
chr22	30304858
chr14	98799174
chr7	114351334
chr11	5367646
chr1	148150384
chr13	112547415
chr11	5431346
chr2	118326854
chr13	29638566
chr7	113627134
chr13	29508466
chr19	59107635
chr15	41656739
chr15	41712539
chr7	27201911
chr14	98611474
chr7	90220390
chr13	29601466
chr14	98719674
chr11	5377746
chr15	41689939
chr11	64113239
chr11	131003648
chr11	5487046
chr22	31582758
chr11	5657246
chr7	126311657
chr22	30172258
chr19	59804835
chr20	33682879
chr8	118899871
chr2	118336554
chr15	41718839
chr2	234623375
chr16	26259378
chr16	25951078
chr11	5247246
chr11	5492946
chr15	41647939
chr14	98929874
chr2	118414854
chr11	4966246
chr22	30913458
chr7	126443557
chr11	130994348
chr16	25866678
chr7	116475522
chr7	126205357
chr15	41779339
chr20	33565979
chr2	220599401
chr11	5546546
chr11	4893546
chr11	130749248
chr8	119366571
chr14	98703774
chr11	116223366
chr7	125970957
chr5	56293057
chr22	30830258
chr19	59700435
chr14	98746574
chr22	30163258
chr11	116089566
chr2	220464001
chr7	126451757
chr22	30762358
chr11	4826446
chr16	25898378
chr21	34133587
chr11	130954948
chr16	25801078
chr5	141994501
chr11	5148346
chr11	5157746
chr15	41747539
chr14	98778674
chr15	41639639
chr16	25787178
chr11	130770448
chr6	41527845
chr22	30624358
chr22	30157058
chr7	126829957
chr7	113932234
chr19	59942535
chr11	64058639
chr11	5558746
chr14	98928174
chrX	152946195
chr5	131423364
chr11	4901846
chr19	60016935
chr22	30543058
chr11	5638946
chr19	59466935
chr1	148051584
chr5	131371764
chr20	33725279
chr15	41644139
chr13	29658266
chr5	56098357
chr11	5611946
chr20	33531079
chr11	131032448
chrX	153571695
chr2	118391654
chr22	31537858
chr11	116104466
chr7	27191711
chr5	132236664
chr11	4993546
chr7	116326022
chr11	5447146
chr11	64158139
chr11	64219339
chr13	112534715
chr11	116022466
chr5	142025501
chr6	73972803
chr20	33553879
chr22	31570158
chr11	2020342
chr7	126324457
chr15	41610839
chr22	31559258
chr11	130941448
chr5	142209001
chr15	41605439
chr5	142096101
chr8	119370171
chr14	53063326
chr13	112623015
chr11	130903148
chr19	59928635
chr22	30698658
chr22	31183958
chr7	126573157
chr5	56032957
chr10	55538069
chr22	31487658
chr19	59991735
chr2	118077854
chr1	148192084
chr7	27209711
chr22	31587458
chr22	31098258
chr11	131037348
chr2	118402854
chr5	142250601
chr16	26055178
chr6	73961803
chr13	112352215
chr11	130895248
chr11	64193039
chr11	5225746
chr22	31221358
chrX	153470795
chr9	128824206
chr22	30154958
chr6	108552147
chr1	148210584
chr21	32798887
chr20	33557879
chr20	33580879
chr7	126709357
chr20	33721779
chr13	29520066
chr19	59986835
chr14	98596274
chr5	132007664
chr22	30958058
chr11	131050548
chr21	33252487
chr5	142027301
chr5	131368964
chr11	130728248
chr2	234626675
chr22	31030658
chr14	98875074
chr22	31723458
chr22	31079258
chr7	126750157
chr11	5503346
chr11	64209439
chr2	220522001
chr11	64275639
chr11	4837646
chr15	41756439
chr13	112348415
chr5	142290601
chr19	59729035
chr19	59958235
chr5	55931157
chr11	4933346
chr5	142262701
chr11	64203639
chr11	64245239
chr20	33523879
chr21	33112287
chr16	25821678
chr16	26271278
chr11	5419246
chr16	25964478
chr5	131343964
chr11	4950746
chr2	118024854
chr7	89767290
chr5	141952101
chr5	131567864
chr11	5025046
chr5	132124464
chr13	112530415
chr21	34159187
chr11	64076239
chr2	118171554
chr22	30745858
chr2	118150254
chr15	41739039
chr2	118031954
chr19	59484935
chr14	98672574
chr16	26076278
chr16	26046078
chr15	41815339
chr15	41661739

cluster CS5 (in hg17)
chr12	39092027
chr22	30325058
chr10	55252769
chr6	41826345
chr21	39492017
chr7	126270857
chr22	30802358
chr14	53215426
chr7	113589434
chr7	126374557
chrX	153451495
chr11	5293546
chr2	220561301
chr2	234689275
chr6	74136503
chr11	5705446
chr7	126306557
chr7	117139722
chr5	142327001
chr7	126739157
chrX	152815095
chr16	60898800
chr11	5211546
chr7	113968234
chr16	61141000
chr5	56078857
chr21	39719217
chr11	5185046
chr6	108614447
chr6	132611090
chrX	122955300
chr6	41835845
chr11	5202546
chrX	152977195
chr14	53159426
chr11	4858046
chrX	153167495
chr5	55992157
chr11	5329346
chr11	4810946
chr7	114337634
chr11	131044448
chr2	234482775
chr5	132256564
chr7	116486222
chr20	33321679
chr12	38874927
chr11	4841646
chr10	55473969
chr21	33301487
chr11	130917348
chr6	74057503
chr7	125915757
chr11	5529646
chr5	132135164
chr2	220528401
chr14	52989826
chr5	142309401
chr2	220216301
chrX	153721395
chr14	53107726
chr21	34185887
chr11	130853648
chr7	126427557
chr16	26149778
chr7	117211322
chr7	113981034
chr11	131011848
chr16	26168578
chr11	5302146
chr11	5538846
chr2	234493975
chr2	118160054
chr2	118471454
chr2	118425954
chr7	90199390
chr21	33141187
chr7	114382934
chr6	41637445
chr11	130722948
chr7	117248122
chr21	33120187
chr14	53072326
chr6	41649445
chr5	131903464
chr5	56014257
chr11	64104439
chr7	126390057
chr18	23827282
chrX	153510995
chr7	90188390
chr7	126348057
chr21	33247287
chr2	234652175
chr7	117240322
chr6	108589947
chr22	30404158
chrX	153489795
chr16	61012600
chr22	31231558
chr16	26096778
chr6	73888303
chr7	90429590
chr15	41529639
chr6	108455947
chr11	5070546
chr13	112822215
chr18	23990182
chr11	4914946
chr22	31136858
chrX	153079295
chr11	116229566
chr7	115783322
chrX	153584195
chr16	26041078
chr11	5015546
chr10	55238369
chr7	116549522
chr14	53012826
chr11	5313646
chr16	92051
chr2	118104954
chr11	116138866
chr7	126385357
chr7	126341657
chr11	130936748
chr11	130650948
chr11	116034666
chr5	131430164
chrX	153939995
chrX	153631095
chr13	29873966
chr7	117096322
chr11	116261966
chr2	234750475
chr7	90465690
chr7	114318234
chr7	117237422
chr2	220313101
chr2	220356301
chr5	55917957
chrX	152742495
chr7	26802511
chr18	59540651
chr21	33268487
chr6	132388990
chrX	153503395
chr7	117101922
chr11	130840248
chr7	27188011
chr7	116334222
chr21	33327087
chr7	114189334
chr11	5279246
chr11	64260939
chr2	118181054
chr22	30968858
chr11	5643346
chr22	31412058
chr11	1893042
chr16	60890500
chr21	33405687
chr16	61208400
chr13	112721615
chr11	116246166
chr7	126134157
chr5	131513964
chr2	52065406
chr7	117122522
chr2	234604975
chr14	53223126
chr7	116424422
chr13	112458715
chr11	5041946
chr7	114346234
chr14	98536374
chr9	128910406
chr6	41500045
chr11	5047246
chr11	5554346
chr7	89466490
chr15	41559739
chr16	492051
chrX	152748495
chr11	5686846
chr7	90300590
chr5	141922101
chr6	73852103
chr7	116350922
chr7	90434790
chr11	5037446
chr16	60863800
chr7	27221911
chr11	5110446
chr21	33142287
chr21	33128987
chr2	118192254
chr11	5174546
chr5	142299101
chr1	148034184
chr7	117160722
chr2	220194801
chr2	220486301
chr11	64013739
chrX	152660595
chr5	131311564
chr9	128994306
chr19	60000535
chr2	234681675
chr16	26236278
chr11	5674946
chr2	234766575
chrX	153688695
chr2	220554701
chr2	118220054
chr11	5178546
chr7	114236734
chr6	41570645
chr11	130868348
chr22	31552158
chr14	98943474
chr7	126086457
chr6	73985003
chr11	116169066
chr11	5299346
chr7	27112911
chr11	5622246
chr19	59339835
chr8	119211071
chr11	4800046
chr11	130667548
chr11	4874146
chr11	130987048
chrX	153908495
chr19	59156835
chr8	119345971
chr14	98693074
chr14	53084426
chr7	116539822
chr8	118972971
chr22	31427858
chr2	234744575
chr12	38953327
chr11	5462346
chr22	31463758
chr20	33563179
chr6	108705947
chr5	131597064
chr12	38721327
chrX	153877995
chr9	128853906
chr11	130616648
chr5	55892757
chr13	112467515
chr22	31652358
chr11	4977646
chr8	119122671
chrX	152939995
chr14	53138426
chr11	4769346
chr11	131037348
chr1	148304984
chr7	116077222
chr7	116322522
chr11	4764546
chr6	41667345
chr7	126136957
chr15	41997839
chr8	119272371
chr16	61018000
chr2	220326301
chr22	31121858
chr22	31116358
chr11	130933248
