# Supplementary material for: ChromaSig: A Probabilistic Approach to Finding Common Chromatin Signatures in the Human Genome
Source: PLoS Comput Biol. 2008 Oct 17;4(10):e1000201. doi: 10.1371/journal.pcbi.1000201 (PMC2556089; doi:10.1371/journal.pcbi.1000201)
Supplement: Table S2 — Genomewide clusters in CD4+ T cells. Locations and orientations of each predicted element (hg18), after applying ChromaSig to 21 histone marks mapped by ChIP-Seq in CD4+ T cells genomewide. (1.52 MB RTF) [file pcbi.1000201.s004.rtf]

Cluster GW1 (in hg18)
chr1	938700	-
chr1	1138800	-
chr1	1499500	+
chr1	1540300	-
chr1	1554400	+
chr1	1701700	-
chr1	1813300	-
chr1	2054600	+
chr1	2111200	-
chr1	2338100	-
chr1	2468700	-
chr1	2506300	+
chr1	2563900	+
chr1	3437300	+
chr1	3531200	+
chr1	3702500	+
chr1	3764300	-
chr1	3806400	+
chr1	5976300	-
chr1	6009800	-
chr1	6181900	+
chr1	6218300	+
chr1	6376000	+
chr1	6447900	+
chr1	6562900	-
chr1	6595900	+
chr1	6684500	+
chr1	7754300	-
chr1	7944600	-
chr1	8861000	+
chr1	9111600	+
chr1	9217500	-
chr1	9276200	-
chr1	9411300	+
chr1	9612700	-
chr1	9806700	+
chr1	9892500	+
chr1	10015900	-
chr1	10193500	-
chr1	10381800	-
chr1	10457800	-
chr1	10478200	+
chr1	10493500	+
chr1	10995600	-
chr1	11042400	+
chr1	11082100	+
chr1	11256100	-
chr1	11637800	-
chr1	11663600	+
chr1	11963400	-
chr1	12002300	-
chr1	12213200	-
chr1	12602000	-
chr1	13929900	+
chr1	15726000	-
chr1	15817100	-
chr1	15884000	-
chr1	16048700	-
chr1	16174600	+
chr1	16405700	+
chr1	16416100	+
chr1	16551100	+
chr1	16567100	-
chr1	16843300	+
chr1	17113000	+
chr1	17210400	+
chr1	17252700	+
chr1	17638100	+
chr1	19101300	+
chr1	19408900	+
chr1	19450900	-
chr1	19511000	+
chr1	19796500	-
chr1	20081800	-
chr1	20706900	+
chr1	20832900	-
chr1	20860300	+
chr1	20985800	+
chr1	21375300	+
chr1	21493000	-
chr1	21544100	+
chr1	21850700	+
chr1	21981600	+
chr1	22224200	+
chr1	22651700	-
chr1	23218900	-
chr1	23367300	+
chr1	23542900	+
chr1	23567500	+
chr1	23891300	-
chr1	23943000	-
chr1	23977700	-
chr1	23990500	-
chr1	23999200	+
chr1	24615600	-
chr1	24842900	-
chr1	25431200	+
chr1	25445900	+
chr1	25630200	-
chr1	26019700	-
chr1	26104900	+
chr1	26310900	-
chr1	26369300	-
chr1	26434000	-
chr1	26479200	-
chr1	26671200	+
chr1	26699800	+
chr1	26729200	-
chr1	27026300	-
chr1	27064000	+
chr1	27089200	+
chr1	27099000	+
chr1	27121000	-
chr1	27353000	+
chr1	27434000	-
chr1	27521700	-
chr1	27541600	-
chr1	27565100	+
chr1	27688700	+
chr1	27721900	-
chr1	27972500	-
chr1	28030300	-
chr1	28072400	-
chr1	28159400	-
chr1	28287400	+
chr1	28435800	-
chr1	28459000	-
chr1	28705700	-
chr1	28717800	-
chr1	28780500	+
chr1	28936500	-
chr1	29085800	+
chr1	29114100	-
chr1	29380400	+
chr1	30964600	-
chr1	31310600	+
chr1	31542300	+
chr1	31882800	+
chr1	32175800	+
chr1	32252800	-
chr1	32311500	-
chr1	32346700	-
chr1	32418500	-
chr1	32439000	+
chr1	32460500	-
chr1	32530600	-
chr1	32632500	+
chr1	32889500	-
chr1	32992500	-
chr1	33055600	+
chr1	33202200	+
chr1	33274600	+
chr1	33319700	-
chr1	33419700	+
chr1	33587500	+
chr1	35097600	+
chr1	35222900	+
chr1	35430400	+
chr1	35795200	+
chr1	35815600	+
chr1	35879400	+
chr1	35956700	+
chr1	36169700	-
chr1	36327400	-
chr1	36387500	+
chr1	36462500	-
chr1	36544800	+
chr1	36623700	+
chr1	36635700	+
chr1	36702100	+
chr1	37752600	+
chr1	37833700	+
chr1	38046200	+
chr1	38097500	+
chr1	38169600	+
chr1	38227700	+
chr1	38251200	-
chr1	39097700	+
chr1	39111300	+
chr1	39230100	-
chr1	39264900	-
chr1	39454300	-
chr1	39813900	+
chr1	39929500	+
chr1	39977100	-
chr1	40121400	+
chr1	40193500	-
chr1	40279400	-
chr1	40400300	-
chr1	40496600	-
chr1	40554400	+
chr1	40612800	-
chr1	40903300	+
chr1	40930300	-
chr1	40947400	-
chr1	41040600	+
chr1	41056800	+
chr1	41100300	+
chr1	41218400	-
chr1	41900000	+
chr1	42573100	+
chr1	42694900	-
chr1	42896800	-
chr1	42920400	-
chr1	43005200	+
chr1	43085000	-
chr1	43196700	+
chr1	43410500	+
chr1	43597300	-
chr1	43606000	+
chr1	43627900	+
chr1	43691900	+
chr1	43888700	-
chr1	43945400	+
chr1	44185300	-
chr1	44213800	-
chr1	44268800	-
chr1	44870500	-
chr1	45224500	+
chr1	45250000	+
chr1	45565000	-
chr1	45578700	-
chr1	45759600	+
chr1	45789400	-
chr1	45822800	-
chr1	46436400	+
chr1	46541200	+
chr1	46842300	+
chr1	46854300	+
chr1	46906100	+
chr1	46956900	+
chr1	47572200	+
chr1	47675400	+
chr1	51198100	+
chr1	51474800	-
chr1	51756800	+
chr1	51855400	-
chr1	52116500	+
chr1	52271300	+
chr1	52294100	-
chr1	52603800	+
chr1	52642900	-
chr1	52790900	+
chr1	52841200	-
chr1	52964900	-
chr1	53159400	+
chr1	53253500	-
chr1	53476500	+
chr1	53565600	+
chr1	54076100	+
chr1	54183800	+
chr1	54291200	+
chr1	54438500	-
chr1	54954400	-
chr1	55038900	+
chr1	55125100	+
chr1	58938100	+
chr1	59054200	+
chr1	61320700	-
chr1	61963100	+
chr1	63831700	+
chr1	65240900	+
chr1	65659000	-
chr1	67163700	-
chr1	67924300	-
chr1	70444400	-
chr1	70460400	-
chr1	70592300	+
chr1	74971500	+
chr1	76024900	-
chr1	77921000	+
chr1	77997700	+
chr1	78018100	-
chr1	78217100	+
chr1	84718400	-
chr1	84743900	+
chr1	84928500	+
chr1	85497400	+
chr1	85514500	+
chr1	85945800	+
chr1	86634100	+
chr1	86943400	-
chr1	87569500	-
chr1	89129300	+
chr1	89260300	+
chr1	89763300	-
chr1	90059000	+
chr1	91259900	+
chr1	92042500	-
chr1	92123800	-
chr1	93070700	-
chr1	93199300	+
chr1	93418300	+
chr1	94117000	+
chr1	94656900	-
chr1	95472500	-
chr1	100208600	-
chr1	100276600	-
chr1	100371200	-
chr1	100504600	-
chr1	100661300	+
chr1	101134300	-
chr1	103841300	-
chr1	107401300	-
chr1	108279700	-
chr1	108543900	+
chr1	108904500	-
chr1	109036900	-
chr1	109091300	-
chr1	109307000	+
chr1	109435200	-
chr1	109444900	-
chr1	109558500	-
chr1	109828300	-
chr1	109839000	-
chr1	109846500	+
chr1	109893100	-
chr1	110255300	-
chr1	110329100	-
chr1	110379100	-
chr1	110555000	-
chr1	110751300	+
chr1	110963700	+
chr1	111484400	-
chr1	111793800	-
chr1	111860200	-
chr1	111964400	-
chr1	112100100	-
chr1	113019400	-
chr1	113300500	-
chr1	113417700	-
chr1	114248900	+
chr1	114855000	+
chr1	114925400	+
chr1	115060600	+
chr1	115101700	+
chr1	115433400	+
chr1	116914400	+
chr1	117950600	-
chr1	118274000	-
chr1	143808700	-
chr1	144227700	-
chr1	144287500	-
chr1	144321400	+
chr1	145538400	-
chr1	145608500	+
chr1	148123300	+
chr1	148166400	+
chr1	148389400	-
chr1	148507600	+
chr1	148560700	-
chr1	148603900	-
chr1	148800200	+
chr1	148868100	+
chr1	149213100	+
chr1	149287200	-
chr1	149396300	-
chr1	149404800	+
chr1	149429100	+
chr1	149438200	-
chr1	149521800	-
chr1	149566300	+
chr1	149585800	+
chr1	149639100	-
chr1	149697500	+
chr1	149851800	-
chr1	150078700	+
chr1	150232200	+
chr1	150274500	+
chr1	151774700	+
chr1	151783900	+
chr1	151873600	-
chr1	151898000	-
chr1	151910000	+
chr1	152015000	-
chr1	152161600	+
chr1	152184800	+
chr1	152196600	+
chr1	152206200	+
chr1	152216300	+
chr1	152230300	-
chr1	152459500	+
chr1	152511800	-
chr1	152565200	-
chr1	152592000	+
chr1	152797300	+
chr1	152847000	+
chr1	153200400	+
chr1	153213900	+
chr1	153222700	-
chr1	153290500	-
chr1	153302700	+
chr1	153324800	-
chr1	153375300	-
chr1	153412500	-
chr1	153430100	+
chr1	153445600	-
chr1	153491500	-
chr1	153498300	+
chr1	153513700	+
chr1	153545500	-
chr1	153560600	-
chr1	153799600	-
chr1	154147400	+
chr1	154170300	+
chr1	154257300	+
chr1	154290700	-
chr1	154430700	-
chr1	154482600	+
chr1	154518800	+
chr1	154531700	+
chr1	154574400	+
chr1	154828400	-
chr1	154942200	+
chr1	154964600	+
chr1	154986200	+
chr1	155004000	-
chr1	155052700	+
chr1	155374100	+
chr1	158017600	-
chr1	158268000	+
chr1	158334800	+
chr1	158442300	-
chr1	158521200	+
chr1	158579300	+
chr1	159033300	-
chr1	159257000	+
chr1	159274800	-
chr1	159281700	+
chr1	159305500	+
chr1	159334500	-
chr1	159354700	-
chr1	159368800	+
chr1	159396200	-
chr1	159413400	+
chr1	159439000	-
chr1	159463200	-
chr1	159542300	+
chr1	159551200	-
chr1	159962900	+
chr1	159986600	-
chr1	160002900	-
chr1	160734600	-
chr1	160798200	-
chr1	164004500	+
chr1	165075800	-
chr1	165111700	+
chr1	165789000	+
chr1	165867100	-
chr1	166415300	-
chr1	168129400	+
chr1	168768200	-
chr1	169721700	-
chr1	169977400	+
chr1	170679300	+
chr1	170769000	-
chr1	171713000	+
chr1	171951100	-
chr1	172103700	+
chr1	172395600	-
chr1	173235900	-
chr1	173258800	+
chr1	173427700	+
chr1	174442500	+
chr1	177262100	-
chr1	177318100	-
chr1	177465000	+
chr1	177530200	-
chr1	178118800	-
chr1	178190800	-
chr1	178390900	-
chr1	178738000	+
chr1	179258100	+
chr1	179269900	-
chr1	179325400	-
chr1	180626400	+
chr1	180840200	+
chr1	181075500	-
chr1	182287600	-
chr1	182990400	+
chr1	183209500	+
chr1	183281600	-
chr1	184610900	+
chr1	191294800	+
chr1	191340600	+
chr1	191358100	-
chr1	195436900	+
chr1	196010700	+
chr1	198644900	+
chr1	199350900	-
chr1	199742400	+
chr1	200065900	-
chr1	200191100	-
chr1	200218700	-
chr1	200379700	+
chr1	200395700	+
chr1	200584800	-
chr1	201124200	+
chr1	201193800	+
chr1	201203000	+
chr1	201243400	-
chr1	202097400	-
chr1	202730100	+
chr1	202753000	-
chr1	203357500	+
chr1	203446900	+
chr1	203867100	+
chr1	203985200	+
chr1	204048500	+
chr1	204711000	-
chr1	204747200	+
chr1	204852100	+
chr1	205290500	+
chr1	205561800	-
chr1	205992300	-
chr1	206108100	+
chr1	208024200	+
chr1	208068700	-
chr1	209818300	+
chr1	210654400	+
chr1	210673100	-
chr1	210939800	+
chr1	211031600	+
chr1	211255100	+
chr1	212521300	-
chr1	216525600	-
chr1	218285400	+
chr1	218334300	-
chr1	218511800	+
chr1	219981200	+
chr1	220858200	-
chr1	221971500	-
chr1	222099700	+
chr1	222368900	-
chr1	222438000	-
chr1	222584100	+
chr1	222611800	-
chr1	222690400	-
chr1	223681800	+
chr1	224032400	-
chr1	224177400	+
chr1	224253500	+
chr1	224317000	+
chr1	224440400	+
chr1	225194600	-
chr1	225989300	+
chr1	226042700	-
chr1	226363500	+
chr1	226420500	-
chr1	226468500	-
chr1	226530100	+
chr1	226712200	+
chr1	226741900	-
chr1	227474400	-
chr1	227710400	+
chr1	227760500	+
chr1	227827900	+
chr1	228270200	-
chr1	229180800	+
chr1	229242100	+
chr1	229731300	-
chr1	231530500	-
chr1	232576300	-
chr1	233358100	+
chr1	233391300	-
chr1	234373000	-
chr1	234511500	+
chr1	234834100	+
chr1	242681300	+
chr1	242883300	-
chr1	244796000	+
chr1	245161500	+
chr1	245440000	+
chr1	245561200	+
chr1	247086700	+
chr1	247099600	-
chr1	247119500	+
chr1	247167400	-
chr10	173200	+
chr10	966800	+
chr10	1024600	-
chr10	1084800	+
chr10	1093100	-
chr10	3100700	-
chr10	3204600	+
chr10	5767500	-
chr10	5775000	+
chr10	5895300	+
chr10	5971800	-
chr10	6171700	-
chr10	6245800	-
chr10	6283600	-
chr10	7870600	-
chr10	7900900	-
chr10	12124400	+
chr10	12151300	-
chr10	12212100	-
chr10	12278200	-
chr10	12432200	-
chr10	13182600	-
chr10	13669200	-
chr10	14653900	-
chr10	14669200	+
chr10	14920400	-
chr10	14960700	+
chr10	15035600	+
chr10	15179700	-
chr10	15250500	+
chr10	16899400	+
chr10	17536100	+
chr10	17726200	-
chr10	18988600	-
chr10	22331900	+
chr10	22645200	-
chr10	23768900	-
chr10	27188800	+
chr10	27483600	+
chr10	27833600	-
chr10	28862100	-
chr10	30064300	+
chr10	30677700	+
chr10	32257700	+
chr10	32384700	+
chr10	32675000	+
chr10	35418800	+
chr10	35665000	+
chr10	38423500	-
chr10	42367900	+
chr10	42598600	-
chr10	42954300	-
chr10	43211900	+
chr10	44774500	+
chr10	44816800	-
chr10	45409500	+
chr10	45487900	+
chr10	47110000	+
chr10	49185200	-
chr10	50416700	+
chr10	51236100	-
chr10	53129100	+
chr10	59765200	-
chr10	59815200	-
chr10	61335500	+
chr10	63333300	-
chr10	64234900	-
chr10	64246800	-
chr10	64895200	+
chr10	64951400	-
chr10	69314900	-
chr10	69504500	+
chr10	69836500	+
chr10	70331100	-
chr10	70386400	-
chr10	70418800	-
chr10	70554200	-
chr10	70610200	-
chr10	70749100	-
chr10	71575200	+
chr10	71599800	+
chr10	71811800	+
chr10	71834600	-
chr10	72032100	+
chr10	72749100	-
chr10	73279800	+
chr10	73646300	-
chr10	73784000	+
chr10	74598100	-
chr10	74682100	+
chr10	74843400	+
chr10	74925800	-
chr10	75174300	-
chr10	75203100	-
chr10	75215700	-
chr10	75295400	+
chr10	75303500	+
chr10	75428400	-
chr10	75580400	+
chr10	75606800	-
chr10	76254800	+
chr10	76541700	-
chr10	76640800	-
chr10	76664700	+
chr10	79458900	+
chr10	80496900	+
chr10	80674000	-
chr10	80777700	-
chr10	80875000	+
chr10	81937100	+
chr10	85889500	-
chr10	86079000	-
chr10	88270900	+
chr10	88842900	+
chr10	89255100	-
chr10	89567800	+
chr10	89611900	+
chr10	90630400	-
chr10	90740500	+
chr10	90751500	-
chr10	91164800	-
chr10	92621900	-
chr10	92912900	-
chr10	92970700	-
chr10	93548600	-
chr10	93658200	+
chr10	93673800	-
chr10	93990100	+
chr10	94041100	-
chr10	94323500	+
chr10	97405900	+
chr10	98336400	+
chr10	98582500	-
chr10	99069700	-
chr10	99150600	+
chr10	99176300	-
chr10	99195500	+
chr10	99247800	+
chr10	99391000	-
chr10	99436800	+
chr10	99487300	-
chr10	99600300	-
chr10	99885100	-
chr10	100164700	+
chr10	100196000	+
chr10	101180200	+
chr10	101369900	+
chr10	101482300	-
chr10	101935400	+
chr10	101979000	+
chr10	102017000	+
chr10	102035900	+
chr10	102097100	-
chr10	102279000	+
chr10	102285900	-
chr10	102662900	-
chr10	102719700	+
chr10	102747900	-
chr10	102782000	-
chr10	102792700	+
chr10	102811200	-
chr10	103104100	-
chr10	103337500	+
chr10	103444000	+
chr10	103532900	+
chr10	103580600	-
chr10	103805500	+
chr10	103815400	-
chr10	103883200	-
chr10	103902500	-
chr10	103995500	-
chr10	104145400	-
chr10	104171700	-
chr10	104185500	-
chr10	104200500	-
chr10	104254700	-
chr10	104464200	-
chr10	104494000	-
chr10	104942400	+
chr10	105145900	+
chr10	105201200	+
chr10	105410400	-
chr10	105427800	+
chr10	105442700	+
chr10	105717200	-
chr10	106004900	-
chr10	106018400	+
chr10	106103100	-
chr10	111672700	+
chr10	111827000	+
chr10	111961700	-
chr10	112054200	+
chr10	112317900	-
chr10	112621700	+
chr10	114196500	+
chr10	115429800	-
chr10	115923500	+
chr10	116054500	+
chr10	116571900	-
chr10	116688300	-
chr10	119795900	+
chr10	120091300	+
chr10	120504100	+
chr10	120779900	-
chr10	120829700	+
chr10	120927900	+
chr10	121055800	-
chr10	121345900	+
chr10	121475800	-
chr10	121621600	+
chr10	121642400	-
chr10	123677200	+
chr10	123724100	+
chr10	124628800	+
chr10	124730300	-
chr10	124757900	+
chr10	124904500	-
chr10	126470200	+
chr10	126480800	-
chr10	126595900	-
chr10	127398300	-
chr10	127502500	-
chr10	129736400	-
chr10	129899700	-
chr10	131799000	+
chr10	131825000	-
chr10	133644900	+
chr10	133994400	+
chr10	134939200	+
chr10	135042300	+
chr11	226900	-
chr11	270900	-
chr11	278700	-
chr11	527100	-
chr11	684800	+
chr11	737900	-
chr11	899800	+
chr11	1240000	+
chr11	1741500	+
chr11	1925700	-
chr11	2279600	-
chr11	2378700	-
chr11	2424400	-
chr11	3034600	+
chr11	3356800	+
chr11	3774900	+
chr11	3786400	-
chr11	3835000	-
chr11	4072900	-
chr11	4371000	+
chr11	5662300	+
chr11	5668600	-
chr11	6212400	+
chr11	6368700	-
chr11	6451700	+
chr11	6459500	-
chr11	6581200	+
chr11	6596600	+
chr11	6633100	+
chr11	6660800	+
chr11	7965700	-
chr11	8661100	-
chr11	8942100	+
chr11	9292200	+
chr11	9363000	-
chr11	10433800	+
chr11	10729800	-
chr11	10786600	+
chr11	12088600	+
chr11	13440800	+
chr11	13646900	-
chr11	14336600	+
chr11	14477700	+
chr11	14498200	+
chr11	16717100	-
chr11	17055500	+
chr11	17185800	+
chr11	17991600	-
chr11	18300800	-
chr11	18373600	-
chr11	18504800	+
chr11	18612200	+
chr11	18684200	+
chr11	19095600	-
chr11	20341900	-
chr11	22603300	+
chr11	22807600	+
chr11	27484700	+
chr11	32562300	-
chr11	33018000	-
chr11	33139000	+
chr11	33713700	+
chr11	33752000	+
chr11	34084100	-
chr11	34336200	+
chr11	34417300	-
chr11	34894100	+
chr11	35640600	-
chr11	36487800	+
chr11	43290500	-
chr11	43337600	-
chr11	43622400	-
chr11	44705700	-
chr11	45783400	-
chr11	45825900	-
chr11	45896100	+
chr11	46324000	-
chr11	46571700	+
chr11	46595900	-
chr11	46824000	+
chr11	47154800	+
chr11	47193600	-
chr11	47248800	-
chr11	47386700	-
chr11	47404000	+
chr11	47530800	+
chr11	47543900	-
chr11	47556700	+
chr11	47620300	+
chr11	47745200	+
chr11	47826200	+
chr11	47959900	-
chr11	56849300	-
chr11	56859400	+
chr11	57054700	+
chr11	57091400	+
chr11	57181900	-
chr11	57192000	-
chr11	57236800	-
chr11	57266000	-
chr11	58667300	-
chr11	59139400	+
chr11	59192800	+
chr11	59279400	-
chr11	60366600	-
chr11	60430200	+
chr11	60438900	-
chr11	60685300	+
chr11	60856900	+
chr11	60886300	-
chr11	60953600	+
chr11	61316300	+
chr11	61352600	-
chr11	61415200	+
chr11	61648400	-
chr11	62098200	-
chr11	62116000	-
chr11	62125500	-
chr11	62177000	+
chr11	62189900	-
chr11	62202600	+
chr11	62277800	+
chr11	62285900	-
chr11	62329200	+
chr11	62355500	+
chr11	62379500	+
chr11	62405400	-
chr11	63061400	-
chr11	63195000	+
chr11	63206000	-
chr11	63337900	-
chr11	63364600	-
chr11	63412800	-
chr11	63463100	-
chr11	63499400	-
chr11	63510800	-
chr11	63709900	+
chr11	63731400	-
chr11	63750200	+
chr11	63770300	+
chr11	63793400	+
chr11	63809200	+
chr11	63842300	-
chr11	63864500	-
chr11	63883700	+
chr11	64334100	+
chr11	64440900	+
chr11	64449100	-
chr11	64538500	-
chr11	64571600	+
chr11	64608500	-
chr11	64658600	+
chr11	64706000	-
chr11	64858300	-
chr11	64905800	+
chr11	64941700	-
chr11	64978800	-
chr11	65064600	+
chr11	65176700	+
chr11	65244500	+
chr11	65304400	+
chr11	65357900	-
chr11	65384800	-
chr11	65415400	-
chr11	65526600	-
chr11	65576600	-
chr11	65596100	-
chr11	65625400	-
chr11	65782100	-
chr11	65792400	+
chr11	65836800	-
chr11	65868700	+
chr11	65895700	+
chr11	65991200	-
chr11	66003900	+
chr11	66069400	+
chr11	66117200	-
chr11	66201500	+
chr11	66367600	-
chr11	66380700	-
chr11	66482200	+
chr11	66644800	-
chr11	66764600	-
chr11	66876900	+
chr11	66916200	-
chr11	66952600	-
chr11	66992800	+
chr11	67032000	+
chr11	67108100	-
chr11	67130900	-
chr11	67153700	+
chr11	67555700	-
chr11	67564000	-
chr11	67795500	+
chr11	67985400	-
chr11	68367100	+
chr11	68428000	+
chr11	69199200	+
chr11	69727600	-
chr11	69794600	-
chr11	70842100	-
chr11	71317600	-
chr11	71387900	-
chr11	71468900	+
chr11	71491600	+
chr11	71614100	-
chr11	71823000	+
chr11	72140400	+
chr11	72169900	+
chr11	72202800	+
chr11	72530600	+
chr11	72765600	-
chr11	72986600	+
chr11	73149000	+
chr11	73265200	+
chr11	73559400	+
chr11	73881700	+
chr11	74137800	-
chr11	74338300	-
chr11	74740300	+
chr11	74788900	-
chr11	74913900	+
chr11	74951400	-
chr11	75204200	-
chr11	75623900	+
chr11	75769300	+
chr11	75833500	+
chr11	76455900	-
chr11	77025900	+
chr11	77208900	+
chr11	77383200	+
chr11	77467900	+
chr11	77576900	+
chr11	77963200	+
chr11	82460800	-
chr11	82582600	+
chr11	82674400	+
chr11	85017300	-
chr11	85052500	+
chr11	85198900	+
chr11	86427000	-
chr11	89595500	+
chr11	93114000	+
chr11	93157700	-
chr11	93502200	-
chr11	93866900	-
chr11	93917200	-
chr11	94345900	+
chr11	94440700	-
chr11	94462900	-
chr11	95163200	-
chr11	95762800	-
chr11	105453700	-
chr11	106833300	+
chr11	107385400	-
chr11	107497800	-
chr11	108041300	-
chr11	109671800	+
chr11	109806400	-
chr11	110978700	-
chr11	111141700	+
chr11	111462300	+
chr11	111602800	-
chr11	113251000	+
chr11	113776600	-
chr11	113815600	-
chr11	116148500	+
chr11	116473500	+
chr11	116520700	-
chr11	116555200	-
chr11	116606800	+
chr11	116704300	-
chr11	117592700	-
chr11	117735800	-
chr11	117777800	-
chr11	117907100	-
chr11	117948600	-
chr11	117984500	-
chr11	118305400	-
chr11	118373500	+
chr11	118394500	-
chr11	118405900	+
chr11	118432500	+
chr11	118444100	-
chr11	118461300	-
chr11	118470300	+
chr11	118477600	+
chr11	118483900	-
chr11	118497700	-
chr11	118582700	-
chr11	118710500	-
chr11	120400200	-
chr11	120668900	-
chr11	122032200	-
chr11	122806700	-
chr11	123117300	+
chr11	123998200	-
chr11	124121600	+
chr11	124175000	+
chr11	124945000	-
chr11	124968000	-
chr11	125263100	-
chr11	125277900	+
chr11	125586400	+
chr11	125643600	+
chr11	125658400	-
chr11	125730400	+
chr11	129270200	+
chr11	129377500	+
chr11	129445000	-
chr11	129689600	+
chr11	133411900	+
chr11	133599600	+
chr11	133628100	+
chr11	133650800	+
chr12	368000	+
chr12	928600	+
chr12	1573200	+
chr12	1776000	+
chr12	1983700	+
chr12	2792700	-
chr12	2856900	-
chr12	2870100	+
chr12	3732200	+
chr12	4517500	+
chr12	4628800	-
chr12	6449400	+
chr12	6473000	+
chr12	6513800	-
chr12	6534900	+
chr12	6547300	+
chr12	6585400	+
chr12	6668600	+
chr12	6703700	-
chr12	6732600	+
chr12	6769100	+
chr12	6831900	-
chr12	6852200	+
chr12	6894300	-
chr12	6916800	+
chr12	6923800	-
chr12	6995700	+
chr12	7152900	-
chr12	7174400	-
chr12	7233700	-
chr12	8076900	-
chr12	8126200	-
chr12	8741700	-
chr12	8958600	-
chr12	8993300	+
chr12	11215200	+
chr12	12401900	-
chr12	12857900	-
chr12	13044900	-
chr12	14818700	-
chr12	14847300	+
chr12	15926900	-
chr12	19484800	-
chr12	21546300	-
chr12	21701400	+
chr12	22090900	-
chr12	22588100	+
chr12	22669800	-
chr12	25294700	+
chr12	26877000	+
chr12	27058100	+
chr12	27067300	-
chr12	27755300	-
chr12	27824600	-
chr12	28235200	-
chr12	29424900	+
chr12	30739500	+
chr12	30798700	+
chr12	32004700	-
chr12	32723900	-
chr12	32799700	+
chr12	38123000	+
chr12	38786100	+
chr12	40824500	+
chr12	40917800	+
chr12	41006700	-
chr12	42439200	-
chr12	42486100	+
chr12	45759600	+
chr12	46385800	+
chr12	46497900	+
chr12	46584400	+
chr12	46785800	+
chr12	46837300	+
chr12	47030400	+
chr12	47361800	+
chr12	47396200	+
chr12	47495300	-
chr12	47531900	+
chr12	47604900	+
chr12	47637000	+
chr12	47698700	+
chr12	47790100	+
chr12	47811000	+
chr12	47945300	-
chr12	48386800	+
chr12	48422000	-
chr12	48508200	+
chr12	48522800	+
chr12	48765500	-
chr12	48792300	-
chr12	48846900	+
chr12	49081500	-
chr12	49185500	-
chr12	49444600	-
chr12	49705700	+
chr12	49763200	+
chr12	49852600	+
chr12	49919200	-
chr12	50704700	+
chr12	50731600	-
chr12	50750200	-
chr12	51686900	-
chr12	51726200	+
chr12	51759100	+
chr12	51861100	-
chr12	51886100	+
chr12	51900000	+
chr12	51912500	-
chr12	51932400	-
chr12	51976000	-
chr12	52173200	-
chr12	52181300	-
chr12	52305800	+
chr12	52355800	+
chr12	52407200	+
chr12	52896200	+
chr12	53178500	-
chr12	53230000	-
chr12	54396400	-
chr12	54409300	-
chr12	54423800	-
chr12	54498200	-
chr12	54509800	+
chr12	54647200	-
chr12	54654200	-
chr12	54722400	-
chr12	54784700	-
chr12	54799300	-
chr12	54809100	-
chr12	54838900	-
chr12	54869200	+
chr12	54901700	+
chr12	54938000	+
chr12	54980000	+
chr12	54995800	+
chr12	55019200	-
chr12	55039900	+
chr12	55315500	+
chr12	55325600	+
chr12	55404800	+
chr12	55769200	-
chr12	55791100	+
chr12	55910100	-
chr12	56157300	+
chr12	56168200	-
chr12	56203300	-
chr12	56226800	+
chr12	56271600	-
chr12	56374400	-
chr12	56432100	+
chr12	56452200	+
chr12	56462700	-
chr12	56525700	+
chr12	58276700	-
chr12	60940600	-
chr12	61147100	-
chr12	63084900	-
chr12	63850300	-
chr12	64810400	+
chr12	64849600	+
chr12	64985000	-
chr12	65949600	-
chr12	66378800	+
chr12	67367100	-
chr12	67920000	-
chr12	68040100	-
chr12	68150700	-
chr12	68265800	-
chr12	68419400	-
chr12	68923700	-
chr12	69046700	-
chr12	70435600	-
chr12	70519800	-
chr12	73218700	-
chr12	74191400	+
chr12	74711500	+
chr12	74764100	+
chr12	75477200	+
chr12	75682400	-
chr12	78852300	+
chr12	81276300	+
chr12	86953800	-
chr12	88269400	+
chr12	88443100	+
chr12	91846500	+
chr12	92296400	-
chr12	92359400	+
chr12	92385800	-
chr12	92491500	-
chr12	93067400	-
chr12	93921400	+
chr12	93990800	+
chr12	94135800	-
chr12	94392100	-
chr12	94777200	-
chr12	94953200	+
chr12	95114000	-
chr12	95316500	+
chr12	95825400	-
chr12	97512000	-
chr12	97563200	-
chr12	99118400	-
chr12	99185700	-
chr12	100325400	+
chr12	102758700	+
chr12	102874700	+
chr12	102884000	-
chr12	102982700	-
chr12	103055600	+
chr12	103206200	-
chr12	103221900	-
chr12	103395700	-
chr12	103638600	-
chr12	104026200	-
chr12	104153600	+
chr12	104249100	-
chr12	105165800	+
chr12	105220900	-
chr12	105692800	-
chr12	105874100	-
chr12	105904800	+
chr12	106011200	+
chr12	106603600	-
chr12	106678700	+
chr12	107433500	-
chr12	107649000	+
chr12	107775300	+
chr12	107975200	+
chr12	108015300	+
chr12	108021100	-
chr12	108399400	+
chr12	108802300	+
chr12	108822900	-
chr12	108922000	-
chr12	108970300	+
chr12	109203800	-
chr12	109325600	+
chr12	109372000	+
chr12	109391100	-
chr12	109423800	+
chr12	109504900	+
chr12	109611100	+
chr12	110520900	+
chr12	110607900	+
chr12	110764700	-
chr12	110935800	-
chr12	111030500	+
chr12	111048100	-
chr12	111304000	+
chr12	111330700	+
chr12	111341500	-
chr12	111830200	-
chr12	111980700	-
chr12	112107900	-
chr12	112256700	+
chr12	112281200	-
chr12	112888100	+
chr12	115659800	+
chr12	116112200	+
chr12	117058600	-
chr12	117281100	+
chr12	117299300	-
chr12	118590200	-
chr12	118912000	-
chr12	119038500	+
chr12	119116600	+
chr12	119368800	-
chr12	119391200	+
chr12	119418600	-
chr12	119609700	-
chr12	119633000	-
chr12	119648300	-
chr12	119825700	+
chr12	119960600	+
chr12	120218700	+
chr12	120274200	+
chr12	120322600	-
chr12	120459700	+
chr12	120635100	-
chr12	120811500	-
chr12	121001700	-
chr12	121277200	-
chr12	121472700	+
chr12	121550800	+
chr12	121803700	-
chr12	121886600	-
chr12	122025900	-
chr12	122126200	+
chr12	122199900	+
chr12	122284100	-
chr12	122415000	+
chr12	122439500	-
chr12	122486600	+
chr12	122635100	-
chr12	122652800	-
chr12	122684500	-
chr12	122763600	-
chr12	123023000	+
chr12	123517200	+
chr12	123978400	-
chr12	124044700	-
chr12	127873900	+
chr12	129889100	+
chr12	129922900	-
chr12	130761800	-
chr12	130946000	-
chr12	130980000	-
chr12	131000800	-
chr12	131135100	-
chr12	131240200	+
chr12	131797600	-
chr12	131848100	+
chr12	131914900	+
chr13	19106100	-
chr13	19254600	+
chr13	19432500	-
chr13	19997700	+
chr13	20245400	+
chr13	20374600	+
chr13	20613000	-
chr13	20649200	-
chr13	20930800	+
chr13	21075800	+
chr13	22846500	+
chr13	23743900	-
chr13	23984000	+
chr13	24759000	+
chr13	24774000	-
chr13	25693800	+
chr13	26724100	-
chr13	27094200	-
chr13	27611000	-
chr13	28131500	-
chr13	29067000	+
chr13	30209900	-
chr13	30633700	+
chr13	31898100	+
chr13	32003300	+
chr13	32010400	+
chr13	36291700	-
chr13	36531500	+
chr13	37822100	-
chr13	38509800	+
chr13	40242700	+
chr13	40604500	+
chr13	40666200	+
chr13	40783900	-
chr13	41744700	-
chr13	42495900	-
chr13	44461700	-
chr13	44592800	-
chr13	44813000	+
chr13	44889800	+
chr13	44937300	-
chr13	45524500	+
chr13	46026000	-
chr13	46268700	+
chr13	47472900	+
chr13	47510000	+
chr13	47566600	+
chr13	47776200	-
chr13	47791800	+
chr13	48968400	-
chr13	49057400	+
chr13	49163000	+
chr13	49264600	+
chr13	49469500	-
chr13	50926500	-
chr13	51056900	-
chr13	51276500	-
chr13	51484500	-
chr13	51921200	+
chr13	51927700	-
chr13	52125200	-
chr13	72199500	+
chr13	72253800	+
chr13	75009600	+
chr13	75022100	-
chr13	75108600	-
chr13	76498700	+
chr13	76801700	+
chr13	77170000	-
chr13	78131100	+
chr13	78877800	+
chr13	78953700	-
chr13	90799600	-
chr13	94046000	+
chr13	95127800	-
chr13	96673800	-
chr13	97426900	-
chr13	98537600	+
chr13	98688300	-
chr13	98952200	-
chr13	99539800	-
chr13	100124700	+
chr13	102049500	-
chr13	102296800	-
chr13	107669100	-
chr13	110066400	-
chr13	110156600	-
chr13	110365000	+
chr13	110604400	-
chr13	112391900	-
chr13	112671100	+
chr13	112855900	+
chr13	112999800	-
chr13	113192500	+
chr13	113288000	-
chr13	113564200	-
chr13	113595100	+
chr13	114018700	-
chr13	114065700	-
chr14	19843400	+
chr14	19993300	-
chr14	19999100	+
chr14	20008200	-
chr14	20528000	-
chr14	20562600	+
chr14	20921700	+
chr14	20974900	+
chr14	20993500	+
chr14	21015300	-
chr14	22127600	+
chr14	22305900	-
chr14	22426400	-
chr14	22457700	+
chr14	22468300	+
chr14	22573600	+
chr14	22633900	+
chr14	22840700	+
chr14	22860900	-
chr14	23008700	-
chr14	23095500	-
chr14	23106300	+
chr14	23492700	+
chr14	23591300	-
chr14	23633800	-
chr14	23675800	-
chr14	23686000	+
chr14	23711300	+
chr14	23734400	+
chr14	23755100	-
chr14	23771900	-
chr14	23781300	+
chr14	23810400	+
chr14	23839000	-
chr14	23849900	+
chr14	23878800	+
chr14	23969400	-
chr14	23981300	+
chr14	30098500	-
chr14	30161500	-
chr14	30565200	-
chr14	30746100	+
chr14	34078200	+
chr14	34168500	+
chr14	34413200	+
chr14	34521800	-
chr14	34585600	-
chr14	34661100	+
chr14	34832200	-
chr14	38806300	-
chr14	44623400	-
chr14	49122100	+
chr14	49157500	-
chr14	49304800	-
chr14	49389100	+
chr14	49767900	+
chr14	49849100	-
chr14	50068600	+
chr14	50367000	+
chr14	51526300	-
chr14	52089200	+
chr14	52231700	+
chr14	52244000	-
chr14	52267000	-
chr14	52327600	+
chr14	52645500	-
chr14	52688700	+
chr14	53977400	+
chr14	54024800	+
chr14	54046500	-
chr14	54438700	+
chr14	54563900	-
chr14	54588600	-
chr14	54808500	-
chr14	54947700	+
chr14	55117200	-
chr14	55655600	-
chr14	56116500	-
chr14	56805700	-
chr14	56927500	-
chr14	57737000	-
chr14	57781700	-
chr14	58725600	-
chr14	59000900	+
chr14	59020500	+
chr14	59628800	-
chr14	60817000	+
chr14	61299000	-
chr14	63079700	+
chr14	63263800	+
chr14	63390300	-
chr14	64002200	-
chr14	64451600	-
chr14	66045000	-
chr14	66896900	-
chr14	67024600	+
chr14	67136500	+
chr14	67210700	+
chr14	67231500	+
chr14	68317700	-
chr14	68514700	+
chr14	68688300	+
chr14	68934700	+
chr14	69303800	-
chr14	69895300	+
chr14	69953000	+
chr14	70136800	+
chr14	70345500	+
chr14	70444500	-
chr14	70857800	-
chr14	72463200	-
chr14	72595100	-
chr14	72673300	-
chr14	73028200	-
chr14	73105500	+
chr14	73249700	-
chr14	73321800	+
chr14	73423500	-
chr14	74249300	+
chr14	74300600	-
chr14	74418500	-
chr14	74539700	-
chr14	74663100	+
chr14	74712500	+
chr14	75058900	-
chr14	75114800	-
chr14	75518100	+
chr14	75688400	-
chr14	76348400	+
chr14	76492700	-
chr14	76634500	-
chr14	76994300	-
chr14	77152100	+
chr14	77244300	-
chr14	77296500	+
chr14	81069600	+
chr14	87543400	-
chr14	88099700	-
chr14	88953700	-
chr14	89154400	+
chr14	89492100	-
chr14	89793100	-
chr14	90596400	+
chr14	91403000	-
chr14	91575900	+
chr14	91642100	+
chr14	91658300	-
chr14	92188300	+
chr14	92330700	-
chr14	92649000	-
chr14	92720900	+
chr14	92742900	+
chr14	92868700	+
chr14	94693200	+
chr14	95052100	+
chr14	95070900	-
chr14	95899000	+
chr14	96038500	-
chr14	96333800	-
chr14	99018400	-
chr14	99140600	+
chr14	99750100	+
chr14	99912700	-
chr14	101298400	-
chr14	101484400	-
chr14	101501300	-
chr14	101623800	+
chr14	101676200	-
chr14	101856200	-
chr14	101898800	+
chr14	102128500	-
chr14	102314300	-
chr14	102921900	-
chr14	103098300	+
chr14	103383400	+
chr14	103457500	+
chr14	104290900	-
chr14	104353000	+
chr14	104558100	+
chr14	104785800	+
chr14	104837900	+
chr14	104852700	-
chr14	104936100	-
chr15	20385200	-
chr15	20585400	+
chr15	20637300	+
chr15	23235000	+
chr15	27349000	+
chr15	29070600	+
chr15	29471900	+
chr15	29552700	+
chr15	29563000	+
chr15	32180800	+
chr15	32289300	+
chr15	32304700	-
chr15	32416600	+
chr15	32446400	+
chr15	32662100	+
chr15	33048900	+
chr15	33067400	+
chr15	38118200	+
chr15	38187900	+
chr15	38386900	+
chr15	38403400	-
chr15	38437900	-
chr15	38447400	+
chr15	38462700	-
chr15	38485500	-
chr15	38550900	-
chr15	38649400	-
chr15	38774300	+
chr15	38834200	+
chr15	38843800	-
chr15	38887100	-
chr15	38923800	-
chr15	38974300	-
chr15	38986900	+
chr15	39021700	-
chr15	39195400	+
chr15	39310900	-
chr15	39364000	-
chr15	39496900	-
chr15	39623600	+
chr15	39740500	-
chr15	39854300	-
chr15	40051200	+
chr15	40287200	+
chr15	40352600	+
chr15	40627900	+
chr15	40816000	+
chr15	41185100	+
chr15	41213600	-
chr15	41265300	-
chr15	41856400	+
chr15	41872300	-
chr15	42368400	-
chr15	42506900	-
chr15	42616600	-
chr15	43246200	+
chr15	43267100	+
chr15	43482400	-
chr15	43537100	+
chr15	43667000	-
chr15	43715200	-
chr15	46411100	-
chr15	47234900	+
chr15	48434600	-
chr15	48504100	-
chr15	48765700	+
chr15	49816500	+
chr15	49909800	-
chr15	50099400	-
chr15	50259000	+
chr15	50608300	+
chr15	50647900	+
chr15	53276000	+
chr15	53335800	-
chr15	53487300	+
chr15	54812500	+
chr15	54998100	+
chr15	55786400	-
chr15	56828500	+
chr15	56851300	-
chr15	57012500	+
chr15	57067700	-
chr15	57736100	+
chr15	57768300	+
chr15	58477100	+
chr15	58558100	+
chr15	60139700	+
chr15	61236300	+
chr15	61269200	-
chr15	61679700	+
chr15	61912900	+
chr15	62172700	+
chr15	62242000	+
chr15	62435000	+
chr15	62540500	-
chr15	62676000	-
chr15	62782200	+
chr15	63068700	+
chr15	63108400	+
chr15	63212600	+
chr15	63264000	+
chr15	63365700	+
chr15	63596700	+
chr15	63610300	-
chr15	63949600	-
chr15	64466800	-
chr15	64576700	+
chr15	64584000	+
chr15	65213200	+
chr15	65333700	+
chr15	65600900	-
chr15	66134100	-
chr15	66308700	+
chr15	66357600	-
chr15	66896400	+
chr15	67532500	-
chr15	70351700	+
chr15	70455200	+
chr15	70862700	+
chr15	71712200	+
chr15	72006700	+
chr15	72074100	+
chr15	72620800	-
chr15	72678300	-
chr15	72922100	+
chr15	72952400	+
chr15	72969400	-
chr15	73016800	+
chr15	73281900	-
chr15	73447400	+
chr15	73719200	+
chr15	73923600	-
chr15	73983600	-
chr15	74390600	+
chr15	75011300	-
chr15	75075900	-
chr15	75500000	-
chr15	76148700	+
chr15	76156000	+
chr15	76229100	-
chr15	76344500	-
chr15	76378700	+
chr15	76517700	-
chr15	76620400	-
chr15	76953000	-
chr15	77975900	+
chr15	78139300	-
chr15	78775100	-
chr15	79068800	+
chr15	79080800	-
chr15	79403100	+
chr15	80341600	+
chr15	81216700	-
chr15	81276000	-
chr15	81446300	-
chr15	81471100	+
chr15	82998500	+
chr15	83060200	+
chr15	83094700	-
chr15	83324800	+
chr15	83725200	-
chr15	83927400	+
chr15	84138400	+
chr15	86811300	+
chr15	86966100	-
chr15	86983900	-
chr15	87239400	-
chr15	87432800	-
chr15	87678900	+
chr15	88346300	-
chr15	88445800	+
chr15	88546000	-
chr15	88610200	-
chr15	88733000	-
chr15	88875000	-
chr15	89061700	-
chr15	89216600	-
chr15	89279500	-
chr15	89366500	+
chr15	90197900	+
chr15	97212900	+
chr15	97226600	+
chr15	97609400	-
chr15	97924100	-
chr15	98090700	+
chr15	98699300	+
chr15	98959800	+
chr15	99609000	+
chr15	99634700	+
chr15	99652400	+
chr15	100009700	+
chr15	100081300	+
chr16	43200	+
chr16	68400	-
chr16	219000	+
chr16	225100	-
chr16	344600	+
chr16	391000	+
chr16	416000	-
chr16	638700	+
chr16	657400	-
chr16	670100	-
chr16	685100	-
chr16	730600	+
chr16	798000	-
chr16	987800	-
chr16	1410600	+
chr16	1483600	-
chr16	1602600	-
chr16	1668500	-
chr16	1696500	-
chr16	1816500	+
chr16	1932800	+
chr16	1954700	+
chr16	1974300	-
chr16	2038100	-
chr16	2195600	-
chr16	2204300	+
chr16	2259100	-
chr16	2330800	-
chr16	2419600	-
chr16	2743000	-
chr16	2767100	+
chr16	2873100	-
chr16	2895000	-
chr16	2902300	-
chr16	3048700	+
chr16	3114700	-
chr16	3124700	+
chr16	3139900	+
chr16	3148500	+
chr16	3224900	+
chr16	3254600	-
chr16	3448500	-
chr16	3566500	+
chr16	3601800	-
chr16	3707200	+
chr16	4262300	+
chr16	4297400	+
chr16	4406000	+
chr16	4416000	-
chr16	4466900	-
chr16	4528000	+
chr16	4615300	-
chr16	4683700	-
chr16	4724300	+
chr16	4757100	+
chr16	4792400	+
chr16	4837300	+
chr16	5023400	+
chr16	8623500	-
chr16	8676000	-
chr16	8799400	-
chr16	8869600	+
chr16	10388200	-
chr16	10745600	-
chr16	11743700	+
chr16	11798800	-
chr16	11852600	+
chr16	11915500	+
chr16	11978300	-
chr16	14634700	-
chr16	15056900	+
chr16	15643900	+
chr16	15652000	-
chr16	15889600	+
chr16	15951500	-
chr16	17471400	+
chr16	18708600	+
chr16	18720000	+
chr16	19443200	-
chr16	19636500	+
chr16	20659900	+
chr16	20819700	-
chr16	21518900	-
chr16	21871800	+
chr16	21927100	-
chr16	22125700	-
chr16	22216600	-
chr16	22292800	+
chr16	23371500	+
chr16	23428600	+
chr16	23476600	-
chr16	23514800	+
chr16	23560400	-
chr16	23597300	+
chr16	23755900	-
chr16	24649100	-
chr16	24933900	+
chr16	24950600	-
chr16	25030700	-
chr16	25177200	-
chr16	27234000	-
chr16	27468400	+
chr16	28111700	+
chr16	28129700	+
chr16	28211800	-
chr16	28473300	-
chr16	28742300	-
chr16	28765100	+
chr16	28783000	-
chr16	28844000	+
chr16	28893800	-
chr16	29709800	-
chr16	29740100	-
chr16	29781600	+
chr16	29844700	+
chr16	29893400	-
chr16	29914800	-
chr16	29995200	-
chr16	30014500	+
chr16	30273800	+
chr16	30287500	-
chr16	30314600	-
chr16	30326800	-
chr16	30336300	+
chr16	30348600	+
chr16	30364100	+
chr16	30392600	-
chr16	30569800	-
chr16	30617600	-
chr16	30667300	-
chr16	30681100	-
chr16	30694100	+
chr16	30812500	+
chr16	30868400	-
chr16	30876800	-
chr16	30952400	-
chr16	30992300	+
chr16	31013400	+
chr16	31027300	-
chr16	31036800	-
chr16	31099300	-
chr16	31120900	+
chr16	31377800	+
chr16	31395600	+
chr16	31406800	-
chr16	31427000	+
chr16	31633500	-
chr16	45280500	+
chr16	45564400	+
chr16	46052400	+
chr16	46836000	-
chr16	46957500	+
chr16	48658000	-
chr16	48866100	-
chr16	49140100	-
chr16	49271700	+
chr16	49333900	-
chr16	52094100	+
chr16	52295600	-
chr16	55016200	+
chr16	55042800	+
chr16	55111000	+
chr16	55274100	-
chr16	55321700	-
chr16	55524300	-
chr16	55778000	-
chr16	55836700	-
chr16	56038400	+
chr16	56054300	-
chr16	56127800	+
chr16	56327300	-
chr16	56720200	+
chr16	56789100	+
chr16	57107200	-
chr16	57220800	+
chr16	57324900	+
chr16	65144300	-
chr16	65342600	+
chr16	65421400	+
chr16	65472400	-
chr16	65525400	+
chr16	65592400	-
chr16	65621000	-
chr16	65701700	-
chr16	65751600	-
chr16	65775800	-
chr16	65783700	-
chr16	65818600	-
chr16	65838500	+
chr16	65870600	+
chr16	66073300	-
chr16	66155500	-
chr16	66237100	-
chr16	66251500	+
chr16	66310600	+
chr16	66397700	+
chr16	66424900	+
chr16	66438100	+
chr16	66464800	-
chr16	66485000	-
chr16	66559500	+
chr16	66614300	+
chr16	66829100	-
chr16	66836900	-
chr16	66856300	-
chr16	66902000	+
chr16	67724000	-
chr16	67779100	-
chr16	67921600	+
chr16	67930700	+
chr16	68016300	-
chr16	68345800	+
chr16	68843000	+
chr16	68938500	-
chr16	68972000	+
chr16	69030000	+
chr16	69115400	-
chr16	69392100	+
chr16	69880800	+
chr16	70053300	+
chr16	70075900	+
chr16	70399900	+
chr16	70437700	-
chr16	70475000	+
chr16	70487000	-
chr16	70685300	-
chr16	72888400	-
chr16	73198100	-
chr16	73258000	+
chr16	73576000	+
chr16	73591100	-
chr16	74024900	+
chr16	74055400	+
chr16	74157900	-
chr16	75782500	-
chr16	75804000	-
chr16	76691300	-
chr16	79597800	+
chr16	79627600	-
chr16	79905800	+
chr16	80037400	-
chr16	80760900	+
chr16	82399500	-
chr16	82490500	-
chr16	82707600	+
chr16	82735800	+
chr16	82777500	+
chr16	83096000	-
chr16	83291700	-
chr16	83602300	+
chr16	83619500	-
chr16	83646800	-
chr16	84390500	+
chr16	85145900	+
chr16	85908800	-
chr16	85974400	+
chr16	85983700	-
chr16	86356600	+
chr16	87164700	-
chr16	87244600	+
chr16	87256900	+
chr16	87299900	+
chr16	87405500	+
chr16	87451500	-
chr16	87811800	-
chr16	88085100	-
chr16	88100600	-
chr16	88251600	+
chr16	88467900	-
chr16	88566700	-
chr17	260600	-
chr17	564400	+
chr17	601900	+
chr17	632500	-
chr17	847600	-
chr17	958700	+
chr17	1249300	+
chr17	1305600	+
chr17	1366300	+
chr17	1499300	-
chr17	1533900	+
chr17	1566800	+
chr17	1679800	+
chr17	1880500	-
chr17	1892200	-
chr17	1939700	+
chr17	2153400	+
chr17	2186100	+
chr17	2361200	+
chr17	2444400	-
chr17	2561500	+
chr17	2664900	-
chr17	3486500	-
chr17	3518700	-
chr17	3545600	+
chr17	3695800	+
chr17	3742800	+
chr17	3993300	+
chr17	4113500	+
chr17	4216000	+
chr17	4405000	+
chr17	4553800	+
chr17	4561100	-
chr17	4565500	-
chr17	4581800	-
chr17	4640000	+
chr17	4683800	-
chr17	4743600	-
chr17	4811900	-
chr17	4831700	-
chr17	4842200	-
chr17	4922700	-
chr17	4955400	+
chr17	5126700	-
chr17	5263900	-
chr17	5282700	+
chr17	5312400	+
chr17	5330100	+
chr17	5427900	+
chr17	6484500	+
chr17	6495400	+
chr17	6600600	-
chr17	6859000	-
chr17	6880100	-
chr17	6887200	+
chr17	7049200	+
chr17	7064300	-
chr17	7077800	+
chr17	7086200	+
chr17	7106100	+
chr17	7173400	+
chr17	7180700	-
chr17	7238200	+
chr17	7247800	+
chr17	7280200	-
chr17	7300000	-
chr17	7323000	+
chr17	7328900	-
chr17	7406200	-
chr17	7427700	+
chr17	7458500	+
chr17	7471400	+
chr17	7679100	-
chr17	7701700	-
chr17	7732200	-
chr17	7759700	+
chr17	7776100	+
chr17	8006400	+
chr17	8020000	+
chr17	8030700	-
chr17	8092800	-
chr17	8133000	-
chr17	8220400	+
chr17	8227100	+
chr17	8280300	-
chr17	8809000	+
chr17	10541500	+
chr17	12861800	+
chr17	13913600	-
chr17	15844300	-
chr17	16061100	-
chr17	16283500	-
chr17	16413000	+
chr17	16887100	-
chr17	17080800	+
chr17	17124700	+
chr17	17321100	-
chr17	17340100	+
chr17	17526500	-
chr17	17538500	+
chr17	17596600	-
chr17	17627900	-
chr17	17680300	+
chr17	17816000	+
chr17	17883100	+
chr17	17932100	-
chr17	18102100	+
chr17	18847600	+
chr17	19221700	+
chr17	19821300	+
chr17	19999800	+
chr17	20886600	+
chr17	20971100	-
chr17	21096800	+
chr17	22646100	-
chr17	22807900	-
chr17	22982600	-
chr17	23686700	+
chr17	23708900	-
chr17	23723300	-
chr17	23736100	-
chr17	23922800	-
chr17	23995800	+
chr17	24012900	+
chr17	24071400	-
chr17	24079400	-
chr17	24095900	-
chr17	24163300	+
chr17	24193500	+
chr17	24206200	-
chr17	24248200	+
chr17	24304100	-
chr17	24742100	-
chr17	24920000	-
chr17	25111800	+
chr17	25281000	+
chr17	25468200	-
chr17	25642700	+
chr17	25730500	-
chr17	25829100	-
chr17	26174900	+
chr17	26861000	-
chr17	27209900	+
chr17	27252400	+
chr17	27493900	-
chr17	27702100	-
chr17	27795800	-
chr17	27838700	-
chr17	30313100	-
chr17	30331900	-
chr17	30440000	+
chr17	30470300	+
chr17	30919600	-
chr17	30938900	-
chr17	31115700	-
chr17	31160800	-
chr17	31916900	-
chr17	31975200	-
chr17	32032600	-
chr17	32380900	-
chr17	32789900	+
chr17	32924100	-
chr17	33077100	+
chr17	33761900	-
chr17	33969200	-
chr17	34140600	-
chr17	34162400	-
chr17	34209100	+
chr17	34234700	+
chr17	34607200	+
chr17	34811100	+
chr17	34860900	+
chr17	34872200	-
chr17	35140000	+
chr17	35390900	-
chr17	35473400	-
chr17	35479300	-
chr17	35509600	+
chr17	35533100	-
chr17	35550500	-
chr17	35629200	-
chr17	36057500	+
chr17	37221400	+
chr17	37274500	+
chr17	37328400	+
chr17	37372500	-
chr17	37422600	+
chr17	37517700	+
chr17	37560500	+
chr17	37589600	+
chr17	37654400	+
chr17	37692400	+
chr17	37793800	+
chr17	37864600	-
chr17	37942300	-
chr17	37960100	+
chr17	37968100	-
chr17	37983800	-
chr17	38014400	+
chr17	38084500	+
chr17	38150300	+
chr17	38203800	+
chr17	38229600	+
chr17	38239000	-
chr17	38370000	+
chr17	38386500	-
chr17	38404100	-
chr17	38427600	+
chr17	38531200	-
chr17	38576200	-
chr17	38832200	-
chr17	38917300	-
chr17	39440100	-
chr17	39447300	+
chr17	39556100	+
chr17	39619500	+
chr17	39631400	+
chr17	39757400	+
chr17	39778700	-
chr17	39935900	+
chr17	40208800	-
chr17	40332100	+
chr17	40494300	-
chr17	40565500	+
chr17	40594200	+
chr17	40695300	-
chr17	40749600	+
chr17	40843000	+
chr17	42355900	-
chr17	43126800	-
chr17	43253600	+
chr17	43263500	+
chr17	43273300	+
chr17	43328600	-
chr17	43374200	-
chr17	43403500	-
chr17	43533300	+
chr17	43540300	-
chr17	43862400	-
chr17	44263800	-
chr17	44341600	-
chr17	44376600	+
chr17	44642400	+
chr17	44793700	+
chr17	44846900	+
chr17	45140100	+
chr17	45172300	+
chr17	45221200	-
chr17	45488500	-
chr17	45527800	-
chr17	45593700	-
chr17	45778900	-
chr17	45804900	+
chr17	45829400	+
chr17	45911700	-
chr17	45974100	+
chr17	46139900	+
chr17	46152500	-
chr17	46552800	+
chr17	46586200	-
chr17	46599100	-
chr17	46692700	+
chr17	50400500	+
chr17	52267100	-
chr17	52345800	+
chr17	52518100	-
chr17	52690500	-
chr17	53282300	+
chr17	53420100	+
chr17	53439300	+
chr17	53516700	-
chr17	53784900	-
chr17	53950100	+
chr17	54538500	+
chr17	54642100	-
chr17	54764800	-
chr17	54998000	-
chr17	55052000	-
chr17	55139300	+
chr17	55325300	-
chr17	55567300	+
chr17	55823900	+
chr17	55957700	+
chr17	56032800	-
chr17	57497100	+
chr17	58981800	-
chr17	59032100	-
chr17	59054100	-
chr17	59205300	-
chr17	59258200	+
chr17	59273400	+
chr17	59362700	+
chr17	59429600	-
chr17	59560800	+
chr17	59577400	-
chr17	59693600	+
chr17	59923300	+
chr17	60482600	+
chr17	61730900	-
chr17	62672600	+
chr17	62792700	+
chr17	62805000	+
chr17	63144900	-
chr17	63610000	-
chr17	63690400	-
chr17	63756100	-
chr17	64020600	-
chr17	68700900	-
chr17	68740000	+
chr17	68819300	+
chr17	69721200	-
chr17	70284200	-
chr17	70380700	+
chr17	70489800	+
chr17	70520600	-
chr17	70555200	-
chr17	70638700	+
chr17	70661700	+
chr17	70713400	-
chr17	70768900	+
chr17	70779100	-
chr17	70796900	+
chr17	70901900	-
chr17	70913000	+
chr17	71024700	-
chr17	71033000	+
chr17	71096800	-
chr17	71174900	-
chr17	71272500	+
chr17	71351300	+
chr17	71362800	+
chr17	71385000	+
chr17	71403700	+
chr17	71579800	+
chr17	71611100	+
chr17	71748000	+
chr17	71861200	+
chr17	71960400	+
chr17	72008600	+
chr17	72065600	-
chr17	72234200	+
chr17	72596600	-
chr17	72648900	-
chr17	73174800	-
chr17	73465100	+
chr17	73676800	-
chr17	73886600	-
chr17	74487200	+
chr17	74517000	+
chr17	74583100	-
chr17	75690000	-
chr17	75734900	+
chr17	75807600	+
chr17	76004000	-
chr17	76133700	-
chr17	76581100	-
chr17	76873900	-
chr17	76883500	+
chr17	77130100	+
chr17	77214100	+
chr17	77243700	+
chr17	77280300	+
chr17	77384000	+
chr17	77528800	-
chr17	77616400	+
chr17	77657400	+
chr17	77885100	+
chr17	77970000	+
chr17	78048400	+
chr17	78071300	-
chr17	78249300	+
chr17	78268200	-
chr17	78303400	-
chr18	148600	-
chr18	257900	+
chr18	701900	+
chr18	2560900	+
chr18	2897100	-
chr18	3252600	-
chr18	3584800	-
chr18	5228200	-
chr18	5284300	+
chr18	9093300	-
chr18	9465700	-
chr18	9604000	+
chr18	10516300	-
chr18	11841200	-
chr18	12366800	+
chr18	12693000	-
chr18	12981600	-
chr18	13208200	+
chr18	13372600	-
chr18	13454600	+
chr18	13601800	-
chr18	13716800	-
chr18	16945400	+
chr18	17434100	+
chr18	17446800	-
chr18	17537800	+
chr18	17575300	-
chr18	19287700	-
chr18	19337900	-
chr18	19420200	+
chr18	19849900	-
chr18	19972700	+
chr18	21924300	+
chr18	22382100	+
chr18	27776800	+
chr18	30875900	-
chr18	31075600	-
chr18	31124200	-
chr18	31177700	+
chr18	31331200	+
chr18	31416000	-
chr18	31806700	-
chr18	37789700	-
chr18	41521700	-
chr18	41801000	+
chr18	41905900	+
chr18	41931700	+
chr18	42008500	-
chr18	42751100	+
chr18	42956200	+
chr18	43710900	+
chr18	44729600	+
chr18	45240500	+
chr18	45267100	+
chr18	45594200	-
chr18	46047100	-
chr18	46061500	+
chr18	46067600	+
chr18	46659800	-
chr18	46811000	-
chr18	46976100	+
chr18	50004200	+
chr18	52456200	+
chr18	54958400	-
chr18	55177100	+
chr18	58005700	-
chr18	58533300	-
chr18	58956600	+
chr18	58973400	+
chr18	59017900	+
chr18	59240400	+
chr18	59788800	-
chr18	69966600	-
chr18	70110000	+
chr18	70415900	+
chr18	71049300	+
chr18	71088700	-
chr18	72662800	+
chr18	74930300	-
chr18	75825500	+
chr18	75895200	-
chr18	75968600	-
chr19	294800	+
chr19	523200	-
chr19	877000	-
chr19	1082600	+
chr19	1157800	-
chr19	1306300	-
chr19	1543100	+
chr19	1556200	+
chr19	1763800	-
chr19	2012100	+
chr19	2115800	-
chr19	2241500	-
chr19	2378300	+
chr19	2734000	+
chr19	2901200	+
chr19	3312400	-
chr19	3385700	+
chr19	3451400	+
chr19	3457600	-
chr19	3508100	+
chr19	3577300	+
chr19	3713000	+
chr19	3921600	+
chr19	4074500	+
chr19	4133100	+
chr19	4294600	-
chr19	4331500	+
chr19	4408000	+
chr19	4590800	-
chr19	4674600	+
chr19	4782200	+
chr19	4866900	+
chr19	4921600	-
chr19	5631500	+
chr19	5670800	+
chr19	5741900	+
chr19	5855100	+
chr19	6150600	+
chr19	6313400	-
chr19	6344100	+
chr19	6375200	+
chr19	6432200	+
chr19	6620800	-
chr19	6688500	-
chr19	6718200	+
chr19	6724500	-
chr19	6752900	-
chr19	7494000	-
chr19	7506200	-
chr19	7600500	+
chr19	7651500	-
chr19	7874900	-
chr19	7914300	+
chr19	8180600	-
chr19	8279100	+
chr19	8292600	-
chr19	8361700	-
chr19	8416400	-
chr19	8484700	+
chr19	8547500	-
chr19	8581100	+
chr19	9555500	+
chr19	9790500	+
chr19	9799800	-
chr19	9807300	-
chr19	9829500	+
chr19	10058200	-
chr19	10165900	+
chr19	10202300	+
chr19	10224000	-
chr19	10242700	-
chr19	10311200	+
chr19	10351900	+
chr19	10374900	+
chr19	10474000	+
chr19	10516000	-
chr19	10575400	-
chr19	10626400	-
chr19	10690000	-
chr19	10807500	+
chr19	10900500	-
chr19	11062000	-
chr19	11310600	+
chr19	11346400	+
chr19	11352700	+
chr19	11407500	-
chr19	11477700	-
chr19	11530700	+
chr19	11550500	+
chr19	11960100	-
chr19	12112000	-
chr19	12135200	-
chr19	12266300	+
chr19	12522700	+
chr19	12583100	-
chr19	12638000	+
chr19	12653100	+
chr19	12667800	+
chr19	12694600	+
chr19	12709300	-
chr19	12773100	+
chr19	12853200	+
chr19	12863200	-
chr19	12905200	+
chr19	12910700	-
chr19	13075800	+
chr19	13720000	-
chr19	13736700	-
chr19	13746600	-
chr19	14003400	+
chr19	14088900	+
chr19	14354200	-
chr19	14501900	-
chr19	15096800	+
chr19	15350700	+
chr19	15390500	+
chr19	15403900	+
chr19	15435700	+
chr19	16083800	-
chr19	16115500	-
chr19	16157500	-
chr19	16170100	-
chr19	16443400	+
chr19	16513900	+
chr19	16544300	-
chr19	16632000	+
chr19	17049200	-
chr19	17187400	-
chr19	17207300	+
chr19	17239400	-
chr19	17309500	-
chr19	17377300	+
chr19	17442000	-
chr19	17483500	+
chr19	17527800	-
chr19	17722800	+
chr19	17844700	+
chr19	18058100	+
chr19	18069700	-
chr19	18125300	-
chr19	18145900	-
chr19	18165300	-
chr19	18175600	+
chr19	18312900	-
chr19	18514900	+
chr19	18544000	-
chr19	18561000	-
chr19	18609200	-
chr19	18655700	-
chr19	18672200	+
chr19	18891400	-
chr19	19005000	+
chr19	19036300	-
chr19	19110100	+
chr19	19164100	+
chr19	19292200	+
chr19	19358000	-
chr19	19487800	+
chr19	19590100	-
chr19	19599600	+
chr19	19614800	+
chr19	19634900	+
chr19	19748400	-
chr19	20996000	-
chr19	24008500	-
chr19	32976700	-
chr19	34395400	+
chr19	34789000	-
chr19	34849400	-
chr19	34897700	+
chr19	37528800	-
chr19	37764200	-
chr19	37875200	-
chr19	38154500	+
chr19	38264300	-
chr19	38359500	+
chr19	38704100	+
chr19	39437700	-
chr19	39548300	-
chr19	39611600	-
chr19	39955200	+
chr19	40338000	-
chr19	40728500	-
chr19	40811900	-
chr19	40826700	+
chr19	40899100	+
chr19	40924000	+
chr19	40941300	-
chr19	41085800	-
chr19	41119500	-
chr19	41142400	+
chr19	41178300	-
chr19	41297600	+
chr19	41323100	-
chr19	41397300	+
chr19	41869900	+
chr19	42261100	-
chr19	43089900	-
chr19	43447400	-
chr19	43519400	-
chr19	43557700	-
chr19	43801000	-
chr19	44014000	+
chr19	44081900	+
chr19	44113500	-
chr19	44517800	+
chr19	44524700	-
chr19	44573400	+
chr19	44663600	-
chr19	44715000	+
chr19	44722200	+
chr19	45006700	+
chr19	45015900	+
chr19	45028400	+
chr19	45169100	-
chr19	45194600	+
chr19	45253500	+
chr19	45288000	+
chr19	45389500	-
chr19	45482200	+
chr19	45546400	-
chr19	45641600	+
chr19	45663900	-
chr19	45774900	-
chr19	45803000	+
chr19	45811400	+
chr19	45915500	-
chr19	45976200	-
chr19	46507800	+
chr19	46561600	+
chr19	46574700	+
chr19	46595600	-
chr19	47056400	-
chr19	47124700	-
chr19	47154800	+
chr19	47272400	-
chr19	47328800	-
chr19	47480500	-
chr19	47498500	+
chr19	47593300	-
chr19	47619300	+
chr19	47724300	-
chr19	48659400	+
chr19	48699800	+
chr19	48722300	+
chr19	48729400	-
chr19	48792200	-
chr19	48815300	+
chr19	48865200	+
chr19	48950600	+
chr19	48976800	-
chr19	49291200	-
chr19	50150500	-
chr19	50274600	-
chr19	50601600	-
chr19	50618500	+
chr19	50638700	+
chr19	50664100	-
chr19	50673300	+
chr19	50703800	-
chr19	50836900	+
chr19	50887500	+
chr19	50912100	+
chr19	50925400	+
chr19	51057800	+
chr19	51097400	+
chr19	51190600	-
chr19	51211100	+
chr19	51798000	-
chr19	51833900	+
chr19	51913400	-
chr19	51982300	+
chr19	52045500	+
chr19	52056700	-
chr19	52243000	+
chr19	52326300	-
chr19	52352700	-
chr19	52469500	-
chr19	52709900	+
chr19	52974000	-
chr19	53365600	-
chr19	53399400	-
chr19	53528600	-
chr19	53586100	+
chr19	53640800	-
chr19	53813900	+
chr19	53832300	+
chr19	53909200	+
chr19	54095800	-
chr19	54150300	-
chr19	54160700	-
chr19	54188000	+
chr19	54213500	+
chr19	54280900	-
chr19	54314100	+
chr19	54531100	-
chr19	54729200	-
chr19	54775300	+
chr19	54860200	+
chr19	54872300	-
chr19	54962100	-
chr19	55013500	-
chr19	55072400	-
chr19	55220200	+
chr19	55579600	-
chr19	55999100	+
chr19	56303000	+
chr19	56561300	+
chr19	56766800	-
chr19	57385400	-
chr19	59298300	-
chr19	59310900	-
chr19	59355100	+
chr19	59386700	-
chr19	60320200	+
chr19	60483400	+
chr19	60542800	-
chr19	60610700	+
chr19	60664500	+
chr19	60680300	-
chr19	60783900	+
chr19	60803600	-
chr19	60809100	-
chr19	60838600	-
chr19	60846000	-
chr19	60879700	-
chr19	61324200	+
chr19	61344700	-
chr19	62483800	-
chr19	63482600	-
chr19	63508600	-
chr19	63530100	-
chr19	63583900	+
chr19	63590600	-
chr19	63611600	+
chr19	63679500	-
chr19	63702400	+
chr19	63722400	+
chr19	63758200	-
chr19	63765800	+
chr19	63776200	+
chr2	254900	-
chr2	666900	+
chr2	3363200	-
chr2	3502200	-
chr2	3583500	+
chr2	3601000	-
chr2	6922900	+
chr2	8894500	+
chr2	9480700	+
chr2	9532400	-
chr2	9613100	+
chr2	9688000	+
chr2	9811200	+
chr2	9901700	-
chr2	10360900	-
chr2	10388500	-
chr2	10505800	+
chr2	10746900	+
chr2	10869400	+
chr2	11213200	-
chr2	11401600	+
chr2	11804800	-
chr2	15618500	+
chr2	15649700	-
chr2	18604700	+
chr2	19964600	+
chr2	20114200	+
chr2	20414300	+
chr2	20714000	+
chr2	24002900	+
chr2	24152800	+
chr2	24160600	+
chr2	24436400	+
chr2	24567600	-
chr2	24996000	+
chr2	25390700	-
chr2	25399300	-
chr2	25749500	+
chr2	25898600	+
chr2	25954400	+
chr2	26058700	+
chr2	26111100	-
chr2	26261700	-
chr2	26321100	-
chr2	26422400	+
chr2	26840900	-
chr2	27109700	-
chr2	27147700	+
chr2	27210500	+
chr2	27288300	+
chr2	27399100	+
chr2	27432800	+
chr2	27447000	-
chr2	27456700	+
chr2	27485600	+
chr2	27505400	-
chr2	27659600	-
chr2	27705500	-
chr2	27740400	-
chr2	27848400	-
chr2	28467300	-
chr2	28828400	-
chr2	28946300	+
chr2	28972000	-
chr2	29192400	-
chr2	30223300	-
chr2	30524100	-
chr2	32088500	+
chr2	32142600	-
chr2	32244600	-
chr2	32356800	-
chr2	32435900	-
chr2	36678100	+
chr2	37047000	+
chr2	37165300	-
chr2	37277500	-
chr2	37312500	-
chr2	37404900	+
chr2	37751900	+
chr2	38457400	+
chr2	39200700	+
chr2	39517500	+
chr2	42441600	+
chr2	43676200	+
chr2	44442300	+
chr2	45691500	+
chr2	45733200	-
chr2	46697700	-
chr2	46996000	+
chr2	47022500	-
chr2	47256600	+
chr2	47484200	-
chr2	47864100	-
chr2	48396900	-
chr2	48522000	-
chr2	53849000	-
chr2	53867700	-
chr2	54051000	+
chr2	54654300	+
chr2	55129600	+
chr2	55774200	+
chr2	60837600	-
chr2	61098500	-
chr2	61551700	+
chr2	61616200	+
chr2	61968600	+
chr2	63669700	-
chr2	63922100	-
chr2	64224200	+
chr2	64605200	-
chr2	64733900	+
chr2	65070000	-
chr2	65137300	-
chr2	65210600	+
chr2	65308700	-
chr2	68142800	+
chr2	68238500	-
chr2	68856000	-
chr2	69467500	+
chr2	69910600	-
chr2	69974800	-
chr2	70166100	+
chr2	70328200	+
chr2	70338900	-
chr2	70382400	+
chr2	71075300	+
chr2	71211200	-
chr2	71413100	-
chr2	73193200	+
chr2	73294800	-
chr2	73315500	-
chr2	73349500	+
chr2	73364700	+
chr2	73466900	-
chr2	73817500	+
chr2	73860400	+
chr2	74228100	+
chr2	74258900	+
chr2	74279800	-
chr2	74471900	+
chr2	74495600	+
chr2	74502500	-
chr2	74539100	-
chr2	74545700	+
chr2	74553000	+
chr2	74563200	+
chr2	74610900	-
chr2	74635700	-
chr2	74735200	-
chr2	74915500	-
chr2	75039600	-
chr2	84539600	+
chr2	85050300	+
chr2	85390700	+
chr2	85435500	-
chr2	85490100	-
chr2	85641900	+
chr2	85659000	-
chr2	85665300	-
chr2	85676400	-
chr2	85683500	-
chr2	85692200	+
chr2	86186900	-
chr2	86275600	+
chr2	86643400	+
chr2	88135800	+
chr2	88707700	+
chr2	88772600	-
chr2	95151000	+
chr2	95195400	-
chr2	95432400	-
chr2	96237800	+
chr2	96294600	+
chr2	96334700	+
chr2	96557300	-
chr2	96667200	+
chr2	96769300	+
chr2	96790500	-
chr2	96802900	+
chr2	96887000	+
chr2	97629200	-
chr2	97646300	+
chr2	97978400	+
chr2	98428500	-
chr2	98447400	-
chr2	98591100	+
chr2	99163600	+
chr2	99319800	+
chr2	99472500	+
chr2	100545600	-
chr2	100985600	-
chr2	101236200	-
chr2	101682400	-
chr2	102719400	+
chr2	105312200	+
chr2	105320700	-
chr2	106079700	-
chr2	106120900	-
chr2	106176500	+
chr2	108432400	-
chr2	112529400	-
chr2	112656700	-
chr2	112956100	-
chr2	113016700	-
chr2	113058800	-
chr2	113120200	-
chr2	113631700	-
chr2	114230300	+
chr2	118289300	-
chr2	118487700	+
chr2	119841200	-
chr2	120153400	-
chr2	120727200	-
chr2	122004300	+
chr2	122123800	-
chr2	122210500	+
chr2	122229900	-
chr2	127131000	-
chr2	127767700	+
chr2	127861500	+
chr2	128000100	+
chr2	128175900	-
chr2	128285000	+
chr2	128331600	+
chr2	128501700	+
chr2	128565600	-
chr2	130656500	-
chr2	130816900	-
chr2	130829900	-
chr2	130845400	+
chr2	131515000	-
chr2	131579800	-
chr2	131966500	+
chr2	135393200	-
chr2	135527100	-
chr2	136216400	-
chr2	136350000	+
chr2	138976400	-
chr2	148494600	+
chr2	150151800	+
chr2	151853700	+
chr2	151975100	-
chr2	152392500	+
chr2	157001200	-
chr2	158441500	-
chr2	160277800	-
chr2	160469000	+
chr2	161725700	-
chr2	161803200	+
chr2	161873300	-
chr2	166518400	+
chr2	168811600	+
chr2	170149800	-
chr2	170364200	-
chr2	170392900	-
chr2	171494500	-
chr2	171998700	+
chr2	172253000	-
chr2	172487300	-
chr2	173001200	-
chr2	173035300	+
chr2	173129200	-
chr2	174821300	+
chr2	174968400	+
chr2	175206800	+
chr2	175255200	+
chr2	175740800	+
chr2	175754200	+
chr2	177966000	-
chr2	178686000	-
chr2	179023700	+
chr2	179054500	-
chr2	179096300	-
chr2	180579800	+
chr2	183697600	-
chr2	187059600	-
chr2	190014700	-
chr2	190234600	-
chr2	190357100	+
chr2	190892600	+
chr2	190981700	-
chr2	191723300	+
chr2	198007600	+
chr2	198026700	-
chr2	198089100	-
chr2	198279100	-
chr2	200484700	-
chr2	200528900	-
chr2	201385100	-
chr2	201536300	+
chr2	201644900	-
chr2	201757100	-
chr2	201806500	-
chr2	201834600	-
chr2	202024200	+
chr2	202810900	+
chr2	202839600	-
chr2	203812500	-
chr2	206732500	-
chr2	207338900	-
chr2	207716500	-
chr2	208103500	-
chr2	208284900	-
chr2	208342500	+
chr2	208839300	-
chr2	210575800	-
chr2	215885200	-
chr2	216586600	-
chr2	216682500	-
chr2	217072100	-
chr2	218790700	-
chr2	218843300	-
chr2	218864400	+
chr2	218979200	+
chr2	219141600	-
chr2	219232000	+
chr2	219244600	+
chr2	219454100	-
chr2	219470000	+
chr2	219733600	-
chr2	219751600	-
chr2	219780100	-
chr2	219791800	-
chr2	219802200	+
chr2	219852900	-
chr2	219960100	+
chr2	220115900	+
chr2	220170900	-
chr2	223228800	+
chr2	223434300	-
chr2	224517700	+
chr2	225157900	+
chr2	225518000	+
chr2	225614600	+
chr2	227371800	+
chr2	227409000	+
chr2	227898600	-
chr2	230492900	+
chr2	230900700	-
chr2	231286600	-
chr2	231630200	-
chr2	232037300	+
chr2	232279600	+
chr2	232359700	-
chr2	232534800	-
chr2	233123800	-
chr2	233270700	-
chr2	233825600	-
chr2	233928500	-
chr2	237659400	-
chr2	238247200	-
chr2	238540900	-
chr2	238634400	-
chr2	238776200	+
chr2	238861800	+
chr2	238894200	-
chr2	239000600	-
chr2	239872400	+
chr2	239885000	+
chr2	239967800	+
chr2	239987600	+
chr2	240613000	+
chr2	240724000	+
chr2	241690000	+
chr2	241738300	-
chr2	241860200	+
chr2	241904100	-
chr2	242205700	-
chr2	242275200	-
chr2	242472500	-
chr2	242622200	+
chr20	227100	-
chr20	254000	-
chr20	276500	-
chr20	337400	-
chr20	390700	+
chr20	471800	+
chr20	762800	-
chr20	775500	-
chr20	1047700	-
chr20	1195300	-
chr20	1254400	-
chr20	1321300	+
chr20	1395000	+
chr20	2031400	-
chr20	2398800	+
chr20	2581500	-
chr20	2592600	+
chr20	2680700	-
chr20	2769100	+
chr20	2802300	-
chr20	2974900	-
chr20	3088200	+
chr20	3132700	+
chr20	3138400	-
chr20	3400200	-
chr20	3696500	+
chr20	3715100	+
chr20	3775700	-
chr20	3818200	-
chr20	3943900	+
chr20	4615600	-
chr20	4751200	+
chr20	4929600	+
chr20	5048100	+
chr20	5055900	-
chr20	5538600	+
chr20	5935100	-
chr20	7947600	+
chr20	10363000	+
chr20	13714000	-
chr20	16659100	-
chr20	17499200	-
chr20	17610400	+
chr20	18066900	-
chr20	18396000	-
chr20	18436700	-
chr20	19946300	-
chr20	19980900	+
chr20	20640700	+
chr20	21232300	-
chr20	23279700	-
chr20	23291000	-
chr20	23349500	+
chr20	24920900	+
chr20	24985700	+
chr20	25124700	-
chr20	25177000	-
chr20	25319000	+
chr20	25552200	+
chr20	25624900	+
chr20	29566400	-
chr20	29726800	+
chr20	30003100	+
chr20	30161700	-
chr20	30241400	+
chr20	30329400	-
chr20	30410300	-
chr20	30871800	-
chr20	31452700	+
chr20	31495100	+
chr20	31541900	-
chr20	31718900	-
chr20	31771300	+
chr20	32163400	+
chr20	32415200	-
chr20	32567600	-
chr20	32728300	+
chr20	32756000	-
chr20	32761200	-
chr20	32876600	+
chr20	32927800	+
chr20	33143900	+
chr20	33198400	+
chr20	33328500	+
chr20	33335600	+
chr20	33506500	-
chr20	33541400	+
chr20	33593400	-
chr20	33670200	+
chr20	33715600	+
chr20	33750100	+
chr20	34005300	+
chr20	34288000	-
chr20	34523500	-
chr20	34637000	-
chr20	34706700	+
chr20	34807600	+
chr20	35157200	+
chr20	35241100	-
chr20	35351500	-
chr20	35756000	-
chr20	36095600	-
chr20	36496700	+
chr20	36535000	-
chr20	36810500	-
chr20	36988900	-
chr20	37024600	-
chr20	39091500	-
chr20	42272700	+
chr20	42372900	+
chr20	42583400	+
chr20	42713400	+
chr20	42948100	-
chr20	43021900	+
chr20	43425500	-
chr20	43435700	+
chr20	43468700	-
chr20	43478300	-
chr20	43854200	-
chr20	43896200	-
chr20	43919000	+
chr20	43953500	-
chr20	43997000	-
chr20	44033600	+
chr20	44151400	+
chr20	44750700	+
chr20	46971500	+
chr20	47096700	-
chr20	47237400	+
chr20	47269600	-
chr20	47763300	+
chr20	47863300	-
chr20	47965000	+
chr20	47986800	-
chr20	48162600	+
chr20	48203200	+
chr20	48781600	-
chr20	48980500	+
chr20	49008800	-
chr20	49591700	+
chr20	52258500	-
chr20	54367600	-
chr20	54401000	-
chr20	54477500	-
chr20	55359900	-
chr20	55628300	+
chr20	55718400	+
chr20	56318500	-
chr20	56398000	-
chr20	56669500	-
chr20	56990900	-
chr20	57014400	+
chr20	57040600	+
chr20	57050700	+
chr20	57168300	+
chr20	57948600	+
chr20	60191600	-
chr20	60247500	-
chr20	60395600	-
chr20	60753300	+
chr20	60896200	+
chr20	61027300	+
chr20	61204000	+
chr20	61375300	-
chr20	61809900	-
chr20	61966300	+
chr20	62083000	-
chr20	62179900	+
chr20	62357800	-
chr21	14677100	+
chr21	16024700	-
chr21	17906200	+
chr21	18113200	+
chr21	25901500	+
chr21	26028700	+
chr21	29286700	+
chr21	29319200	-
chr21	29367500	+
chr21	29593500	-
chr21	31954300	-
chr21	32026000	+
chr21	32687000	+
chr21	32706700	-
chr21	32906200	+
chr21	33021800	+
chr21	33065700	+
chr21	33560500	+
chr21	33619800	-
chr21	33698200	-
chr21	33773700	+
chr21	33936100	+
chr21	34209700	+
chr21	34368400	-
chr21	34669900	-
chr21	36354300	+
chr21	36364700	-
chr21	36451100	-
chr21	36615000	-
chr21	37367500	-
chr21	37552900	+
chr21	37662300	-
chr21	39100000	-
chr21	39476800	+
chr21	39607300	+
chr21	39642600	+
chr21	39674600	-
chr21	39681500	+
chr21	41656400	-
chr21	41720500	-
chr21	42171900	+
chr21	42513100	-
chr21	42807500	-
chr21	42908800	+
chr21	43172100	+
chr21	43186700	-
chr21	43268100	-
chr21	43401200	+
chr21	43470700	-
chr21	43904700	-
chr21	43964000	-
chr21	44020200	+
chr21	44111000	-
chr21	44583400	+
chr21	44599000	-
chr21	45045500	+
chr21	45117400	+
chr21	45164600	+
chr21	45175800	+
chr21	45723200	-
chr21	45835400	-
chr21	46473000	+
chr21	46530200	+
chr21	46567600	+
chr21	46702700	+
chr21	46804700	-
chr21	46881000	-
chr22	15947200	-
chr22	16019700	+
chr22	16491300	+
chr22	16501800	-
chr22	16940900	-
chr22	17489400	+
chr22	17539500	-
chr22	17545800	+
chr22	17799000	+
chr22	17815300	-
chr22	17846400	+
chr22	18222000	+
chr22	18309600	-
chr22	18388900	-
chr22	18574500	-
chr22	19078600	-
chr22	19179500	+
chr22	19192500	-
chr22	19542700	+
chr22	19602000	-
chr22	19666800	-
chr22	19686000	+
chr22	20326800	-
chr22	20336300	+
chr22	20350700	-
chr22	20551400	+
chr22	21742800	-
chr22	21854600	-
chr22	22369000	-
chr22	22388400	+
chr22	22459600	-
chr22	22510900	+
chr22	22566700	-
chr22	22703300	-
chr22	22738100	-
chr22	22883400	-
chr22	22997200	-
chr22	23154500	-
chr22	23280600	+
chr22	24130500	+
chr22	24174300	-
chr22	25155300	-
chr22	25210200	-
chr22	25237900	+
chr22	25315600	+
chr22	25399700	-
chr22	26644900	+
chr22	27468400	-
chr22	27499200	-
chr22	27525900	+
chr22	28114000	+
chr22	28279800	-
chr22	28330000	-
chr22	28493400	-
chr22	28564000	+
chr22	28609700	-
chr22	28992200	+
chr22	29015300	+
chr22	29082500	+
chr22	29123500	-
chr22	29152000	-
chr22	29317500	+
chr22	29361800	-
chr22	29393500	+
chr22	29810600	+
chr22	29833200	+
chr22	29938600	-
chr22	30071400	+
chr22	30125700	-
chr22	30215700	+
chr22	30223100	-
chr22	30356100	+
chr22	30387800	+
chr22	30670800	-
chr22	31137700	+
chr22	31200700	+
chr22	33983900	-
chr22	34026300	-
chr22	34375300	-
chr22	34964500	+
chr22	35207400	+
chr22	35502000	+
chr22	35875100	+
chr22	36211500	-
chr22	36335500	-
chr22	36385200	-
chr22	36402100	+
chr22	36412500	-
chr22	36472400	-
chr22	36575500	-
chr22	36679900	-
chr22	36907200	+
chr22	36928600	-
chr22	36998400	+
chr22	37382900	-
chr22	37408100	-
chr22	37871000	+
chr22	37878400	+
chr22	37968100	+
chr22	38126100	-
chr22	38228700	-
chr22	38246300	-
chr22	38771100	-
chr22	38904000	+
chr22	39073100	-
chr22	39097000	-
chr22	39188600	+
chr22	39362500	+
chr22	39544900	+
chr22	39582400	+
chr22	39677500	-
chr22	39820200	-
chr22	39931300	-
chr22	40011900	+
chr22	40028100	-
chr22	40107900	-
chr22	40140000	-
chr22	40194700	+
chr22	40270000	+
chr22	40315800	+
chr22	40347100	-
chr22	40408300	+
chr22	40414200	+
chr22	40507700	-
chr22	40526700	-
chr22	40559200	-
chr22	40816600	+
chr22	41340600	+
chr22	41375100	+
chr22	41582600	+
chr22	41740700	+
chr22	41815100	+
chr22	41837100	-
chr22	41877800	-
chr22	41912700	+
chr22	42682800	-
chr22	42909400	-
chr22	43938300	+
chr22	44015200	+
chr22	44084800	-
chr22	44446800	-
chr22	44788100	+
chr22	44828700	-
chr22	44925100	-
chr22	45024700	+
chr22	45110600	-
chr22	45352000	-
chr22	45388400	+
chr22	45448900	+
chr22	45537600	-
chr22	48436700	+
chr22	48504200	-
chr22	48697700	-
chr22	49095400	-
chr22	49315800	+
chr22	49334700	-
chr22	49367800	+
chr22	49413300	+
chr3	3143900	-
chr3	3196000	+
chr3	4320400	-
chr3	4521200	-
chr3	4994100	+
chr3	5139400	-
chr3	5204600	+
chr3	9380100	-
chr3	9748800	-
chr3	9767300	-
chr3	9809000	+
chr3	9860400	+
chr3	9908000	-
chr3	9950600	-
chr3	9968700	+
chr3	10003400	+
chr3	10042800	+
chr3	10132800	-
chr3	10159400	-
chr3	10265800	-
chr3	10337300	+
chr3	11289200	-
chr3	11659200	+
chr3	11862800	+
chr3	12574000	-
chr3	12680000	+
chr3	12857700	+
chr3	13031700	+
chr3	13496800	-
chr3	14141600	-
chr3	14194800	+
chr3	14419500	-
chr3	14668600	-
chr3	14964700	-
chr3	15081100	+
chr3	15115400	+
chr3	15223200	-
chr3	15348700	+
chr3	15444300	-
chr3	15617800	+
chr3	15876100	+
chr3	16281100	+
chr3	16529200	+
chr3	19964000	-
chr3	20057700	-
chr3	23827700	-
chr3	23933900	-
chr3	23962500	-
chr3	25680600	+
chr3	25799500	+
chr3	27385100	-
chr3	27500300	+
chr3	28258300	-
chr3	28365100	+
chr3	32123400	-
chr3	32409100	-
chr3	32518800	+
chr3	32586600	+
chr3	32702200	-
chr3	33113000	+
chr3	33130600	+
chr3	33455100	+
chr3	33734300	+
chr3	33815300	-
chr3	36924400	+
chr3	36961000	+
chr3	37192900	-
chr3	37260000	-
chr3	38040800	+
chr3	38182200	-
chr3	38470900	-
chr3	38513300	-
chr3	39068900	-
chr3	39123500	+
chr3	39423900	-
chr3	40474100	-
chr3	41216900	-
chr3	42030700	-
chr3	42519100	-
chr3	42598800	-
chr3	42607500	-
chr3	42617500	-
chr3	42820400	+
chr3	43122000	+
chr3	44493600	+
chr3	44526700	+
chr3	44641900	-
chr3	44878700	-
chr3	44992200	+
chr3	45610800	-
chr3	45681600	-
chr3	45705900	-
chr3	46996900	-
chr3	47180200	+
chr3	47299500	-
chr3	47397800	-
chr3	47492000	+
chr3	47529500	+
chr3	47797900	+
chr3	47819800	-
chr3	47841900	-
chr3	48104800	+
chr3	48317300	+
chr3	48457100	-
chr3	48463400	-
chr3	48495600	+
chr3	48516100	+
chr3	48621500	+
chr3	48647600	+
chr3	48698000	+
chr3	48728800	+
chr3	48859600	+
chr3	48910800	+
chr3	49003100	-
chr3	49020000	-
chr3	49034200	-
chr3	49041700	+
chr3	49116800	+
chr3	49132900	+
chr3	49352300	+
chr3	49370200	+
chr3	49483000	-
chr3	49702300	-
chr3	49798700	+
chr3	49815900	-
chr3	49825900	+
chr3	49952900	-
chr3	50102300	-
chr3	50240200	-
chr3	50304400	+
chr3	50311600	+
chr3	50333400	+
chr3	50340300	+
chr3	50352900	+
chr3	50371500	+
chr3	50377800	-
chr3	50630000	-
chr3	51397700	-
chr3	51508700	+
chr3	51548000	-
chr3	51680300	+
chr3	51950700	+
chr3	51976400	+
chr3	51984100	-
chr3	51992000	+
chr3	52004700	+
chr3	52207500	-
chr3	52255500	-
chr3	52419600	-
chr3	52465100	-
chr3	52546000	-
chr3	52695100	-
chr3	52715100	-
chr3	52906200	+
chr3	53171100	-
chr3	53264200	+
chr3	53356100	+
chr3	53890700	+
chr3	53900500	+
chr3	56566700	-
chr3	56691600	+
chr3	56764400	-
chr3	56810600	+
chr3	57237300	-
chr3	57517500	-
chr3	57557900	+
chr3	57653400	+
chr3	57717500	-
chr3	57970000	-
chr3	58267500	-
chr3	58294100	-
chr3	58394100	+
chr3	58453200	-
chr3	63824500	+
chr3	63873300	-
chr3	63983800	+
chr3	67787600	+
chr3	69183500	+
chr3	69331400	+
chr3	71195100	+
chr3	71359100	+
chr3	71521900	-
chr3	71856900	+
chr3	72979900	+
chr3	73129100	-
chr3	88191100	-
chr3	88282100	-
chr3	99173700	+
chr3	99723900	+
chr3	99794700	+
chr3	101019600	-
chr3	101462600	-
chr3	101602300	+
chr3	101911500	-
chr3	102714300	+
chr3	102763800	-
chr3	102775800	-
chr3	102877700	+
chr3	102887700	+
chr3	102981100	-
chr3	112750400	-
chr3	112876900	-
chr3	113181200	-
chr3	113762800	+
chr3	114193400	-
chr3	114221000	+
chr3	114734700	-
chr3	114897900	+
chr3	114948000	+
chr3	115258400	-
chr3	120441900	+
chr3	120524900	-
chr3	120664900	+
chr3	120671000	-
chr3	120878500	+
chr3	121550500	+
chr3	121798300	-
chr3	121944400	-
chr3	122861500	+
chr3	122951000	+
chr3	123036300	+
chr3	123585600	-
chr3	123618000	-
chr3	123715900	+
chr3	123766100	-
chr3	123882500	-
chr3	123996200	-
chr3	125932300	-
chr3	126256800	+
chr3	126559100	+
chr3	126576300	+
chr3	126721200	+
chr3	127285200	+
chr3	128184800	-
chr3	128791800	+
chr3	128874600	-
chr3	129254300	-
chr3	129277400	-
chr3	129324800	+
chr3	129819600	-
chr3	129851700	+
chr3	129928100	-
chr3	130081300	-
chr3	130363000	-
chr3	130384700	+
chr3	130451500	-
chr3	130481000	-
chr3	130600900	+
chr3	130641600	+
chr3	131094700	+
chr3	131948200	+
chr3	132228000	+
chr3	132583500	-
chr3	133619500	-
chr3	133923600	+
chr3	134863100	+
chr3	135007500	-
chr3	135451900	+
chr3	135687600	-
chr3	138064100	-
chr3	139376000	+
chr3	139389300	-
chr3	140036100	+
chr3	140545600	-
chr3	140590700	+
chr3	142433700	-
chr3	142688700	-
chr3	142940500	-
chr3	143649300	+
chr3	143780000	+
chr3	144203300	-
chr3	144321900	-
chr3	147744400	+
chr3	150192400	-
chr3	150330400	-
chr3	151013800	-
chr3	151746700	+
chr3	151804300	-
chr3	157054500	+
chr3	157755100	+
chr3	157875500	-
chr3	158360200	+
chr3	158376000	-
chr3	159845400	-
chr3	160002600	-
chr3	161765800	+
chr3	161956300	-
chr3	162422500	-
chr3	168935100	+
chr3	170973700	-
chr3	171167600	-
chr3	171381700	+
chr3	172070200	+
chr3	172448900	-
chr3	178396000	+
chr3	180272000	+
chr3	180349100	-
chr3	180524100	-
chr3	180548700	-
chr3	180763900	-
chr3	180805400	-
chr3	182113700	-
chr3	182189900	+
chr3	184180600	+
chr3	184299500	+
chr3	184628700	+
chr3	184899000	-
chr3	185084900	+
chr3	185217900	+
chr3	185335700	-
chr3	185356400	-
chr3	185375700	-
chr3	185386900	-
chr3	185449600	+
chr3	185499900	-
chr3	185536900	-
chr3	185563800	-
chr3	185911900	+
chr3	186013100	-
chr3	186698800	+
chr3	186787100	-
chr3	187137800	+
chr3	187771400	-
chr3	188132100	-
chr3	189355000	-
chr3	194794000	-
chr3	195689000	-
chr3	195874000	+
chr3	195888600	+
chr3	196462200	-
chr3	196472800	+
chr3	197106800	+
chr3	197118600	-
chr3	197292800	+
chr3	197498700	+
chr3	197643400	+
chr3	197713500	+
chr3	197780300	-
chr3	197851500	-
chr3	197951700	-
chr3	198079500	-
chr3	198153600	-
chr3	198509100	+
chr3	198766200	+
chr3	198947700	+
chr3	198961600	-
chr3	199003100	-
chr3	199171300	+
chr4	483500	-
chr4	658200	+
chr4	765200	+
chr4	863000	+
chr4	915800	+
chr4	993700	-
chr4	1151200	-
chr4	1185100	+
chr4	1274000	-
chr4	1330400	+
chr4	1655700	-
chr4	1694800	-
chr4	1827800	-
chr4	1844600	-
chr4	1980300	+
chr4	2213100	+
chr4	2384600	+
chr4	2440900	-
chr4	2508100	-
chr4	2727200	+
chr4	2784400	-
chr4	2809500	+
chr4	2815600	-
chr4	2934600	+
chr4	3046600	-
chr4	3264300	+
chr4	3273900	-
chr4	3503300	+
chr4	4300500	+
chr4	4342900	-
chr4	4594400	+
chr4	4627700	+
chr4	6627800	-
chr4	6693700	-
chr4	6727800	-
chr4	6761800	+
chr4	6769400	-
chr4	6836100	-
chr4	6962700	-
chr4	7040000	-
chr4	7120400	+
chr4	8023400	-
chr4	8252300	-
chr4	8480800	+
chr4	8494000	-
chr4	13094900	+
chr4	13238000	+
chr4	15265600	+
chr4	15292900	-
chr4	15389600	-
chr4	15835400	+
chr4	17188400	-
chr4	17421300	+
chr4	20311400	-
chr4	24194300	+
chr4	24845900	-
chr4	24988400	-
chr4	26468700	-
chr4	37364100	+
chr4	37504900	-
chr4	37569700	-
chr4	38188500	-
chr4	38723200	-
chr4	39043900	+
chr4	39205300	+
chr4	39376600	-
chr4	39655100	+
chr4	39735200	-
chr4	41632300	-
chr4	41687500	-
chr4	42353500	+
chr4	48038700	-
chr4	48476600	+
chr4	48527900	-
chr4	48603100	+
chr4	53273700	-
chr4	53939100	-
chr4	54625000	+
chr4	55957300	-
chr4	56415100	-
chr4	56509700	-
chr4	56996600	+
chr4	57028200	+
chr4	57471000	-
chr4	57537400	+
chr4	68249100	+
chr4	71773400	-
chr4	71790200	-
chr4	71923700	+
chr4	72078600	-
chr4	74343200	+
chr4	76658500	+
chr4	76816900	+
chr4	76868900	-
chr4	77080200	+
chr4	77130400	+
chr4	77288300	+
chr4	77353800	+
chr4	78090000	-
chr4	78214300	+
chr4	81325100	-
chr4	81342700	+
chr4	84030900	+
chr4	84175700	-
chr4	84625000	+
chr4	89148200	-
chr4	89423800	+
chr4	89663400	+
chr4	89732900	-
chr4	89837600	+
chr4	90251200	+
chr4	90429500	-
chr4	99798200	+
chr4	100069000	+
chr4	100136300	-
chr4	101034300	+
chr4	102487200	+
chr4	103485100	+
chr4	103900700	+
chr4	106614000	+
chr4	106849100	+
chr4	107457200	-
chr4	108860800	+
chr4	109760900	-
chr4	109791400	-
chr4	110574900	-
chr4	110701200	-
chr4	110843300	+
chr4	110870200	+
chr4	110956400	-
chr4	113286500	-
chr4	113425800	+
chr4	113778000	-
chr4	119420000	-
chr4	119826500	-
chr4	120440600	+
chr4	123292900	-
chr4	124063400	-
chr4	129106300	-
chr4	129202200	-
chr4	129428300	+
chr4	129951000	-
chr4	130233600	+
chr4	140223900	+
chr4	140594800	-
chr4	140697000	+
chr4	141293800	+
chr4	141514400	-
chr4	142362500	-
chr4	144654600	-
chr4	146239300	-
chr4	146319100	+
chr4	147316900	-
chr4	148758300	-
chr4	148824300	+
chr4	148873000	-
chr4	152240500	-
chr4	153678000	-
chr4	153920400	-
chr4	154611900	-
chr4	154629800	+
chr4	154662600	-
chr4	155690500	+
chr4	159863500	+
chr4	159909800	-
chr4	160244500	-
chr4	164306800	+
chr4	166348400	-
chr4	166468600	-
chr4	169637800	+
chr4	170778400	-
chr4	170915100	+
chr4	177478500	-
chr4	184603100	-
chr4	185805800	+
chr4	185983700	+
chr4	186362000	+
chr4	186368400	-
chr4	186554400	-
chr4	186583900	+
chr5	272000	-
chr5	496400	-
chr5	525900	+
chr5	665200	+
chr5	945600	+
chr5	1397900	+
chr5	1439200	+
chr5	1576500	+
chr5	1852500	+
chr5	5476200	-
chr5	6431300	+
chr5	6686100	+
chr5	6769200	-
chr5	7922600	-
chr5	10303700	-
chr5	10407300	-
chr5	10617900	-
chr5	10813700	+
chr5	14635700	-
chr5	14647800	+
chr5	14718000	-
chr5	14923300	+
chr5	16518500	+
chr5	31567700	+
chr5	32348300	+
chr5	32479700	+
chr5	32621800	-
chr5	33477000	-
chr5	34951600	-
chr5	34965800	-
chr5	36187900	-
chr5	36277900	-
chr5	37406500	+
chr5	39109800	+
chr5	40790900	+
chr5	40833700	+
chr5	40870600	+
chr5	41905800	+
chr5	41940600	-
chr5	42987400	-
chr5	43079000	-
chr5	43157600	-
chr5	43348900	+
chr5	43592400	+
chr5	43639300	-
chr5	44845200	-
chr5	52131900	-
chr5	52440800	+
chr5	53852100	+
chr5	54638800	+
chr5	56283100	+
chr5	58370300	+
chr5	60277000	-
chr5	61638500	-
chr5	61735000	+
chr5	61744700	-
chr5	64100100	+
chr5	64956000	+
chr5	66336300	-
chr5	67547800	-
chr5	68426200	-
chr5	68549700	-
chr5	68700700	+
chr5	70787600	-
chr5	70919300	-
chr5	71639900	-
chr5	71651400	+
chr5	72180500	-
chr5	72830500	-
chr5	72896900	+
chr5	74017400	-
chr5	74099100	-
chr5	74668800	-
chr5	74842800	+
chr5	75048100	+
chr5	75736200	-
chr5	77107300	+
chr5	77692100	+
chr5	78944600	-
chr5	79986200	+
chr5	80292000	+
chr5	80633400	-
chr5	81082400	+
chr5	81609500	+
chr5	82408600	+
chr5	85949700	-
chr5	86599700	-
chr5	86744100	+
chr5	87600700	-
chr5	89805500	+
chr5	89860700	+
chr5	93980300	-
chr5	94916200	+
chr5	94982500	-
chr5	95322700	+
chr5	96024100	-
chr5	96064600	-
chr5	96544600	+
chr5	100254000	-
chr5	102484200	-
chr5	107745500	+
chr5	110102800	-
chr5	110456200	-
chr5	110876000	+
chr5	111524800	-
chr5	112071600	-
chr5	112101400	-
chr5	112225500	-
chr5	112285700	+
chr5	112340800	-
chr5	112877600	-
chr5	114625800	+
chr5	114907400	+
chr5	114989300	+
chr5	115205300	+
chr5	118653900	-
chr5	121325800	-
chr5	122139400	-
chr5	122876100	-
chr5	125964900	-
chr5	126141500	-
chr5	126393900	+
chr5	126881700	-
chr5	127447600	-
chr5	128458800	-
chr5	130528100	+
chr5	130534600	-
chr5	130998400	+
chr5	131160300	+
chr5	131733700	-
chr5	131920800	-
chr5	132100800	+
chr5	132230300	-
chr5	132326600	+
chr5	132389700	+
chr5	132415700	-
chr5	133331800	+
chr5	133540000	+
chr5	133589300	+
chr5	133735300	-
chr5	133775000	+
chr5	133996200	+
chr5	134012700	-
chr5	134102300	-
chr5	134122700	-
chr5	134210000	-
chr5	134238400	-
chr5	137098400	+
chr5	137117400	+
chr5	137396200	+
chr5	137542400	-
chr5	137702200	-
chr5	137717200	-
chr5	137906600	+
chr5	137938800	+
chr5	138637400	+
chr5	138658000	-
chr5	138705800	-
chr5	138756500	+
chr5	138767300	+
chr5	138841700	+
chr5	138921700	-
chr5	139473900	+
chr5	139761500	-
chr5	139907800	-
chr5	139916600	+
chr5	139924700	-
chr5	139999500	-
chr5	140007200	+
chr5	140025000	-
chr5	140051100	+
chr5	140872700	-
chr5	140976600	+
chr5	140996400	+
chr5	141010700	+
chr5	141283700	-
chr5	141469300	-
chr5	142130600	-
chr5	143529900	+
chr5	145541800	+
chr5	145563600	-
chr5	145807500	-
chr5	147744000	-
chr5	148705900	-
chr5	148718200	-
chr5	149320500	-
chr5	149360800	-
chr5	149717800	-
chr5	149772100	+
chr5	149809000	+
chr5	150060200	+
chr5	150118300	+
chr5	150446000	-
chr5	150582900	+
chr5	151118200	+
chr5	153398400	+
chr5	153550300	-
chr5	153805700	-
chr5	154297500	+
chr5	156502200	+
chr5	156935000	+
chr5	157218200	+
chr5	159368900	-
chr5	162797500	-
chr5	167846300	-
chr5	167938800	+
chr5	170747800	-
chr5	171365900	+
chr5	172194600	-
chr5	172344000	-
chr5	172975500	+
chr5	173248300	-
chr5	174838700	-
chr5	175721100	+
chr5	175748200	+
chr5	175808000	-
chr5	175902200	+
chr5	176007300	-
chr5	176365700	+
chr5	176494000	-
chr5	176663100	+
chr5	176710900	+
chr5	176717900	-
chr5	176763600	-
chr5	176805100	+
chr5	176814700	+
chr5	176857000	+
chr5	176876500	+
chr5	176913600	+
chr5	176959900	-
chr5	177564700	-
chr5	177591900	+
chr5	177985900	+
chr5	178910800	-
chr5	178983500	+
chr5	179038400	-
chr5	179058700	-
chr5	179093000	-
chr5	179155300	+
chr5	179218100	+
chr5	179431000	+
chr5	179651600	+
chr5	179854300	-
chr5	180166900	-
chr5	180190500	+
chr5	180220300	+
chr5	180566200	+
chr5	180576800	-
chr5	180602900	+
chr5	180620100	+
chr6	337500	-
chr6	391700	+
chr6	637700	+
chr6	2190500	+
chr6	2712200	-
chr6	2786800	+
chr6	2945200	-
chr6	2968700	+
chr6	3014300	-
chr6	3063600	+
chr6	3794800	-
chr6	3966900	-
chr6	4721300	-
chr6	7258100	+
chr6	7335300	-
chr6	7535500	-
chr6	8047400	+
chr6	8380300	+
chr6	10803400	-
chr6	10856100	-
chr6	11202500	-
chr6	11341300	-
chr6	11490200	+
chr6	12117500	-
chr6	13683000	-
chr6	13723700	-
chr6	13818000	+
chr6	13922200	+
chr6	14226200	-
chr6	15353900	-
chr6	15770600	+
chr6	16428100	-
chr6	16870500	-
chr6	17709100	-
chr6	18372200	+
chr6	20314700	+
chr6	24511600	-
chr6	24774600	+
chr6	24883600	-
chr6	25135000	-
chr6	26135300	-
chr6	26567000	-
chr6	26646300	-
chr6	26705100	-
chr6	27548500	+
chr6	28217200	-
chr6	28431900	+
chr6	28998900	+
chr6	29704000	-
chr6	29824400	+
chr6	30136800	+
chr6	30143100	-
chr6	30178400	+
chr6	30402400	+
chr6	30632300	+
chr6	30647300	-
chr6	30702800	+
chr6	30723000	+
chr6	30748500	+
chr6	30818400	+
chr6	30905300	+
chr6	30961700	+
chr6	30984100	-
chr6	31273900	-
chr6	31479300	+
chr6	31617000	+
chr6	31652100	-
chr6	31727900	+
chr6	31736000	+
chr6	31741000	+
chr6	31778400	+
chr6	31811100	+
chr6	31882200	+
chr6	31911400	-
chr6	31938500	+
chr6	31973900	-
chr6	32034300	+
chr6	32047700	+
chr6	32204400	+
chr6	32230400	-
chr6	32254200	-
chr6	32970700	-
chr6	33325100	-
chr6	33347400	+
chr6	33364700	+
chr6	33374600	+
chr6	33389200	+
chr6	33398500	+
chr6	33486800	-
chr6	33493600	+
chr6	33530600	-
chr6	33646700	+
chr6	33654600	+
chr6	33697300	-
chr6	33787100	+
chr6	34313000	-
chr6	34324600	+
chr6	34467900	+
chr6	34501300	+
chr6	34772000	+
chr6	34833300	-
chr6	34964000	+
chr6	35335700	-
chr6	35528500	-
chr6	35544700	-
chr6	35763700	+
chr6	35996000	+
chr6	36104200	-
chr6	36206900	-
chr6	36519500	-
chr6	36755300	+
chr6	36961700	-
chr6	37030800	-
chr6	37061000	+
chr6	37333900	-
chr6	37509400	-
chr6	37593300	-
chr6	37895200	-
chr6	38715800	-
chr6	41636700	-
chr6	41809700	+
chr6	41862700	+
chr6	41997400	-
chr6	42442900	+
chr6	42640300	-
chr6	42821800	+
chr6	42966500	+
chr6	43054500	+
chr6	43135000	+
chr6	43248000	-
chr6	43258300	-
chr6	43444800	+
chr6	43503800	-
chr6	43530600	+
chr6	43553600	-
chr6	43592500	+
chr6	43704800	+
chr6	43712000	-
chr6	43763100	+
chr6	44149600	-
chr6	44203000	+
chr6	44299000	+
chr6	44322500	-
chr6	44332600	+
chr6	44339900	+
chr6	44388600	+
chr6	44463800	-
chr6	52257200	+
chr6	52335600	-
chr6	52549600	+
chr6	52644100	-
chr6	53038500	-
chr6	53308700	-
chr6	64340800	-
chr6	64404100	-
chr6	70563100	+
chr6	72055500	-
chr6	74228400	-
chr6	76009700	+
chr6	76050900	+
chr6	76369000	-
chr6	83013800	+
chr6	83959500	+
chr6	84626300	-
chr6	86359900	+
chr6	86408300	+
chr6	87922400	-
chr6	88356000	+
chr6	88468000	+
chr6	89729800	+
chr6	90118400	+
chr6	90585800	+
chr6	90596500	-
chr6	91051700	-
chr6	91353100	+
chr6	97452000	+
chr6	99502100	+
chr6	99979500	+
chr6	100122600	+
chr6	101435500	+
chr6	105956600	+
chr6	106879900	+
chr6	107074200	-
chr6	107184400	-
chr6	107456300	-
chr6	107887300	+
chr6	108385600	+
chr6	108502300	+
chr6	108986100	+
chr6	109809900	+
chr6	109869000	-
chr6	109883100	+
chr6	109910100	+
chr6	111242800	+
chr6	111410100	-
chr6	112033600	+
chr6	114398100	+
chr6	116529100	-
chr6	116681400	+
chr6	116707300	+
chr6	116999600	-
chr6	117109200	-
chr6	118029800	+
chr6	119093300	-
chr6	119137300	+
chr6	119441700	-
chr6	122762900	-
chr6	122834100	+
chr6	126153600	-
chr6	126319900	-
chr6	127630200	-
chr6	127705800	+
chr6	131990800	+
chr6	132875400	+
chr6	134316100	-
chr6	135417400	+
chr6	135545900	-
chr6	136613500	-
chr6	137185800	-
chr6	137581600	+
chr6	138767200	-
chr6	139136600	-
chr6	139350400	+
chr6	139391900	-
chr6	142510600	-
chr6	143874500	-
chr6	143901700	-
chr6	144206300	-
chr6	144458100	+
chr6	144513900	-
chr6	144707400	-
chr6	146177800	-
chr6	149908400	+
chr6	149929400	-
chr6	150011000	+
chr6	150080400	+
chr6	150112800	-
chr6	150368200	-
chr6	151229200	-
chr6	151742900	-
chr6	151815600	-
chr6	152664100	+
chr6	153346700	-
chr6	153365200	+
chr6	155096700	-
chr6	157384800	-
chr6	157721000	+
chr6	158323100	-
chr6	158367900	-
chr6	158573700	-
chr6	158901900	-
chr6	158985100	+
chr6	160033800	+
chr6	160068700	-
chr6	160103300	-
chr6	160130900	+
chr6	160311000	-
chr6	161333200	-
chr6	161614800	+
chr6	166716400	+
chr6	167289600	+
chr6	167332100	+
chr6	169844500	-
chr6	169864900	+
chr6	169893400	+
chr6	170246500	-
chr6	170422800	-
chr6	170458000	-
chr6	170704300	+
chr6	170735200	+
chr7	590300	+
chr7	733100	-
chr7	822900	-
chr7	929200	+
chr7	1051100	-
chr7	1144100	+
chr7	1166100	+
chr7	1465500	+
chr7	1576000	+
chr7	1946000	+
chr7	2044400	+
chr7	2238500	+
chr7	2248500	-
chr7	2361200	-
chr7	2411700	-
chr7	2561300	+
chr7	2638500	-
chr7	2850100	+
chr7	2876500	-
chr7	4689400	-
chr7	4731400	+
chr7	4782100	-
chr7	5196900	-
chr7	5493700	-
chr7	5519600	+
chr7	5538500	-
chr7	5700800	-
chr7	5829800	-
chr7	6015700	-
chr7	6111500	-
chr7	6381600	-
chr7	6453700	+
chr7	6489700	+
chr7	6596300	+
chr7	6712600	+
chr7	6736200	-
chr7	7189100	-
chr7	10946400	+
chr7	12217900	-
chr7	12693600	-
chr7	16651700	+
chr7	17946300	+
chr7	19714900	+
chr7	22828700	+
chr7	22860700	-
chr7	23019500	+
chr7	23188600	-
chr7	23305700	-
chr7	23537300	+
chr7	24986900	+
chr7	25131200	+
chr7	26159300	-
chr7	27746600	-
chr7	28185900	+
chr7	30141600	-
chr7	30484500	+
chr7	30510500	+
chr7	30601300	-
chr7	32496500	+
chr7	32502200	-
chr7	32897800	+
chr7	33068500	+
chr7	33114800	+
chr7	35700900	+
chr7	35807300	-
chr7	36159700	-
chr7	37349400	-
chr7	37359400	+
chr7	37454400	+
chr7	38915100	+
chr7	39572800	-
chr7	39630100	-
chr7	40140300	+
chr7	42917800	+
chr7	42938500	-
chr7	43735300	+
chr7	43875200	+
chr7	43912100	+
chr7	43932900	-
chr7	44051300	-
chr7	44087700	+
chr7	44129300	+
chr7	44152100	+
chr7	44496300	+
chr7	44579900	+
chr7	44588100	+
chr7	44613000	-
chr7	44803100	-
chr7	44853900	+
chr7	44984500	+
chr7	44992300	+
chr7	45006500	-
chr7	45034100	-
chr7	47985300	+
chr7	48095700	-
chr7	54794000	+
chr7	55608300	-
chr7	56087000	-
chr7	56100000	-
chr7	56141300	+
chr7	63411500	-
chr7	63892400	-
chr7	64853700	-
chr7	65083800	+
chr7	65217600	-
chr7	65694400	+
chr7	65731800	-
chr7	65756700	+
chr7	65843400	-
chr7	66024200	-
chr7	72033900	-
chr7	72573800	+
chr7	72609200	+
chr7	72630600	+
chr7	72735000	+
chr7	72771300	+
chr7	72790800	+
chr7	73226900	-
chr7	73306400	+
chr7	73342100	-
chr7	73709900	-
chr7	74994900	+
chr7	75346600	-
chr7	75382600	-
chr7	75461300	+
chr7	75515600	-
chr7	75770700	-
chr7	75785700	-
chr7	75825900	+
chr7	77164400	-
chr7	77265400	+
chr7	86620200	-
chr7	87343900	-
chr7	87686900	+
chr7	89814200	-
chr7	91408300	-
chr7	91601300	+
chr7	91713500	-
chr7	91995700	-
chr7	92056800	+
chr7	92300700	+
chr7	92585000	+
chr7	92699700	-
chr7	93977500	-
chr7	96176800	+
chr7	97339400	+
chr7	97439300	+
chr7	97574500	-
chr7	97719300	-
chr7	98314700	-
chr7	98579200	+
chr7	98762000	-
chr7	98844100	+
chr7	98874600	-
chr7	98901400	+
chr7	98908600	-
chr7	98935000	+
chr7	98940800	-
chr7	98987700	-
chr7	98993900	+
chr7	99052900	-
chr7	99354700	+
chr7	99451600	-
chr7	99517100	+
chr7	99524700	-
chr7	99536500	+
chr7	99555400	-
chr7	99562600	+
chr7	99656600	-
chr7	99865100	-
chr7	99871600	+
chr7	99975100	-
chr7	100040100	+
chr7	100048500	-
chr7	100141800	-
chr7	100288600	-
chr7	100311100	-
chr7	100325200	+
chr7	100674600	+
chr7	100681900	+
chr7	101286900	+
chr7	101717600	-
chr7	101824000	-
chr7	101860600	-
chr7	101892700	-
chr7	102503100	-
chr7	102725600	-
chr7	102776200	-
chr7	103635100	+
chr7	104695700	+
chr7	104949400	+
chr7	104960100	-
chr7	105106100	-
chr7	105119000	+
chr7	105305100	-
chr7	105539500	+
chr7	106087900	+
chr7	106596800	-
chr7	106991100	+
chr7	107171800	-
chr7	107319200	-
chr7	107953500	+
chr7	107997800	-
chr7	111878000	-
chr7	112217000	+
chr7	112366800	+
chr7	116290400	-
chr7	117611600	-
chr7	122984700	+
chr7	123175800	+
chr7	126819300	+
chr7	127012400	+
chr7	127079500	-
chr7	127770600	+
chr7	127836900	+
chr7	127883300	-
chr7	128166900	-
chr7	128290400	-
chr7	128481700	+
chr7	128652500	-
chr7	129039100	-
chr7	129376300	+
chr7	130663500	-
chr7	132417100	+
chr7	133793800	+
chr7	134483500	-
chr7	134505200	+
chr7	134894000	-
chr7	134997800	-
chr7	135312100	+
chr7	137796200	-
chr7	138370500	+
chr7	138695400	-
chr7	139122200	+
chr7	139408100	+
chr7	140019800	-
chr7	140043600	-
chr7	140270700	+
chr7	140360800	+
chr7	141047600	+
chr7	141084600	-
chr7	142263000	-
chr7	142671400	-
chr7	142695900	-
chr7	142815700	+
chr7	143230000	+
chr7	148027100	-
chr7	148211700	+
chr7	148356300	+
chr7	148476000	-
chr7	148524200	-
chr7	148568100	-
chr7	148590500	-
chr7	148613100	-
chr7	148788100	+
chr7	148825200	+
chr7	148952300	+
chr7	149043200	-
chr7	149166900	-
chr7	149202300	-
chr7	149697000	-
chr7	149707500	-
chr7	149733900	-
chr7	150303000	-
chr7	150336300	-
chr7	150346100	+
chr7	150386500	-
chr7	150554900	+
chr7	150560800	-
chr7	150604700	+
chr7	150669500	-
chr7	150846400	+
chr7	150960200	+
chr7	151354100	-
chr7	154425900	-
chr7	154721900	-
chr7	155130100	-
chr7	156378200	+
chr7	156435700	-
chr7	156625000	-
chr7	156823100	-
chr7	157098400	+
chr7	158190700	-
chr7	158461700	+
chr8	1699600	-
chr8	6553500	-
chr8	8898000	-
chr8	9045400	+
chr8	9451200	-
chr8	10734100	+
chr8	11019500	+
chr8	11179700	-
chr8	11664800	-
chr8	11698100	-
chr8	12656600	+
chr8	17148900	-
chr8	17825100	-
chr8	17985700	+
chr8	19719500	-
chr8	20099500	-
chr8	22022400	+
chr8	22054700	+
chr8	22158800	-
chr8	22464600	+
chr8	22493900	-
chr8	22518900	-
chr8	22581800	+
chr8	22607900	+
chr8	22913800	-
chr8	22931900	+
chr8	22982000	+
chr8	23202200	-
chr8	23370700	+
chr8	23442900	-
chr8	25371800	+
chr8	26296900	-
chr8	26492600	-
chr8	27404800	-
chr8	27685600	+
chr8	28006400	-
chr8	28803800	-
chr8	29176100	+
chr8	30133500	-
chr8	30634700	+
chr8	30704500	+
chr8	30789300	+
chr8	33462300	-
chr8	33543600	+
chr8	37739400	-
chr8	37826500	+
chr8	37875000	+
chr8	38082300	-
chr8	38153300	+
chr8	38208600	-
chr8	38245400	+
chr8	38747200	+
chr8	38972700	+
chr8	41467600	-
chr8	41555200	-
chr8	42028200	+
chr8	42248300	-
chr8	42315500	-
chr8	42369000	-
chr8	42515300	+
chr8	42817300	+
chr8	42871400	-
chr8	43031100	-
chr8	43068100	-
chr8	43115100	-
chr8	49083800	-
chr8	52974000	+
chr8	53789100	+
chr8	54917900	+
chr8	55096800	+
chr8	55176200	+
chr8	56848600	-
chr8	57287300	-
chr8	58068300	+
chr8	59486900	-
chr8	59628800	-
chr8	60193700	-
chr8	61592600	-
chr8	64243900	-
chr8	66708600	+
chr8	67504300	-
chr8	67999800	+
chr8	68136600	+
chr8	68418200	+
chr8	71476400	+
chr8	74084100	-
chr8	74821300	+
chr8	74953200	+
chr8	78075000	+
chr8	81104600	+
chr8	81561800	-
chr8	82795700	+
chr8	86319600	+
chr8	87425800	-
chr8	87596200	-
chr8	90839600	-
chr8	90984100	-
chr8	91726800	+
chr8	92151600	-
chr8	94822000	+
chr8	94998400	-
chr8	95634600	+
chr8	96029900	+
chr8	96106600	-
chr8	96215600	-
chr8	97316400	+
chr8	97343600	-
chr8	98726300	-
chr8	99126800	+
chr8	100094300	+
chr8	100974500	+
chr8	101640700	+
chr8	102208400	-
chr8	102286400	+
chr8	103320200	+
chr8	103493100	+
chr8	103737700	-
chr8	103888800	-
chr8	103944900	+
chr8	104102500	-
chr8	104496800	-
chr8	107739600	-
chr8	109329700	+
chr8	109525100	-
chr8	110415300	+
chr8	117836700	+
chr8	117954200	+
chr8	118602200	-
chr8	121526200	+
chr8	124123100	+
chr8	124154500	-
chr8	124322100	+
chr8	124355800	+
chr8	124622400	+
chr8	124850200	-
chr8	125532400	-
chr8	125620600	-
chr8	126172800	+
chr8	126512600	-
chr8	127638600	+
chr8	128876800	-
chr8	128998500	-
chr8	131020800	+
chr8	131399800	-
chr8	131439200	+
chr8	132986200	-
chr8	133857200	-
chr8	134155900	-
chr8	135794000	+
chr8	141179000	+
chr8	141590500	+
chr8	142208400	-
chr8	142471500	-
chr8	143482200	+
chr8	143747600	+
chr8	143805900	-
chr8	143817300	+
chr8	144400600	-
chr8	144434900	+
chr8	144487900	+
chr8	144522800	-
chr8	144615400	-
chr8	144731300	+
chr8	144762300	+
chr8	144770700	+
chr8	144789600	-
chr8	145132500	+
chr8	145205700	-
chr8	145222300	-
chr8	145520500	+
chr8	145605200	+
chr8	145624300	+
chr8	145640300	+
chr8	145674600	-
chr8	145705300	-
chr8	145951300	+
chr8	145988300	+
chr8	145995400	-
chr8	146024300	-
chr8	146049100	+
chr8	146097900	-
chr8	146198700	+
chr8	146248800	-
chr9	2147800	+
chr9	2833700	+
chr9	4652800	-
chr9	4669800	-
chr9	4730900	+
chr9	4783200	-
chr9	4975200	-
chr9	5427600	+
chr9	5822600	+
chr9	5997700	+
chr9	6404000	-
chr9	7789300	+
chr9	15296700	+
chr9	15413000	-
chr9	15500700	+
chr9	19092300	+
chr9	19117500	+
chr9	19399200	-
chr9	20674600	-
chr9	21793000	-
chr9	26936700	+
chr9	27563100	+
chr9	32515800	+
chr9	32562700	+
chr9	32991200	+
chr9	33015300	-
chr9	33034000	+
chr9	33066500	+
chr9	33156900	+
chr9	33254300	+
chr9	33280800	-
chr9	33436900	+
chr9	33463500	+
chr9	33807600	-
chr9	34038500	+
chr9	34116300	+
chr9	34169300	-
chr9	34319600	-
chr9	34370300	+
chr9	34448000	+
chr9	34601700	+
chr9	34610100	+
chr9	34627600	+
chr9	34637100	-
chr9	34979400	+
chr9	35069500	+
chr9	35086100	+
chr9	35092800	+
chr9	35105700	+
chr9	35595100	-
chr9	35640000	+
chr9	35648700	-
chr9	35738600	+
chr9	35746900	+
chr9	35802000	-
chr9	36027300	-
chr9	36126900	-
chr9	36133900	+
chr9	36181400	-
chr9	36248100	+
chr9	36390500	+
chr9	36477200	+
chr9	37413200	-
chr9	37455000	+
chr9	37476000	-
chr9	37581900	+
chr9	37744100	-
chr9	37774600	+
chr9	37791300	-
chr9	37893700	+
chr9	70585100	-
chr9	70841000	-
chr9	71564200	+
chr9	72063900	-
chr9	73716000	-
chr9	76832500	+
chr9	78199200	+
chr9	78982600	-
chr9	80041000	-
chr9	85512300	+
chr9	87546100	+
chr9	87746300	-
chr9	88087100	+
chr9	88158800	+
chr9	89530400	-
chr9	90193400	-
chr9	91116000	-
chr9	91123800	-
chr9	91410600	+
chr9	93163500	+
chr9	93225800	+
chr9	94095500	+
chr9	94127300	+
chr9	94472100	+
chr9	94566200	+
chr9	94750000	-
chr9	94861700	-
chr9	94899900	-
chr9	94935900	+
chr9	95255600	-
chr9	95969100	-
chr9	96061500	-
chr9	97296900	+
chr9	97309200	+
chr9	97677900	+
chr9	98219700	+
chr9	98252800	-
chr9	98579900	+
chr9	98656200	+
chr9	99214800	-
chr9	99435900	-
chr9	99498900	+
chr9	99787400	-
chr9	99859200	-
chr9	99994400	+
chr9	100057700	+
chr9	100598100	+
chr9	100908900	-
chr9	101024300	-
chr9	101709200	-
chr9	101900900	+
chr9	102154600	+
chr9	103201000	-
chr9	103336100	-
chr9	109085900	-
chr9	110736000	+
chr9	110815200	+
chr9	110921400	+
chr9	112058300	+
chr9	113285300	+
chr9	113433800	-
chr9	113464100	-
chr9	113699500	-
chr9	114519800	+
chr9	114553400	-
chr9	115023400	+
chr9	115077300	+
chr9	115178400	+
chr9	115203000	+
chr9	115212400	+
chr9	115320400	-
chr9	115384100	+
chr9	116199900	+
chr9	116390300	-
chr9	116414100	-
chr9	118489600	-
chr9	122516000	+
chr9	122595200	+
chr9	122645200	-
chr9	123003500	+
chr9	123171800	+
chr9	123894800	+
chr9	123961400	+
chr9	124067000	+
chr9	124630000	+
chr9	124715000	+
chr9	124733800	-
chr9	124743300	-
chr9	125070400	+
chr9	125731700	+
chr9	126217200	+
chr9	126663700	+
chr9	126991500	+
chr9	127043100	+
chr9	127064300	-
chr9	127508800	+
chr9	128607400	-
chr9	128663300	-
chr9	129226700	-
chr9	129253300	+
chr9	129371400	+
chr9	129517400	+
chr9	129578400	+
chr9	129605400	-
chr9	129656800	-
chr9	129707100	+
chr9	129718600	+
chr9	129739600	+
chr9	129870200	+
chr9	129929900	+
chr9	129962700	-
chr9	129993300	+
chr9	130077800	+
chr9	130125000	-
chr9	130143100	-
chr9	130173800	-
chr9	130258700	-
chr9	130307100	-
chr9	130355500	-
chr9	130485900	+
chr9	130526500	-
chr9	130573600	+
chr9	130589700	-
chr9	130621000	-
chr9	130631600	+
chr9	130749500	+
chr9	130839000	-
chr9	130913300	+
chr9	130942500	-
chr9	131038600	+
chr9	131291000	-
chr9	131428900	-
chr9	131443800	+
chr9	131605700	-
chr9	131844700	+
chr9	131856300	-
chr9	132445100	-
chr9	132559200	-
chr9	132578600	-
chr9	132700000	+
chr9	132991000	-
chr9	133368800	-
chr9	133396000	+
chr9	133944900	+
chr9	134107100	+
chr9	134219500	+
chr9	134271500	+
chr9	134535600	-
chr9	134809700	+
chr9	134896000	-
chr9	135013800	+
chr9	135272500	+
chr9	135315200	-
chr9	135991400	-
chr9	136019700	-
chr9	136423200	-
chr9	137532400	-
chr9	137938300	+
chr9	138141600	-
chr9	138452500	+
chr9	138742100	+
chr9	138805800	-
chr9	138821900	+
chr9	138896500	-
chr9	139007100	-
chr9	139101400	-
chr9	139143200	+
chr9	139220100	-
chr9	139237500	+
chr9	139269900	-
chr9	139566200	-
chr9	139592300	+
chr9	139620300	-
chr9	139633700	-
chrX	11039500	-
chrX	15603600	-
chrX	15666600	-
chrX	16798100	+
chrX	19272500	-
chrX	23711800	-
chrX	40325300	-
chrX	40389700	+
chrX	46290200	-
chrX	46318900	-
chrX	46889600	-
chrX	46935700	-
chrX	46963600	-
chrX	47305800	-
chrX	47373700	+
chrX	47394300	+
chrX	48212100	+
chrX	48219900	-
chrX	48252600	-
chrX	48265400	-
chrX	48428100	-
chrX	48440200	-
chrX	48633800	-
chrX	48639600	+
chrX	48699500	+
chrX	48743000	+
chrX	48787500	+
chrX	48818400	+
chrX	48909900	+
chrX	49013100	-
chrX	49531300	-
chrX	53270100	+
chrX	53465600	+
chrX	53727400	+
chrX	54225600	+
chrX	54483800	-
chrX	54851900	-
chrX	63342000	+
chrX	64670800	+
chrX	68301100	+
chrX	69270500	-
chrX	70255500	-
chrX	70390700	+
chrX	70503200	-
chrX	71318300	-
chrX	75565300	-
chrX	77246900	-
chrX	100765400	-
chrX	103298100	-
chrX	106758800	-
chrX	109132900	-
chrX	117993000	-
chrX	118254800	-
chrX	118623700	+
chrX	118961400	+
chrX	119486600	+
chrX	122693900	+
chrX	128868100	-
chrX	129127100	+
chrX	130985400	-
chrX	134013300	+
chrX	134305300	+
chrX	134883400	+
chrX	148520600	+
chrX	149817600	+
chrX	150316500	-
chrX	151750300	-
chrX	152938300	+
chrX	153318900	-
chrX	153397400	+
chrX	153644600	-
chrX	153952900	-
chrX	154098200	-

Cluster GW2 (in hg18)
chr1	1037600	+
chr1	1148100	+
chr1	1217000	+
chr1	1239800	+
chr1	1254800	+
chr1	1266600	-
chr1	1306500	-
chr1	1404500	+
chr1	1463600	+
chr1	1480300	+
chr1	1488100	-
chr1	1604500	+
chr1	2001800	-
chr1	2017500	-
chr1	2101200	+
chr1	2124500	-
chr1	2328500	-
chr1	2389300	+
chr1	2429300	-
chr1	2437300	+
chr1	3398100	+
chr1	3417700	-
chr1	3425600	+
chr1	3775300	-
chr1	5960600	+
chr1	5993200	+
chr1	6033400	+
chr1	6082300	-
chr1	8340900	+
chr1	9307800	+
chr1	9320600	-
chr1	9743300	-
chr1	9751300	+
chr1	10648800	+
chr1	10659100	+
chr1	16142400	+
chr1	16830100	-
chr1	19542400	+
chr1	19820000	+
chr1	25105300	+
chr1	25117000	+
chr1	27599300	-
chr1	38157300	-
chr1	41765100	+
chr1	42566200	+
chr1	109301400	+
chr1	109909300	+
chr1	204731900	+
chr1	224094300	+
chr1	242669700	+
chr10	15228900	-
chr10	28927800	-
chr10	29938200	+
chr10	73502800	-
chr10	133624000	+
chr10	133931900	+
chr10	133943300	-
chr10	133955800	+
chr10	134071800	+
chr10	134268000	-
chr10	134308600	+
chr10	134318900	-
chr10	134429100	+
chr10	134440600	+
chr11	286100	-
chr11	399400	+
chr11	422700	-
chr11	427800	-
chr11	463400	-
chr11	474900	-
chr11	487800	-
chr11	631900	+
chr11	753500	-
chr11	769800	+
chr11	792000	-
chr11	878900	+
chr11	1509800	+
chr11	1541100	+
chr11	1724100	-
chr11	2371100	+
chr11	2527200	-
chr11	2549700	-
chr11	46353000	-
chr11	47521700	+
chr11	63522500	-
chr11	64253900	-
chr11	64278800	-
chr11	64928300	-
chr11	65124000	+
chr11	66149300	+
chr11	67019100	+
chr11	71403100	-
chr11	118276500	+
chr12	780100	-
chr12	46469100	-
chr12	49264900	+
chr12	56407300	+
chr12	78799100	-
chr12	103664700	-
chr12	107540900	-
chr12	119022800	-
chr12	120446500	-
chr12	121896200	-
chr12	129883200	+
chr12	130968200	+
chr12	131611700	+
chr12	131631300	+
chr12	131658000	-
chr12	131805500	+
chr12	131834000	+
chr12	131875100	+
chr13	112403200	-
chr13	112417000	-
chr13	112715600	+
chr13	113021300	+
chr13	113247300	-
chr13	113295100	-
chr13	113570300	-
chr13	113767500	-
chr13	113786000	+
chr13	113793600	-
chr13	113825700	-
chr13	113865900	+
chr13	113874200	+
chr14	20765400	+
chr14	22415500	+
chr14	89073100	+
chr14	90829500	-
chr14	92223200	-
chr14	92615700	+
chr14	96054000	-
chr14	99650100	-
chr14	99656400	-
chr14	101452900	-
chr14	101723400	+
chr14	103082800	+
chr14	103087900	+
chr14	103234200	-
chr14	103261800	+
chr14	104247900	+
chr14	104259400	-
chr14	104588200	-
chr14	104779300	-
chr14	104809500	-
chr14	104817700	-
chr14	104861500	-
chr14	104885400	-
chr14	104984200	-
chr14	104993800	-
chr15	32265700	-
chr15	39919500	+
chr15	49929100	+
chr15	72702500	+
chr15	75106000	+
chr15	75115500	-
chr15	76069600	+
chr15	88390300	+
chr16	257300	+
chr16	358300	-
chr16	456400	+
chr16	510900	-
chr16	524800	-
chr16	539100	+
chr16	586500	+
chr16	600600	+
chr16	611400	+
chr16	651300	-
chr16	676600	+
chr16	706300	+
chr16	719900	+
chr16	860400	+
chr16	884300	+
chr16	903500	-
chr16	910000	-
chr16	1345600	+
chr16	1378100	+
chr16	1450600	-
chr16	1490400	-
chr16	1754100	+
chr16	1777800	+
chr16	1800000	+
chr16	1810300	+
chr16	2224400	-
chr16	2233800	-
chr16	2519300	-
chr16	2683000	+
chr16	2759900	+
chr16	2919500	+
chr16	3016400	-
chr16	3554200	+
chr16	4254800	-
chr16	4330900	+
chr16	4372100	+
chr16	4382100	+
chr16	21431600	-
chr16	25185700	-
chr16	28008700	-
chr16	28016500	-
chr16	28753600	-
chr16	30032200	-
chr16	30883900	-
chr16	48884100	+
chr16	65800400	+
chr16	66534100	-
chr16	80218200	-
chr16	84230700	-
chr16	84245000	+
chr16	85914600	-
chr16	86435500	+
chr16	86565800	+
chr16	86588700	+
chr16	87098500	-
chr16	87119600	-
chr16	87270800	-
chr16	87309100	+
chr16	87321500	+
chr16	87330100	-
chr16	87361400	-
chr16	87670200	+
chr16	87752500	+
chr16	87868800	-
chr16	88211600	-
chr16	88285400	+
chr16	88341100	-
chr16	88478500	+
chr16	88508000	-
chr16	88547600	-
chr16	88589500	+
chr17	251800	+
chr17	2672000	-
chr17	2678700	+
chr17	2728700	-
chr17	2747000	-
chr17	2799300	+
chr17	2813100	-
chr17	3532500	+
chr17	3778800	-
chr17	3787000	+
chr17	3795000	+
chr17	4399200	-
chr17	4734700	+
chr17	7310600	-
chr17	17546200	-
chr17	30425800	+
chr17	34129700	-
chr17	40827900	+
chr17	45570600	-
chr17	52529500	-
chr17	71139800	-
chr17	72997400	+
chr17	73620600	-
chr17	75867100	-
chr17	75874000	-
chr17	75936900	+
chr17	76472900	+
chr17	76588700	+
chr17	76838100	-
chr17	77292400	+
chr17	77403700	-
chr17	77458200	-
chr17	77536700	-
chr17	77545400	-
chr17	77557800	-
chr17	77580200	-
chr17	77801700	+
chr17	77833300	-
chr17	77987400	-
chr17	78103800	-
chr17	78135400	-
chr17	78457400	+
chr18	72283900	+
chr18	72300100	-
chr18	75282000	+
chr18	75288800	+
chr18	75305900	+
chr18	75312800	-
chr18	75340000	+
chr19	260300	-
chr19	604100	+
chr19	766200	+
chr19	812500	-
chr19	823400	-
chr19	831100	-
chr19	841400	+
chr19	850900	-
chr19	883900	-
chr19	944400	-
chr19	1057300	+
chr19	1101200	-
chr19	1113900	+
chr19	1122000	+
chr19	1172200	-
chr19	1206100	+
chr19	1230800	-
chr19	1346700	+
chr19	1376200	+
chr19	1383700	-
chr19	1536700	+
chr19	1565100	+
chr19	1570800	+
chr19	1581300	-
chr19	1777700	-
chr19	1865500	+
chr19	1905700	-
chr19	1949200	-
chr19	2081000	-
chr19	2090800	-
chr19	2124500	+
chr19	2131400	+
chr19	2141600	+
chr19	2150100	+
chr19	2158000	-
chr19	2183800	+
chr19	2226900	+
chr19	2719100	-
chr19	3006500	-
chr19	3326200	+
chr19	3338600	-
chr19	3464200	+
chr19	3478800	+
chr19	3498900	+
chr19	3569300	-
chr19	3616900	+
chr19	3635700	+
chr19	3718400	-
chr19	3915100	-
chr19	3964800	-
chr19	4004200	+
chr19	4053600	-
chr19	4201400	+
chr19	4218700	-
chr19	4744800	+
chr19	4767300	-
chr19	4937500	+
chr19	4963800	+
chr19	4974100	-
chr19	4998700	+
chr19	5027700	+
chr19	5045000	-
chr19	5644500	+
chr19	5847100	-
chr19	5866200	-
chr19	6169000	+
chr19	6181000	-
chr19	6779800	+
chr19	7389700	-
chr19	7448100	+
chr19	7521700	-
chr19	7879100	-
chr19	10088600	-
chr19	10325500	+
chr19	10851500	-
chr19	10876100	-
chr19	12846900	+
chr19	14382200	-
chr19	14517000	+
chr19	14532800	-
chr19	15216700	+
chr19	15228000	+
chr19	15426700	-
chr19	17458700	-
chr19	17548600	-
chr19	17807000	-
chr19	18237300	-
chr19	18421400	-
chr19	18627700	-
chr19	18639600	+
chr19	18680800	+
chr19	18696800	+
chr19	18707700	-
chr19	18742700	-
chr19	18840100	-
chr19	18885600	-
chr19	19366100	+
chr19	38597000	-
chr19	41303500	-
chr19	46517400	-
chr19	46768000	-
chr19	47101700	-
chr19	50327000	-
chr19	50972800	-
chr19	51013300	+
chr19	51872500	-
chr19	51893200	-
chr19	51951900	+
chr19	52866400	-
chr19	54002200	+
chr19	54297500	+
chr19	54643700	+
chr19	54849000	+
chr19	54968900	-
chr19	55022400	-
chr19	55083300	-
chr19	55118700	+
chr19	55573900	+
chr19	56549200	+
chr19	59319000	+
chr19	59656800	+
chr19	60302100	-
chr19	60435300	+
chr19	60443100	+
chr19	60469600	+
chr19	60894600	+
chr19	61303900	-
chr2	3300300	-
chr2	61537500	+
chr2	68309600	-
chr2	97716600	+
chr2	127539300	+
chr2	128776700	+
chr2	149159200	-
chr2	160886800	+
chr2	170405500	+
chr2	197999600	-
chr2	224493100	-
chr2	239651400	-
chr2	241102000	+
chr2	241183300	+
chr2	241217900	+
chr2	241659500	+
chr2	242492900	-
chr2	242540000	-
chr20	349400	+
chr20	25133600	-
chr20	30509300	+
chr20	45623500	+
chr20	57201600	+
chr20	60200500	+
chr20	60911400	+
chr20	61392400	+
chr20	61624500	+
chr20	61692200	+
chr20	61700600	-
chr20	61788400	+
chr20	62009800	-
chr20	62022100	+
chr20	62033600	-
chr20	62126500	-
chr21	42552800	-
chr21	43142000	+
chr21	43984900	-
chr21	44179000	+
chr21	44189400	-
chr21	44409300	-
chr21	44537400	-
chr21	44560200	+
chr21	44578900	+
chr21	44611400	+
chr21	44660600	+
chr21	45134700	-
chr21	45537100	+
chr22	16009500	-
chr22	18683200	-
chr22	19266100	+
chr22	20291300	-
chr22	20512600	-
chr22	20662400	+
chr22	21940100	-
chr22	24086800	-
chr22	35026700	+
chr22	36350100	-
chr22	37464800	-
chr22	38040400	-
chr22	38798500	+
chr22	39170100	-
chr22	41357500	-
chr22	43471900	-
chr22	44859200	-
chr22	44868400	+
chr22	45739200	+
chr22	45806400	+
chr22	45902900	-
chr22	48356300	+
chr22	48366600	-
chr22	48411000	-
chr22	48583700	-
chr22	48726400	-
chr22	48749500	+
chr22	49000600	+
chr22	49015200	+
chr22	49189400	+
chr22	49232800	+
chr22	49242200	+
chr22	49307900	-
chr3	33098100	+
chr3	47018000	-
chr3	47151900	+
chr3	49025300	-
chr3	52240400	-
chr3	128198600	-
chr3	137357200	+
chr3	137932900	-
chr3	184003200	+
chr4	643500	+
chr4	704900	+
chr4	714300	-
chr4	738800	-
chr4	1177300	+
chr4	1208500	-
chr4	1214400	+
chr4	1302300	+
chr4	1547100	-
chr4	1587500	+
chr4	1688800	+
chr4	1962800	-
chr4	2243900	-
chr4	2306900	+
chr4	2329100	+
chr4	2531900	+
chr4	2775600	-
chr4	2927500	+
chr4	3474500	+
chr4	3488900	+
chr4	48425800	+
chr4	78924300	-
chr5	355100	+
chr5	369800	+
chr5	702000	+
chr5	909900	-
chr5	935500	-
chr5	1118600	+
chr5	1129800	+
chr5	1141700	+
chr5	1347200	-
chr5	36940600	-
chr5	36947100	-
chr5	36987900	-
chr5	130663100	-
chr5	130944500	-
chr5	133508900	+
chr5	133923200	-
chr5	133930800	+
chr5	172462500	-
chr5	176966900	+
chr6	24983500	-
chr6	31963300	-
chr6	33381300	+
chr6	35393600	-
chr6	41769900	-
chr6	110617500	-
chr6	149813800	-
chr6	157371200	+
chr7	747100	+
chr7	754300	+
chr7	891500	-
chr7	969900	-
chr7	1023700	+
chr7	1077500	-
chr7	1480200	+
chr7	1546500	+
chr7	1823900	-
chr7	1830600	-
chr7	1872600	-
chr7	1878200	-
chr7	1918100	+
chr7	1971500	+
chr7	2430100	-
chr7	2583900	-
chr7	4759700	+
chr7	4770800	+
chr7	5340100	+
chr7	5507300	+
chr7	5668700	-
chr7	35866600	-
chr7	44063300	-
chr7	45078700	-
chr7	45110800	-
chr7	65660000	+
chr7	75418700	+
chr7	97700000	+
chr7	99903000	+
chr7	129044900	-
chr7	150429200	+
chr7	150452700	-
chr7	151071900	-
chr7	151091400	-
chr7	151726300	-
chr7	156892700	+
chr8	640100	+
chr8	8271800	-
chr8	22502300	-
chr8	61625100	-
chr8	61790100	-
chr8	142298700	-
chr8	142311400	-
chr8	142365500	-
chr8	143440800	+
chr8	144448900	+
chr8	144529500	+
chr8	144594900	+
chr8	144630400	+
chr8	144635300	+
chr8	144644500	+
chr8	144652800	+
chr8	144658400	-
chr8	144949500	-
chr8	144993200	-
chr8	145104700	+
chr8	145138100	+
chr8	145473900	+
chr8	145503900	-
chr8	145597000	-
chr8	146091000	+
chr9	91174600	+
chr9	91187000	-
chr9	94811400	+
chr9	129555800	+
chr9	131677400	+
chr9	133125900	+
chr9	136371900	-
chr9	137959700	-
chr9	137988500	-
chr9	138048800	-
chr9	138055700	+
chr9	138093900	+
chr9	138119000	-
chr9	138267400	+
chr9	138375600	-
chr9	138469700	+
chr9	138512700	+
chr9	138519400	+
chr9	138529400	+
chr9	138645000	+
chr9	138836000	-
chr9	138903900	+
chr9	138954700	-
chr9	139094100	+
chr9	139120700	-
chr9	139227300	-
chr9	139286200	-
chr9	139293900	+
chr9	139430700	-
chr9	139626600	-
chr9	139789600	+
chrX	40840300	-
chrX	46914200	-
chrX	48320200	+
chrX	64867000	+
chrX	152862600	+
chrX	152872100	+
chrX	152951700	-
chrX	152969800	+
chrX	153239900	+
chrX	153246700	-
chrX	153331800	+
chrX	153434600	+

Cluster GW3 (in hg18)
chr1	1680400	-
chr1	2077200	+
chr1	3546300	-
chr1	3757700	-
chr1	6054300	-
chr1	6207000	+
chr1	7966000	+
chr1	8349200	+
chr1	8854700	-
chr1	9333200	+
chr1	10041800	-
chr1	10057500	+
chr1	12175200	+
chr1	15917100	-
chr1	16824900	+
chr1	19552200	-
chr1	22320300	+
chr1	22672900	-
chr1	22688100	+
chr1	26753200	-
chr1	26971300	-
chr1	27050200	+
chr1	27532300	+
chr1	30969800	+
chr1	31236900	+
chr1	32566100	+
chr1	35089500	-
chr1	35853900	+
chr1	36599400	+
chr1	39626800	-
chr1	39800900	-
chr1	39983300	+
chr1	39990900	-
chr1	40654200	+
chr1	40661500	-
chr1	41782000	+
chr1	43659500	-
chr1	43910600	-
chr1	43929900	+
chr1	44880400	-
chr1	53553000	-
chr1	54484200	-
chr1	54499100	-
chr1	55027400	+
chr1	65092600	+
chr1	66601100	+
chr1	90123300	+
chr1	90139300	-
chr1	91178100	-
chr1	95479100	-
chr1	100736700	+
chr1	100748300	-
chr1	110266700	-
chr1	110385600	-
chr1	113031900	-
chr1	116732900	-
chr1	116743000	+
chr1	149527400	+
chr1	152170300	-
chr1	152175800	+
chr1	152410200	+
chr1	154703800	+
chr1	155362100	-
chr1	159050500	+
chr1	159289800	+
chr1	165669900	-
chr1	165929400	+
chr1	173398500	-
chr1	196937800	-
chr1	199217600	-
chr1	199226300	+
chr1	199734600	-
chr1	201117100	+
chr1	201253100	-
chr1	202058200	-
chr1	202778200	-
chr1	204035000	-
chr1	204719800	-
chr1	205143400	-
chr1	218470300	-
chr1	219946500	-
chr1	222629400	-
chr1	224643700	-
chr1	225234900	+
chr1	226749000	+
chr1	227496000	-
chr1	227838200	-
chr1	229897400	+
chr1	244868400	+
chr10	842900	+
chr10	875000	+
chr10	1040700	+
chr10	1123000	+
chr10	1138200	+
chr10	1151500	-
chr10	1160200	+
chr10	3143500	+
chr10	6297400	+
chr10	6505900	+
chr10	6514100	+
chr10	6522200	-
chr10	6538500	-
chr10	7433600	+
chr10	7448100	+
chr10	11409900	+
chr10	14603800	+
chr10	21966600	-
chr10	22897400	+
chr10	26850300	+
chr10	28908700	-
chr10	31748800	+
chr10	33255200	-
chr10	35851800	+
chr10	35878400	-
chr10	43014800	-
chr10	47057400	-
chr10	47095000	-
chr10	50407100	-
chr10	63429500	-
chr10	63441100	+
chr10	63506200	-
chr10	63521300	-
chr10	70799000	+
chr10	70829900	+
chr10	71848200	+
chr10	73261200	-
chr10	75223400	+
chr10	75268700	+
chr10	75542700	+
chr10	76585700	-
chr10	79471300	-
chr10	80620200	-
chr10	80650200	-
chr10	80705100	+
chr10	80730600	+
chr10	80738900	-
chr10	81907000	+
chr10	86167800	+
chr10	90757700	+
chr10	100185100	-
chr10	102298200	-
chr10	103368000	-
chr10	103379500	-
chr10	103406200	+
chr10	103412800	+
chr10	103421600	-
chr10	103790300	-
chr10	103890100	-
chr10	103909400	+
chr10	104032800	-
chr10	104119500	+
chr10	104272500	+
chr10	105631800	+
chr10	114132500	+
chr10	115447100	-
chr10	121074400	-
chr10	121176900	-
chr10	121336900	+
chr10	124605700	-
chr10	124915300	+
chr10	126626000	+
chr10	134079300	+
chr10	134418900	+
chr10	134957600	-
chr10	135084700	-
chr11	588400	+
chr11	892000	+
chr11	935200	-
chr11	966800	-
chr11	986000	+
chr11	992600	+
chr11	1256200	+
chr11	1264100	-
chr11	1448800	+
chr11	2647800	+
chr11	2666600	-
chr11	2930500	-
chr11	2942300	-
chr11	3008100	+
chr11	3014900	-
chr11	3020300	+
chr11	6201400	+
chr11	6476200	+
chr11	7972400	-
chr11	10462700	-
chr11	13335200	-
chr11	35168900	-
chr11	35650900	-
chr11	35773600	+
chr11	44769300	+
chr11	44877200	+
chr11	46381100	-
chr11	46683400	-
chr11	47283900	-
chr11	47974400	-
chr11	48003600	+
chr11	58075100	-
chr11	59132700	-
chr11	60422300	-
chr11	60540600	-
chr11	60971700	+
chr11	60979400	-
chr11	62043200	+
chr11	62133100	-
chr11	62157700	-
chr11	63427700	+
chr11	63431400	+
chr11	64967000	+
chr11	65153400	+
chr11	65490500	+
chr11	65710500	-
chr11	66054900	-
chr11	66777600	+
chr11	66825100	-
chr11	68577000	+
chr11	68588900	-
chr11	70872600	+
chr11	71412300	-
chr11	72083400	+
chr11	72098400	-
chr11	72209100	-
chr11	74179600	+
chr11	74678900	+
chr11	77142900	+
chr11	93545300	+
chr11	95457400	+
chr11	105386500	+
chr11	107583300	-
chr11	116578900	+
chr11	117881200	-
chr11	118507400	+
chr11	120890400	+
chr11	120910900	+
chr11	120932500	+
chr11	127832100	+
chr11	128185000	-
chr11	129261800	-
chr11	133277700	+
chr12	265200	-
chr12	887500	+
chr12	6571900	-
chr12	6658500	-
chr12	6795000	+
chr12	8135100	-
chr12	11751000	+
chr12	31350600	-
chr12	44876200	-
chr12	47373400	-
chr12	47511000	+
chr12	47711600	-
chr12	47723100	-
chr12	52166200	-
chr12	53021700	+
chr12	56177800	+
chr12	56493600	+
chr12	56506900	-
chr12	65012400	+
chr12	65971600	+
chr12	69004900	+
chr12	100971100	+
chr12	103509100	-
chr12	103671800	+
chr12	104140900	+
chr12	105244500	-
chr12	105250400	+
chr12	107461600	-
chr12	108901300	-
chr12	109258300	-
chr12	109460900	-
chr12	111919800	-
chr12	112210400	+
chr12	112240600	+
chr12	116077900	+
chr12	116964400	+
chr12	119082900	-
chr12	119106500	+
chr12	119616200	+
chr12	119695900	-
chr12	120267800	-
chr12	122060300	+
chr12	122999800	+
chr12	123415600	+
chr12	123456600	-
chr12	123464900	+
chr12	123491000	+
chr12	130784100	-
chr12	130797300	+
chr12	130824300	-
chr12	130837000	-
chr12	130845300	-
chr12	131069800	-
chr12	131077000	-
chr12	131087000	+
chr12	131100600	+
chr12	131600700	+
chr12	131953000	-
chr13	19116100	-
chr13	19515700	-
chr13	23763300	+
chr13	23771100	-
chr13	29023300	+
chr13	30223400	-
chr13	40236300	-
chr13	42398500	+
chr13	48638700	+
chr13	50407200	-
chr13	73285200	-
chr13	73466500	+
chr13	97913000	-
chr13	98396500	+
chr13	102313300	-
chr13	110085300	-
chr13	110102700	+
chr13	110701500	+
chr13	110709400	+
chr13	110737400	+
chr13	112264000	+
chr13	112924200	-
chr13	113014900	-
chr13	113326000	-
chr14	23748400	+
chr14	23830500	+
chr14	30661800	-
chr14	38905700	-
chr14	44477900	-
chr14	50345000	-
chr14	51490200	+
chr14	60994400	+
chr14	61077100	-
chr14	64024100	-
chr14	64059100	-
chr14	64273400	+
chr14	64496300	-
chr14	67007100	-
chr14	68435300	-
chr14	68590600	+
chr14	69247400	-
chr14	69320200	+
chr14	69885200	-
chr14	69935600	+
chr14	72541200	+
chr14	73263400	-
chr14	73832400	-
chr14	75084400	-
chr14	75705200	+
chr14	77233400	+
chr14	88712200	-
chr14	88723300	+
chr14	88732000	+
chr14	88852300	+
chr14	89012900	+
chr14	89021900	-
chr14	89100600	+
chr14	90843100	-
chr14	90857900	-
chr14	90874600	-
chr14	92215800	-
chr14	92487600	-
chr14	92525000	-
chr14	92565200	-
chr14	92592600	-
chr14	93597100	+
chr14	94638500	+
chr14	95010200	+
chr14	99030900	+
chr14	99045000	-
chr14	101552900	+
chr14	101731200	-
chr14	101744600	+
chr14	102404400	+
chr14	102992700	+
chr14	102998500	+
chr14	104752600	-
chr15	23531500	+
chr15	27133400	+
chr15	36595900	-
chr15	47115100	-
chr15	50233500	-
chr15	50750900	-
chr15	61330200	-
chr15	61619600	-
chr15	61854100	-
chr15	66164200	-
chr15	68130000	-
chr15	68139900	+
chr15	70286600	-
chr15	70297100	-
chr15	70345600	+
chr15	71676600	-
chr15	72103700	+
chr15	72112800	-
chr15	72649400	+
chr15	75123800	+
chr15	76086900	-
chr15	76100500	+
chr15	77397900	-
chr15	77925100	-
chr15	81302600	-
chr15	83142800	-
chr15	84113200	-
chr15	87667400	-
chr15	88412100	-
chr15	88433900	-
chr15	88955700	-
chr15	88986600	-
chr15	89250900	+
chr15	90450700	-
chr15	97162700	+
chr15	98974400	-
chr15	100067900	-
chr16	109000	+
chr16	916600	+
chr16	945900	+
chr16	1389100	-
chr16	1610900	+
chr16	1622100	+
chr16	1632200	-
chr16	1641400	-
chr16	1717400	-
chr16	2576100	+
chr16	2689900	-
chr16	3132100	-
chr16	3476100	+
chr16	3524700	+
chr16	3546900	-
chr16	3631900	+
chr16	4392000	-
chr16	8631300	+
chr16	8822500	+
chr16	11556700	-
chr16	11699800	+
chr16	12136100	+
chr16	15715500	+
chr16	16100000	-
chr16	17145800	-
chr16	19630800	-
chr16	22226500	+
chr16	22240500	-
chr16	23405800	+
chr16	23412600	-
chr16	23975000	+
chr16	23981800	+
chr16	23995700	+
chr16	24060800	-
chr16	24678100	+
chr16	24696200	+
chr16	24741400	+
chr16	25036900	-
chr16	27461600	-
chr16	28046700	+
chr16	28236800	+
chr16	31046100	-
chr16	31417900	-
chr16	47129400	-
chr16	49302900	+
chr16	55340400	+
chr16	55626700	-
chr16	55661000	-
chr16	65633500	+
chr16	65738200	-
chr16	66036100	+
chr16	66044200	-
chr16	66334700	+
chr16	66365700	+
chr16	66501000	+
chr16	66520900	+
chr16	66930700	-
chr16	67906800	-
chr16	69312000	+
chr16	69337600	-
chr16	69376000	+
chr16	70511900	+
chr16	80166700	+
chr16	80197900	+
chr16	80278500	-
chr16	80753000	-
chr16	82499100	-
chr16	82510600	+
chr16	82646500	+
chr16	82680600	+
chr16	83120200	-
chr16	83162900	+
chr16	83252400	-
chr16	83581400	+
chr16	84222100	+
chr16	84372100	+
chr16	84377700	-
chr16	84396200	-
chr16	85934900	-
chr16	85952300	+
chr16	86015300	-
chr16	86300700	+
chr16	86313500	+
chr16	86331700	-
chr16	86339200	-
chr16	86346300	+
chr16	86606200	+
chr16	86654000	+
chr16	86659800	+
chr16	87199900	-
chr16	87205400	+
chr16	87216700	-
chr16	87436300	+
chr16	87726800	-
chr16	87738100	+
chr16	88116900	+
chr16	88486400	-
chr16	88500000	-
chr17	292700	-
chr17	639500	-
chr17	884900	-
chr17	929900	+
chr17	1510800	+
chr17	1915900	+
chr17	2715000	+
chr17	2807800	+
chr17	4648800	+
chr17	4822300	-
chr17	5389600	-
chr17	5405800	-
chr17	7168500	+
chr17	7340600	-
chr17	8076400	+
chr17	8082800	+
chr17	8315100	+
chr17	8727800	+
chr17	12848500	+
chr17	16271600	+
chr17	16976300	+
chr17	16984200	-
chr17	16990300	-
chr17	17012300	+
chr17	17073100	+
chr17	17328900	+
chr17	17942700	-
chr17	20864200	-
chr17	23985800	+
chr17	24234300	-
chr17	24454400	+
chr17	24472900	-
chr17	24983100	-
chr17	30615200	-
chr17	33020200	-
chr17	34324800	+
chr17	35245500	+
chr17	35404100	+
chr17	35435200	-
chr17	36049900	-
chr17	37263200	-
chr17	38114500	+
chr17	40669600	+
chr17	40716800	-
chr17	43642500	+
chr17	45048900	-
chr17	45150400	+
chr17	45271000	+
chr17	45534100	+
chr17	45786500	-
chr17	52335500	+
chr17	52538400	+
chr17	52991500	-
chr17	53927900	+
chr17	54820700	+
chr17	59077600	-
chr17	59106200	-
chr17	59480400	-
chr17	62223500	+
chr17	62565700	-
chr17	62958700	+
chr17	62983600	-
chr17	63002200	+
chr17	68708800	-
chr17	70572700	-
chr17	71191600	-
chr17	71557600	+
chr17	71596900	+
chr17	71906400	+
chr17	71915000	+
chr17	72223800	+
chr17	73003100	+
chr17	73562200	-
chr17	73907500	+
chr17	74187700	+
chr17	74310600	-
chr17	74499100	-
chr17	76148000	-
chr17	76222600	-
chr17	76358300	-
chr17	76851500	-
chr17	77158000	+
chr17	78014300	+
chr17	78030300	+
chr17	78151400	+
chr17	78212000	+
chr17	78341000	-
chr17	78353300	+
chr17	78379300	-
chr17	78423800	+
chr17	78449600	-
chr18	2995700	-
chr18	9176000	+
chr18	9224200	+
chr18	12317200	-
chr18	12353300	-
chr18	13536000	-
chr18	27895100	+
chr18	30909900	-
chr18	30930600	+
chr18	30967500	+
chr18	41787800	-
chr18	41924100	-
chr18	42095100	-
chr18	43675000	-
chr18	46957500	+
chr18	58370200	-
chr18	70330900	+
chr18	70904500	+
chr18	71128700	+
chr18	72861400	-
chr18	72866600	-
chr18	75188300	+
chr18	75198600	+
chr18	75273600	+
chr18	75322200	-
chr18	75350700	+
chr18	75357500	+
chr18	75558400	+
chr18	75564700	-
chr18	75580800	-
chr18	75610300	+
chr18	75779200	+
chr18	75788300	-
chr18	75803500	-
chr19	279200	+
chr19	286500	-
chr19	1340300	+
chr19	2072400	-
chr19	2688000	+
chr19	2753800	-
chr19	3396500	+
chr19	3701300	-
chr19	3987000	-
chr19	4443800	+
chr19	4956800	+
chr19	4991500	+
chr19	5009400	+
chr19	5876000	-
chr19	5884800	+
chr19	6705400	+
chr19	6712500	+
chr19	6772100	+
chr19	7336400	-
chr19	7346500	-
chr19	7354600	+
chr19	7373500	+
chr19	7949300	+
chr19	7959200	+
chr19	10230400	-
chr19	10336000	+
chr19	10463100	+
chr19	10651700	-
chr19	10956900	+
chr19	12703300	-
chr19	13107600	-
chr19	13968200	-
chr19	15235900	-
chr19	16053700	+
chr19	16096700	+
chr19	16198500	+
chr19	16388900	-
chr19	16809900	+
chr19	16824900	+
chr19	16840200	+
chr19	17073400	-
chr19	17113700	+
chr19	17173600	-
chr19	17706100	+
chr19	18096300	+
chr19	18283600	+
chr19	18433000	-
chr19	18826200	-
chr19	18878800	+
chr19	19015300	+
chr19	19064600	+
chr19	19314000	-
chr19	19464300	+
chr19	19470300	+
chr19	19497100	-
chr19	19627900	+
chr19	34794400	+
chr19	37555700	+
chr19	38571100	+
chr19	38627800	-
chr19	39564500	-
chr19	39574800	+
chr19	40454000	+
chr19	40910500	+
chr19	43491500	-
chr19	44509600	-
chr19	44615900	-
chr19	44640900	-
chr19	45022500	-
chr19	45454500	-
chr19	45900300	+
chr19	45960500	-
chr19	46470500	+
chr19	46629500	-
chr19	46786300	-
chr19	47603000	-
chr19	48750300	-
chr19	48808300	-
chr19	50255300	+
chr19	51886800	-
chr19	52188500	+
chr19	52263900	+
chr19	52270000	-
chr19	52549300	-
chr19	52686300	-
chr19	52693400	-
chr19	52859800	+
chr19	52888500	-
chr19	53666800	+
chr19	54808400	-
chr19	54976200	-
chr19	54986900	+
chr19	55058900	-
chr19	55078400	+
chr19	55104100	+
chr19	55183200	+
chr19	55203500	-
chr19	55211100	-
chr19	55226600	+
chr19	55993800	+
chr19	60309600	-
chr19	61292800	+
chr19	63522400	-
chr19	63597200	-
chr2	659300	+
chr2	3164000	+
chr2	3249300	-
chr2	3261700	-
chr2	3273100	+
chr2	3313200	-
chr2	3372500	-
chr2	3408200	+
chr2	3429700	+
chr2	3486500	+
chr2	7025300	+
chr2	9473300	-
chr2	9541400	-
chr2	10414400	+
chr2	10479700	+
chr2	11823300	+
chr2	11843300	+
chr2	12799800	+
chr2	20692400	-
chr2	25317200	+
chr2	25323300	-
chr2	26178100	+
chr2	47052300	+
chr2	47075700	-
chr2	47082600	-
chr2	47094000	+
chr2	47101800	-
chr2	47127000	+
chr2	54710200	-
chr2	54720200	-
chr2	54726000	-
chr2	55056400	-
chr2	55064700	-
chr2	61112400	+
chr2	62306600	-
chr2	63962500	-
chr2	68888100	-
chr2	69546000	-
chr2	69579900	-
chr2	69592200	+
chr2	71440700	+
chr2	74161900	-
chr2	74218400	-
chr2	86557000	+
chr2	86837800	-
chr2	96283900	+
chr2	96635900	-
chr2	98535100	-
chr2	98553800	+
chr2	98573900	-
chr2	101265800	-
chr2	106109800	+
chr2	112537600	+
chr2	113133800	+
chr2	113670700	-
chr2	118462800	+
chr2	121959100	-
chr2	127172500	+
chr2	127523800	-
chr2	131529400	-
chr2	131600700	-
chr2	131613200	-
chr2	135548800	+
chr2	143770600	+
chr2	143978500	-
chr2	144226300	+
chr2	148436900	-
chr2	152380900	-
chr2	160865000	+
chr2	161737900	-
chr2	173161900	-
chr2	175147000	-
chr2	175722400	-
chr2	179105700	+
chr2	191558700	-
chr2	191569800	+
chr2	197597400	-
chr2	197668000	-
chr2	201632700	+
chr2	201710300	-
chr2	201724800	+
chr2	204531700	-
chr2	206629500	-
chr2	215899800	+
chr2	216672500	+
chr2	225552600	-
chr2	230782100	-
chr2	230955300	+
chr2	233698500	+
chr2	233706500	+
chr2	233764400	+
chr2	233776400	-
chr2	233836700	+
chr2	234009400	-
chr2	234018900	+
chr2	234037200	+
chr2	237154100	-
chr2	238320700	+
chr2	238333200	-
chr2	238747500	+
chr2	238756000	+
chr2	239020300	-
chr2	239666700	+
chr2	239684500	+
chr2	239696100	-
chr2	239730200	+
chr2	239746400	+
chr2	239773700	-
chr2	239782200	+
chr2	239977700	-
chr2	240552000	+
chr2	240565300	+
chr2	240605700	+
chr2	241057300	-
chr2	241089700	+
chr2	241114300	+
chr2	241677500	+
chr2	241851700	+
chr2	242086800	-
chr2	242239900	+
chr2	242259300	-
chr2	242308200	-
chr2	242487400	+
chr20	1064100	+
chr20	1076200	+
chr20	1082500	+
chr20	1089700	+
chr20	1099600	+
chr20	1564800	-
chr20	2790300	+
chr20	2916400	+
chr20	3793100	-
chr20	4827400	+
chr20	4855700	-
chr20	17571900	-
chr20	21266000	+
chr20	24893800	-
chr20	24939600	+
chr20	25205500	-
chr20	29613600	+
chr20	30487000	-
chr20	32129800	+
chr20	32578100	-
chr20	32586500	+
chr20	33548800	+
chr20	33558100	+
chr20	33683300	-
chr20	33979800	-
chr20	34297000	+
chr20	34303700	+
chr20	35574300	+
chr20	35772600	-
chr20	36074900	-
chr20	36965700	+
chr20	39471300	+
chr20	39595100	+
chr20	42686600	-
chr20	43063100	-
chr20	43905500	+
chr20	45310600	-
chr20	45374100	+
chr20	46707000	-
chr20	46741700	+
chr20	46753100	-
chr20	46990100	+
chr20	47880600	-
chr20	48146500	-
chr20	48183200	-
chr20	48943200	-
chr20	49503900	+
chr20	51621700	+
chr20	54514900	+
chr20	56353800	-
chr20	56704000	+
chr20	56917900	+
chr20	57033700	+
chr20	57216800	+
chr20	59992900	+
chr20	60022900	-
chr20	60140800	-
chr20	60271500	+
chr20	60286700	-
chr20	60993200	+
chr20	61720700	-
chr20	61766300	+
chr20	61980200	+
chr20	61987200	+
chr20	62371300	-
chr21	16055000	-
chr21	16076000	-
chr21	33845400	-
chr21	34437500	-
chr21	35152000	-
chr21	37806900	+
chr21	42570100	+
chr21	42956600	+
chr21	43134300	+
chr21	43159200	+
chr21	43997400	-
chr21	44037700	-
chr21	44144100	-
chr21	44218900	-
chr21	44362600	+
chr21	44620400	+
chr21	45054600	-
chr21	45191200	+
chr21	45201400	+
chr21	45378700	-
chr21	45407600	-
chr21	45510400	-
chr21	45527100	-
chr21	45774000	-
chr21	46464000	-
chr21	46489000	-
chr21	46510200	+
chr21	46630100	-
chr21	46734000	+
chr21	46906900	-
chr22	17411000	+
chr22	17415700	+
chr22	17431800	+
chr22	17438300	+
chr22	17500400	+
chr22	17507500	+
chr22	17772600	-
chr22	17836900	-
chr22	18189000	+
chr22	18330000	-
chr22	18411500	-
chr22	18422100	+
chr22	18431800	-
chr22	18457000	-
chr22	18490600	-
chr22	19521300	+
chr22	20130900	+
chr22	20357200	-
chr22	20491600	-
chr22	20629100	+
chr22	21955800	+
chr22	22505200	+
chr22	22827900	-
chr22	23274000	+
chr22	25454500	+
chr22	28556900	+
chr22	29062000	-
chr22	29310800	+
chr22	29659800	+
chr22	29912700	-
chr22	30053200	-
chr22	34383500	-
chr22	35780200	+
chr22	35932300	-
chr22	36391700	-
chr22	36890700	+
chr22	36973400	-
chr22	37452600	+
chr22	37861500	+
chr22	39152200	-
chr22	40050600	+
chr22	40162400	+
chr22	40258700	-
chr22	40891900	+
chr22	41310900	+
chr22	41321800	-
chr22	41614200	-
chr22	41633700	+
chr22	42932500	-
chr22	43506500	-
chr22	45127700	+
chr22	45413600	-
chr22	45470100	+
chr22	45483100	+
chr22	45567700	+
chr22	45586400	+
chr22	45637600	+
chr22	45655900	-
chr22	45691700	-
chr22	45709400	+
chr22	45726100	+
chr22	45733000	+
chr22	45766400	+
chr22	45771300	+
chr22	45827500	+
chr22	45887300	-
chr22	45910100	-
chr22	45933100	-
chr22	48306700	-
chr22	48389500	-
chr22	48666000	+
chr22	48680400	-
chr22	48991700	+
chr22	49009400	+
chr22	49303200	+
chr3	5228600	-
chr3	9491500	+
chr3	9696300	+
chr3	11571100	+
chr3	11583600	+
chr3	11592100	+
chr3	13343800	-
chr3	13357400	+
chr3	13371200	+
chr3	13391200	+
chr3	13395200	-
chr3	14131700	-
chr3	14175200	+
chr3	14184400	+
chr3	14432100	+
chr3	14677900	+
chr3	15074700	+
chr3	15098300	-
chr3	15276900	-
chr3	16365100	+
chr3	16382400	-
chr3	16417900	-
chr3	17056500	+
chr3	20088200	+
chr3	36848500	-
chr3	36873300	-
chr3	36906600	-
chr3	38034200	+
chr3	38144000	-
chr3	41242200	+
chr3	42532600	+
chr3	42586700	+
chr3	43315700	+
chr3	44956300	-
chr3	44973600	+
chr3	45947400	+
chr3	46703300	-
chr3	46939300	-
chr3	47447100	-
chr3	49711500	+
chr3	50659900	+
chr3	52302200	+
chr3	52498400	-
chr3	53008000	-
chr3	56737800	-
chr3	56752900	+
chr3	63956300	+
chr3	66519400	+
chr3	66542500	+
chr3	67758100	-
chr3	71109000	+
chr3	99156100	+
chr3	102643400	+
chr3	107054100	-
chr3	109254300	-
chr3	120430200	+
chr3	121971000	+
chr3	126215200	+
chr3	126230700	-
chr3	127648700	-
chr3	128190500	+
chr3	129270100	-
chr3	129287900	-
chr3	129530200	+
chr3	129536100	-
chr3	129543700	-
chr3	129594000	+
chr3	129827300	+
chr3	130377600	-
chr3	142645300	-
chr3	151048700	+
chr3	168910600	+
chr3	172357700	+
chr3	172539700	+
chr3	178277300	+
chr3	184692900	-
chr3	184701400	-
chr3	185342900	-
chr3	185443000	+
chr3	188184100	-
chr3	188269800	-
chr3	188276100	-
chr3	189397600	-
chr3	196559400	+
chr3	197089900	+
chr3	198758700	-
chr4	491600	-
chr4	516100	-
chr4	745400	-
chr4	756700	-
chr4	853700	+
chr4	870800	-
chr4	883500	+
chr4	1009300	-
chr4	1337700	+
chr4	1813900	-
chr4	2314400	+
chr4	2343000	-
chr4	2365700	+
chr4	2481600	-
chr4	2602800	+
chr4	2610300	-
chr4	2659900	+
chr4	2798500	+
chr4	2880400	+
chr4	3200800	-
chr4	4351600	-
chr4	6648500	-
chr4	6854700	-
chr4	6927200	+
chr4	7052800	+
chr4	7070400	-
chr4	8291300	-
chr4	8467200	+
chr4	54611500	-
chr4	57491800	-
chr4	68261000	-
chr4	89406700	+
chr4	89746700	-
chr4	103707400	+
chr4	110764600	-
chr4	129990200	+
chr4	140195100	-
chr4	143174900	-
chr4	154700400	-
chr4	154747600	-
chr4	185555400	+
chr5	259700	-
chr5	363200	+
chr5	916100	+
chr5	1518900	+
chr5	1539500	+
chr5	1860000	-
chr5	1867400	+
chr5	6423700	-
chr5	10485400	-
chr5	10780000	-
chr5	10801400	-
chr5	14765900	-
chr5	14837700	+
chr5	32309800	-
chr5	35904300	-
chr5	37526200	-
chr5	40727600	-
chr5	54687900	-
chr5	67628400	+
chr5	75897500	-
chr5	79500600	-
chr5	94022500	+
chr5	96157300	+
chr5	98274300	-
chr5	108712500	-
chr5	110730800	-
chr5	112270500	-
chr5	133937800	+
chr5	134249800	-
chr5	140989300	+
chr5	142672300	-
chr5	149369400	-
chr5	150394900	+
chr5	150468200	+
chr5	150481600	-
chr5	150545900	-
chr5	156655900	+
chr5	156670600	+
chr5	156690400	+
chr5	156698000	+
chr5	156711400	-
chr5	156720200	+
chr5	156734700	-
chr5	156893200	+
chr5	158545600	+
chr5	167912700	+
chr5	169035600	-
chr5	169104700	-
chr5	169128200	-
chr5	169138100	-
chr5	169170600	-
chr5	169228300	+
chr5	169242800	-
chr5	169351200	+
chr5	171454600	-
chr5	176698000	+
chr5	176954600	+
chr5	177506800	+
chr5	179132100	-
chr5	179263600	+
chr5	180152700	+
chr6	565700	-
chr6	614400	+
chr6	2724100	-
chr6	3028600	-
chr6	3051000	-
chr6	4891300	+
chr6	7131300	-
chr6	7175300	-
chr6	12229500	+
chr6	13695900	+
chr6	13702400	+
chr6	15581800	-
chr6	15601300	+
chr6	15607100	-
chr6	16252800	-
chr6	16435000	+
chr6	25232100	+
chr6	26573900	+
chr6	30274000	-
chr6	30787800	-
chr6	31486500	-
chr6	31582400	+
chr6	33732000	-
chr6	33761800	-
chr6	33851900	-
chr6	36286600	+
chr6	36570100	+
chr6	37051900	-
chr6	38212400	+
chr6	38221200	-
chr6	41799500	-
chr6	43014300	+
chr6	43100700	+
chr6	43431900	+
chr6	44180900	-
chr6	44472500	-
chr6	44521300	+
chr6	52377300	+
chr6	52539800	+
chr6	53234400	-
chr6	70513500	+
chr6	70531100	+
chr6	86346600	+
chr6	88023900	-
chr6	105942400	-
chr6	109788200	-
chr6	109900500	+
chr6	112101400	-
chr6	119017700	+
chr6	128081900	-
chr6	130570200	-
chr6	134341400	+
chr6	138241700	-
chr6	143121500	+
chr6	149742000	-
chr6	149822200	+
chr6	155145300	+
chr6	157453600	+
chr6	157487700	+
chr6	158210500	-
chr6	158340300	+
chr6	158360700	-
chr6	158937700	-
chr6	159125400	-
chr6	160377100	+
chr6	160383700	+
chr6	160391900	+
chr6	160420100	+
chr6	160446200	+
chr6	161382600	+
chr6	167272500	-
chr6	170480600	+
chr6	170500000	+
chr6	170508900	-
chr6	170516800	+
chr6	170548200	-
chr7	705400	+
chr7	873500	-
chr7	1494900	+
chr7	1851400	+
chr7	2082800	-
chr7	2148600	+
chr7	2226900	+
chr7	2382100	+
chr7	2438300	+
chr7	2548700	+
chr7	2950900	+
chr7	3002900	+
chr7	5001500	+
chr7	5359000	-
chr7	5368200	-
chr7	5683800	-
chr7	6647300	-
chr7	32538000	+
chr7	32876200	+
chr7	35673400	+
chr7	36899600	-
chr7	36909500	+
chr7	37202700	+
chr7	43925300	-
chr7	44398000	-
chr7	44410400	-
chr7	44680600	+
chr7	47973200	-
chr7	48108600	+
chr7	50436300	+
chr7	55500000	+
chr7	65742500	+
chr7	75355900	+
chr7	75797100	+
chr7	98358200	-
chr7	98390000	-
chr7	98884800	-
chr7	99789700	+
chr7	100318100	-
chr7	104565500	+
chr7	105043200	+
chr7	105069900	-
chr7	105076500	+
chr7	105083200	-
chr7	105093700	+
chr7	105226200	-
chr7	106971500	-
chr7	126794000	-
chr7	127121500	+
chr7	127417700	-
chr7	127468700	+
chr7	127505300	+
chr7	127513300	-
chr7	133786800	-
chr7	137262000	+
chr7	138404700	+
chr7	138423900	+
chr7	139062300	+
chr7	139369400	+
chr7	139505700	-
chr7	141036900	+
chr7	148595000	+
chr7	148778000	-
chr7	148889000	+
chr7	148944700	-
chr7	149189600	-
chr7	150362300	-
chr7	150919900	+
chr7	154728900	-
chr7	156849900	-
chr7	156901000	+
chr7	158244800	+
chr7	158268300	-
chr7	158281200	+
chr8	656000	-
chr8	6411400	-
chr8	10685000	+
chr8	10711700	+
chr8	22163400	-
chr8	22528600	+
chr8	22950200	+
chr8	22956500	+
chr8	26306300	+
chr8	30555900	-
chr8	37851000	+
chr8	38895700	+
chr8	38906300	+
chr8	38929800	+
chr8	41575900	-
chr8	41909700	+
chr8	42282500	-
chr8	54804800	+
chr8	61817400	-
chr8	67865900	+
chr8	71209100	+
chr8	71250100	-
chr8	82064900	-
chr8	102261600	-
chr8	103413700	-
chr8	123954100	+
chr8	123965600	+
chr8	123981100	+
chr8	124012300	+
chr8	124025900	-
chr8	124034100	-
chr8	124308900	-
chr8	124613600	+
chr8	126180400	-
chr8	129024300	-
chr8	129031300	-
chr8	134119200	+
chr8	134340000	-
chr8	134547200	-
chr8	140845900	+
chr8	140884600	-
chr8	140911100	-
chr8	141039400	-
chr8	141154300	-
chr8	141432400	-
chr8	141625400	+
chr8	141640400	-
chr8	142250600	-
chr8	143432400	-
chr8	144406400	+
chr8	144738800	+
chr8	144843100	-
chr8	145294600	-
chr8	145480600	+
chr8	145646400	-
chr8	146143600	-
chr9	2132400	+
chr9	5010600	+
chr9	6917600	-
chr9	19110800	+
chr9	32978400	-
chr9	33456600	-
chr9	33895100	-
chr9	34097600	-
chr9	34231900	+
chr9	35052500	+
chr9	35079900	+
chr9	35696700	+
chr9	36194600	-
chr9	36202400	+
chr9	37432000	+
chr9	70851400	+
chr9	79538100	+
chr9	81418700	+
chr9	91198900	-
chr9	94456900	-
chr9	94822300	-
chr9	94916300	+
chr9	95296100	-
chr9	95328000	+
chr9	95335800	-
chr9	95437500	+
chr9	95452800	+
chr9	95459800	+
chr9	95984500	-
chr9	99811000	-
chr9	114062600	-
chr9	115339300	-
chr9	116162500	-
chr9	122365100	-
chr9	127316500	+
chr9	127388100	-
chr9	128636100	-
chr9	128682500	-
chr9	129551700	+
chr9	129611500	+
chr9	129688500	+
chr9	129909400	+
chr9	129970000	+
chr9	129980700	-
chr9	130190200	-
chr9	130372400	+
chr9	130431200	+
chr9	130557200	-
chr9	130815500	+
chr9	131617900	-
chr9	131655100	-
chr9	131659300	-
chr9	133298200	-
chr9	133310700	+
chr9	133335500	+
chr9	133342300	-
chr9	133478500	+
chr9	133490100	-
chr9	133906600	+
chr9	133921900	+
chr9	133937300	+
chr9	134207500	+
chr9	134544100	-
chr9	134966500	-
chr9	136008600	+
chr9	137889100	-
chr9	137970400	+
chr9	138257200	-
chr9	138488400	+
chr9	138877800	-
chr9	139585100	+
chr9	139746300	+
chr9	139802800	+
chr9	139840900	-
chrX	48445200	+
chrX	148383300	-

Cluster GW4 (in hg18)
chr1	747500	-
chr1	882100	-
chr1	1185000	+
chr1	1197300	-
chr1	1784200	-
chr1	1794500	-
chr1	1802600	-
chr1	2315400	+
chr1	3652200	-
chr1	3696900	-
chr1	5985400	+
chr1	6047800	+
chr1	6574600	+
chr1	6610400	+
chr1	8365100	-
chr1	8373100	-
chr1	8380300	+
chr1	8391700	+
chr1	8401400	+
chr1	8413200	-
chr1	8535200	-
chr1	9227100	-
chr1	9242400	-
chr1	9284800	-
chr1	9298200	+
chr1	9576200	-
chr1	9659900	+
chr1	9670500	+
chr1	9686000	-
chr1	9791000	-
chr1	10029100	-
chr1	11268400	+
chr1	11277900	-
chr1	11723900	+
chr1	11906900	-
chr1	12162400	+
chr1	12192700	-
chr1	12567500	-
chr1	12575100	-
chr1	12584400	-
chr1	12592800	-
chr1	13923200	-
chr1	13953200	-
chr1	13961100	-
chr1	13968200	-
chr1	13994700	-
chr1	14000800	+
chr1	16589700	+
chr1	16647900	-
chr1	19127000	+
chr1	19140800	+
chr1	19274000	+
chr1	19321900	+
chr1	19559300	+
chr1	19569000	+
chr1	19589200	-
chr1	19599100	-
chr1	19606700	-
chr1	19629400	-
chr1	19637500	+
chr1	19647600	+
chr1	19653800	-
chr1	19668200	+
chr1	19679900	-
chr1	20979200	+
chr1	21468800	-
chr1	21507100	+
chr1	21512600	+
chr1	21524400	+
chr1	22266600	+
chr1	22275700	+
chr1	22299300	+
chr1	22304900	+
chr1	22311200	+
chr1	22661000	+
chr1	23339800	-
chr1	24176300	-
chr1	24625500	+
chr1	25154500	+
chr1	25235500	+
chr1	25754900	-
chr1	25771300	+
chr1	25847000	-
chr1	25857200	+
chr1	25903800	+
chr1	25910700	+
chr1	25925600	+
chr1	25930400	+
chr1	25940900	+
chr1	25954500	+
chr1	25973700	-
chr1	25996100	+
chr1	26900800	-
chr1	26911300	-
chr1	26926800	-
chr1	26933100	-
chr1	26949200	+
chr1	27627000	-
chr1	27637700	+
chr1	27647300	+
chr1	27674600	+
chr1	27767600	+
chr1	27795000	-
chr1	28086000	-
chr1	28955200	+
chr1	29097600	-
chr1	29149900	+
chr1	29155700	+
chr1	29182000	-
chr1	29369700	-
chr1	31256400	+
chr1	31266200	+
chr1	31275100	+
chr1	31300600	+
chr1	31878000	+
chr1	32150500	-
chr1	32365600	+
chr1	33411000	-
chr1	33497100	+
chr1	33511000	-
chr1	33577300	+
chr1	36124800	-
chr1	36589400	+
chr1	36616600	+
chr1	38090200	+
chr1	39460100	+
chr1	39468700	-
chr1	39507800	+
chr1	39636400	-
chr1	39651500	+
chr1	40286300	-
chr1	41001700	-
chr1	41885100	-
chr1	41965700	+
chr1	41977400	-
chr1	42448600	+
chr1	42480600	+
chr1	42926800	+
chr1	45917000	-
chr1	46939300	-
chr1	51506000	+
chr1	53546400	+
chr1	54507600	-
chr1	54517800	-
chr1	54529200	-
chr1	54541900	+
chr1	54550200	+
chr1	54561000	-
chr1	54570200	-
chr1	54586800	-
chr1	54595200	+
chr1	54603200	-
chr1	54615000	-
chr1	54626800	+
chr1	55418200	-
chr1	55434200	-
chr1	55441500	-
chr1	65100800	-
chr1	65113600	-
chr1	65129900	+
chr1	65153000	+
chr1	65165300	-
chr1	65171900	-
chr1	65187100	-
chr1	65288000	-
chr1	66590800	+
chr1	67177000	-
chr1	84402000	+
chr1	84462900	-
chr1	87145400	+
chr1	88976100	+
chr1	89105800	-
chr1	89499600	+
chr1	89874900	-
chr1	89910500	+
chr1	91164100	-
chr1	91231300	-
chr1	92068200	-
chr1	100604200	-
chr1	100618700	-
chr1	111974600	+
chr1	111992500	-
chr1	112004400	-
chr1	112017000	+
chr1	112970600	+
chr1	114132800	-
chr1	114284800	-
chr1	114811800	-
chr1	116724900	-
chr1	117730900	-
chr1	117959900	-
chr1	117967000	+
chr1	118003000	+
chr1	144191300	+
chr1	149551100	+
chr1	152092400	+
chr1	152121200	-
chr1	152418500	+
chr1	152658700	+
chr1	152667200	+
chr1	152681300	+
chr1	152841000	+
chr1	153228200	+
chr1	153255500	+
chr1	153751000	-
chr1	154080200	+
chr1	154204700	-
chr1	154365100	+
chr1	154436100	+
chr1	154718800	+
chr1	157252300	+
chr1	158871400	+
chr1	158917600	-
chr1	160013300	-
chr1	163882000	+
chr1	163921300	-
chr1	165678200	+
chr1	165683000	-
chr1	165709400	-
chr1	165721800	+
chr1	165731900	+
chr1	165738900	-
chr1	165746000	-
chr1	165878300	-
chr1	165890300	+
chr1	165899500	-
chr1	165908700	+
chr1	166465600	+
chr1	166478800	-
chr1	169734100	-
chr1	169746900	+
chr1	170020000	+
chr1	173406500	+
chr1	173418800	+
chr1	174421900	-
chr1	178205600	+
chr1	179211800	+
chr1	181723600	+
chr1	181737000	-
chr1	191303300	-
chr1	196434100	-
chr1	199244200	+
chr1	199252800	-
chr1	201032600	+
chr1	201874500	+
chr1	201900300	+
chr1	201912200	+
chr1	201917900	-
chr1	201929200	+
chr1	202033900	+
chr1	202715100	+
chr1	203546900	-
chr1	203552600	+
chr1	204041400	-
chr1	204822600	+
chr1	204931400	-
chr1	204947600	-
chr1	204956500	-
chr1	204965800	+
chr1	205586400	+
chr1	206048100	+
chr1	206055600	-
chr1	206071200	-
chr1	206084000	+
chr1	206093400	-
chr1	209578900	-
chr1	209593600	+
chr1	210535900	-
chr1	210555600	-
chr1	211000800	+
chr1	221981500	+
chr1	222080300	+
chr1	224103300	+
chr1	224121000	+
chr1	224658400	-
chr1	224888600	+
chr1	224907500	-
chr1	224915100	-
chr1	224921400	+
chr1	224939500	+
chr1	224952700	-
chr1	224967400	+
chr1	224980000	-
chr1	225214700	+
chr1	226002000	-
chr1	226016000	-
chr1	226028600	+
chr1	226426600	+
chr1	226658500	-
chr1	228287500	+
chr1	228308000	+
chr1	228314200	+
chr1	228328700	-
chr1	228389500	+
chr1	229587200	-
chr1	229760200	-
chr1	233489300	+
chr1	233516000	-
chr1	234379200	-
chr1	244814100	-
chr1	244829100	-
chr1	244838800	-
chr10	859800	+
chr10	883100	+
chr10	902300	-
chr10	907500	-
chr10	942000	-
chr10	958300	+
chr10	3127700	-
chr10	5857400	-
chr10	6128400	+
chr10	6230600	+
chr10	6255700	+
chr10	6567400	+
chr10	6594400	-
chr10	6602300	+
chr10	6610200	-
chr10	6619500	-
chr10	6646000	+
chr10	7341800	+
chr10	7379800	-
chr10	7398000	+
chr10	7440400	+
chr10	7471500	+
chr10	7990700	-
chr10	8151700	+
chr10	11264300	+
chr10	11273900	+
chr10	11284000	+
chr10	11289500	-
chr10	11309500	+
chr10	11318700	+
chr10	11328300	+
chr10	11337600	-
chr10	11343800	-
chr10	11365100	+
chr10	12441400	+
chr10	12583500	+
chr10	14615600	-
chr10	14622800	+
chr10	14635600	-
chr10	14642600	-
chr10	14682900	-
chr10	22034400	+
chr10	22922100	+
chr10	22930000	-
chr10	22942500	+
chr10	22951700	-
chr10	22966400	+
chr10	22972000	+
chr10	23005500	+
chr10	23019900	-
chr10	23037000	-
chr10	26785600	-
chr10	26799100	-
chr10	27110400	-
chr10	27134300	+
chr10	27174200	+
chr10	28884500	+
chr10	28889300	-
chr10	28898700	-
chr10	32378600	-
chr10	32636900	+
chr10	32647400	-
chr10	32657600	-
chr10	32667800	+
chr10	33268000	-
chr10	35403800	+
chr10	35670900	+
chr10	35681900	+
chr10	35697100	-
chr10	35708400	+
chr10	35726400	-
chr10	35743500	-
chr10	35756700	-
chr10	35777500	+
chr10	35788300	-
chr10	35798900	-
chr10	35820900	-
chr10	35828400	+
chr10	35840000	-
chr10	42961200	-
chr10	42971400	+
chr10	42977300	-
chr10	45373900	+
chr10	47069600	-
chr10	47081400	-
chr10	51246100	+
chr10	52040400	+
chr10	61299600	-
chr10	61310000	-
chr10	61318500	+
chr10	61324100	+
chr10	63340300	+
chr10	63351600	+
chr10	63392700	+
chr10	63416000	-
chr10	64671000	-
chr10	64688000	-
chr10	70767900	+
chr10	70779700	+
chr10	70790000	+
chr10	71593700	-
chr10	73191800	+
chr10	73266700	+
chr10	73765600	+
chr10	75237200	-
chr10	75278600	-
chr10	75288800	-
chr10	76316700	+
chr10	76327400	-
chr10	80513400	-
chr10	80532400	+
chr10	80545700	+
chr10	80568000	+
chr10	80579600	+
chr10	80697300	+
chr10	80751100	-
chr10	81922600	-
chr10	81930100	+
chr10	81945500	-
chr10	81950800	-
chr10	82224200	-
chr10	82240600	-
chr10	82248200	+
chr10	82279600	-
chr10	86084400	+
chr10	86092200	+
chr10	86112600	+
chr10	89666500	-
chr10	89675600	+
chr10	90646300	-
chr10	92960500	+
chr10	93681600	+
chr10	98653000	+
chr10	99239500	+
chr10	103394100	-
chr10	104014600	-
chr10	104243200	+
chr10	104398800	+
chr10	104410700	-
chr10	104502000	-
chr10	104526600	+
chr10	104536200	+
chr10	104546100	+
chr10	105418800	+
chr10	106058700	+
chr10	106067900	-
chr10	106083500	+
chr10	111771800	-
chr10	111782800	-
chr10	111789000	-
chr10	111810700	+
chr10	111845400	-
chr10	111852300	+
chr10	116241600	-
chr10	116249500	+
chr10	116258300	-
chr10	120482200	+
chr10	121021000	+
chr10	121062800	-
chr10	121097300	+
chr10	121109800	-
chr10	121123900	+
chr10	121143200	+
chr10	121275200	+
chr10	121281000	-
chr10	121287900	+
chr10	121408400	-
chr10	121418100	+
chr10	124749600	+
chr10	126304900	-
chr10	126319900	-
chr10	126337300	+
chr10	126357100	-
chr10	126368200	-
chr10	126377400	+
chr10	126388100	+
chr10	126396200	-
chr10	126413700	+
chr10	126614100	-
chr10	126622000	+
chr10	129752900	+
chr10	131844300	+
chr10	133579300	-
chr10	133584100	-
chr10	133592100	+
chr10	134062000	+
chr10	134207500	-
chr10	134219300	-
chr10	134235400	-
chr10	134253400	-
chr10	134258300	+
chr10	134293000	-
chr10	134351500	+
chr10	134376200	-
chr11	830500	-
chr11	919600	+
chr11	946000	+
chr11	960300	-
chr11	1007400	+
chr11	1285200	+
chr11	1476900	-
chr11	1502200	+
chr11	1734600	-
chr11	1868500	+
chr11	2364100	-
chr11	2444600	-
chr11	2450100	+
chr11	2919700	+
chr11	2960600	+
chr11	3028100	+
chr11	3802500	-
chr11	3843000	-
chr11	3857100	-
chr11	3872400	-
chr11	3913200	+
chr11	3931100	-
chr11	3944800	-
chr11	3962600	-
chr11	3974800	+
chr11	6382700	+
chr11	6392700	+
chr11	6586300	+
chr11	9376200	-
chr11	10455500	-
chr11	11849500	-
chr11	13259800	+
chr11	13276300	+
chr11	13285900	+
chr11	13309500	-
chr11	13317600	+
chr11	13323200	+
chr11	13658900	-
chr11	18593600	+
chr11	33701000	+
chr11	34045600	-
chr11	35130300	-
chr11	35151400	-
chr11	43346600	-
chr11	44079200	+
chr11	44547900	+
chr11	44556300	-
chr11	44601100	-
chr11	44712600	-
chr11	44840600	+
chr11	45908900	+
chr11	46331600	+
chr11	46393200	+
chr11	46675200	-
chr11	47477900	-
chr11	47502700	+
chr11	48022500	+
chr11	48034300	+
chr11	48058600	+
chr11	48068500	-
chr11	58087100	+
chr11	58128400	-
chr11	60519500	+
chr11	60529200	+
chr11	60550700	-
chr11	60636100	+
chr11	60653900	+
chr11	63374900	-
chr11	63393200	-
chr11	63404700	-
chr11	63716500	-
chr11	64396100	+
chr11	64913200	+
chr11	65076900	+
chr11	65141700	+
chr11	65635700	-
chr11	65643900	+
chr11	65812600	-
chr11	66168000	+
chr11	66895100	+
chr11	67724100	+
chr11	67734300	-
chr11	67996500	+
chr11	68005000	-
chr11	68016200	+
chr11	68028300	+
chr11	68052100	-
chr11	68066900	-
chr11	68073300	-
chr11	68341600	-
chr11	68821500	-
chr11	69836600	-
chr11	72128900	+
chr11	72772900	-
chr11	74172800	+
chr11	74346600	-
chr11	74880300	-
chr11	77160300	+
chr11	77178700	+
chr11	77190100	+
chr11	77199500	+
chr11	82558700	+
chr11	85433300	+
chr11	85442600	+
chr11	85448500	-
chr11	93523700	-
chr11	95395100	+
chr11	95529100	-
chr11	95673700	-
chr11	95687900	+
chr11	101786700	+
chr11	101798600	-
chr11	101805700	-
chr11	101824700	+
chr11	107401600	-
chr11	107575300	-
chr11	116161400	-
chr11	116397600	-
chr11	116450400	+
chr11	117377400	-
chr11	117617500	+
chr11	117817800	-
chr11	117829700	+
chr11	120839700	-
chr11	120846800	-
chr11	120859000	+
chr11	120872400	+
chr11	122045900	+
chr11	122092700	-
chr11	122131300	+
chr11	125704200	+
chr11	128075800	+
chr11	128084900	-
chr11	128097000	+
chr11	128104000	+
chr11	128116500	+
chr11	128133300	+
chr11	128141300	+
chr11	128147800	+
chr11	128158800	+
chr11	128165700	-
chr11	128178100	+
chr11	133319800	-
chr11	133605400	-
chr12	770000	+
chr12	2777400	+
chr12	3723300	-
chr12	4256900	+
chr12	4266100	-
chr12	4272400	-
chr12	6303600	+
chr12	8082900	+
chr12	11706600	+
chr12	11720800	+
chr12	11789700	-
chr12	14469000	+
chr12	19496400	+
chr12	19501900	-
chr12	21487000	+
chr12	22680200	+
chr12	44659600	-
chr12	44917300	+
chr12	45916600	-
chr12	46483200	-
chr12	48245500	-
chr12	48430200	-
chr12	49301100	-
chr12	49927200	+
chr12	51877100	-
chr12	52062800	-
chr12	53091200	-
chr12	55683800	-
chr12	56419300	-
chr12	56514500	+
chr12	60949000	-
chr12	60955300	-
chr12	60961200	-
chr12	63311200	+
chr12	63319500	+
chr12	63332500	-
chr12	63869800	+
chr12	64836700	-
chr12	64992000	-
chr12	65957500	+
chr12	66337900	+
chr12	66346300	+
chr12	67301700	+
chr12	67927800	-
chr12	68205000	+
chr12	68952000	-
chr12	70331400	+
chr12	70447300	+
chr12	70545800	+
chr12	75388400	-
chr12	75420400	+
chr12	75470200	-
chr12	75695400	-
chr12	78838100	+
chr12	88566100	-
chr12	88575700	+
chr12	88584500	+
chr12	88592200	-
chr12	88607000	-
chr12	92331600	-
chr12	92343600	-
chr12	93901300	-
chr12	95126100	-
chr12	95144200	-
chr12	95152600	-
chr12	95159200	+
chr12	95167800	-
chr12	95293100	-
chr12	103387800	+
chr12	103403100	-
chr12	103425000	-
chr12	103431700	+
chr12	103437900	+
chr12	103450400	+
chr12	103466100	-
chr12	103472700	+
chr12	103487600	-
chr12	103495500	-
chr12	103503400	-
chr12	103521800	-
chr12	103530000	+
chr12	103539100	-
chr12	103547400	+
chr12	103552800	+
chr12	103562500	+
chr12	103571900	-
chr12	103588900	+
chr12	103601200	+
chr12	103612900	-
chr12	103626300	-
chr12	103630800	+
chr12	103648600	-
chr12	103659000	+
chr12	106249800	-
chr12	106255000	-
chr12	106669100	-
chr12	107535100	-
chr12	107756100	-
chr12	108384700	-
chr12	108392200	+
chr12	109271400	-
chr12	109489300	-
chr12	109660200	-
chr12	110331700	+
chr12	110336700	-
chr12	110348100	-
chr12	110358600	+
chr12	110367400	+
chr12	111909200	-
chr12	111992700	-
chr12	112013900	+
chr12	112155000	-
chr12	115166600	+
chr12	115847100	+
chr12	117269100	-
chr12	119163700	-
chr12	119172100	-
chr12	119460700	+
chr12	119759000	+
chr12	119777400	-
chr12	120377900	-
chr12	120553700	+
chr12	120560100	+
chr12	120704400	-
chr12	120734000	-
chr12	121158500	+
chr12	121174600	+
chr12	121924900	+
chr12	121938900	-
chr12	122084700	-
chr12	122116600	-
chr12	123442800	+
chr12	123482200	-
chr12	123530500	-
chr12	123548400	-
chr12	123567500	-
chr12	123575300	-
chr12	123593400	+
chr12	123605900	+
chr12	123614800	-
chr12	127860800	-
chr12	130955400	-
chr12	131009400	+
chr12	131117600	+
chr12	131193200	-
chr12	131578800	+
chr12	131605200	-
chr12	131893700	+
chr12	131960600	+
chr13	23750600	+
chr13	26641200	-
chr13	26913700	-
chr13	27103600	-
chr13	27638400	+
chr13	29010800	-
chr13	29054200	-
chr13	29062600	+
chr13	30102500	+
chr13	30216400	+
chr13	40038000	+
chr13	40044700	-
chr13	40050600	+
chr13	40062900	+
chr13	40069600	+
chr13	40079200	+
chr13	40086400	+
chr13	40101400	+
chr13	40112400	-
chr13	40121100	+
chr13	40126200	+
chr13	40132000	-
chr13	40434500	-
chr13	40442800	+
chr13	45642500	+
chr13	46043600	-
chr13	46102400	-
chr13	50389500	+
chr13	73313200	+
chr13	73343900	+
chr13	73370900	-
chr13	73411500	-
chr13	73423500	+
chr13	73440500	+
chr13	73456900	-
chr13	73512100	-
chr13	73527700	-
chr13	73542500	-
chr13	73560000	+
chr13	73573400	-
chr13	76770900	+
chr13	76781600	+
chr13	76788800	+
chr13	78122200	+
chr13	95157500	+
chr13	95192400	+
chr13	96692100	+
chr13	96711500	-
chr13	96752600	-
chr13	97928900	+
chr13	97940400	+
chr13	97947500	-
chr13	97957300	+
chr13	97973600	-
chr13	97979600	+
chr13	97988100	+
chr13	97993900	+
chr13	98000200	+
chr13	98007900	-
chr13	98017100	-
chr13	98411400	-
chr13	98448700	-
chr13	98494000	+
chr13	98509500	+
chr13	98528900	+
chr13	98656600	+
chr13	98668400	+
chr13	98676800	+
chr13	98964100	-
chr13	98977100	+
chr13	106015100	-
chr13	107675900	-
chr13	110077300	-
chr13	110617600	-
chr13	110627200	-
chr13	110635500	-
chr13	110653000	+
chr13	110664100	-
chr13	112276800	+
chr13	112428000	-
chr13	112445400	-
chr13	112456400	-
chr13	112470900	+
chr13	112485100	-
chr13	112906200	-
chr13	112917800	-
chr13	113007400	+
chr13	113305200	-
chr13	113309500	+
chr13	113316500	+
chr13	114010300	+
chr13	114103100	-
chr14	23725200	-
chr14	30728800	+
chr14	34842500	+
chr14	49117100	+
chr14	50334100	-
chr14	50358100	-
chr14	51408700	+
chr14	51423000	+
chr14	51434400	+
chr14	51449200	+
chr14	51459600	+
chr14	51467200	-
chr14	51476000	-
chr14	52223400	-
chr14	56792500	-
chr14	60895000	-
chr14	60904500	-
chr14	60915600	-
chr14	60922300	+
chr14	60931600	-
chr14	60950800	-
chr14	60957400	+
chr14	60976200	+
chr14	60982100	+
chr14	61008300	+
chr14	61015700	-
chr14	61029500	+
chr14	61047000	-
chr14	61054200	+
chr14	61062200	+
chr14	61088700	-
chr14	61255500	-
chr14	63047300	+
chr14	63474700	-
chr14	64476000	+
chr14	64482900	+
chr14	64616200	+
chr14	64630900	-
chr14	67018300	-
chr14	68474900	+
chr14	68482900	+
chr14	68492500	+
chr14	68504200	-
chr14	68654000	-
chr14	68670300	+
chr14	68678700	-
chr14	69156800	+
chr14	69165900	-
chr14	69180800	+
chr14	69186900	-
chr14	69197000	-
chr14	69210000	-
chr14	69221500	-
chr14	69231100	-
chr14	69239500	-
chr14	70202700	+
chr14	70462300	-
chr14	73302100	+
chr14	73311500	+
chr14	74706000	+
chr14	77120200	-
chr14	88700700	-
chr14	88738700	-
chr14	88756700	-
chr14	88766900	-
chr14	88777400	+
chr14	88791400	-
chr14	88805400	+
chr14	88812100	+
chr14	88821200	+
chr14	88829500	-
chr14	88839600	-
chr14	88861800	+
chr14	88885800	-
chr14	88900200	-
chr14	88907900	-
chr14	88924400	+
chr14	88936700	-
chr14	88945100	+
chr14	88978800	-
chr14	89005700	+
chr14	89045700	-
chr14	89054500	+
chr14	89087500	-
chr14	89110400	-
chr14	89125600	-
chr14	90573800	+
chr14	90681700	+
chr14	90768900	+
chr14	90868100	+
chr14	90893200	+
chr14	90900700	-
chr14	90911200	+
chr14	90924100	-
chr14	90942100	+
chr14	91033200	-
chr14	91376100	+
chr14	91396400	-
chr14	92149400	-
chr14	92196300	-
chr14	92205800	+
chr14	92228800	-
chr14	92337300	+
chr14	92540000	+
chr14	92551000	+
chr14	92570500	+
chr14	92581500	+
chr14	92602300	+
chr14	92628600	+
chr14	93495500	-
chr14	94994100	+
chr14	95016400	-
chr14	95032300	+
chr14	95043300	+
chr14	97485100	-
chr14	97500300	-
chr14	98740100	+
chr14	98744700	+
chr14	98752300	-
chr14	98765700	-
chr14	99005400	+
chr14	99619200	+
chr14	99633800	-
chr14	99642200	+
chr14	99683400	-
chr14	99793200	-
chr14	99811400	-
chr14	99960500	-
chr14	101371200	+
chr14	101390700	+
chr14	101400900	-
chr14	101700500	-
chr14	101717700	+
chr14	101736300	-
chr14	101755400	-
chr14	102181300	-
chr14	102331000	-
chr14	102342500	+
chr14	102355300	-
chr14	102362400	-
chr14	102937300	-
chr14	103067700	+
chr14	103174200	-
chr14	104229200	+
chr14	104552000	-
chr14	104598700	-
chr14	104823500	+
chr14	104831100	+
chr14	104870600	+
chr14	104977800	-
chr15	23536500	-
chr15	23591500	+
chr15	23600700	-
chr15	23607600	-
chr15	23627700	-
chr15	23634500	-
chr15	26998800	-
chr15	27015900	-
chr15	27028000	-
chr15	27035000	+
chr15	27064800	-
chr15	27082500	-
chr15	27117300	-
chr15	27144600	-
chr15	29057500	-
chr15	29064200	-
chr15	29429100	+
chr15	29447000	+
chr15	32379100	+
chr15	36612000	+
chr15	36634200	-
chr15	38522500	+
chr15	38531400	-
chr15	38866700	-
chr15	39870300	-
chr15	39911700	-
chr15	41831700	+
chr15	42528000	+
chr15	43725500	+
chr15	43738300	-
chr15	49806700	-
chr15	49941500	+
chr15	50639500	+
chr15	56770600	+
chr15	56777100	+
chr15	56812000	-
chr15	58469400	+
chr15	58614100	+
chr15	58622400	+
chr15	58631200	-
chr15	58644000	+
chr15	58653200	+
chr15	58663300	+
chr15	60133700	+
chr15	61280900	+
chr15	61291100	-
chr15	61323700	-
chr15	61604400	+
chr15	61843500	-
chr15	61886400	-
chr15	62191100	+
chr15	62924700	+
chr15	62934800	-
chr15	64473800	+
chr15	64488100	-
chr15	64513100	+
chr15	65156600	+
chr15	65166000	+
chr15	65172300	+
chr15	65182500	+
chr15	65189400	-
chr15	65195900	+
chr15	65228400	+
chr15	65241400	+
chr15	65254000	-
chr15	66142000	-
chr15	66369500	+
chr15	66862900	+
chr15	66869000	-
chr15	66889800	+
chr15	68149100	-
chr15	68158000	-
chr15	68164600	+
chr15	68172000	+
chr15	68583500	-
chr15	70302300	+
chr15	70438900	+
chr15	70447600	-
chr15	70562100	-
chr15	70572900	+
chr15	70583500	+
chr15	70595800	+
chr15	71681700	-
chr15	72094300	-
chr15	72626300	+
chr15	72697900	+
chr15	72937500	+
chr15	72946900	-
chr15	73526000	+
chr15	75083000	-
chr15	75094900	+
chr15	75536000	-
chr15	76118200	+
chr15	76138500	-
chr15	76534200	-
chr15	79391300	+
chr15	81523000	-
chr15	83048200	+
chr15	83104800	-
chr15	83114500	+
chr15	83130000	-
chr15	83734000	+
chr15	83752800	+
chr15	83819900	+
chr15	83877600	-
chr15	83942300	+
chr15	83969000	+
chr15	84129600	-
chr15	86997900	+
chr15	88351800	+
chr15	88379400	+
chr15	88554800	+
chr15	88574700	-
chr15	88745600	+
chr15	88757400	+
chr15	88902400	+
chr15	89301500	+
chr15	90205100	-
chr15	90210900	+
chr15	90230900	-
chr15	90248200	-
chr15	90254800	+
chr15	90264700	-
chr15	90287400	+
chr15	90298400	+
chr15	90325700	+
chr15	90336500	-
chr15	90363400	+
chr15	90377800	+
chr15	90387400	+
chr15	90413600	-
chr15	90419500	+
chr15	90440400	+
chr15	91265900	+
chr15	97060100	+
chr15	97065300	-
chr15	97076500	-
chr15	97093000	+
chr15	99002400	-
chr15	99600800	-
chr16	74700	+
chr16	299400	-
chr16	324200	+
chr16	330600	+
chr16	336800	+
chr16	954100	+
chr16	1369100	-
chr16	1461200	-
chr16	1549100	-
chr16	2583000	-
chr16	3460100	-
chr16	3775700	+
chr16	3781600	+
chr16	3788600	+
chr16	3798000	-
chr16	3807300	+
chr16	3814000	+
chr16	3839800	+
chr16	3858800	-
chr16	4362600	+
chr16	4485800	+
chr16	4504300	-
chr16	4630400	-
chr16	4637200	-
chr16	4667100	+
chr16	8930800	-
chr16	8944200	+
chr16	8953200	+
chr16	8959700	-
chr16	9110300	-
chr16	9126600	-
chr16	10950300	-
chr16	10966500	+
chr16	10989800	+
chr16	11141500	-
chr16	11573100	-
chr16	11673600	+
chr16	11901200	+
chr16	12534900	+
chr16	12568100	-
chr16	15990100	-
chr16	16026100	-
chr16	16063200	-
chr16	17248100	+
chr16	17349100	+
chr16	17441300	+
chr16	17452600	+
chr16	17462700	-
chr16	21528000	+
chr16	22285700	-
chr16	23767200	-
chr16	23777300	-
chr16	23807600	+
chr16	23816600	+
chr16	23826500	+
chr16	23894100	+
chr16	23953500	-
chr16	24072400	+
chr16	24660300	+
chr16	24671200	-
chr16	24894900	+
chr16	24904800	+
chr16	24916100	+
chr16	27149200	-
chr16	27245500	+
chr16	27260500	+
chr16	27345800	+
chr16	28026900	+
chr16	28093200	+
chr16	28100300	-
chr16	28105700	-
chr16	28121900	+
chr16	28217300	+
chr16	28481400	+
chr16	28493000	-
chr16	29749200	+
chr16	30580300	+
chr16	30983400	+
chr16	31622100	+
chr16	31797000	-
chr16	47147900	-
chr16	47168700	+
chr16	47189300	-
chr16	47197600	+
chr16	48796000	+
chr16	48876000	-
chr16	49194500	+
chr16	55595900	+
chr16	55608600	+
chr16	55619200	+
chr16	55634300	+
chr16	65197800	+
chr16	66048800	+
chr16	66056600	-
chr16	66066200	+
chr16	66352600	+
chr16	66372400	-
chr16	66448500	-
chr16	66508000	+
chr16	66584100	-
chr16	66688000	-
chr16	66704900	-
chr16	66713400	-
chr16	66948300	+
chr16	68363600	-
chr16	69010200	-
chr16	69329700	+
chr16	69383800	+
chr16	70387500	+
chr16	70447000	-
chr16	70500700	-
chr16	73156400	-
chr16	73161800	-
chr16	73169300	-
chr16	73251000	+
chr16	80043800	+
chr16	80051000	+
chr16	80072700	+
chr16	80079500	+
chr16	80089500	-
chr16	80106300	+
chr16	80140800	-
chr16	80154200	-
chr16	80188800	-
chr16	80207300	-
chr16	80223600	-
chr16	80270100	-
chr16	82697100	+
chr16	83170300	+
chr16	83198100	-
chr16	83205500	-
chr16	83241500	+
chr16	83304200	+
chr16	83328800	-
chr16	83350800	+
chr16	83586500	-
chr16	83594500	+
chr16	84155600	+
chr16	84163100	+
chr16	84181000	+
chr16	85961900	+
chr16	86027100	-
chr16	86056100	+
chr16	86063700	+
chr16	86294300	+
chr16	86305500	+
chr16	86547700	-
chr16	86554900	+
chr16	87072700	+
chr16	87173300	-
chr16	87179400	+
chr16	87353000	+
chr16	87691900	-
chr16	87717900	-
chr16	87891600	+
chr16	87919900	+
chr16	87938900	-
chr16	87945000	+
chr16	87971700	+
chr16	87980100	-
chr16	87989600	+
chr16	88007900	-
chr16	88017200	+
chr16	88028000	-
chr16	88041300	+
chr16	88047300	-
chr16	88075600	+
chr16	88159200	-
chr17	945400	-
chr17	1225300	-
chr17	1241800	+
chr17	1297500	+
chr17	1410900	-
chr17	1973700	-
chr17	2042100	-
chr17	2454600	-
chr17	2463400	+
chr17	2658200	+
chr17	2753800	-
chr17	2780400	-
chr17	2854500	+
chr17	3737500	+
chr17	3987600	-
chr17	4172800	+
chr17	4192300	+
chr17	4691500	+
chr17	4700800	+
chr17	5358400	+
chr17	7093900	-
chr17	8308400	-
chr17	8741800	-
chr17	8760100	+
chr17	8779800	+
chr17	8790600	-
chr17	14153400	+
chr17	14162000	-
chr17	14174000	-
chr17	14183400	-
chr17	16263700	-
chr17	16896800	+
chr17	16907200	-
chr17	16916600	+
chr17	16932300	-
chr17	16947800	-
chr17	16954500	+
chr17	16962600	-
chr17	16969800	+
chr17	17664100	-
chr17	17670900	+
chr17	17780400	+
chr17	18042500	-
chr17	18161600	+
chr17	18745400	+
chr17	18836400	-
chr17	18868100	-
chr17	19215000	+
chr17	20875600	-
chr17	21046800	-
chr17	21055200	-
chr17	22894900	-
chr17	23395200	+
chr17	24278500	+
chr17	24291600	-
chr17	24638100	+
chr17	25099700	-
chr17	30599200	+
chr17	34295800	+
chr17	35053200	-
chr17	35063600	+
chr17	35175700	-
chr17	35333800	-
chr17	35485100	-
chr17	35740500	+
chr17	35958000	+
chr17	37538600	-
chr17	37547500	+
chr17	37768300	+
chr17	39544100	-
chr17	39872100	+
chr17	40321100	-
chr17	40475600	+
chr17	40689200	-
chr17	40726500	+
chr17	40732500	+
chr17	41540800	+
chr17	41555200	-
chr17	41566200	-
chr17	41571100	-
chr17	41587100	-
chr17	41596200	-
chr17	41604700	+
chr17	41617700	+
chr17	42376400	+
chr17	43139500	+
chr17	43338700	-
chr17	43485600	+
chr17	43627200	-
chr17	43659900	-
chr17	43673200	-
chr17	43810600	-
chr17	43818200	+
chr17	43848000	+
chr17	45091900	-
chr17	45160900	-
chr17	45188400	+
chr17	46162800	+
chr17	52702200	+
chr17	52713900	-
chr17	52721400	-
chr17	52726900	-
chr17	52758800	+
chr17	52772400	+
chr17	52787200	-
chr17	52863600	-
chr17	52872600	-
chr17	52887100	-
chr17	52894600	-
chr17	52918500	+
chr17	52969000	-
chr17	54784800	-
chr17	55059300	-
chr17	55066800	+
chr17	59439500	-
chr17	59523300	+
chr17	59546800	-
chr17	60450400	+
chr17	60455100	-
chr17	60463400	-
chr17	60964900	-
chr17	60976800	-
chr17	60983300	+
chr17	61777800	-
chr17	61791300	-
chr17	61801200	+
chr17	61814500	+
chr17	61842900	+
chr17	61852700	-
chr17	61861500	+
chr17	61872600	+
chr17	61894300	+
chr17	61919700	+
chr17	61926600	+
chr17	61935400	-
chr17	61952900	-
chr17	61968100	-
chr17	61974800	+
chr17	62005400	-
chr17	62012600	-
chr17	62043000	-
chr17	62101400	+
chr17	62507900	-
chr17	62838400	+
chr17	62930500	-
chr17	62946900	+
chr17	63258200	+
chr17	63281300	-
chr17	70872600	+
chr17	70879900	+
chr17	71199100	+
chr17	71929000	+
chr17	71949400	-
chr17	72000400	-
chr17	72900200	-
chr17	72917500	-
chr17	72928100	-
chr17	72934200	-
chr17	72983700	+
chr17	73472400	+
chr17	73490700	-
chr17	73497500	-
chr17	73507000	-
chr17	73535300	-
chr17	73553300	+
chr17	73894300	+
chr17	73921700	-
chr17	74174000	+
chr17	74225900	-
chr17	74235100	+
chr17	74261100	-
chr17	74273000	+
chr17	75858300	-
chr17	76237600	-
chr17	76408900	+
chr17	76460700	+
chr17	76527900	+
chr17	77441100	-
chr17	77791200	+
chr17	77821300	+
chr17	78085700	-
chr17	78110500	+
chr17	78195800	-
chr18	2706800	+
chr18	2957400	-
chr18	3001500	-
chr18	8602400	+
chr18	8614300	+
chr18	9148500	-
chr18	9164500	+
chr18	9909500	+
chr18	9915900	-
chr18	12870200	-
chr18	13411300	+
chr18	13435200	-
chr18	13446400	+
chr18	13465000	-
chr18	13471400	+
chr18	13568700	+
chr18	13580400	+
chr18	16890400	-
chr18	16912600	-
chr18	19293300	+
chr18	19859800	-
chr18	19872700	-
chr18	19877800	+
chr18	19884800	-
chr18	19895900	-
chr18	27868500	-
chr18	27883400	+
chr18	30885000	+
chr18	30893800	-
chr18	30952000	-
chr18	31966700	-
chr18	43679800	-
chr18	43692000	-
chr18	43700200	+
chr18	44704000	-
chr18	44714500	+
chr18	45180200	+
chr18	46821300	-
chr18	46964800	+
chr18	54494000	-
chr18	54499400	+
chr18	54508500	-
chr18	54517400	+
chr18	54965800	-
chr18	58354100	+
chr18	58403200	+
chr18	59001600	-
chr18	59077000	-
chr18	59085900	+
chr18	59095400	-
chr18	59104700	-
chr18	59111100	+
chr18	59117400	-
chr18	59234500	+
chr18	64530500	-
chr18	65703600	-
chr18	70463600	-
chr18	71070200	+
chr18	71110100	-
chr18	71118400	+
chr18	72215700	+
chr18	72700800	+
chr18	72847700	+
chr18	72875800	-
chr18	72885300	+
chr18	72894600	-
chr18	72903900	+
chr18	72909400	+
chr18	72918700	-
chr18	72931500	-
chr18	72940400	-
chr18	72960100	+
chr18	72969600	-
chr18	74955800	+
chr18	74968000	+
chr18	75266100	+
chr18	75367900	-
chr18	75543200	+
chr18	75551500	-
chr18	75773200	-
chr18	75810200	-
chr18	75844000	-
chr19	1165400	+
chr19	1797400	-
chr19	1917600	-
chr19	2277200	-
chr19	2287600	+
chr19	2726500	-
chr19	4065900	-
chr19	4342300	-
chr19	4425000	+
chr19	4929100	+
chr19	4982100	+
chr19	5536900	-
chr19	5577200	+
chr19	5584200	+
chr19	5904500	-
chr19	5910800	-
chr19	5916100	+
chr19	5925400	-
chr19	6191400	+
chr19	6222700	-
chr19	6228700	-
chr19	6742400	+
chr19	7328600	+
chr19	7610300	+
chr19	7813800	-
chr19	7972800	-
chr19	8368100	+
chr19	8423300	+
chr19	8448200	-
chr19	8562800	-
chr19	9818700	+
chr19	10596600	+
chr19	10634500	+
chr19	10702300	+
chr19	10720200	+
chr19	10727600	+
chr19	10734900	+
chr19	10768700	-
chr19	10781900	+
chr19	10868300	-
chr19	10935500	+
chr19	10949400	+
chr19	13091700	+
chr19	13925600	+
chr19	14421900	-
chr19	14508500	+
chr19	15243600	-
chr19	15255400	-
chr19	15269200	-
chr19	15289400	+
chr19	15296500	-
chr19	15377000	-
chr19	16418000	-
chr19	16429800	+
chr19	16509900	+
chr19	16560500	+
chr19	16574200	+
chr19	16588600	+
chr19	17063300	+
chr19	17085100	-
chr19	17284100	+
chr19	17692600	+
chr19	18395000	+
chr19	18413800	-
chr19	18452500	+
chr19	18467600	-
chr19	18479400	+
chr19	18487300	+
chr19	18620200	-
chr19	18689200	+
chr19	19051900	-
chr19	19306300	+
chr19	19383200	+
chr19	19393500	+
chr19	19402000	+
chr19	19434400	+
chr19	19447200	+
chr19	34856500	+
chr19	34863100	-
chr19	35146500	-
chr19	37536900	-
chr19	37855600	-
chr19	38560300	+
chr19	38689000	-
chr19	38697600	-
chr19	39362800	-
chr19	39372100	-
chr19	39500200	-
chr19	40035900	-
chr19	40186800	+
chr19	43851900	-
chr19	43859000	-
chr19	43877100	-
chr19	44339900	-
chr19	44631200	+
chr19	45446200	+
chr19	45460600	+
chr19	45472200	-
chr19	45886400	+
chr19	45953100	+
chr19	46495300	-
chr19	46526300	+
chr19	46542100	-
chr19	46555700	+
chr19	46775000	+
chr19	47381200	+
chr19	47398900	+
chr19	47404600	-
chr19	47447400	-
chr19	50087800	+
chr19	50300400	+
chr19	50312700	-
chr19	50365000	-
chr19	50376600	+
chr19	50449200	+
chr19	50709600	-
chr19	51568700	+
chr19	51923600	+
chr19	51936400	-
chr19	52098100	-
chr19	52120700	+
chr19	52163700	+
chr19	52173400	+
chr19	52436600	-
chr19	52453800	+
chr19	52810300	-
chr19	52828800	-
chr19	52846000	-
chr19	52944900	+
chr19	54070200	+
chr19	54840200	-
chr19	55049700	+
chr19	55198100	-
chr19	55674000	+
chr19	57390100	+
chr19	57401000	-
chr19	59071700	-
chr19	59335400	+
chr19	59404600	-
chr19	59638000	+
chr19	61310200	+
chr19	61315700	+
chr19	63672500	+
chr2	3279500	-
chr2	6982000	+
chr2	6993400	+
chr2	6999800	+
chr2	7065500	+
chr2	9670500	-
chr2	10367800	+
chr2	10376500	+
chr2	10395800	-
chr2	10404200	+
chr2	10426100	+
chr2	10433000	-
chr2	10444600	-
chr2	10458300	-
chr2	10464800	-
chr2	12781800	+
chr2	12793400	+
chr2	20399900	+
chr2	20406800	-
chr2	24394800	+
chr2	24418300	-
chr2	24427300	-
chr2	25382800	+
chr2	25407400	+
chr2	25414500	+
chr2	25454400	+
chr2	25930000	+
chr2	26134300	+
chr2	26144600	-
chr2	26848500	-
chr2	27673600	+
chr2	28487300	+
chr2	28844700	+
chr2	30317100	+
chr2	30324800	+
chr2	30331200	+
chr2	32077600	-
chr2	32455700	-
chr2	37728900	-
chr2	37738200	+
chr2	42260900	-
chr2	42270400	+
chr2	42280400	+
chr2	42299200	-
chr2	42317000	+
chr2	42322200	-
chr2	42422600	-
chr2	44073600	+
chr2	44264200	+
chr2	47044500	-
chr2	47066600	+
chr2	47150900	-
chr2	47950200	+
chr2	54669200	-
chr2	54679300	+
chr2	54687300	+
chr2	54693400	+
chr2	54700900	+
chr2	55103900	+
chr2	55688600	+
chr2	55702200	-
chr2	61260700	+
chr2	61514300	-
chr2	61520000	-
chr2	62282800	+
chr2	62295500	-
chr2	63956100	-
chr2	64718000	-
chr2	64724600	-
chr2	65149000	+
chr2	65201300	-
chr2	68133900	-
chr2	68299900	+
chr2	68319900	+
chr2	68328700	-
chr2	68823300	+
chr2	68878400	+
chr2	68910400	+
chr2	69618500	+
chr2	69636100	+
chr2	69646200	+
chr2	69651900	-
chr2	69658400	+
chr2	69665600	+
chr2	69677700	-
chr2	69700100	+
chr2	71153100	+
chr2	71420600	-
chr2	71429900	-
chr2	74017800	-
chr2	74032000	+
chr2	74079000	-
chr2	74089000	+
chr2	74101300	-
chr2	74121000	-
chr2	74461300	+
chr2	84532300	+
chr2	84910600	+
chr2	85070600	+
chr2	85092000	-
chr2	85954300	-
chr2	85959200	-
chr2	86525300	+
chr2	86827400	+
chr2	88696100	+
chr2	95064100	+
chr2	96576300	+
chr2	96624600	+
chr2	96849100	+
chr2	96869000	+
chr2	96895900	-
chr2	97710600	-
chr2	97725000	-
chr2	97952000	+
chr2	98438800	+
chr2	98460500	-
chr2	98497100	+
chr2	98530500	-
chr2	98673400	+
chr2	98685800	+
chr2	98695600	+
chr2	101283100	+
chr2	101699600	-
chr2	101716700	+
chr2	101727800	+
chr2	101735400	+
chr2	101745600	+
chr2	101755200	-
chr2	101764900	+
chr2	101785000	+
chr2	101799600	+
chr2	101813000	-
chr2	101825900	+
chr2	105291700	+
chr2	105736200	-
chr2	105743300	-
chr2	105750300	+
chr2	105759900	+
chr2	105773400	+
chr2	105784000	-
chr2	105796700	+
chr2	105807100	+
chr2	105822800	-
chr2	105832100	-
chr2	105847000	+
chr2	105852100	-
chr2	105860100	+
chr2	105878400	-
chr2	105885100	+
chr2	106098200	+
chr2	106158900	-
chr2	108447200	+
chr2	108942800	+
chr2	112726000	+
chr2	113656400	+
chr2	114374600	+
chr2	120259800	-
chr2	120269900	-
chr2	120736200	-
chr2	120752200	+
chr2	121954300	-
chr2	122020900	-
chr2	122041300	-
chr2	122091900	+
chr2	127139300	+
chr2	127152500	-
chr2	127158200	-
chr2	127854600	+
chr2	127989200	-
chr2	128251300	+
chr2	128354300	+
chr2	128746100	-
chr2	128766800	+
chr2	130853600	-
chr2	131587200	-
chr2	134600900	-
chr2	134615600	-
chr2	134624500	+
chr2	134638200	-
chr2	134648300	+
chr2	134659800	-
chr2	134665700	-
chr2	134672600	+
chr2	134718900	+
chr2	134732500	-
chr2	134756100	-
chr2	134763100	-
chr2	134841500	+
chr2	134885300	-
chr2	135400900	+
chr2	135408000	-
chr2	143615500	+
chr2	143626900	+
chr2	143646100	+
chr2	143657800	-
chr2	143666900	-
chr2	143736800	+
chr2	143750100	+
chr2	143763400	-
chr2	143795800	+
chr2	143806500	-
chr2	143891300	-
chr2	149132700	+
chr2	149149200	+
chr2	149193700	-
chr2	153273500	-
chr2	160287100	+
chr2	160302800	-
chr2	160895800	-
chr2	160904100	-
chr2	160914700	-
chr2	160918900	-
chr2	160936900	+
chr2	160945900	-
chr2	160952800	-
chr2	160962600	+
chr2	160968500	-
chr2	161025600	+
chr2	168747700	-
chr2	168757700	-
chr2	168777200	-
chr2	171505700	-
chr2	171715900	+
chr2	173011500	+
chr2	173029000	-
chr2	174494900	-
chr2	174502100	-
chr2	174513700	+
chr2	175162500	-
chr2	175191000	+
chr2	177813900	+
chr2	177830700	+
chr2	180564500	+
chr2	182045200	+
chr2	191009900	-
chr2	191030200	+
chr2	191036300	+
chr2	191717100	-
chr2	197695000	+
chr2	197709600	-
chr2	197725700	+
chr2	197735600	-
chr2	197748900	-
chr2	197762300	-
chr2	197804900	-
chr2	197819900	-
chr2	197841300	+
chr2	197853200	+
chr2	201840300	+
chr2	204288700	-
chr2	204518100	-
chr2	207729000	-
chr2	216754600	-
chr2	218856000	-
chr2	219150500	-
chr2	224503100	-
chr2	224533600	+
chr2	225100700	-
chr2	225112700	-
chr2	225127200	+
chr2	225138300	-
chr2	225148700	+
chr2	225483900	+
chr2	225501300	+
chr2	225531400	+
chr2	225543600	-
chr2	225560800	+
chr2	225566800	+
chr2	225574700	-
chr2	225581900	+
chr2	225593900	+
chr2	225604300	+
chr2	228077300	+
chr2	228089600	+
chr2	230472300	-
chr2	230789900	-
chr2	230920800	+
chr2	230996800	-
chr2	231022300	-
chr2	231031300	-
chr2	231291100	+
chr2	231307100	-
chr2	231324700	+
chr2	231331900	-
chr2	231352100	-
chr2	231363600	-
chr2	231478900	+
chr2	232865300	-
chr2	233117400	+
chr2	233294600	+
chr2	233653500	-
chr2	233664800	-
chr2	233673400	-
chr2	233680000	-
chr2	233691400	+
chr2	233737900	-
chr2	233960700	+
chr2	234002100	+
chr2	238298100	-
chr2	238306000	-
chr2	238551600	-
chr2	239704300	+
chr2	239791000	-
chr2	239802700	-
chr2	239826000	-
chr2	239833700	-
chr2	239839300	-
chr2	239847300	+
chr2	239862300	+
chr2	239926100	-
chr2	239933100	-
chr2	239957400	+
chr2	241146800	-
chr2	241177000	+
chr2	241877400	-
chr2	241891700	+
chr2	241896900	-
chr2	242093500	-
chr2	242227900	+
chr2	242291700	+
chr2	242478200	-
chr20	364000	+
chr20	445000	-
chr20	1222700	+
chr20	1306700	+
chr20	1574100	-
chr20	2039700	-
chr20	2048600	+
chr20	2054800	+
chr20	2066300	-
chr20	2818900	+
chr20	3077300	-
chr20	3729200	+
chr20	4626000	-
chr20	5075500	+
chr20	5084900	-
chr20	5098100	+
chr20	20613500	+
chr20	21243300	-
chr20	21253200	-
chr20	24909100	+
chr20	24915200	+
chr20	24953600	+
chr20	24971300	-
chr20	25187000	-
chr20	29588200	-
chr20	29751300	-
chr20	29758000	+
chr20	30255600	+
chr20	30416900	+
chr20	30458500	+
chr20	30522800	+
chr20	30528900	-
chr20	30546000	-
chr20	30568800	+
chr20	30599000	+
chr20	30606900	+
chr20	30619800	+
chr20	30633100	-
chr20	31866400	+
chr20	31877500	-
chr20	31889700	+
chr20	31898200	+
chr20	32060800	+
chr20	32068700	-
chr20	32076300	-
chr20	32089400	+
chr20	32101900	-
chr20	32110500	+
chr20	32121100	+
chr20	33696100	+
chr20	33705800	-
chr20	33957800	-
chr20	34529800	+
chr20	34542400	+
chr20	34554000	-
chr20	35256600	-
chr20	35358100	-
chr20	35371300	+
chr20	35586700	-
chr20	36085900	-
chr20	36545800	+
chr20	36874900	-
chr20	36891300	-
chr20	36917800	-
chr20	36924900	-
chr20	36934100	-
chr20	36949100	+
chr20	36956100	+
chr20	39105200	+
chr20	39134800	+
chr20	39629100	-
chr20	39635000	+
chr20	39669700	+
chr20	42956900	-
chr20	42972100	-
chr20	43111400	-
chr20	43130600	-
chr20	44420900	+
chr20	44464100	+
chr20	45380100	+
chr20	45392900	-
chr20	45409000	+
chr20	46760300	+
chr20	46766400	+
chr20	46788900	+
chr20	46801500	+
chr20	46827100	-
chr20	46832800	-
chr20	46840800	-
chr20	46851100	+
chr20	46858800	-
chr20	46982500	-
chr20	47104100	-
chr20	47219000	-
chr20	47320900	-
chr20	47873000	-
chr20	48135000	-
chr20	48196500	-
chr20	48243500	+
chr20	48564700	-
chr20	48572300	+
chr20	48576800	+
chr20	48596100	-
chr20	48610800	+
chr20	48619900	+
chr20	48960400	+
chr20	48965300	-
chr20	49481900	+
chr20	49488600	-
chr20	49495100	+
chr20	49514300	+
chr20	49530800	-
chr20	49543900	-
chr20	49563400	-
chr20	49576200	+
chr20	49582200	+
chr20	54448300	-
chr20	54493100	+
chr20	56324900	-
chr20	56410000	-
chr20	56425500	+
chr20	56441400	-
chr20	57026300	+
chr20	57191400	+
chr20	60028600	+
chr20	60061800	+
chr20	60071900	-
chr20	60133100	+
chr20	60166000	+
chr20	60254900	-
chr20	60414100	-
chr20	61001900	+
chr20	61016300	-
chr20	61037000	-
chr20	61304100	+
chr20	61314700	+
chr20	61757000	+
chr20	61999100	+
chr20	62015700	+
chr20	62069900	-
chr21	14817700	+
chr21	16037300	+
chr21	16048300	+
chr21	29612800	-
chr21	31426400	-
chr21	33530300	-
chr21	33539600	+
chr21	33625400	-
chr21	34380800	-
chr21	34390500	-
chr21	34426400	+
chr21	35103000	-
chr21	35110600	-
chr21	35129900	+
chr21	35139300	+
chr21	35160200	+
chr21	35209000	+
chr21	35217900	-
chr21	35227200	-
chr21	35241200	+
chr21	35259700	+
chr21	35278600	+
chr21	35292900	-
chr21	35300300	+
chr21	35309400	-
chr21	35315400	-
chr21	35329000	-
chr21	37378100	+
chr21	37538800	-
chr21	37667700	+
chr21	37672700	-
chr21	37680700	-
chr21	37691800	+
chr21	37703300	+
chr21	37724700	-
chr21	37738800	-
chr21	37744400	+
chr21	37757300	+
chr21	37765400	+
chr21	37770600	+
chr21	41667600	+
chr21	42521300	-
chr21	42530600	-
chr21	42545200	+
chr21	42561800	-
chr21	42717100	-
chr21	42732600	-
chr21	43910800	-
chr21	44123900	-
chr21	44166700	+
chr21	44203400	+
chr21	44269400	+
chr21	44293200	-
chr21	44310300	+
chr21	44380200	+
chr21	44545600	+
chr21	45061000	-
chr21	45098900	+
chr21	45110400	+
chr21	45211300	+
chr21	45323300	+
chr21	45331700	+
chr21	45339700	+
chr21	45399700	+
chr21	46499700	+
chr21	46616800	+
chr21	46717700	+
chr21	46893100	+
chr22	15959300	-
chr22	15979800	+
chr22	16615200	+
chr22	16643200	-
chr22	17456000	-
chr22	17466200	-
chr22	17473800	-
chr22	17791000	+
chr22	18213200	+
chr22	18399800	+
chr22	18451200	+
chr22	18467500	-
chr22	18503800	-
chr22	19173900	-
chr22	19207300	-
chr22	19215500	+
chr22	19253300	-
chr22	19549800	-
chr22	19624800	-
chr22	19630800	+
chr22	20257800	-
chr22	20276900	+
chr22	20448400	+
chr22	20502300	+
chr22	20529200	+
chr22	20545300	-
chr22	21864400	-
chr22	21876900	-
chr22	21892000	-
chr22	21914600	-
chr22	21922900	+
chr22	21928300	+
chr22	22871000	-
chr22	23029900	+
chr22	23133900	-
chr22	23160100	-
chr22	23290500	-
chr22	24109100	+
chr22	24115600	+
chr22	25269400	+
chr22	25281900	+
chr22	25290400	-
chr22	25298000	-
chr22	25306100	+
chr22	25416300	-
chr22	25436900	-
chr22	28107300	+
chr22	28347400	-
chr22	29036600	+
chr22	29045000	+
chr22	29071500	-
chr22	29619500	-
chr22	29904900	-
chr22	29949800	-
chr22	29973800	-
chr22	30061300	-
chr22	30678000	+
chr22	34041500	-
chr22	34876100	-
chr22	35082800	-
chr22	35094000	-
chr22	36018400	-
chr22	36034700	+
chr22	36901700	-
chr22	37030200	-
chr22	37040700	-
chr22	37595200	-
chr22	38141100	+
chr22	38639900	-
chr22	38663500	+
chr22	38673700	+
chr22	38684700	-
chr22	38781400	+
chr22	38808100	+
chr22	38821500	+
chr22	38831100	+
chr22	38837600	+
chr22	38842500	-
chr22	38919600	+
chr22	38929600	-
chr22	38937600	-
chr22	38945500	+
chr22	38964100	+
chr22	38975000	-
chr22	38991900	-
chr22	39128300	+
chr22	39176800	-
chr22	39828400	+
chr22	39841800	+
chr22	40034200	+
chr22	40207700	-
chr22	40572800	-
chr22	40634600	-
chr22	40898700	+
chr22	40911900	+
chr22	40921000	+
chr22	40928200	-
chr22	40944300	+
chr22	40954200	-
chr22	40975000	-
chr22	40989400	-
chr22	41020800	+
chr22	41051500	+
chr22	41329900	-
chr22	41622000	+
chr22	41672500	+
chr22	41727300	-
chr22	42920600	-
chr22	43459400	-
chr22	43485700	+
chr22	44097300	+
chr22	44456600	+
chr22	44464500	+
chr22	44849900	+
chr22	45434700	-
chr22	45500300	-
chr22	45552500	+
chr22	45578000	-
chr22	45603400	+
chr22	45618200	-
chr22	45628700	-
chr22	45681700	+
chr22	45745300	+
chr22	45779200	+
chr22	45794300	-
chr22	45818100	-
chr22	45842900	+
chr22	45866100	-
chr22	48380100	-
chr22	48420100	+
chr22	48426700	-
chr22	48569200	+
chr22	48596800	-
chr22	48635800	+
chr22	48648300	+
chr22	49132800	+
chr22	49147500	-
chr3	3151400	-
chr3	5158700	+
chr3	5165700	-
chr3	5178800	+
chr3	5183300	-
chr3	9670900	+
chr3	9755800	-
chr3	10286300	+
chr3	10301000	-
chr3	11599400	+
chr3	11606000	+
chr3	11629600	-
chr3	11651300	+
chr3	12646700	-
chr3	12672900	+
chr3	12933400	+
chr3	12940900	+
chr3	12952400	-
chr3	12965800	-
chr3	12980800	-
chr3	12990000	-
chr3	13000100	+
chr3	13007500	-
chr3	13025800	-
chr3	13404800	+
chr3	13433000	+
chr3	14458300	-
chr3	15008400	-
chr3	15285900	-
chr3	15293500	+
chr3	15301100	+
chr3	15307900	-
chr3	15317500	-
chr3	15331700	+
chr3	15336400	-
chr3	16322400	+
chr3	16333600	-
chr3	16355100	+
chr3	16391100	-
chr3	16402900	+
chr3	16428300	+
chr3	16512600	-
chr3	16917900	+
chr3	16933300	-
chr3	16944200	-
chr3	16957500	+
chr3	16977000	+
chr3	16991600	+
chr3	17009000	-
chr3	17020700	+
chr3	17046200	+
chr3	18390100	-
chr3	20071700	+
chr3	20081700	-
chr3	23842400	+
chr3	23876700	+
chr3	23895500	-
chr3	30637700	-
chr3	30658300	+
chr3	30663800	+
chr3	30675900	+
chr3	30698900	+
chr3	31579500	+
chr3	31591200	-
chr3	32154200	-
chr3	32421900	+
chr3	32437900	+
chr3	32488000	+
chr3	32509900	-
chr3	33446900	-
chr3	33824000	+
chr3	38020600	-
chr3	38189800	-
chr3	38221600	-
chr3	38491600	+
chr3	39402700	+
chr3	41228600	+
chr3	42656400	+
chr3	43328300	+
chr3	43336800	+
chr3	43344500	+
chr3	44979900	-
chr3	44986300	+
chr3	44999000	+
chr3	45007900	-
chr3	45616800	+
chr3	45627600	-
chr3	45982900	+
chr3	46009400	-
chr3	46083200	-
chr3	46948200	-
chr3	46962000	-
chr3	46970000	-
chr3	46976500	+
chr3	46987600	-
chr3	47002500	-
chr3	47035300	-
chr3	47168500	-
chr3	47312500	-
chr3	47411500	-
chr3	48486100	-
chr3	49428900	+
chr3	49532100	-
chr3	50636300	-
chr3	50642800	-
chr3	50651200	+
chr3	51686700	-
chr3	51696100	+
chr3	51707500	+
chr3	51714400	-
chr3	52060200	-
chr3	52316200	+
chr3	52884800	+
chr3	53179600	-
chr3	53253100	-
chr3	56621900	+
chr3	56771500	-
chr3	56787500	-
chr3	56797300	-
chr3	56821100	-
chr3	56839700	+
chr3	56870500	+
chr3	56895900	-
chr3	56911600	-
chr3	57640900	+
chr3	63838000	-
chr3	63895000	-
chr3	63903600	-
chr3	63941000	+
chr3	66565800	-
chr3	66573500	-
chr3	66607300	+
chr3	66616900	+
chr3	67729800	-
chr3	67763500	-
chr3	67771800	+
chr3	71123300	+
chr3	71142600	-
chr3	71159000	+
chr3	71166300	-
chr3	71182800	+
chr3	71210700	-
chr3	71220200	+
chr3	71232800	+
chr3	71243000	+
chr3	71251900	+
chr3	71346600	+
chr3	71380000	+
chr3	71389700	-
chr3	71397400	-
chr3	71405400	+
chr3	71564600	+
chr3	71793900	+
chr3	71833400	-
chr3	71841700	-
chr3	72514000	-
chr3	72532200	+
chr3	72576300	-
chr3	73140600	+
chr3	73150800	+
chr3	73161100	+
chr3	102588600	-
chr3	102626000	-
chr3	109273900	+
chr3	109286300	-
chr3	110048100	+
chr3	112812700	+
chr3	112818000	-
chr3	114134000	+
chr3	114762300	+
chr3	114808800	+
chr3	121224700	+
chr3	121272700	+
chr3	121544800	-
chr3	126240500	-
chr3	126505600	+
chr3	126568100	-
chr3	127265000	-
chr3	127673700	-
chr3	128112900	-
chr3	128775700	-
chr3	128781900	-
chr3	129936100	-
chr3	129950600	+
chr3	129961600	+
chr3	129968400	-
chr3	129984000	+
chr3	130298100	+
chr3	130308500	+
chr3	137375900	+
chr3	137391700	-
chr3	138095300	-
chr3	138108600	-
chr3	139405500	+
chr3	139979500	-
chr3	142627900	+
chr3	142706300	-
chr3	142753600	-
chr3	151810200	+
chr3	151942600	-
chr3	151952700	-
chr3	153462100	+
chr3	153499800	+
chr3	153508500	-
chr3	153535100	-
chr3	153543900	+
chr3	153551300	+
chr3	153584200	+
chr3	153603400	-
chr3	155520400	+
chr3	157890800	-
chr3	161755300	-
chr3	168928700	-
chr3	172431200	+
chr3	172515000	+
chr3	172547800	-
chr3	172561400	+
chr3	172573900	-
chr3	172582900	-
chr3	172589700	+
chr3	172615100	-
chr3	172629900	-
chr3	172638300	-
chr3	178302800	-
chr3	178311200	+
chr3	178333400	+
chr3	178342700	+
chr3	178351100	-
chr3	178371000	+
chr3	178382900	-
chr3	180360700	-
chr3	180385200	+
chr3	182124400	+
chr3	184718600	+
chr3	184734200	-
chr3	184742000	-
chr3	185517000	+
chr3	187131000	-
chr3	188139600	-
chr3	188149000	+
chr3	188161400	+
chr3	188173900	-
chr3	188190700	+
chr3	188197900	+
chr3	188216600	-
chr3	188240100	-
chr3	188246300	-
chr3	188264500	+
chr3	189364400	-
chr3	189379300	+
chr3	196620000	-
chr3	196628700	-
chr3	196743000	-
chr3	197111900	+
chr3	197634000	-
chr3	197986300	+
chr4	697500	+
chr4	899800	-
chr4	907100	+
chr4	955300	-
chr4	1315400	+
chr4	2261600	-
chr4	2269200	+
chr4	2335400	-
chr4	2458100	+
chr4	2542600	+
chr4	2559800	-
chr4	2565000	+
chr4	2592700	-
chr4	2623000	-
chr4	2686000	+
chr4	2718100	-
chr4	2791900	+
chr4	2833200	-
chr4	2908900	+
chr4	3168100	-
chr4	6846700	+
chr4	6978800	-
chr4	6996000	+
chr4	7015500	-
chr4	8281100	-
chr4	9710400	-
chr4	15817700	+
chr4	15827700	-
chr4	17228000	+
chr4	24168600	+
chr4	24181500	+
chr4	25453700	+
chr4	26484100	-
chr4	26522900	+
chr4	37344700	-
chr4	38350200	+
chr4	38362800	-
chr4	39398300	-
chr4	39620300	-
chr4	39642500	-
chr4	39890500	-
chr4	39896500	+
chr4	39910000	+
chr4	42325700	+
chr4	42338800	-
chr4	47820300	-
chr4	48332200	-
chr4	48439300	+
chr4	48446100	+
chr4	48464000	-
chr4	54618100	-
chr4	68894800	-
chr4	74329500	-
chr4	77339700	+
chr4	78916800	-
chr4	78934000	+
chr4	83577400	-
chr4	88174600	+
chr4	88179600	+
chr4	88185300	+
chr4	88199500	-
chr4	89739100	-
chr4	90388400	+
chr4	90397300	+
chr4	90418400	+
chr4	90423700	-
chr4	99750200	-
chr4	100048000	+
chr4	100154900	+
chr4	100163700	+
chr4	103649000	+
chr4	103659600	+
chr4	103950800	-
chr4	103958200	-
chr4	109212400	-
chr4	109234600	-
chr4	109243800	+
chr4	109252100	-
chr4	109275500	-
chr4	109293000	-
chr4	110588700	+
chr4	110603600	+
chr4	110715800	+
chr4	110729800	+
chr4	110758500	-
chr4	122829900	-
chr4	129967000	+
chr4	129981400	+
chr4	130003400	-
chr4	143353700	+
chr4	143406300	+
chr4	143454400	-
chr4	143465800	+
chr4	143509000	+
chr4	149576400	+
chr4	152141500	-
chr4	154654400	+
chr4	154671400	+
chr4	154676500	+
chr4	154682500	-
chr4	154755900	-
chr4	185543000	+
chr4	185590200	+
chr4	185596700	+
chr4	185606800	-
chr4	185615500	-
chr4	185799300	+
chr5	518000	+
chr5	1389000	-
chr5	1559500	+
chr5	1569400	+
chr5	6780100	+
chr5	7933500	-
chr5	10427100	-
chr5	10758700	+
chr5	10765300	-
chr5	10807000	-
chr5	14729200	-
chr5	14847300	-
chr5	14855100	-
chr5	14867200	+
chr5	14908700	+
chr5	16578200	+
chr5	16590000	+
chr5	16634100	+
chr5	16650500	+
chr5	16657300	-
chr5	16661900	+
chr5	32181400	+
chr5	32194600	-
chr5	32334600	+
chr5	32457300	-
chr5	36921700	-
chr5	36970900	+
chr5	39058700	+
chr5	39084000	-
chr5	40818200	-
chr5	40827500	-
chr5	43190600	+
chr5	50022200	-
chr5	50058300	-
chr5	53873800	+
chr5	56158200	-
chr5	56167600	+
chr5	56174500	-
chr5	56181700	+
chr5	56518600	+
chr5	56539700	+
chr5	61650600	-
chr5	61658000	+
chr5	61665500	+
chr5	61673800	+
chr5	65267100	-
chr5	65284600	-
chr5	65292600	+
chr5	65299000	-
chr5	65315500	-
chr5	67562700	+
chr5	67574600	-
chr5	67582200	+
chr5	67590600	+
chr5	67597200	-
chr5	74365500	-
chr5	75757200	+
chr5	75780100	+
chr5	75790200	+
chr5	75804600	+
chr5	75811000	+
chr5	75829700	+
chr5	75836500	+
chr5	75859200	+
chr5	75870600	+
chr5	75887700	+
chr5	78591600	-
chr5	79534600	-
chr5	79542700	-
chr5	79550300	-
chr5	79562000	+
chr5	79570700	+
chr5	82401000	-
chr5	86631100	+
chr5	87582100	-
chr5	96079700	+
chr5	96165800	+
chr5	96324700	+
chr5	108751400	-
chr5	109068300	+
chr5	109075700	+
chr5	109104200	+
chr5	110599900	-
chr5	110606300	+
chr5	110618500	+
chr5	110628100	+
chr5	110673600	+
chr5	110738800	-
chr5	112348000	+
chr5	118646600	+
chr5	118685900	-
chr5	118710500	+
chr5	118723300	+
chr5	118730700	+
chr5	118742200	+
chr5	118748500	-
chr5	118758400	-
chr5	130679400	+
chr5	130690700	-
chr5	130728400	+
chr5	130890400	-
chr5	130904700	-
chr5	130973000	+
chr5	131759500	-
chr5	131790900	+
chr5	131799600	-
chr5	131810200	-
chr5	131830600	+
chr5	132307400	+
chr5	132425800	-
chr5	133364200	+
chr5	133896900	+
chr5	133908300	+
chr5	134736900	-
chr5	134757900	-
chr5	137387500	+
chr5	137733100	+
chr5	137894300	-
chr5	138662300	+
chr5	138671100	-
chr5	138835800	+
chr5	138938400	+
chr5	138996200	-
chr5	139492000	+
chr5	139784100	+
chr5	140958100	+
chr5	141481100	+
chr5	141488700	+
chr5	142710000	-
chr5	142729000	+
chr5	142737100	+
chr5	142743200	+
chr5	142748800	+
chr5	148726700	-
chr5	148879100	+
chr5	148888800	+
chr5	148896500	-
chr5	150428000	-
chr5	150437000	-
chr5	150454200	-
chr5	153404900	+
chr5	154126300	-
chr5	154133500	-
chr5	154148000	+
chr5	156585200	-
chr5	156630400	-
chr5	156643300	+
chr5	156648800	+
chr5	156704300	-
chr5	169002100	+
chr5	169011200	+
chr5	169026800	+
chr5	169261300	-
chr5	169629200	+
chr5	171317800	+
chr5	171424800	-
chr5	171465800	-
chr5	171493000	-
chr5	171504100	+
chr5	171523200	+
chr5	171529800	-
chr5	172212800	+
chr5	172220400	-
chr5	172363000	-
chr5	172370700	+
chr5	172375500	-
chr5	172388600	-
chr5	174849100	-
chr5	175023900	+
chr5	175031100	-
chr5	175044900	+
chr5	175715300	-
chr5	175773800	-
chr5	176018200	-
chr5	176395900	-
chr5	176671800	-
chr5	177583500	+
chr5	179114300	-
chr5	179122300	+
chr5	179163700	-
chr5	179183600	-
chr5	180158600	-
chr6	629600	-
chr6	2719800	-
chr6	3206900	+
chr6	3972700	+
chr6	4851500	-
chr6	4878100	-
chr6	7060700	-
chr6	7073700	+
chr6	7091300	-
chr6	7099000	-
chr6	7110300	+
chr6	7116700	+
chr6	7147700	+
chr6	11320000	+
chr6	11428400	+
chr6	11438300	-
chr6	11453400	-
chr6	11461900	-
chr6	11475700	+
chr6	11482900	+
chr6	12220900	-
chr6	13811300	-
chr6	15366800	-
chr6	15374100	+
chr6	15402200	+
chr6	15412900	-
chr6	15425700	-
chr6	15436000	-
chr6	15473700	+
chr6	15483500	+
chr6	15498900	+
chr6	15512600	+
chr6	15518500	+
chr6	15530800	+
chr6	15546800	-
chr6	15554700	+
chr6	15560400	+
chr6	15570100	-
chr6	15589300	-
chr6	16245400	+
chr6	16857000	+
chr6	25002000	-
chr6	25010100	+
chr6	25021100	+
chr6	25117200	-
chr6	26075100	+
chr6	26514100	+
chr6	30382600	+
chr6	30391900	-
chr6	31634700	-
chr6	33507700	+
chr6	33709600	-
chr6	33772800	+
chr6	33862000	-
chr6	34668600	+
chr6	34674800	-
chr6	35436300	-
chr6	35446800	+
chr6	35458100	-
chr6	35469100	-
chr6	35479500	-
chr6	35487500	+
chr6	36113800	-
chr6	36125800	-
chr6	36277200	+
chr6	36595400	+
chr6	36604700	-
chr6	37906900	-
chr6	37953500	-
chr6	38131400	+
chr6	41783600	+
chr6	42028700	-
chr6	42039800	+
chr6	42050900	+
chr6	42086200	+
chr6	42097600	-
chr6	42106600	+
chr6	42355200	-
chr6	42371200	+
chr6	42379300	+
chr6	42391100	+
chr6	42411400	+
chr6	42456400	+
chr6	42479100	-
chr6	42493900	+
chr6	42504600	+
chr6	43063300	+
chr6	43092900	+
chr6	43570800	-
chr6	44196300	+
chr6	44491700	+
chr6	45571200	-
chr6	47578000	+
chr6	52510500	+
chr6	53021400	-
chr6	53273900	+
chr6	53282400	-
chr6	53299300	-
chr6	53317900	+
chr6	64429000	+
chr6	64443400	+
chr6	71439000	-
chr6	71467200	-
chr6	76043500	-
chr6	76387100	+
chr6	87929900	-
chr6	87941200	+
chr6	87947400	+
chr6	87967100	+
chr6	87978400	-
chr6	88341200	-
chr6	88452200	+
chr6	88457700	-
chr6	89675400	-
chr6	90560200	-
chr6	90718300	-
chr6	90752400	+
chr6	90795700	-
chr6	90811200	+
chr6	90833900	+
chr6	90862700	-
chr6	107088200	-
chr6	108140800	+
chr6	108148700	-
chr6	108213000	-
chr6	108234500	+
chr6	108376900	+
chr6	109008500	-
chr6	109015100	-
chr6	109064500	+
chr6	109080600	+
chr6	109101800	+
chr6	109802100	+
chr6	111310700	+
chr6	111994500	-
chr6	112111800	+
chr6	112151700	-
chr6	112166800	+
chr6	112222200	+
chr6	112233500	+
chr6	112239100	+
chr6	112246500	+
chr6	112257300	-
chr6	112266700	+
chr6	112282300	+
chr6	112289600	+
chr6	112297000	-
chr6	118011500	+
chr6	118108000	+
chr6	119052200	-
chr6	119072600	-
chr6	119083900	-
chr6	119125300	-
chr6	119261900	-
chr6	128154700	-
chr6	128175800	+
chr6	128232300	-
chr6	132854100	-
chr6	135410700	+
chr6	137102200	+
chr6	137116600	+
chr6	137147700	-
chr6	139335600	+
chr6	139512000	-
chr6	139526700	+
chr6	139536600	-
chr6	142526600	-
chr6	143136700	-
chr6	143171700	+
chr6	143185200	+
chr6	143234600	-
chr6	143243200	+
chr6	143255400	+
chr6	143262500	-
chr6	143911900	-
chr6	144726800	-
chr6	144741700	-
chr6	144771100	+
chr6	144779000	+
chr6	145025100	-
chr6	149691600	-
chr6	149705300	+
chr6	149731000	+
chr6	149834700	-
chr6	150073900	-
chr6	151761600	-
chr6	154529200	+
chr6	154560900	+
chr6	154600200	+
chr6	155112200	-
chr6	155131600	+
chr6	157160700	-
chr6	157169900	+
chr6	157183100	-
chr6	157189900	+
chr6	157210200	+
chr6	157225000	-
chr6	157235000	+
chr6	157241500	-
chr6	157273100	+
chr6	157291800	+
chr6	157319300	-
chr6	157345100	+
chr6	157351400	+
chr6	157357800	+
chr6	157510000	+
chr6	158331100	-
chr6	158349900	-
chr6	158389800	+
chr6	158914100	+
chr6	158995600	+
chr6	159113300	+
chr6	159137000	-
chr6	159143400	-
chr6	159154400	-
chr6	160338400	+
chr6	160346300	+
chr6	160410200	+
chr6	166672900	+
chr6	167263600	-
chr6	167452900	-
chr6	170465500	+
chr7	829600	-
chr7	837900	-
chr7	885200	+
chr7	1014900	+
chr7	1062300	+
chr7	1864800	-
chr7	1905200	-
chr7	1957100	-
chr7	2091600	+
chr7	2105300	-
chr7	2125600	-
chr7	2959900	-
chr7	2977700	-
chr7	2984700	-
chr7	3014800	+
chr7	3033700	-
chr7	3047400	-
chr7	4696400	+
chr7	4702700	-
chr7	4708800	-
chr7	4717800	-
chr7	4723300	+
chr7	4739600	-
chr7	5388500	-
chr7	5637500	-
chr7	5650000	+
chr7	5707200	-
chr7	5784200	+
chr7	6391000	+
chr7	6400600	+
chr7	6482500	-
chr7	21436800	+
chr7	24919800	+
chr7	24930300	-
chr7	24971900	-
chr7	24977500	-
chr7	30476500	-
chr7	32518200	-
chr7	35657100	+
chr7	35686700	-
chr7	35830700	+
chr7	36992800	-
chr7	37070900	+
chr7	37107400	+
chr7	37271200	-
chr7	37282900	-
chr7	37291900	-
chr7	37316200	-
chr7	37325200	+
chr7	37338200	-
chr7	37369700	+
chr7	37392900	-
chr7	37398600	+
chr7	37426800	+
chr7	37439500	+
chr7	38909100	+
chr7	39975300	+
chr7	43611600	-
chr7	43618900	+
chr7	43649800	+
chr7	44056500	+
chr7	44423700	+
chr7	44439600	+
chr7	44468000	+
chr7	44477200	-
chr7	44761200	+
chr7	44965700	-
chr7	45012800	-
chr7	45043200	+
chr7	45060100	+
chr7	45067600	-
chr7	50333700	+
chr7	50398000	-
chr7	50408900	-
chr7	50419200	-
chr7	50428900	-
chr7	50441200	-
chr7	55511600	+
chr7	55520500	+
chr7	55536700	-
chr7	55550700	-
chr7	55557700	+
chr7	55572700	+
chr7	55583800	+
chr7	55597000	-
chr7	56005900	-
chr7	64484900	+
chr7	66032300	-
chr7	73235800	-
chr7	75396400	+
chr7	75804800	+
chr7	75813500	-
chr7	77185700	-
chr7	77195400	+
chr7	77219800	+
chr7	86668100	-
chr7	86679500	-
chr7	96169100	+
chr7	97670700	+
chr7	97711800	+
chr7	97752000	+
chr7	98913600	-
chr7	100017000	-
chr7	100128100	-
chr7	100652100	+
chr7	104431500	+
chr7	104633400	+
chr7	104666300	+
chr7	105234300	-
chr7	114376600	-
chr7	114382300	-
chr7	114398000	+
chr7	115650400	-
chr7	115657700	-
chr7	115665500	-
chr7	116301900	+
chr7	116313200	-
chr7	126808300	+
chr7	127094800	+
chr7	127178500	-
chr7	127431900	+
chr7	127480100	+
chr7	127487300	+
chr7	127497100	-
chr7	128451300	+
chr7	128456700	+
chr7	128468800	-
chr7	129054100	+
chr7	129271200	+
chr7	129289300	-
chr7	130217300	-
chr7	130223500	-
chr7	130230800	+
chr7	130239500	-
chr7	130245800	-
chr7	130254200	+
chr7	130262200	+
chr7	130271500	+
chr7	130287000	+
chr7	130321700	-
chr7	130331300	+
chr7	130348400	+
chr7	130356200	-
chr7	130363300	+
chr7	130390900	-
chr7	130408900	-
chr7	130414800	+
chr7	130427200	+
chr7	134827300	+
chr7	135274600	-
chr7	135292800	-
chr7	135301200	-
chr7	138705600	-
chr7	138730000	+
chr7	139017500	-
chr7	139383600	+
chr7	139395000	-
chr7	139737400	+
chr7	141043500	-
chr7	142792600	-
chr7	148035700	-
chr7	148042600	-
chr7	148049800	-
chr7	149183800	+
chr7	150800500	-
chr7	150825300	-
chr7	150838000	+
chr7	151037100	-
chr7	151083500	-
chr7	151123900	+
chr7	151161300	+
chr7	151168000	+
chr7	151179300	-
chr7	151187100	-
chr7	151199200	-
chr7	151753900	-
chr7	155139100	-
chr7	155148100	+
chr7	155155300	-
chr7	155169700	-
chr7	155178100	+
chr7	155198900	-
chr7	155218800	-
chr7	155238800	-
chr7	155249100	-
chr7	156637100	-
chr7	156834700	+
chr7	156841100	-
chr7	156860600	-
chr7	156876100	+
chr7	156885000	-
chr7	158290500	+
chr7	158297400	-
chr8	1704800	+
chr8	1711600	-
chr8	6274200	+
chr8	8225400	-
chr8	9508900	+
chr8	11707100	+
chr8	11748300	-
chr8	11756500	+
chr8	19598800	+
chr8	21874300	+
chr8	22367000	-
chr8	22555800	-
chr8	23074000	-
chr8	23113000	+
chr8	23134200	-
chr8	26209300	+
chr8	26228300	-
chr8	26235000	-
chr8	26250000	-
chr8	26265200	-
chr8	27200600	+
chr8	27212700	-
chr8	27221900	-
chr8	27253200	-
chr8	27261900	-
chr8	27288700	-
chr8	27306300	-
chr8	27318700	-
chr8	27326500	-
chr8	27332800	-
chr8	30052800	-
chr8	37858200	+
chr8	38767100	+
chr8	38774000	-
chr8	38782300	-
chr8	38881100	+
chr8	38915500	+
chr8	41564700	-
chr8	41581800	+
chr8	41934400	+
chr8	41942200	+
chr8	41961100	+
chr8	41987600	+
chr8	41991800	-
chr8	42011200	+
chr8	42257800	+
chr8	42262900	+
chr8	42269700	+
chr8	42276400	-
chr8	43074300	-
chr8	52947900	-
chr8	53763200	+
chr8	53770100	+
chr8	56856100	-
chr8	58060100	+
chr8	61602500	-
chr8	61614200	+
chr8	61768500	+
chr8	61841700	-
chr8	64275700	+
chr8	66866400	+
chr8	66876000	+
chr8	67728400	+
chr8	71228100	-
chr8	71391300	-
chr8	71408900	+
chr8	71677300	-
chr8	74946200	+
chr8	75044400	-
chr8	78067100	-
chr8	82074700	+
chr8	82080500	+
chr8	82090600	-
chr8	82100700	+
chr8	82113400	-
chr8	82119200	-
chr8	82139400	+
chr8	82151800	+
chr8	82166200	+
chr8	87431600	+
chr8	87447000	-
chr8	94813500	-
chr8	95004500	-
chr8	96023600	-
chr8	97351100	+
chr8	97358200	-
chr8	97363800	-
chr8	97419800	+
chr8	100961000	-
chr8	101384100	-
chr8	103449000	-
chr8	103465300	-
chr8	103476900	+
chr8	117751700	+
chr8	117777100	+
chr8	117786600	+
chr8	117811700	-
chr8	117946400	-
chr8	123867600	-
chr8	123876000	-
chr8	123888500	-
chr8	123900000	-
chr8	123905900	-
chr8	123922700	-
chr8	123929400	+
chr8	123935700	+
chr8	123944400	+
chr8	123992200	+
chr8	124602700	+
chr8	128892300	+
chr8	128901100	-
chr8	128912900	-
chr8	128934100	+
chr8	128948100	+
chr8	128955800	-
chr8	128986700	+
chr8	129009400	+
chr8	129068600	-
chr8	129074700	-
chr8	129082800	+
chr8	129113600	+
chr8	129130400	-
chr8	130967600	-
chr8	130991300	-
chr8	131035300	+
chr8	131043200	+
chr8	131068000	-
chr8	131076800	-
chr8	131094400	+
chr8	131329800	-
chr8	131416000	-
chr8	131431000	+
chr8	133017500	+
chr8	134134500	-
chr8	134330700	-
chr8	134352900	-
chr8	134365900	+
chr8	134374900	-
chr8	134560700	+
chr8	134571000	+
chr8	134598600	+
chr8	134614900	+
chr8	134623000	+
chr8	134634500	+
chr8	141117600	+
chr8	141294900	+
chr8	141528100	-
chr8	141654200	-
chr8	141659500	-
chr8	141669600	-
chr8	141677200	-
chr8	141688200	-
chr8	141695400	-
chr8	142351100	+
chr8	143382800	-
chr8	143421300	-
chr8	143462500	-
chr8	143474300	-
chr8	144174200	+
chr8	144422700	+
chr8	144683300	-
chr8	144691900	+
chr8	144967800	-
chr8	145211800	+
chr8	145657300	-
chr9	272000	-
chr9	286400	-
chr9	341300	+
chr9	2018300	+
chr9	2033700	-
chr9	2116800	+
chr9	2162500	-
chr9	6420600	+
chr9	6427800	-
chr9	6439900	+
chr9	6772400	-
chr9	6865500	+
chr9	20448700	-
chr9	20503700	-
chr9	20548800	+
chr9	33119500	-
chr9	33129500	+
chr9	34188800	+
chr9	35058300	-
chr9	35717800	-
chr9	36372900	+
chr9	36382700	-
chr9	37117600	+
chr9	71551300	-
chr9	72197300	-
chr9	72205400	+
chr9	76909100	-
chr9	76916400	+
chr9	76926400	-
chr9	79652700	+
chr9	79826000	-
chr9	81388300	+
chr9	85504600	-
chr9	87406400	+
chr9	91156800	+
chr9	91211000	-
chr9	91218800	+
chr9	91241500	-
chr9	91252800	-
chr9	91265400	-
chr9	91271200	-
chr9	94530500	+
chr9	94540400	-
chr9	94549300	-
chr9	94789800	-
chr9	94832400	+
chr9	94879100	-
chr9	94885100	-
chr9	94922300	+
chr9	95268900	+
chr9	95310500	-
chr9	95380600	+
chr9	95389600	-
chr9	95401900	-
chr9	95424600	+
chr9	99897400	+
chr9	99963200	+
chr9	99972500	+
chr9	99983000	+
chr9	100915900	-
chr9	100929400	-
chr9	109119300	+
chr9	113711900	+
chr9	113975800	-
chr9	115391200	-
chr9	116724000	+
chr9	122668700	-
chr9	122716600	-
chr9	122987900	-
chr9	126157900	-
chr9	126172400	+
chr9	126191600	+
chr9	126674400	+
chr9	127092500	-
chr9	127323900	-
chr9	129571900	-
chr9	129590400	+
chr9	129757300	-
chr9	129763000	+
chr9	129864400	+
chr9	129894800	-
chr9	129900600	+
chr9	130693900	-
chr9	130714500	-
chr9	130885800	+
chr9	131641800	-
chr9	131735500	-
chr9	131792300	-
chr9	131797800	-
chr9	131812800	+
chr9	131825800	+
chr9	131833100	-
chr9	133272300	+
chr9	133286900	+
chr9	133449900	-
chr9	133455700	-
chr9	133522500	+
chr9	133541500	-
chr9	133563700	+
chr9	133582900	+
chr9	133601300	+
chr9	133873000	+
chr9	133915500	-
chr9	135190500	-
chr9	135202400	-
chr9	135915600	-
chr9	136428000	-
chr9	137871400	+
chr9	137920300	+
chr9	138274800	-
chr9	138557900	-
chr9	138909800	+
chr9	138916200	-
chr9	139200900	-
chr9	139603000	-
chr9	139646300	-
chr9	139672400	+
chr9	139680500	-
chr9	139688000	+
chr9	139709700	+
chr9	139723100	-
chr9	139739400	-
chr9	139772700	+
chrX	7011400	+
chrX	19554300	-
chrX	19577500	+
chrX	19637400	-
chrX	19653400	-
chrX	39810800	+
chrX	39833800	+
chrX	44643000	+
chrX	76885900	-
chrX	129060300	+
chrX	149615700	+
chrX	152255500	+
chrX	152515700	-

Cluster GW5 (in hg18)
chr1	24591500	+
chr1	32128100	-
chr1	40037700	-
chr1	42767700	+
chr1	49015300	+
chr1	55878900	+
chr1	57012300	-
chr1	64258400	+
chr1	64483200	+
chr1	72522900	+
chr1	74248300	+
chr1	76533100	-
chr1	77083000	-
chr1	78243400	-
chr1	78318900	+
chr1	83303200	-
chr1	84383200	-
chr1	85182800	-
chr1	85265600	+
chr1	85536500	-
chr1	87922800	-
chr1	88900200	-
chr1	89436800	-
chr1	89646200	+
chr1	93280300	+
chr1	95093100	-
chr1	95201300	-
chr1	109221600	+
chr1	111055800	+
chr1	117346600	-
chr1	148452900	-
chr1	148936400	+
chr1	152732000	+
chr1	156883300	+
chr1	158684300	+
chr1	166756400	-
chr1	168404500	+
chr1	169550200	-
chr1	170616700	-
chr1	170635300	-
chr1	170686300	-
chr1	177365400	+
chr1	191604300	+
chr1	191654500	+
chr1	192288800	-
chr1	193431900	-
chr1	196863400	-
chr1	198167500	+
chr1	208583000	+
chr1	209622700	+
chr1	218972600	+
chr1	220694400	+
chr1	221363200	-
chr1	228058800	-
chr1	234202100	-
chr1	237777700	+
chr1	239761900	-
chr1	243065500	+
chr1	243200800	+
chr1	244347800	+
chr1	245620300	-
chr1	246136700	-
chr10	4684100	+
chr10	5127400	-
chr10	10220800	-
chr10	14091100	+
chr10	17525600	+
chr10	21736400	-
chr10	22488700	-
chr10	22581000	-
chr10	27620700	-
chr10	28287500	+
chr10	31327700	+
chr10	34451100	-
chr10	53308700	+
chr10	58051700	+
chr10	62208500	-
chr10	62827500	+
chr10	63736000	+
chr10	69193900	-
chr10	72412200	+
chr10	85430000	+
chr10	87491800	-
chr10	90332700	+
chr10	90510000	-
chr10	92756800	-
chr10	95136300	+
chr10	99169100	-
chr10	107587100	+
chr10	112421800	+
chr10	114701300	+
chr10	115312800	+
chr11	4588700	-
chr11	5787000	-
chr11	7414700	-
chr11	9438500	-
chr11	18224500	+
chr11	34613000	-
chr11	58210000	+
chr11	58451500	+
chr11	61629200	+
chr11	65452200	+
chr11	67408600	-
chr11	68487600	+
chr11	71176000	+
chr11	73167900	+
chr11	76187600	+
chr11	77435000	-
chr11	78034100	+
chr11	82506700	+
chr11	85591400	-
chr11	85607200	-
chr11	89624400	-
chr11	95587600	+
chr11	104274400	-
chr11	109704600	+
chr11	109725600	-
chr11	113723200	-
chr11	123491700	+
chr11	125437900	+
chr11	125855400	+
chr12	698500	+
chr12	7685700	+
chr12	8287100	-
chr12	8688900	-
chr12	12626600	+
chr12	14410000	-
chr12	15265600	-
chr12	15634900	-
chr12	18210500	-
chr12	21572200	-
chr12	24785600	-
chr12	24859100	+
chr12	25231300	-
chr12	30895600	-
chr12	31697200	+
chr12	32318100	-
chr12	38147800	-
chr12	38691200	+
chr12	41148900	+
chr12	55432200	-
chr12	57105400	+
chr12	60931700	-
chr12	64598100	-
chr12	65394500	-
chr12	65581200	-
chr12	66508300	-
chr12	73170300	+
chr12	75077000	-
chr12	86580100	-
chr12	87937700	+
chr12	88315900	+
chr12	89217400	-
chr12	90729800	+
chr12	95338900	-
chr12	101835400	-
chr12	109046800	-
chr12	119982500	-
chr12	126134300	-
chr12	127594300	-
chr13	20949100	+
chr13	22793900	+
chr13	28598200	-
chr13	32209600	+
chr13	33194700	-
chr13	37652700	+
chr13	39294800	+
chr13	40735800	-
chr13	42840100	+
chr13	43137600	-
chr13	73608300	+
chr13	82160700	+
chr13	83608700	+
chr13	92256800	-
chr13	101068500	+
chr13	110162900	-
chr14	29810000	-
chr14	32436700	-
chr14	35365300	+
chr14	41288300	-
chr14	44202100	-
chr14	44675400	+
chr14	49399400	+
chr14	54611400	-
chr14	56028500	-
chr14	59122900	-
chr14	59720600	-
chr14	63273600	+
chr14	66723500	-
chr14	70804400	-
chr14	81000600	+
chr14	85470900	+
chr14	89177600	+
chr14	90214900	+
chr14	100384900	+
chr14	101840600	+
chr14	104571100	-
chr15	30737000	+
chr15	33747100	-
chr15	33753000	-
chr15	38262800	-
chr15	49702300	-
chr15	50087600	+
chr15	53761700	-
chr15	54548300	+
chr15	54986600	+
chr15	56490400	-
chr15	70235900	-
chr15	70541300	+
chr15	72515800	+
chr15	75163800	-
chr15	85941700	+
chr15	86890400	+
chr15	91485500	+
chr15	94223500	+
chr15	95235000	-
chr16	12804600	+
chr16	21035500	-
chr16	30763300	+
chr16	45204000	-
chr16	45629500	-
chr16	46371700	-
chr16	56008800	-
chr16	56984000	+
chr16	57405900	+
chr16	78232100	+
chr16	82785500	-
chr17	15527700	+
chr17	15543500	-
chr17	15741300	+
chr17	19352000	+
chr17	19917100	-
chr17	20752200	+
chr17	20943600	-
chr17	26510100	-
chr17	27910100	-
chr17	30637800	-
chr17	34377300	+
chr17	42662700	-
chr17	43974300	-
chr17	46285000	-
chr17	51722300	-
chr17	53265900	-
chr17	55853700	+
chr17	56009300	+
chr17	57295300	+
chr17	65645700	-
chr18	3974800	-
chr18	5049400	-
chr18	7948300	+
chr18	12911100	+
chr18	18855300	-
chr18	19321700	+
chr18	28564100	+
chr18	46887500	+
chr18	54218300	+
chr18	58239300	-
chr18	58687700	+
chr18	63237100	-
chr19	7629000	+
chr19	13978200	+
chr19	14220500	+
chr19	14661600	+
chr19	20398700	+
chr19	21115500	-
chr19	21334000	-
chr19	40423200	+
chr19	42500700	-
chr19	46720300	-
chr2	1018300	-
chr2	9751300	-
chr2	16653200	+
chr2	20885800	+
chr2	26488400	+
chr2	31400600	+
chr2	31891400	-
chr2	35970500	+
chr2	37680700	-
chr2	39309100	-
chr2	41948700	-
chr2	50054400	+
chr2	54196500	-
chr2	60185900	-
chr2	61045300	-
chr2	61752000	-
chr2	61844500	+
chr2	64116300	+
chr2	64832000	+
chr2	67478500	-
chr2	68446600	-
chr2	99049300	+
chr2	100537400	+
chr2	107695200	+
chr2	111152100	+
chr2	112648700	-
chr2	127239000	+
chr2	128707300	+
chr2	144277100	+
chr2	147839100	+
chr2	148271400	-
chr2	150109700	-
chr2	153671600	-
chr2	160080800	+
chr2	160789500	-
chr2	161655000	+
chr2	171883200	-
chr2	173266400	-
chr2	173516700	+
chr2	177570800	-
chr2	177721800	-
chr2	177738800	+
chr2	181679100	-
chr2	181882200	+
chr2	181967800	+
chr2	193921000	-
chr2	196416200	-
chr2	197489100	-
chr2	197900700	+
chr2	198578800	-
chr2	200423800	+
chr2	200957100	-
chr2	201286900	-
chr2	202538200	-
chr2	203444400	-
chr2	204718900	-
chr2	205650100	-
chr2	207910800	+
chr2	210363400	-
chr2	215744200	+
chr2	217796600	+
chr2	225210900	+
chr2	240991200	+
chr20	3349600	-
chr20	8531400	+
chr20	18722800	-
chr20	20013800	-
chr20	39403000	-
chr20	39755500	-
chr20	42215900	-
chr20	42799000	-
chr20	51702700	-
chr20	51790000	+
chr20	57437600	+
chr21	14980600	+
chr21	15056800	+
chr21	15809000	-
chr21	16489100	-
chr21	17433700	+
chr21	24181900	-
chr21	26085200	-
chr21	29040200	-
chr21	29743700	+
chr21	31273700	-
chr21	31307400	-
chr21	36373400	+
chr21	42400900	+
chr22	15685900	+
chr22	17627700	-
chr22	25020800	-
chr22	28141700	-
chr22	28773300	+
chr22	35579200	+
chr22	44357600	+
chr3	7222900	-
chr3	12412200	-
chr3	12488200	-
chr3	28189200	-
chr3	34500500	-
chr3	36460600	+
chr3	50963500	-
chr3	56951700	+
chr3	67818400	+
chr3	69381400	+
chr3	69459800	+
chr3	72615100	-
chr3	72681300	+
chr3	75567400	+
chr3	88128900	-
chr3	88163500	+
chr3	99230900	-
chr3	99758300	+
chr3	101804600	+
chr3	103616500	+
chr3	105481300	-
chr3	105493400	+
chr3	107084500	-
chr3	108442100	+
chr3	109748500	-
chr3	110022700	-
chr3	110115800	+
chr3	113495000	+
chr3	115040500	+
chr3	118059400	+
chr3	131381100	-
chr3	138216200	-
chr3	142496100	+
chr3	144393100	+
chr3	150902700	-
chr3	153398000	-
chr3	153446900	+
chr3	154410700	+
chr3	157361700	-
chr3	161111800	-
chr3	171146000	+
chr3	173763900	-
chr3	173796200	+
chr3	178649400	+
chr3	181467500	-
chr3	184403200	-
chr3	187435800	+
chr3	188080500	+
chr3	190322600	-
chr3	190595200	+
chr3	191370200	+
chr3	193532600	+
chr4	14612700	-
chr4	15573700	+
chr4	20030100	-
chr4	22902300	+
chr4	25844000	+
chr4	35922200	+
chr4	38534800	-
chr4	38593600	-
chr4	46707300	-
chr4	53573200	+
chr4	57361200	-
chr4	68123800	+
chr4	75482800	-
chr4	83277200	-
chr4	88989400	+
chr4	89519400	-
chr4	90610100	+
chr4	100703800	-
chr4	101673200	-
chr4	101710100	-
chr4	102035600	-
chr4	102073600	+
chr4	102863700	+
chr4	103571300	-
chr4	111656200	+
chr4	119732500	-
chr4	119991400	-
chr4	120281000	-
chr4	123760800	+
chr4	129654400	+
chr4	129944600	-
chr4	138673400	-
chr4	141038700	+
chr4	143557800	-
chr4	143707900	+
chr4	144499300	+
chr4	146874600	+
chr4	146970900	-
chr4	148198400	-
chr4	149517500	+
chr4	153022800	+
chr4	159793200	+
chr4	160070600	+
chr4	166750600	-
chr4	170238200	-
chr4	177354100	-
chr4	178387400	-
chr4	185186500	+
chr5	40312800	-
chr5	40615100	-
chr5	40877500	-
chr5	42793300	-
chr5	42944400	+
chr5	43043400	-
chr5	49998200	-
chr5	51801100	-
chr5	52276800	-
chr5	52372400	+
chr5	56362500	-
chr5	56630800	-
chr5	56831400	-
chr5	58629000	-
chr5	61425500	+
chr5	61587700	+
chr5	66880700	-
chr5	67701100	+
chr5	68374100	+
chr5	71089800	-
chr5	71115000	-
chr5	73025100	+
chr5	75506900	+
chr5	82672600	-
chr5	86513800	+
chr5	93472700	+
chr5	94443000	+
chr5	95928000	+
chr5	100144200	-
chr5	106752700	+
chr5	110090600	-
chr5	116818800	+
chr5	119821100	-
chr5	123947700	-
chr5	126125500	+
chr5	128489800	+
chr5	130200700	-
chr5	137221600	+
chr5	143151500	+
chr5	146237900	+
chr5	152916100	-
chr5	157011800	-
chr5	158480400	+
chr5	170714600	+
chr6	2935300	+
chr6	6756800	-
chr6	12591900	-
chr6	14182900	+
chr6	14985500	+
chr6	22023500	+
chr6	26358000	-
chr6	26379900	-
chr6	26548500	-
chr6	26582600	+
chr6	26677000	-
chr6	27221800	-
chr6	27253500	+
chr6	27900200	-
chr6	30303400	+
chr6	32605300	+
chr6	38778800	-
chr6	40010100	-
chr6	43218800	+
chr6	45814000	-
chr6	47274300	-
chr6	52968000	-
chr6	74352600	-
chr6	78241200	+
chr6	80930900	-
chr6	88599200	-
chr6	90241800	+
chr6	91706800	-
chr6	106142200	+
chr6	106336800	-
chr6	106455100	-
chr6	114059500	-
chr6	120218800	+
chr6	121697900	-
chr6	122686300	-
chr6	128475400	+
chr6	130381700	-
chr6	130623000	-
chr6	135477800	-
chr6	135586900	-
chr6	135802700	+
chr6	138818400	-
chr6	147685700	-
chr6	151688000	+
chr6	153311200	+
chr6	154640500	-
chr6	155470500	-
chr6	169038100	-
chr7	7926400	-
chr7	12488300	-
chr7	12642100	+
chr7	16569400	+
chr7	17150300	+
chr7	18501500	+
chr7	21344600	+
chr7	22891700	+
chr7	23353300	+
chr7	24080800	+
chr7	25268700	+
chr7	25502600	+
chr7	25626900	+
chr7	34729800	-
chr7	38432100	-
chr7	43840800	-
chr7	45210700	-
chr7	45774900	+
chr7	50166800	-
chr7	51156800	+
chr7	55109300	-
chr7	71987900	+
chr7	93554200	+
chr7	95946900	-
chr7	101184300	+
chr7	103239800	+
chr7	110438200	-
chr7	110454200	+
chr7	110509900	-
chr7	110518600	-
chr7	114344000	-
chr7	124076200	+
chr7	129478600	-
chr7	129821300	+
chr7	141322300	+
chr7	141969500	+
chr7	148743100	+
chr8	2200200	-
chr8	8123500	+
chr8	10943500	-
chr8	12967100	+
chr8	16506000	-
chr8	18634000	+
chr8	24897600	+
chr8	26171900	-
chr8	27188600	-
chr8	32905200	-
chr8	37378300	+
chr8	42222800	-
chr8	42742400	+
chr8	54599500	+
chr8	58820600	+
chr8	61047400	+
chr8	62170500	+
chr8	64114000	+
chr8	64301300	+
chr8	66953400	+
chr8	68580300	-
chr8	69336200	-
chr8	71017400	-
chr8	71692200	-
chr8	81507700	-
chr8	86613000	+
chr8	94047100	-
chr8	94986900	+
chr8	95537600	-
chr8	101467200	+
chr8	102583100	-
chr8	103279200	-
chr8	117465900	+
chr8	126718800	-
chr8	127149900	+
chr8	129687100	+
chr8	135032900	+
chr8	135125900	-
chr8	144514300	+
chr9	6070000	+
chr9	6740700	+
chr9	19462400	-
chr9	19916800	-
chr9	21029200	+
chr9	22199400	-
chr9	31201000	+
chr9	32334300	+
chr9	35267200	-
chr9	70800900	+
chr9	74937100	-
chr9	81662800	+
chr9	99733000	+
chr9	101055700	+
chr9	102011800	+
chr9	116499500	-
chr9	124266800	-
chr9	127631800	+
chrX	12771800	+
chrX	13402400	-
chrX	13581300	-
chrX	13616900	-
chrX	14425400	-
chrX	14961500	-
chrX	15421800	-
chrX	15783400	+
chrX	18282700	+
chrX	18603300	+
chrX	21586400	+
chrX	23835600	+
chrX	23881500	-
chrX	23953800	+
chrX	30505200	+
chrX	32376600	-
chrX	40480100	+
chrX	40677600	+
chrX	45595000	-
chrX	46977700	-
chrX	47581200	-
chrX	48934900	-
chrX	49574200	+
chrX	52021500	+
chrX	52980900	-
chrX	54682900	+
chrX	56772700	-
chrX	57164900	-
chrX	57329600	-
chrX	57954200	+
chrX	65775200	+
chrX	70629400	-
chrX	72351300	+
chrX	73081000	-
chrX	73750900	+
chrX	77053200	-
chrX	77281300	+
chrX	77468900	+
chrX	78509100	+
chrX	80263600	+
chrX	80344000	-
chrX	82777100	-
chrX	83643700	+
chrX	85189000	+
chrX	92815200	+
chrX	96705600	-
chrX	99778000	-
chrX	100239400	+
chrX	100800800	+
chrX	101297100	-
chrX	101911100	-
chrX	102696500	-
chrX	102770900	-
chrX	105509100	-
chrX	105614400	+
chrX	105742100	+
chrX	106335700	+
chrX	108666700	+
chrX	110253500	+
chrX	119579100	+
chrX	119622400	-
chrX	122726900	-
chrX	122921500	-
chrX	123308300	-
chrX	130775100	-
chrX	130792900	-
chrX	132885000	-
chrX	133335200	-
chrX	133768800	+
chrX	135508700	+
chrX	135790300	+
chrX	151951800	-
chrX	152528800	+
chrX	154515700	-
chrY	13283700	-
chrY	13770700	-
chrY	14173800	+
chrY	20123900	+
chrY	20188700	-
chrY	20366200	+
chrY	21147100	-
chrY	21369600	+

Cluster GW6 (in hg18)
chr1	3610900	-
chr1	20353700	+
chr1	26094700	+
chr1	26876300	+
chr1	27244100	-
chr1	28140300	+
chr1	29486000	+
chr1	31733400	-
chr1	34922600	-
chr1	35975800	-
chr1	37936700	+
chr1	40164400	-
chr1	40867700	-
chr1	46304300	-
chr1	51406700	+
chr1	51453300	-
chr1	55204000	+
chr1	55515800	+
chr1	55526700	-
chr1	55702400	-
chr1	59012200	-
chr1	59168400	-
chr1	59935900	-
chr1	61509400	+
chr1	66674800	+
chr1	66977600	+
chr1	67571700	-
chr1	68725700	+
chr1	70733500	-
chr1	70755000	-
chr1	76538400	+
chr1	77546800	-
chr1	78294100	-
chr1	79075000	+
chr1	82399800	+
chr1	83057400	+
chr1	84509900	+
chr1	85479000	+
chr1	86755500	+
chr1	87012300	-
chr1	87688200	-
chr1	89379700	-
chr1	91293400	-
chr1	91403000	+
chr1	92255300	+
chr1	92440000	-
chr1	92742700	+
chr1	93211700	-
chr1	93853400	+
chr1	93954000	-
chr1	95040300	-
chr1	95121700	+
chr1	98626400	+
chr1	98644400	+
chr1	99051800	+
chr1	100059200	-
chr1	100544800	+
chr1	108388700	-
chr1	110547700	-
chr1	113115000	+
chr1	113485100	-
chr1	117684300	-
chr1	120143600	+
chr1	143652900	-
chr1	145639100	+
chr1	145701400	+
chr1	150719500	+
chr1	157055400	-
chr1	157067500	+
chr1	158283100	+
chr1	161092400	+
chr1	166579000	-
chr1	167227600	+
chr1	167322800	-
chr1	167744600	-
chr1	169881200	-
chr1	170960400	+
chr1	171014900	+
chr1	171358700	+
chr1	171768300	+
chr1	171786800	+
chr1	173265800	-
chr1	177239100	-
chr1	178761100	-
chr1	178846200	-
chr1	179660500	+
chr1	180283800	+
chr1	180471600	-
chr1	180485000	+
chr1	181514900	+
chr1	183609500	+
chr1	183910100	+
chr1	183916200	+
chr1	190680900	+
chr1	190786100	-
chr1	191730100	-
chr1	192370000	+
chr1	195336500	-
chr1	195717100	+
chr1	196338300	+
chr1	196770600	-
chr1	197399300	+
chr1	198365100	+
chr1	198573000	-
chr1	198677300	+
chr1	198797900	-
chr1	198864000	-
chr1	200463700	-
chr1	201235600	-
chr1	203228000	+
chr1	204456900	-
chr1	205202500	+
chr1	209788800	-
chr1	209921600	-
chr1	210083200	+
chr1	210330700	+
chr1	210365400	-
chr1	210426900	-
chr1	210871100	+
chr1	210923700	-
chr1	212636600	+
chr1	212873000	-
chr1	215900500	-
chr1	220235100	+
chr1	220547100	-
chr1	222360700	+
chr1	222768400	-
chr1	223000300	-
chr1	225244400	+
chr1	227408100	-
chr1	227956700	-
chr1	228253600	+
chr1	230428900	-
chr1	231801800	-
chr1	232512600	-
chr1	232690000	-
chr1	237892400	+
chr1	240087500	-
chr1	242469600	-
chr1	244929000	+
chr10	3034500	-
chr10	3884700	+
chr10	3899100	-
chr10	7621100	-
chr10	9377000	-
chr10	10500000	+
chr10	13255100	-
chr10	13263300	-
chr10	13565900	-
chr10	19029700	+
chr10	21702100	-
chr10	28761700	-
chr10	31080500	-
chr10	31369100	+
chr10	31429800	+
chr10	32074700	+
chr10	33361300	+
chr10	33540800	-
chr10	33815900	+
chr10	34580800	-
chr10	34966500	+
chr10	43637800	+
chr10	49166800	-
chr10	52820100	-
chr10	53137700	-
chr10	53643400	+
chr10	60096200	+
chr10	61056400	-
chr10	61112300	-
chr10	62217100	-
chr10	62249900	+
chr10	62713200	-
chr10	63195800	-
chr10	63610800	-
chr10	65542600	-
chr10	69213800	-
chr10	72906000	+
chr10	76168800	-
chr10	78468000	-
chr10	79750400	+
chr10	79792400	-
chr10	80008700	+
chr10	80374800	-
chr10	81853900	+
chr10	88335100	-
chr10	89385500	+
chr10	89778000	+
chr10	90557200	+
chr10	90836000	+
chr10	91556000	-
chr10	91695500	+
chr10	92733400	+
chr10	94506400	+
chr10	95129600	-
chr10	95259700	+
chr10	97582000	+
chr10	97614600	+
chr10	98037600	+
chr10	98397500	+
chr10	98445500	-
chr10	99664800	-
chr10	103938700	+
chr10	104991000	+
chr10	105697400	+
chr10	107607800	-
chr10	109401300	-
chr10	112524300	-
chr10	112861700	-
chr10	114086000	-
chr10	116661300	-
chr10	118135000	+
chr10	121436500	-
chr10	122692600	-
chr10	127815000	+
chr10	130790300	-
chr11	1567200	-
chr11	6750700	+
chr11	7871200	+
chr11	8986400	+
chr11	8995300	+
chr11	12154000	-
chr11	13207400	+
chr11	16880700	-
chr11	18094000	-
chr11	18742100	+
chr11	18756900	-
chr11	19208300	-
chr11	19930100	-
chr11	22681400	-
chr11	28058800	+
chr11	31304900	+
chr11	31936300	-
chr11	32277100	-
chr11	32753900	+
chr11	35327700	-
chr11	39341000	+
chr11	39349800	-
chr11	43247000	-
chr11	45633300	+
chr11	47352100	-
chr11	56307700	-
chr11	57323700	-
chr11	58022400	-
chr11	58164600	+
chr11	58216400	+
chr11	58250300	-
chr11	58808400	+
chr11	59342800	+
chr11	60006300	+
chr11	76575900	-
chr11	78693900	-
chr11	83113400	-
chr11	85581700	+
chr11	88030200	-
chr11	93018500	-
chr11	94011500	+
chr11	94998000	+
chr11	95014500	+
chr11	95088900	-
chr11	95839600	+
chr11	101953300	+
chr11	102412600	-
chr11	103058300	+
chr11	103985900	+
chr11	104148700	+
chr11	107065100	+
chr11	109322300	+
chr11	110822200	+
chr11	110950700	-
chr11	111965300	-
chr11	113756000	+
chr11	114700800	+
chr11	123986500	-
chr11	124827100	-
chr11	125316900	+
chr11	127614300	-
chr11	129588800	+
chr12	676900	-
chr12	2074100	+
chr12	4545000	-
chr12	7264200	-
chr12	8364300	+
chr12	10598700	+
chr12	12852300	+
chr12	12954000	+
chr12	13255700	+
chr12	18917400	-
chr12	19448200	+
chr12	21341600	+
chr12	22037300	-
chr12	22079500	-
chr12	23250500	-
chr12	24866400	+
chr12	25069900	-
chr12	25421500	+
chr12	29204300	+
chr12	29471700	+
chr12	32500000	+
chr12	32626200	+
chr12	32633300	+
chr12	34099300	-
chr12	37452000	+
chr12	40202200	-
chr12	40980000	-
chr12	44198500	+
chr12	44752200	-
chr12	49034300	-
chr12	53681700	-
chr12	55104800	+
chr12	55517400	-
chr12	55612300	+
chr12	62495000	-
chr12	63836700	+
chr12	64236900	+
chr12	65457200	+
chr12	66721400	-
chr12	66878400	-
chr12	67639300	-
chr12	67741900	-
chr12	67834600	-
chr12	67841600	+
chr12	68369300	-
chr12	70184200	-
chr12	73937900	-
chr12	74262300	-
chr12	74418300	+
chr12	74492900	-
chr12	74624700	+
chr12	75952800	+
chr12	75968100	+
chr12	87145200	+
chr12	88853200	+
chr12	88994100	-
chr12	91259000	+
chr12	91379900	+
chr12	91421000	-
chr12	91906200	-
chr12	92016300	-
chr12	92102500	-
chr12	92167000	+
chr12	92900800	+
chr12	92965500	+
chr12	93712800	+
chr12	93748900	+
chr12	94021200	-
chr12	95497400	-
chr12	97672100	+
chr12	100885900	-
chr12	102419400	-
chr12	103072200	+
chr12	105684700	-
chr12	107582900	-
chr12	109620300	+
chr12	109699000	-
chr12	115247400	+
chr12	120314100	+
chr12	121504300	+
chr12	126728600	-
chr12	127881000	+
chr13	20019000	-
chr13	20691100	-
chr13	20704900	+
chr13	20728400	+
chr13	21323200	-
chr13	21357700	-
chr13	23535600	+
chr13	25975700	-
chr13	26949600	-
chr13	27520700	-
chr13	27830500	+
chr13	28057900	-
chr13	28267600	-
chr13	28835000	+
chr13	29584300	+
chr13	29642100	-
chr13	31245600	-
chr13	32677600	+
chr13	32701000	+
chr13	33117900	+
chr13	33139900	-
chr13	33216000	+
chr13	33248600	-
chr13	33265200	-
chr13	35910800	+
chr13	37887700	-
chr13	38567900	+
chr13	39574700	+
chr13	40743800	-
chr13	40759600	-
chr13	41449800	+
chr13	42830100	-
chr13	43589300	-
chr13	43663600	+
chr13	43765600	-
chr13	43824600	+
chr13	44528100	+
chr13	45806300	+
chr13	48352100	-
chr13	49699600	-
chr13	49730300	-
chr13	49791700	-
chr13	50067000	-
chr13	50318900	+
chr13	50531400	-
chr13	50542600	-
chr13	59003800	+
chr13	59151300	+
chr13	59194700	+
chr13	59242300	+
chr13	59336700	+
chr13	59492000	-
chr13	59619600	-
chr13	59628400	-
chr13	75348400	+
chr13	76195900	+
chr13	76860600	+
chr13	77610300	-
chr13	79872800	+
chr13	80034400	-
chr13	84405700	-
chr13	93762800	+
chr13	93802500	+
chr13	94907600	-
chr13	95503400	-
chr13	101405700	+
chr13	101907300	+
chr13	104325100	+
chr13	105736900	+
chr13	109020200	+
chr14	20117600	-
chr14	21095500	+
chr14	22354800	-
chr14	29894700	-
chr14	30061700	-
chr14	31466600	+
chr14	32103600	+
chr14	32992500	-
chr14	33658700	-
chr14	33871300	+
chr14	34204900	-
chr14	35560600	+
chr14	37450100	-
chr14	37664300	+
chr14	38767900	+
chr14	41257400	+
chr14	48998900	-
chr14	49015600	-
chr14	50214100	+
chr14	51104000	-
chr14	54718100	+
chr14	56597500	-
chr14	57730600	+
chr14	58108300	+
chr14	58198800	-
chr14	59883700	-
chr14	62820000	-
chr14	62832000	+
chr14	63802200	+
chr14	64672700	+
chr14	67895800	-
chr14	69292000	+
chr14	69555300	+
chr14	73004500	+
chr14	74900400	-
chr14	78102600	-
chr14	80514400	+
chr14	85459900	+
chr14	87716800	+
chr14	95653800	-
chr14	96978600	-
chr15	18438900	-
chr15	19398800	+
chr15	19428800	+
chr15	29129200	-
chr15	35843200	-
chr15	36148300	+
chr15	36667000	-
chr15	38703800	+
chr15	38938400	+
chr15	39230400	-
chr15	40198600	-
chr15	45893100	-
chr15	46365400	-
chr15	46941700	+
chr15	46985600	+
chr15	48214200	-
chr15	48222800	+
chr15	48960900	-
chr15	50809800	-
chr15	56539400	+
chr15	56708100	+
chr15	57032100	-
chr15	57190500	+
chr15	57203700	+
chr15	57217300	+
chr15	57694900	-
chr15	57882500	+
chr15	60203900	+
chr15	62024600	+
chr15	63884700	-
chr15	64772500	-
chr15	66026300	-
chr15	67186500	+
chr15	67478100	-
chr15	67497300	-
chr15	71095400	-
chr15	73896600	+
chr15	74230100	-
chr15	74261300	-
chr15	77461100	-
chr15	78034200	-
chr15	78057700	-
chr15	78884600	+
chr15	85914200	+
chr15	86590700	+
chr15	86746500	+
chr15	87932100	+
chr15	88651100	-
chr15	89332700	+
chr15	90632600	-
chr15	92276800	-
chr15	92464500	-
chr15	94183900	-
chr16	6632200	+
chr16	10258300	+
chr16	10854300	+
chr16	13999100	+
chr16	25012200	+
chr16	25758000	-
chr16	45539900	-
chr16	46692900	-
chr16	56281000	+
chr16	65956100	+
chr16	67393000	+
chr16	70597200	-
chr16	70716800	-
chr16	71757800	+
chr16	73811300	+
chr16	75827300	+
chr16	76594400	-
chr16	79032000	+
chr16	79829400	+
chr16	80389600	+
chr16	80784200	-
chr16	84587300	-
chr16	84748900	-
chr17	4225200	+
chr17	4246600	-
chr17	4412200	+
chr17	6340400	-
chr17	12875400	-
chr17	22569900	+
chr17	25352200	-
chr17	27455500	-
chr17	40375300	+
chr17	50781400	+
chr17	50786700	-
chr17	51057200	+
chr17	52442300	+
chr17	54872900	+
chr17	57552900	+
chr17	61341100	+
chr17	67930200	+
chr17	75317100	+
chr18	3035600	-
chr18	9675100	+
chr18	10113200	+
chr18	12226900	+
chr18	14914800	+
chr18	17012400	-
chr18	18673300	-
chr18	19719500	-
chr18	19763500	+
chr18	20203200	-
chr18	32435600	-
chr18	33264000	+
chr18	35146600	-
chr18	37529200	+
chr18	37948400	-
chr18	40727300	+
chr18	40843200	-
chr18	42158800	-
chr18	42983700	+
chr18	46136900	-
chr18	50028000	-
chr18	50536500	-
chr18	54905900	-
chr18	55700500	-
chr18	55745900	-
chr18	57790700	-
chr18	71318700	+
chr19	2838800	-
chr19	6051800	-
chr19	7743400	+
chr19	13249600	-
chr19	20219100	+
chr19	20296900	-
chr19	20748600	-
chr19	23773800	+
chr19	34460200	-
chr19	37520600	+
chr19	37641800	-
chr19	37976400	-
chr2	2884900	-
chr2	7668700	+
chr2	7831000	+
chr2	7883200	+
chr2	8382300	-
chr2	8434900	-
chr2	8490500	+
chr2	10022000	-
chr2	10187200	+
chr2	10922400	+
chr2	20429100	-
chr2	20488400	-
chr2	24903800	+
chr2	25272500	+
chr2	26686100	+
chr2	30192000	-
chr2	30875200	-
chr2	32342800	+
chr2	33401100	-
chr2	36055800	-
chr2	37636100	+
chr2	37914100	+
chr2	38989100	+
chr2	40290600	-
chr2	41796400	-
chr2	44086200	+
chr2	44125200	-
chr2	44217400	-
chr2	45251900	+
chr2	47403800	+
chr2	47833200	-
chr2	48226100	+
chr2	50025200	+
chr2	57988400	-
chr2	58694900	-
chr2	62416100	+
chr2	62491900	+
chr2	64304100	-
chr2	64354400	+
chr2	64920800	-
chr2	65246000	+
chr2	65401300	+
chr2	68917000	-
chr2	70196800	-
chr2	72004800	-
chr2	74654800	-
chr2	74665900	+
chr2	74858400	-
chr2	84114800	-
chr2	84578900	+
chr2	85371500	+
chr2	95698100	+
chr2	99488000	-
chr2	100274200	+
chr2	100907500	+
chr2	101358200	-
chr2	101484600	+
chr2	102045700	+
chr2	102645200	+
chr2	102669600	+
chr2	104431200	-
chr2	104921800	-
chr2	104959800	-
chr2	112380500	+
chr2	112734000	-
chr2	113230200	+
chr2	113286100	-
chr2	113295800	-
chr2	119813200	+
chr2	128065400	+
chr2	129115300	+
chr2	134318400	-
chr2	143558300	-
chr2	144708400	+
chr2	149647200	+
chr2	150102200	-
chr2	150162400	+
chr2	152590300	+
chr2	153159200	+
chr2	156112000	+
chr2	157757300	+
chr2	157810600	+
chr2	157825700	+
chr2	159309400	-
chr2	159659200	+
chr2	161081400	+
chr2	161558700	+
chr2	162274700	-
chr2	166008600	+
chr2	168902000	-
chr2	169529700	-
chr2	170026900	+
chr2	171442900	+
chr2	173721800	-
chr2	173943400	+
chr2	175609800	-
chr2	175777100	-
chr2	177019900	+
chr2	177399600	+
chr2	177766800	-
chr2	180698600	+
chr2	181993800	+
chr2	183240900	-
chr2	188021300	-
chr2	190662800	+
chr2	191296000	-
chr2	192735000	-
chr2	196186400	-
chr2	196674200	+
chr2	196988300	+
chr2	197324100	-
chr2	202558400	+
chr2	202745400	+
chr2	203162000	-
chr2	204460000	-
chr2	204567600	+
chr2	205702700	-
chr2	206210600	+
chr2	206279700	+
chr2	207182300	+
chr2	213531900	+
chr2	223326000	-
chr2	223386200	+
chr2	225219300	-
chr2	225757800	+
chr2	231162700	-
chr2	231935600	-
chr2	234412200	-
chr20	1141400	+
chr20	1636800	-
chr20	4298200	+
chr20	4649100	-
chr20	5574100	+
chr20	5666200	+
chr20	8002200	-
chr20	8170800	+
chr20	9249000	+
chr20	9538000	-
chr20	9906200	-
chr20	10019500	+
chr20	11133900	+
chr20	12894300	-
chr20	29797900	+
chr20	29822400	-
chr20	34280200	-
chr20	39392000	-
chr20	39801100	+
chr20	41744500	+
chr20	42671900	+
chr20	43417000	+
chr20	48854900	-
chr20	54382200	+
chr20	56218300	+
chr20	57957600	-
chr21	14748600	+
chr21	14924800	+
chr21	15031000	+
chr21	15719500	-
chr21	15774000	+
chr21	17939000	+
chr21	21495200	+
chr21	25715900	-
chr21	25758400	+
chr21	36897300	-
chr21	38561900	+
chr21	39034900	+
chr22	20841100	+
chr22	20855100	+
chr22	20895300	+
chr22	24462800	-
chr22	24476300	-
chr22	27129300	-
chr22	28804700	+
chr22	28910800	+
chr22	29469800	+
chr22	31712000	+
chr22	34748300	-
chr22	37608800	-
chr22	38585800	+
chr3	2811300	+
chr3	2944100	-
chr3	8345700	+
chr3	8432700	+
chr3	10436900	-
chr3	11113100	+
chr3	12155000	-
chr3	12479200	-
chr3	16263900	-
chr3	16840900	-
chr3	18576300	-
chr3	18772600	-
chr3	18797600	-
chr3	21346100	+
chr3	22994000	-
chr3	25910600	-
chr3	27240600	-
chr3	27879100	+
chr3	28933400	-
chr3	30280000	+
chr3	30950800	-
chr3	31772500	+
chr3	32899300	+
chr3	34265200	-
chr3	36717300	+
chr3	36974800	+
chr3	37973900	+
chr3	38168600	-
chr3	38977200	+
chr3	39223800	+
chr3	39311700	+
chr3	39363900	+
chr3	42264400	-
chr3	44843600	+
chr3	47538900	+
chr3	48129500	-
chr3	50984300	+
chr3	51283200	+
chr3	52010600	+
chr3	53712100	+
chr3	59349100	+
chr3	59368100	-
chr3	62149400	+
chr3	64315800	+
chr3	69437400	-
chr3	69557600	+
chr3	69571300	+
chr3	72004600	+
chr3	72406600	-
chr3	87403100	+
chr3	87465200	-
chr3	96907200	+
chr3	99193100	-
chr3	99240600	+
chr3	99830000	+
chr3	101791700	+
chr3	101853200	-
chr3	102335700	-
chr3	107079000	-
chr3	107092100	+
chr3	107136000	+
chr3	107190000	-
chr3	107270200	+
chr3	107386400	+
chr3	109112400	-
chr3	109494900	-
chr3	109609200	-
chr3	111829100	-
chr3	111888200	+
chr3	113215200	-
chr3	113276400	+
chr3	113565800	-
chr3	113845000	-
chr3	115114700	-
chr3	117958600	-
chr3	120144200	-
chr3	120764300	-
chr3	120797800	-
chr3	120982800	+
chr3	121769400	-
chr3	122608600	+
chr3	122803000	+
chr3	123196800	+
chr3	123231900	+
chr3	123268500	+
chr3	123282000	+
chr3	123293400	-
chr3	123303100	-
chr3	123448900	+
chr3	123497200	-
chr3	124177200	+
chr3	124515900	+
chr3	124528000	-
chr3	125962700	+
chr3	126102100	-
chr3	128899000	+
chr3	129057900	-
chr3	131730800	+
chr3	132052000	+
chr3	134649700	+
chr3	134662800	-
chr3	134676500	+
chr3	136004700	-
chr3	139267500	+
chr3	140064700	+
chr3	142213300	-
chr3	142306400	+
chr3	142394000	-
chr3	142522600	-
chr3	144284000	-
chr3	147751300	+
chr3	147825400	+
chr3	150446400	+
chr3	150477400	+
chr3	150672100	+
chr3	152428900	+
chr3	152476600	+
chr3	152584700	+
chr3	153413600	+
chr3	153434200	-
chr3	153796900	+
chr3	154600300	-
chr3	157865100	-
chr3	158304500	-
chr3	158732500	+
chr3	158809500	+
chr3	161129800	+
chr3	161212700	+
chr3	161227500	+
chr3	161843000	+
chr3	162711000	+
chr3	169414200	+
chr3	170710400	+
chr3	172871900	+
chr3	178824900	+
chr3	178880000	+
chr3	179286400	+
chr3	180664800	+
chr3	183883200	-
chr3	183917300	+
chr3	184638400	+
chr3	186737700	+
chr3	187143700	+
chr3	187443000	+
chr3	188818100	-
chr3	188913800	-
chr3	189071100	-
chr3	190372000	+
chr3	190505700	-
chr3	191147900	-
chr3	194944600	-
chr4	1516900	+
chr4	8592500	+
chr4	10187500	+
chr4	10268200	-
chr4	13267700	+
chr4	13592200	+
chr4	13788700	+
chr4	14986600	+
chr4	15024800	+
chr4	15280000	-
chr4	15734000	+
chr4	17428300	+
chr4	17434700	+
chr4	20232300	-
chr4	24030100	-
chr4	25686300	+
chr4	36004100	+
chr4	36027300	-
chr4	40130300	-
chr4	40171100	-
chr4	40274000	-
chr4	40419500	-
chr4	40605800	-
chr4	40612500	-
chr4	42476000	+
chr4	44326300	+
chr4	44502700	+
chr4	56319000	+
chr4	56862500	-
chr4	57201400	+
chr4	57283200	-
chr4	70731800	+
chr4	70777300	-
chr4	71459900	+
chr4	72226300	+
chr4	72795900	+
chr4	73387500	+
chr4	76843200	-
chr4	77164100	+
chr4	77213400	-
chr4	77230300	+
chr4	78286700	-
chr4	78526100	-
chr4	78573600	-
chr4	79753900	+
chr4	79877100	-
chr4	80743900	+
chr4	82791700	-
chr4	83117500	-
chr4	84528600	-
chr4	84644400	-
chr4	85286900	-
chr4	87684700	+
chr4	88818000	-
chr4	89460800	+
chr4	90161700	-
chr4	90300300	-
chr4	90578900	-
chr4	91688600	+
chr4	95483400	-
chr4	96053100	-
chr4	96328600	+
chr4	101989100	+
chr4	102150500	-
chr4	104277500	+
chr4	105911500	-
chr4	106107300	+
chr4	106202000	-
chr4	109567400	-
chr4	111070900	+
chr4	111147700	-
chr4	119254000	-
chr4	120108600	+
chr4	122279100	-
chr4	122393300	-
chr4	123039300	-
chr4	123118500	+
chr4	123683300	+
chr4	123776300	-
chr4	123804800	-
chr4	123851600	-
chr4	129849800	+
chr4	130405200	-
chr4	130505500	+
chr4	140791700	+
chr4	140798500	-
chr4	141383700	+
chr4	141861400	+
chr4	142004000	-
chr4	144546800	+
chr4	145318400	+
chr4	148158000	-
chr4	151126300	-
chr4	152347400	+
chr4	153363100	-
chr4	154539800	-
chr4	155046300	-
chr4	155757400	-
chr4	160218600	-
chr4	160570000	+
chr4	178409100	+
chr4	178482200	-
chr4	178559800	+
chr4	178912200	-
chr4	184986600	-
chr4	185446700	-
chr4	185742400	-
chr4	185904600	-
chr4	190361300	-
chr5	8192500	+
chr5	10542900	+
chr5	11016000	-
chr5	16812500	+
chr5	16837700	+
chr5	31756400	-
chr5	31807400	-
chr5	32009300	-
chr5	32069200	-
chr5	32246700	+
chr5	32541300	-
chr5	34730100	+
chr5	35941200	-
chr5	36393900	-
chr5	36455100	+
chr5	38227800	-
chr5	41101900	+
chr5	42912000	+
chr5	44912000	-
chr5	52339100	-
chr5	52362400	-
chr5	52743200	+
chr5	54165700	-
chr5	54369000	-
chr5	55647500	+
chr5	55823200	-
chr5	58218800	-
chr5	58976800	+
chr5	60006100	+
chr5	60020400	-
chr5	61616300	+
chr5	66153700	-
chr5	66172300	+
chr5	67472900	+
chr5	67738000	+
chr5	68415400	+
chr5	71219700	-
chr5	72851600	-
chr5	73822300	+
chr5	74306300	-
chr5	76122400	+
chr5	76711600	-
chr5	78465500	-
chr5	78884900	-
chr5	78893000	-
chr5	79134300	+
chr5	79295000	-
chr5	81170200	-
chr5	81971400	-
chr5	82128900	-
chr5	86527300	+
chr5	90265900	-
chr5	90271900	+
chr5	90434600	+
chr5	90494000	+
chr5	94523800	-
chr5	95990400	+
chr5	98339800	-
chr5	101959500	+
chr5	111916600	-
chr5	116142100	-
chr5	122802300	-
chr5	126111500	+
chr5	126452300	+
chr5	129135400	-
chr5	131219100	+
chr5	132608000	-
chr5	132695100	-
chr5	132787400	-
chr5	137650300	+
chr5	142807000	-
chr5	142943100	+
chr5	143502000	-
chr5	145785800	-
chr5	146424500	+
chr5	148922500	+
chr5	149006600	+
chr5	149084800	+
chr5	149710500	+
chr5	151023100	-
chr5	151849700	+
chr5	151887300	-
chr5	154510000	+
chr5	156252300	-
chr5	156302000	-
chr5	157278700	-
chr5	158216400	-
chr5	158370400	+
chr5	159621300	+
chr5	162827400	+
chr5	162838200	-
chr5	162849500	+
chr5	165728300	+
chr5	167082900	+
chr5	167587400	+
chr5	167742700	+
chr5	167973200	-
chr5	169775900	-
chr5	172048700	-
chr5	173779300	-
chr5	174824500	-
chr5	176476700	-
chr6	3154400	+
chr6	7592600	+
chr6	10792300	+
chr6	11062700	-
chr6	11568700	-
chr6	16069900	-
chr6	16910100	+
chr6	20400800	+
chr6	21955600	-
chr6	23065600	+
chr6	24851200	-
chr6	25332300	-
chr6	28379500	-
chr6	41257600	+
chr6	46154500	-
chr6	47726600	+
chr6	47804600	+
chr6	52106600	-
chr6	52134700	+
chr6	53204700	+
chr6	53399200	+
chr6	53597000	+
chr6	56566900	-
chr6	69508200	-
chr6	74480200	+
chr6	74489400	-
chr6	74499700	+
chr6	74513600	-
chr6	74622700	-
chr6	76346500	-
chr6	80782200	+
chr6	80796400	-
chr6	83038700	-
chr6	83981900	-
chr6	86228100	+
chr6	88485200	+
chr6	88497500	-
chr6	88508800	-
chr6	88592000	+
chr6	88699400	-
chr6	89817700	-
chr6	100783800	-
chr6	106008100	+
chr6	107003000	-
chr6	109664600	+
chr6	110448600	+
chr6	110817800	+
chr6	110966400	+
chr6	112630900	-
chr6	114236500	-
chr6	117959400	+
chr6	118615000	+
chr6	119415200	-
chr6	119473400	-
chr6	119508000	-
chr6	119830200	-
chr6	120105900	-
chr6	123114700	-
chr6	124019000	-
chr6	125251800	+
chr6	125720700	+
chr6	128841400	-
chr6	129646700	+
chr6	129733300	+
chr6	130094600	+
chr6	130270600	-
chr6	130880800	+
chr6	130948800	-
chr6	131814800	-
chr6	131882800	-
chr6	134742600	-
chr6	134882800	-
chr6	134993800	-
chr6	135512000	+
chr6	135559800	+
chr6	135940200	+
chr6	136161400	+
chr6	136873500	-
chr6	137534200	+
chr6	137899500	+
chr6	138002300	-
chr6	138313000	-
chr6	139582600	-
chr6	139853400	-
chr6	139878000	-
chr6	142209200	+
chr6	143891000	+
chr6	144399400	-
chr6	144493400	+
chr6	145576700	-
chr6	147148000	-
chr6	147220800	-
chr6	149558200	-
chr6	152039000	-
chr6	152065500	+
chr6	152120800	-
chr6	155046900	-
chr6	155548600	-
chr6	155586900	+
chr6	155696800	-
chr6	163074400	+
chr6	166218500	+
chr6	169047900	+
chr7	7423000	-
chr7	13063900	+
chr7	16899200	-
chr7	17131700	-
chr7	17165700	+
chr7	17408900	+
chr7	17958400	+
chr7	18361800	-
chr7	20223600	-
chr7	21268400	+
chr7	21389900	-
chr7	22408500	-
chr7	22431900	-
chr7	23577500	-
chr7	24042200	-
chr7	25566400	-
chr7	26285900	+
chr7	27037800	+
chr7	28246400	-
chr7	29831800	+
chr7	30256600	+
chr7	30708800	-
chr7	31140300	+
chr7	34003100	+
chr7	36411800	+
chr7	36418100	-
chr7	36425600	+
chr7	36684400	+
chr7	38419600	+
chr7	39362100	-
chr7	43310700	-
chr7	45929500	-
chr7	47653600	-
chr7	50261700	+
chr7	51176100	-
chr7	55030800	-
chr7	55693500	-
chr7	55919700	-
chr7	68799000	+
chr7	86112100	+
chr7	92484000	+
chr7	92504200	-
chr7	94802100	-
chr7	94943200	+
chr7	94979700	-
chr7	95963300	-
chr7	99225000	-
chr7	100895900	-
chr7	105853100	-
chr7	106032700	-
chr7	106144100	-
chr7	106158800	-
chr7	107303200	+
chr7	107563400	+
chr7	110333100	+
chr7	110421400	+
chr7	111565500	+
chr7	112226600	-
chr7	112849400	-
chr7	114211200	-
chr7	114786900	+
chr7	120220300	+
chr7	120868900	-
chr7	120948400	-
chr7	126750400	-
chr7	128896200	-
chr7	134034100	+
chr7	134590000	-
chr7	134618100	+
chr7	138217400	+
chr7	139993000	+
chr7	140399700	+
chr7	142900900	+
chr7	147809100	-
chr7	147892400	-
chr7	149949000	+
chr7	154199700	-
chr7	158553700	+
chr8	10856500	+
chr8	17516300	+
chr8	17636600	-
chr8	22895300	-
chr8	25388300	-
chr8	28388900	-
chr8	29321700	+
chr8	31226400	+
chr8	37393600	-
chr8	38058400	-
chr8	42715100	-
chr8	42778400	-
chr8	47156700	+
chr8	49863800	+
chr8	53062400	-
chr8	53076100	-
chr8	54046300	-
chr8	54768000	+
chr8	55228900	-
chr8	58187300	-
chr8	58327300	+
chr8	59470800	-
chr8	59789800	-
chr8	60994100	+
chr8	61124500	+
chr8	67090400	-
chr8	68677800	-
chr8	68820700	+
chr8	69043500	+
chr8	71552800	+
chr8	71749900	-
chr8	74069400	+
chr8	74244700	+
chr8	78193600	-
chr8	79580000	-
chr8	81305700	+
chr8	81451400	-
chr8	82855500	+
chr8	86602800	+
chr8	86696300	-
chr8	86737700	-
chr8	95522100	-
chr8	102414800	+
chr8	103617000	+
chr8	103638700	+
chr8	105600100	+
chr8	105870200	+
chr8	107847300	-
chr8	108614700	+
chr8	116157900	+
chr8	117529900	-
chr8	121063900	+
chr8	121998900	+
chr8	122090100	-
chr8	126782500	-
chr8	129306500	-
chr8	129475400	-
chr8	129710700	+
chr8	130143100	+
chr8	130497200	+
chr8	131831500	-
chr8	135801400	-
chr8	140017600	+
chr9	883800	+
chr9	1866400	-
chr9	2645400	+
chr9	3722100	+
chr9	4926400	+
chr9	5522000	+
chr9	6347200	-
chr9	6557000	+
chr9	7461300	-
chr9	7563500	+
chr9	7604200	-
chr9	7745700	-
chr9	7862100	-
chr9	19174000	+
chr9	19988900	-
chr9	20005200	+
chr9	20246600	-
chr9	21135100	-
chr9	21566700	-
chr9	22094900	+
chr9	22407400	-
chr9	31227600	+
chr9	31293300	-
chr9	31380900	-
chr9	31415800	-
chr9	31498600	+
chr9	31531900	+
chr9	31558800	+
chr9	34063700	+
chr9	36494200	-
chr9	36628700	+
chr9	70365100	+
chr9	70784300	+
chr9	71573800	+
chr9	74898800	-
chr9	78208200	+
chr9	78448000	+
chr9	79281300	+
chr9	86120400	-
chr9	86679500	-
chr9	88289900	+
chr9	89250700	-
chr9	89617700	-
chr9	93936300	-
chr9	94180900	+
chr9	95956100	+
chr9	96483200	-
chr9	100194200	-
chr9	100986800	+
chr9	101235800	+
chr9	103404500	+
chr9	105832000	-
chr9	109046000	+
chr9	110799700	+
chr9	111213500	+
chr9	111740000	+
chr9	111753800	-
chr9	111991500	-
chr9	116538500	-
chr9	116838300	+
chr9	123550700	-
chr9	132911500	+
chrX	9828100	-
chrX	9939800	-
chrX	12821700	+
chrX	15070700	-
chrX	15132300	+
chrX	15198200	-
chrX	22064200	+
chrX	39236200	-
chrX	40283300	+
chrX	43301700	+
chrX	43717400	+
chrX	46055400	-
chrX	56426200	-
chrX	65183000	-
chrX	68031800	+
chrX	68675300	-
chrX	69438900	-
chrX	71757400	-
chrX	73488200	+
chrX	78226900	+
chrX	96781500	-
chrX	100349900	+
chrX	110228700	+
chrX	119771300	+
chrX	119808800	-
chrX	120123000	-
chrX	120270200	-
chrX	123185700	-
chrX	123694900	+
chrX	124256500	+
chrX	127742500	-
chrX	130638800	+
chrX	132684500	+
chrX	147223000	-
chrX	151732800	-
chrX	153709000	-
chrX	153862100	+
chrY	21256500	+

Cluster GW7 (in hg18)
chr1	6188200	+
chr1	6330900	+
chr1	6480700	-
chr1	6537300	-
chr1	7663300	+
chr1	7936700	+
chr1	8508800	-
chr1	8800100	-
chr1	8953100	+
chr1	9925800	-
chr1	10413200	-
chr1	10922600	+
chr1	11245300	-
chr1	11701800	+
chr1	11891700	-
chr1	11917100	+
chr1	13899200	+
chr1	15802800	-
chr1	15896600	-
chr1	16435900	-
chr1	16620400	-
chr1	16698200	+
chr1	17088800	-
chr1	17095300	+
chr1	17103300	+
chr1	17554900	+
chr1	19998800	+
chr1	20385200	+
chr1	20448800	-
chr1	20521700	-
chr1	20693600	-
chr1	20932200	-
chr1	21639300	+
chr1	23683300	+
chr1	23819000	-
chr1	23826200	+
chr1	23834300	-
chr1	24024500	-
chr1	24067300	-
chr1	24158800	+
chr1	24386000	-
chr1	24944500	-
chr1	26122000	-
chr1	26189300	-
chr1	26197000	-
chr1	26234500	+
chr1	26505600	+
chr1	26631400	+
chr1	26819800	+
chr1	26891600	+
chr1	26943600	+
chr1	26987400	-
chr1	27559800	-
chr1	27858900	+
chr1	27871400	-
chr1	27925200	+
chr1	28113700	-
chr1	28448100	-
chr1	28528200	+
chr1	28568800	+
chr1	28752100	+
chr1	28842300	-
chr1	28974700	-
chr1	29429600	+
chr1	31400700	-
chr1	31619400	-
chr1	31639900	+
chr1	32026900	-
chr1	32182700	-
chr1	32478800	+
chr1	32575500	-
chr1	33219800	-
chr1	33337900	-
chr1	33365400	+
chr1	33711100	-
chr1	35104900	-
chr1	35269500	-
chr1	35317400	+
chr1	35507200	+
chr1	35744500	+
chr1	35946300	-
chr1	36007600	+
chr1	36046500	+
chr1	36558900	+
chr1	36688500	+
chr1	38185000	+
chr1	38233600	+
chr1	38242400	+
chr1	39343700	+
chr1	39443600	-
chr1	40335800	-
chr1	40715700	+
chr1	40769600	+
chr1	41480400	+
chr1	42130200	-
chr1	42273900	-
chr1	42701700	-
chr1	43054900	+
chr1	44230000	-
chr1	44451500	+
chr1	44912800	-
chr1	44962300	-
chr1	44978200	-
chr1	45024900	-
chr1	45058100	+
chr1	45289700	-
chr1	45444600	+
chr1	45542400	-
chr1	45729100	-
chr1	45738100	+
chr1	45988700	+
chr1	46041900	+
chr1	46371100	+
chr1	46405500	-
chr1	46485400	+
chr1	46579000	+
chr1	46681500	-
chr1	47552400	-
chr1	48710600	-
chr1	51215800	-
chr1	51967900	-
chr1	52380700	-
chr1	52936400	+
chr1	52942100	-
chr1	53165900	-
chr1	53435000	+
chr1	53458800	-
chr1	53606400	-
chr1	54128200	-
chr1	54713300	+
chr1	54727600	-
chr1	54780800	-
chr1	55002800	-
chr1	58784700	-
chr1	59141900	+
chr1	59385000	+
chr1	59534900	+
chr1	60052900	+
chr1	61315600	-
chr1	61879500	+
chr1	61980600	+
chr1	62926400	+
chr1	63022300	+
chr1	63605700	+
chr1	63761600	-
chr1	64983200	+
chr1	65065100	+
chr1	65492900	-
chr1	66485400	-
chr1	66539600	+
chr1	67008700	-
chr1	67292100	-
chr1	67975000	-
chr1	70649200	+
chr1	74436200	+
chr1	75962800	+
chr1	76312800	+
chr1	76392300	+
chr1	77457900	-
chr1	77521400	-
chr1	78381100	+
chr1	78858400	+
chr1	78889200	+
chr1	84316400	-
chr1	84329500	-
chr1	84540600	+
chr1	84676000	-
chr1	84812600	-
chr1	84858700	+
chr1	86741500	+
chr1	88922300	+
chr1	89230600	-
chr1	89303300	-
chr1	90001400	-
chr1	90081900	+
chr1	90233200	+
chr1	91739100	+
chr1	91787200	-
chr1	92318400	+
chr1	92537200	+
chr1	93023000	+
chr1	93317300	+
chr1	94147500	-
chr1	94623000	-
chr1	95164500	+
chr1	95310900	+
chr1	96960100	+
chr1	98159200	-
chr1	100088100	+
chr1	100487900	-
chr1	101264000	+
chr1	108129900	+
chr1	109005900	-
chr1	109386100	-
chr1	109420400	-
chr1	109457600	+
chr1	109627400	+
chr1	109770400	-
chr1	110000000	+
chr1	110975100	+
chr1	111570200	+
chr1	112740100	+
chr1	112810800	-
chr1	112853200	-
chr1	113059700	-
chr1	114103400	-
chr1	114156200	-
chr1	114216300	-
chr1	114291200	-
chr1	114690700	-
chr1	115014100	+
chr1	115443300	+
chr1	116350900	-
chr1	116496500	-
chr1	116512800	-
chr1	116669800	+
chr1	116762600	-
chr1	117145200	-
chr1	117222100	+
chr1	117331200	+
chr1	117404000	+
chr1	117465800	+
chr1	118079200	-
chr1	119484300	+
chr1	120056600	-
chr1	120962600	-
chr1	144094100	-
chr1	144167600	-
chr1	144181300	+
chr1	144273800	+
chr1	144300700	-
chr1	145023100	-
chr1	145110300	+
chr1	145164100	-
chr1	145181000	+
chr1	145479900	+
chr1	146202400	+
chr1	146256900	+
chr1	146273200	+
chr1	148138200	-
chr1	148156100	-
chr1	148249000	-
chr1	148305900	+
chr1	148349300	-
chr1	148521200	-
chr1	148532800	+
chr1	148726400	+
chr1	148754800	-
chr1	149004500	-
chr1	149115400	-
chr1	149165100	+
chr1	149245000	+
chr1	149310200	-
chr1	149370000	+
chr1	149385700	-
chr1	149493800	+
chr1	149713000	-
chr1	149750500	+
chr1	149779600	-
chr1	150002700	-
chr1	150030100	-
chr1	150287400	-
chr1	150428300	+
chr1	150699600	-
chr1	151967400	+
chr1	152625400	-
chr1	152866600	-
chr1	152986700	-
chr1	153176100	-
chr1	153463900	-
chr1	153480800	+
chr1	153873300	+
chr1	154096200	-
chr1	154397400	+
chr1	154693200	+
chr1	154818300	+
chr1	154838000	-
chr1	154977700	-
chr1	155200100	-
chr1	156386300	+
chr1	157168200	+
chr1	157304300	+
chr1	157313700	-
chr1	158091000	+
chr1	158498700	-
chr1	158901800	-
chr1	159003800	+
chr1	159074900	-
chr1	159249600	+
chr1	159390400	+
chr1	159402800	+
chr1	159626500	+
chr1	159636000	+
chr1	160171100	-
chr1	161558300	-
chr1	163660200	+
chr1	163833800	+
chr1	163866900	+
chr1	163934400	-
chr1	164064100	+
chr1	164726100	-
chr1	165211400	-
chr1	165279400	+
chr1	165325900	-
chr1	165357800	-
chr1	165455600	+
chr1	165853400	-
chr1	165950000	+
chr1	165957800	+
chr1	166372600	-
chr1	166675100	+
chr1	166702700	+
chr1	166749300	+
chr1	167342500	+
chr1	167603800	+
chr1	167721700	-
chr1	168030700	-
chr1	168310400	-
chr1	170077500	-
chr1	170894600	+
chr1	171054900	-
chr1	171131300	-
chr1	172060300	-
chr1	172257800	-
chr1	173036200	-
chr1	173200700	+
chr1	173444700	+
chr1	176778400	-
chr1	177601600	+
chr1	178051100	-
chr1	178067900	-
chr1	178867700	+
chr1	179298900	-
chr1	179340900	-
chr1	180265600	-
chr1	180824800	-
chr1	181025300	+
chr1	181871600	-
chr1	182124400	+
chr1	182727300	+
chr1	183393200	-
chr1	183929700	+
chr1	184853800	+
chr1	190845200	-
chr1	191044700	-
chr1	191776200	+
chr1	195382500	-
chr1	196759100	+
chr1	196857600	-
chr1	197155300	+
chr1	197173400	-
chr1	198537900	+
chr1	198587600	+
chr1	198710100	-
chr1	198767200	-
chr1	198856800	-
chr1	198905800	-
chr1	198975000	+
chr1	199098500	+
chr1	199124000	+
chr1	199390100	+
chr1	199406700	+
chr1	199717300	-
chr1	199975600	+
chr1	200577100	+
chr1	201047000	-
chr1	201162900	-
chr1	201262100	+
chr1	201563300	-
chr1	201886100	-
chr1	202000800	+
chr1	202523100	-
chr1	202598700	+
chr1	202618800	-
chr1	202743000	+
chr1	203464100	+
chr1	203676600	+
chr1	203740300	+
chr1	203828000	+
chr1	204010800	-
chr1	204085300	+
chr1	204875400	-
chr1	206123100	+
chr1	206203600	-
chr1	206326000	+
chr1	206484500	-
chr1	208045900	+
chr1	208568800	+
chr1	209754900	+
chr1	209915200	+
chr1	209979500	-
chr1	210070400	+
chr1	210179000	-
chr1	210275500	-
chr1	210798600	+
chr1	210807000	-
chr1	210906000	-
chr1	211098200	+
chr1	212550400	-
chr1	212679100	+
chr1	212843500	-
chr1	213807400	+
chr1	215871100	-
chr1	216585600	+
chr1	217414000	+
chr1	220706000	-
chr1	220829600	+
chr1	220884000	+
chr1	221054800	+
chr1	221383000	+
chr1	221604500	-
chr1	223722500	-
chr1	224337800	-
chr1	224364600	+
chr1	224563400	-
chr1	225137000	+
chr1	225817900	+
chr1	226633000	-
chr1	226718800	+
chr1	226755200	+
chr1	226849900	+
chr1	226938300	+
chr1	227006700	+
chr1	227341600	+
chr1	227357600	+
chr1	227544900	-
chr1	229071000	-
chr1	229414400	-
chr1	229443500	+
chr1	229806800	-
chr1	229829400	+
chr1	230780900	+
chr1	231153000	+
chr1	231498000	-
chr1	232528600	+
chr1	232603800	-
chr1	232681200	-
chr1	233214200	-
chr1	233597000	+
chr1	233734400	-
chr1	233872600	-
chr1	234096800	-
chr1	234117600	+
chr1	234350400	+
chr1	234754200	+
chr1	235025500	+
chr1	238044900	-
chr1	239749400	-
chr1	239781600	+
chr1	239870300	-
chr1	239906900	+
chr1	240077700	+
chr1	242081300	-
chr1	242304700	+
chr1	242529900	-
chr1	242691500	-
chr1	242960100	-
chr1	243541200	-
chr1	244183300	+
chr1	244736900	+
chr1	244954400	+
chr1	245308600	-
chr1	245646100	+
chr1	246166600	+
chr1	247123600	+
chr1	247134600	-
chr10	3505100	-
chr10	3655500	+
chr10	3797500	-
chr10	3861000	+
chr10	3968000	-
chr10	4066700	-
chr10	4106600	+
chr10	5323500	+
chr10	5444900	-
chr10	5479000	+
chr10	5748300	+
chr10	6059200	-
chr10	6222700	+
chr10	6382500	-
chr10	6430200	-
chr10	6625200	-
chr10	7009400	+
chr10	7554300	-
chr10	8219200	+
chr10	8414200	-
chr10	11231200	+
chr10	11693400	-
chr10	11766700	+
chr10	11945800	-
chr10	12688300	-
chr10	13243800	-
chr10	13381800	+
chr10	13431200	-
chr10	14730400	+
chr10	14741600	+
chr10	15041600	+
chr10	15291600	-
chr10	15336600	-
chr10	17283600	-
chr10	17510800	-
chr10	17699100	+
chr10	18980300	-
chr10	21823400	+
chr10	21855000	-
chr10	21863100	+
chr10	22352100	-
chr10	22558700	-
chr10	22766100	+
chr10	23425200	+
chr10	23673800	-
chr10	23692200	+
chr10	24792400	+
chr10	24848600	+
chr10	25053600	-
chr10	25281300	-
chr10	25345500	+
chr10	27026700	+
chr10	27429500	+
chr10	28507200	+
chr10	28632000	-
chr10	29738400	+
chr10	31034100	-
chr10	31113800	-
chr10	31360800	-
chr10	31463200	-
chr10	31932100	+
chr10	32089000	-
chr10	32775300	-
chr10	33309900	-
chr10	35456000	+
chr10	35478700	-
chr10	35524600	-
chr10	38186700	-
chr10	38305300	-
chr10	38339500	+
chr10	42453900	-
chr10	42920600	+
chr10	43177800	-
chr10	43236000	+
chr10	43252500	-
chr10	43271200	-
chr10	43411000	-
chr10	43421800	+
chr10	43505000	+
chr10	44694900	-
chr10	45235000	+
chr10	45396500	-
chr10	47126300	-
chr10	48151400	+
chr10	48194000	-
chr10	51159400	-
chr10	52169500	+
chr10	57791000	-
chr10	61667600	+
chr10	64067500	-
chr10	64563300	+
chr10	65060000	-
chr10	65471800	-
chr10	69279700	-
chr10	69901100	+
chr10	69957300	-
chr10	70029900	+
chr10	70151200	+
chr10	70881200	+
chr10	71060100	+
chr10	71522600	+
chr10	71662800	-
chr10	71988800	+
chr10	72245600	+
chr10	72318100	-
chr10	72807400	-
chr10	73539800	-
chr10	73678400	-
chr10	73690500	+
chr10	74055800	-
chr10	74122100	+
chr10	74526200	-
chr10	74788700	-
chr10	75055500	+
chr10	75077000	+
chr10	75159800	+
chr10	77201800	+
chr10	79732700	+
chr10	80403700	-
chr10	81828500	+
chr10	82158100	+
chr10	82285900	+
chr10	88150300	+
chr10	88286900	-
chr10	88461300	-
chr10	88506400	+
chr10	90135700	+
chr10	90725300	-
chr10	90913600	+
chr10	91001500	-
chr10	91051600	+
chr10	91142300	+
chr10	91394400	+
chr10	91451000	+
chr10	91587600	-
chr10	91611100	-
chr10	92951700	-
chr10	93159700	+
chr10	94342300	-
chr10	94598500	-
chr10	95246000	+
chr10	95252400	+
chr10	95452300	+
chr10	95644000	+
chr10	96112100	-
chr10	96295600	+
chr10	97044800	+
chr10	97310900	-
chr10	97443500	-
chr10	97657300	-
chr10	97793600	+
chr10	98470300	-
chr10	98788100	-
chr10	98945800	+
chr10	99042200	-
chr10	99383300	-
chr10	99463300	+
chr10	100217400	+
chr10	101409000	+
chr10	101759700	-
chr10	102123400	-
chr10	104211200	-
chr10	104423800	+
chr10	104603700	+
chr10	104668000	+
chr10	104804200	-
chr10	105100700	-
chr10	105118000	+
chr10	105334500	-
chr10	105521800	+
chr10	105871600	-
chr10	105982100	-
chr10	111684200	+
chr10	112473900	+
chr10	113933600	-
chr10	115132100	-
chr10	115604300	-
chr10	118925100	-
chr10	119580900	-
chr10	120915300	-
chr10	120958600	-
chr10	122600800	+
chr10	124124300	+
chr10	124704300	-
chr10	125814100	-
chr10	126033100	+
chr10	126067800	-
chr10	126097300	+
chr10	129678800	+
chr10	129814400	-
chr10	129869900	-
chr10	129876200	-
chr10	131155400	+
chr10	134512100	-
chr10	134626100	-
chr10	135052600	+
chr11	258600	+
chr11	1360300	+
chr11	2870500	-
chr11	5602600	+
chr11	7491500	+
chr11	8667200	-
chr11	8889200	-
chr11	9243000	-
chr11	9342100	+
chr11	9579200	+
chr11	9592200	-
chr11	9641600	+
chr11	9736800	-
chr11	10272300	-
chr11	10519100	-
chr11	11820200	+
chr11	12355700	+
chr11	13186400	-
chr11	13465600	+
chr11	14054000	-
chr11	14181000	-
chr11	14869900	-
chr11	14883300	+
chr11	17254600	+
chr11	17367600	-
chr11	18084100	-
chr11	18361800	+
chr11	18566400	+
chr11	18676600	+
chr11	18699800	-
chr11	20365700	+
chr11	27341500	-
chr11	27450300	-
chr11	28085600	+
chr11	30301800	-
chr11	31348300	-
chr11	31487900	-
chr11	31973400	+
chr11	32069400	+
chr11	32871300	+
chr11	32993800	+
chr11	33236000	+
chr11	33678900	+
chr11	36267100	+
chr11	36339400	+
chr11	43658900	+
chr11	44044800	-
chr11	44074400	-
chr11	44575500	-
chr11	44658900	+
chr11	44928400	-
chr11	45125100	+
chr11	45749800	+
chr11	46078200	+
chr11	46096900	+
chr11	46216300	+
chr11	46538500	+
chr11	46805200	-
chr11	46915000	-
chr11	47693000	+
chr11	57039200	-
chr11	57162900	+
chr11	58631100	-
chr11	58696300	+
chr11	59074400	+
chr11	59085100	-
chr11	59334900	-
chr11	59971200	-
chr11	60566700	-
chr11	60607500	-
chr11	60615100	+
chr11	60916800	-
chr11	61219800	+
chr11	61339800	+
chr11	61550200	-
chr11	61861400	+
chr11	61926600	-
chr11	62145700	-
chr11	62233800	-
chr11	62295500	-
chr11	62366200	-
chr11	63098600	+
chr11	63137800	+
chr11	63291500	+
chr11	63689600	+
chr11	64495500	+
chr11	64564900	+
chr11	64786100	+
chr11	65001300	+
chr11	65651400	-
chr11	65962900	-
chr11	66034600	+
chr11	66092200	+
chr11	66268800	+
chr11	66841900	-
chr11	67138100	+
chr11	67645800	-
chr11	69165900	-
chr11	71501400	-
chr11	71907900	+
chr11	71973400	+
chr11	72182100	+
chr11	72696300	+
chr11	73176600	+
chr11	73787200	-
chr11	73981600	+
chr11	74377400	+
chr11	74972000	-
chr11	75541500	-
chr11	76075600	-
chr11	76249600	+
chr11	76515700	+
chr11	76536800	-
chr11	76800400	-
chr11	76863000	-
chr11	76978300	+
chr11	77528100	+
chr11	77591400	-
chr11	77806300	-
chr11	81259100	-
chr11	82146200	-
chr11	82289900	-
chr11	85036400	+
chr11	85243800	-
chr11	85633700	+
chr11	85691000	+
chr11	86060700	-
chr11	87710900	-
chr11	92570600	-
chr11	92915700	+
chr11	93034400	+
chr11	94523600	+
chr11	94605600	-
chr11	94626800	+
chr11	94710200	+
chr11	95296600	+
chr11	95640800	-
chr11	101291300	-
chr11	101644400	+
chr11	101723100	+
chr11	102467800	-
chr11	102485200	+
chr11	104294200	-
chr11	104344200	-
chr11	104410700	-
chr11	104446900	-
chr11	104476900	-
chr11	106941700	-
chr11	107234800	-
chr11	107842900	+
chr11	107874000	-
chr11	107968900	+
chr11	108230400	-
chr11	110755500	-
chr11	111247300	-
chr11	111255100	-
chr11	111302900	+
chr11	111312600	+
chr11	111401100	+
chr11	111450100	+
chr11	111655700	+
chr11	112690700	-
chr11	113149900	-
chr11	113436400	-
chr11	113716100	+
chr11	114570700	-
chr11	116212000	-
chr11	116306700	+
chr11	117600200	+
chr11	117628200	-
chr11	117640400	-
chr11	117680400	+
chr11	117941800	-
chr11	118217100	+
chr11	118246200	+
chr11	118544800	+
chr11	118697000	+
chr11	118757600	-
chr11	119105200	-
chr11	119587100	+
chr11	121031400	+
chr11	121956700	+
chr11	122066500	+
chr11	122073400	-
chr11	122103900	-
chr11	122228600	-
chr11	122258700	+
chr11	122609800	-
chr11	122678500	-
chr11	124048600	-
chr11	124251600	+
chr11	124329500	-
chr11	124486700	-
chr11	125001100	-
chr11	125679200	+
chr11	125792300	-
chr11	127411200	+
chr11	127429300	-
chr11	127797500	+
chr11	127923600	+
chr11	128280600	+
chr11	129071200	-
chr11	129228500	+
chr11	129764300	-
chr11	133706700	+
chr12	380800	+
chr12	439600	-
chr12	556000	-
chr12	622700	+
chr12	642300	+
chr12	970500	-
chr12	1183800	+
chr12	1670500	+
chr12	2043200	+
chr12	2670900	-
chr12	3056500	+
chr12	3852000	-
chr12	3884900	+
chr12	3968700	-
chr12	4007500	-
chr12	4122700	-
chr12	4144000	+
chr12	4300500	+
chr12	4584100	+
chr12	6424300	+
chr12	6611100	-
chr12	6808100	+
chr12	6870900	-
chr12	6884400	-
chr12	8004200	+
chr12	8014400	+
chr12	8034600	-
chr12	8070500	+
chr12	9409200	-
chr12	9492400	-
chr12	9857400	+
chr12	10198000	-
chr12	10256900	+
chr12	10409000	-
chr12	10657400	-
chr12	11590400	-
chr12	11599800	+
chr12	12077400	-
chr12	12115600	+
chr12	12311100	-
chr12	12656200	+
chr12	12740400	+
chr12	12831500	-
chr12	13088900	-
chr12	13145900	-
chr12	14261300	-
chr12	14430900	-
chr12	15955700	-
chr12	22453500	+
chr12	24839600	+
chr12	25096200	+
chr12	25239300	+
chr12	25430100	+
chr12	27288800	+
chr12	27568000	+
chr12	27866000	+
chr12	29148400	-
chr12	29193500	-
chr12	30866700	+
chr12	31118000	+
chr12	31703200	+
chr12	31773300	-
chr12	32151100	+
chr12	32183600	+
chr12	34066600	+
chr12	36996600	+
chr12	37586100	-
chr12	40778500	-
chr12	42516200	-
chr12	43896100	+
chr12	44350000	-
chr12	45063500	+
chr12	45204400	-
chr12	46041000	+
chr12	46562800	+
chr12	46643800	+
chr12	46799100	+
chr12	46878300	+
chr12	47545800	-
chr12	47598500	-
chr12	47749900	-
chr12	47868700	-
chr12	48002900	+
chr12	48047200	-
chr12	48218500	+
chr12	48302400	+
chr12	48625500	-
chr12	48705500	-
chr12	48963400	+
chr12	49728300	+
chr12	49898000	+
chr12	49950300	+
chr12	50009400	-
chr12	50075000	-
chr12	50105300	-
chr12	50500800	+
chr12	50631700	+
chr12	51310400	+
chr12	51371200	+
chr12	51948300	+
chr12	52004900	-
chr12	52025100	-
chr12	52051500	-
chr12	52121900	+
chr12	53005000	+
chr12	53039100	-
chr12	53048200	+
chr12	53098500	-
chr12	53240600	+
chr12	53672000	-
chr12	54327000	-
chr12	54418000	+
chr12	54687700	+
chr12	54701800	-
chr12	54759800	+
chr12	54832500	-
chr12	54946800	+
chr12	55129700	-
chr12	55168300	-
chr12	55202200	-
chr12	55758800	-
chr12	55807800	+
chr12	56110900	-
chr12	56139100	+
chr12	56285200	-
chr12	56621500	+
chr12	61283100	+
chr12	62460100	-
chr12	62902200	-
chr12	63132200	+
chr12	63264500	-
chr12	63439500	-
chr12	64421700	-
chr12	66797800	+
chr12	67012100	+
chr12	67044600	+
chr12	67131500	+
chr12	67426400	+
chr12	67488400	+
chr12	67613300	-
chr12	68011000	-
chr12	70366600	-
chr12	74070900	+
chr12	75266500	-
chr12	87060000	+
chr12	88780800	+
chr12	90480900	-
chr12	90696500	+
chr12	92660200	+
chr12	93020000	-
chr12	93126100	+
chr12	93180300	+
chr12	93377700	+
chr12	93539600	+
chr12	94860900	+
chr12	95637800	+
chr12	97315100	+
chr12	97421400	+
chr12	99060400	-
chr12	100197700	+
chr12	100615600	-
chr12	100748700	-
chr12	100795700	+
chr12	100979900	-
chr12	101037800	+
chr12	102819200	+
chr12	103134100	-
chr12	103876100	+
chr12	103904100	+
chr12	105275900	+
chr12	107609900	-
chr12	108076800	+
chr12	108354900	+
chr12	108495900	-
chr12	108995900	-
chr12	109002300	-
chr12	109536100	+
chr12	110291300	-
chr12	110916500	-
chr12	110924000	-
chr12	111900900	+
chr12	112075100	+
chr12	112143700	+
chr12	115198400	+
chr12	115456100	-
chr12	115481500	-
chr12	115740800	+
chr12	115833100	+
chr12	116021200	-
chr12	116938900	+
chr12	116984800	-
chr12	117025800	+
chr12	118799100	+
chr12	119214800	-
chr12	119451300	-
chr12	119503400	-
chr12	119938200	+
chr12	120055000	+
chr12	120132400	+
chr12	120163200	-
chr12	120503900	-
chr12	120944400	+
chr12	121295400	-
chr12	121317000	-
chr12	121577700	-
chr12	121825200	+
chr12	122509000	+
chr12	122721700	+
chr12	122988300	+
chr12	123763800	+
chr12	123825500	-
chr12	123914100	-
chr12	123972400	-
chr12	123990600	-
chr12	124100400	-
chr12	124115700	+
chr12	129931000	-
chr12	130156100	+
chr12	131974400	+
chr13	19059500	+
chr13	19590800	+
chr13	19704100	-
chr13	19948400	-
chr13	20039100	-
chr13	20175800	-
chr13	20770100	+
chr13	21146500	+
chr13	21533200	+
chr13	22938300	+
chr13	23167900	+
chr13	23361400	-
chr13	23438500	+
chr13	23632800	+
chr13	24394800	+
chr13	25484000	+
chr13	25659000	-
chr13	25726300	+
chr13	26194300	-
chr13	26742900	+
chr13	26825900	-
chr13	26922600	-
chr13	28190900	-
chr13	29375300	-
chr13	29426800	-
chr13	29779600	-
chr13	29848800	-
chr13	29867800	-
chr13	29894100	-
chr13	30045100	-
chr13	30146100	+
chr13	30246000	+
chr13	30268800	-
chr13	30672400	+
chr13	31503600	-
chr13	31787500	+
chr13	32058800	+
chr13	33015200	-
chr13	33083000	+
chr13	33151900	-
chr13	33259100	+
chr13	33290200	+
chr13	35818200	-
chr13	39127900	+
chr13	39429400	+
chr13	39498800	-
chr13	39861000	+
chr13	39911000	-
chr13	39966700	-
chr13	40261400	+
chr13	40393900	-
chr13	41433100	-
chr13	41512700	+
chr13	41520900	-
chr13	42046600	+
chr13	43351900	-
chr13	43614200	-
chr13	43731100	+
chr13	44390200	+
chr13	45683700	+
chr13	45793400	-
chr13	46139900	+
chr13	46151400	+
chr13	47693900	-
chr13	47964000	+
chr13	48004800	-
chr13	48126500	-
chr13	48289300	+
chr13	48448400	+
chr13	48582200	+
chr13	48720200	+
chr13	48774000	-
chr13	49101100	-
chr13	49408500	-
chr13	49625100	-
chr13	49708600	+
chr13	49827700	-
chr13	49984900	-
chr13	50365300	-
chr13	50382500	+
chr13	51236600	+
chr13	51414000	+
chr13	51601000	-
chr13	51631300	+
chr13	51667000	-
chr13	52089200	+
chr13	59636100	+
chr13	59740400	-
chr13	66702600	-
chr13	70763100	+
chr13	72531400	+
chr13	72992900	+
chr13	73238100	+
chr13	73703600	+
chr13	73760200	-
chr13	74798600	-
chr13	75235000	-
chr13	76358000	-
chr13	76464000	+
chr13	76951000	-
chr13	78865700	+
chr13	93778000	-
chr13	93999700	-
chr13	94052400	-
chr13	94310800	+
chr13	94751500	+
chr13	94853800	+
chr13	94929500	-
chr13	98764900	+
chr13	98914800	+
chr13	99056500	+
chr13	99108100	-
chr13	99956300	+
chr13	100038600	+
chr13	101078100	-
chr13	101845000	-
chr13	102224000	-
chr13	104590400	-
chr13	105986700	-
chr13	107720200	+
chr13	110012200	-
chr13	112290400	-
chr13	113096500	-
chr14	19870400	+
chr14	19881200	+
chr14	19951600	-
chr14	20148100	+
chr14	20163400	-
chr14	20319300	+
chr14	20509400	-
chr14	20641800	+
chr14	20729800	+
chr14	20846600	+
chr14	21049000	-
chr14	21064400	-
chr14	22137000	+
chr14	22368700	+
chr14	22496000	-
chr14	22521700	-
chr14	22545800	+
chr14	22560000	+
chr14	22659700	-
chr14	22721800	+
chr14	22825300	-
chr14	23171800	-
chr14	23620600	-
chr14	30959500	-
chr14	30996500	-
chr14	31100100	+
chr14	33557900	-
chr14	33599100	+
chr14	34001200	-
chr14	34253400	-
chr14	35472300	-
chr14	35859500	-
chr14	38641900	+
chr14	38653100	+
chr14	38709200	-
chr14	44436200	+
chr14	44791800	+
chr14	49135300	-
chr14	49229300	+
chr14	49507900	+
chr14	49514500	+
chr14	49540200	-
chr14	49598000	+
chr14	49618800	+
chr14	49652700	-
chr14	49900100	-
chr14	49933400	-
chr14	50204400	-
chr14	50396100	+
chr14	50776600	+
chr14	51188100	+
chr14	51383200	+
chr14	51757400	+
chr14	52277900	+
chr14	52754500	-
chr14	53933400	+
chr14	54104500	-
chr14	54164000	+
chr14	54267300	-
chr14	54418000	+
chr14	54666200	+
chr14	54728300	-
chr14	54869800	+
chr14	55344500	-
chr14	57932700	-
chr14	57964000	-
chr14	59501700	-
chr14	59701800	-
chr14	59784800	+
chr14	59868300	-
chr14	60271800	-
chr14	60517800	+
chr14	60714300	+
chr14	61105100	+
chr14	61199700	-
chr14	61426200	+
chr14	63178600	-
chr14	63875000	-
chr14	63924600	+
chr14	64245000	+
chr14	64523300	+
chr14	64640000	-
chr14	64742400	+
chr14	64766000	+
chr14	64818800	+
chr14	65474300	-
chr14	66777900	-
chr14	67051500	+
chr14	67156500	-
chr14	67356500	+
chr14	67747700	-
chr14	68044200	-
chr14	68164700	+
chr14	68200700	-
chr14	68727900	+
chr14	69263200	+
chr14	70408900	-
chr14	70422300	+
chr14	71092800	-
chr14	72013600	-
chr14	72563500	-
chr14	72994700	-
chr14	73128300	+
chr14	73149500	-
chr14	73170700	-
chr14	73181100	+
chr14	73388300	+
chr14	73486800	-
chr14	73555400	+
chr14	73621000	+
chr14	73839000	+
chr14	73938500	-
chr14	74029700	+
chr14	74154600	+
chr14	74478700	-
chr14	74587800	-
chr14	74600300	-
chr14	74606200	-
chr14	74795800	-
chr14	74831800	-
chr14	74870400	+
chr14	75050900	+
chr14	75197100	-
chr14	76297500	+
chr14	76569600	-
chr14	76576700	+
chr14	76661100	-
chr14	76856800	+
chr14	76912800	+
chr14	77178200	-
chr14	77336000	+
chr14	80466600	+
chr14	80477900	-
chr14	80496200	-
chr14	80706500	-
chr14	87529100	+
chr14	87921500	+
chr14	88328900	-
chr14	88360800	-
chr14	89237600	+
chr14	89260400	+
chr14	89868000	-
chr14	89919400	-
chr14	90294300	+
chr14	90364800	-
chr14	90559000	-
chr14	90650700	+
chr14	91110200	+
chr14	92049900	+
chr14	92113100	+
chr14	92284200	+
chr14	93506900	+
chr14	93563100	+
chr14	93665800	-
chr14	93710200	+
chr14	95060000	-
chr14	95928000	-
chr14	96128600	+
chr14	96755400	-
chr14	96915300	+
chr14	96995400	-
chr14	97139000	+
chr14	97170800	+
chr14	97672100	+
chr14	98049700	-
chr14	99219600	+
chr14	99728900	+
chr14	99757800	+
chr14	102060700	+
chr14	103144000	-
chr14	103408100	+
chr14	104214900	+
chr14	104364000	-
chr14	105757300	-
chr14	106034000	+
chr14	106323800	+
chr15	19888900	-
chr15	19950700	+
chr15	23878600	-
chr15	28983100	+
chr15	29295400	-
chr15	29514200	+
chr15	29950700	-
chr15	31170800	-
chr15	31206400	-
chr15	31274700	-
chr15	31281800	-
chr15	32118400	+
chr15	32398500	-
chr15	33625600	-
chr15	34658900	+
chr15	36330700	+
chr15	36732200	-
chr15	36775300	+
chr15	37862300	+
chr15	38013500	+
chr15	38240300	+
chr15	38673500	+
chr15	39032600	+
chr15	39411800	+
chr15	39482300	-
chr15	39701100	-
chr15	40574900	+
chr15	40655000	+
chr15	40661200	-
chr15	41000400	-
chr15	41202900	-
chr15	41410300	-
chr15	41450500	+
chr15	41572300	+
chr15	41590500	-
chr15	41826100	+
chr15	41907200	-
chr15	42743000	-
chr15	42808400	+
chr15	42814900	+
chr15	43102400	+
chr15	43358700	-
chr15	43601900	-
chr15	43784600	-
chr15	46257400	+
chr15	46890500	-
chr15	47125800	-
chr15	47249600	+
chr15	47700500	+
chr15	48327000	+
chr15	48844700	+
chr15	48987800	-
chr15	49830900	+
chr15	50051300	-
chr15	50301500	-
chr15	50758200	-
chr15	53347300	-
chr15	53369100	-
chr15	53399000	-
chr15	53578500	-
chr15	54073000	-
chr15	54122200	+
chr15	54966600	+
chr15	56330200	-
chr15	57184600	-
chr15	58206800	+
chr15	58799200	+
chr15	60146600	+
chr15	61127800	+
chr15	61201400	+
chr15	61356700	+
chr15	62460900	-
chr15	62466700	+
chr15	62714500	-
chr15	62904300	-
chr15	62974600	-
chr15	63383900	-
chr15	63464900	-
chr15	63691000	-
chr15	63871500	-
chr15	64061300	+
chr15	64373300	-
chr15	64436000	-
chr15	64780600	+
chr15	65621900	+
chr15	65919700	-
chr15	66834900	-
chr15	67240300	-
chr15	67542100	-
chr15	68221100	-
chr15	68614500	+
chr15	70197100	-
chr15	70765300	+
chr15	70876600	+
chr15	71131500	+
chr15	72045100	-
chr15	72453400	+
chr15	72540900	-
chr15	72775500	-
chr15	72805400	+
chr15	72986100	-
chr15	73102900	+
chr15	73704800	+
chr15	73726800	+
chr15	73818500	-
chr15	74366200	-
chr15	74984600	-
chr15	75150400	-
chr15	75622400	+
chr15	76586600	+
chr15	76645000	+
chr15	77024400	-
chr15	78003100	-
chr15	78050700	-
chr15	78232100	+
chr15	80126400	-
chr15	81146800	-
chr15	82945400	-
chr15	83674400	+
chr15	87257500	-
chr15	87587900	+
chr15	87920000	-
chr15	88034600	+
chr15	88095000	-
chr15	88238200	-
chr15	88529100	+
chr15	89338600	+
chr15	91077900	+
chr15	91163900	-
chr15	91176600	-
chr15	92575800	-
chr15	96305200	-
chr15	96961500	-
chr15	97808500	+
chr15	97843900	-
chr15	98064800	+
chr15	98286900	-
chr15	98707500	+
chr15	99207800	-
chr15	99365600	+
chr15	99678900	-
chr16	243400	-
chr16	2593200	+
chr16	2672300	+
chr16	3161300	+
chr16	3433400	+
chr16	3490700	+
chr16	4055700	-
chr16	4174100	-
chr16	5061700	+
chr16	11226400	+
chr16	11355800	+
chr16	11623700	-
chr16	11785000	-
chr16	12091600	-
chr16	13921500	+
chr16	14072800	-
chr16	14286900	+
chr16	14311100	-
chr16	14976600	+
chr16	15095800	-
chr16	15436000	+
chr16	17371700	+
chr16	17531300	+
chr16	17941200	-
chr16	18845000	-
chr16	18986600	+
chr16	19474300	+
chr16	19804400	-
chr16	20595100	-
chr16	20683100	-
chr16	20725400	+
chr16	20793400	-
chr16	21077800	-
chr16	21220300	-
chr16	22108100	+
chr16	23067900	+
chr16	24968000	+
chr16	24986000	-
chr16	27122700	-
chr16	27187600	-
chr16	28196200	+
chr16	28413400	+
chr16	28798800	-
chr16	29881000	-
chr16	30041700	-
chr16	30301200	-
chr16	30454200	-
chr16	30477200	-
chr16	30490400	+
chr16	30504500	+
chr16	30529000	-
chr16	30553000	+
chr16	30724700	-
chr16	30733500	-
chr16	30794300	+
chr16	30822800	-
chr16	30841200	+
chr16	30915700	+
chr16	31062100	-
chr16	45213000	-
chr16	45381400	+
chr16	45422400	-
chr16	45475900	-
chr16	45735600	-
chr16	46976400	-
chr16	47215300	-
chr16	48120200	-
chr16	48616700	+
chr16	49181200	-
chr16	49433000	-
chr16	51646300	+
chr16	51690700	+
chr16	51722100	+
chr16	51964500	+
chr16	52026500	+
chr16	54870900	-
chr16	54948600	-
chr16	55201000	-
chr16	55217300	-
chr16	55234700	+
chr16	55249500	-
chr16	55300600	-
chr16	55726300	-
chr16	55891800	-
chr16	56201100	+
chr16	56576300	+
chr16	56616500	+
chr16	56841600	-
chr16	57092300	+
chr16	57276100	-
chr16	65288000	-
chr16	65464000	+
chr16	65605300	+
chr16	66258400	+
chr16	66591700	-
chr16	67061600	-
chr16	67121700	-
chr16	67130900	+
chr16	67435100	+
chr16	68036000	-
chr16	68121700	+
chr16	68317800	+
chr16	68353700	+
chr16	68881000	-
chr16	68890700	+
chr16	69045900	+
chr16	70156600	-
chr16	70315000	-
chr16	70408900	+
chr16	71256400	-
chr16	71379000	+
chr16	73292100	-
chr16	73603600	+
chr16	73657000	-
chr16	73740100	+
chr16	74108500	-
chr16	74147500	-
chr16	74214300	-
chr16	76313800	+
chr16	77334400	-
chr16	77428600	-
chr16	77863700	-
chr16	77871000	-
chr16	77878600	-
chr16	78362200	-
chr16	79248300	-
chr16	79588900	-
chr16	79668100	+
chr16	79687400	-
chr16	80309400	+
chr16	80321300	+
chr16	80337100	+
chr16	81728700	-
chr16	83144400	+
chr16	83748600	+
chr16	84145000	+
chr16	84326600	-
chr16	84539100	-
chr16	85195600	+
chr16	86227300	-
chr16	86398000	-
chr16	88359200	-
chr16	88410200	+
chr16	88457800	-
chr16	88616600	-
chr17	267000	-
chr17	1478800	-
chr17	2065700	-
chr17	2899100	+
chr17	3573700	-
chr17	4283200	+
chr17	4336600	-
chr17	4589400	-
chr17	4875200	+
chr17	5036100	-
chr17	6288000	+
chr17	6866800	+
chr17	7393100	+
chr17	7766700	+
chr17	7834000	+
chr17	8044400	-
chr17	8475100	-
chr17	8589800	+
chr17	9083200	+
chr17	9420200	+
chr17	10574000	-
chr17	11841500	-
chr17	11864900	+
chr17	14272800	-
chr17	16130700	-
chr17	16197100	-
chr17	16380400	-
chr17	16497600	-
chr17	17435900	-
chr17	17508000	-
chr17	17804000	-
chr17	18207500	-
chr17	18699800	+
chr17	19492400	+
chr17	19568700	-
chr17	19711500	-
chr17	19720400	+
chr17	19853100	+
chr17	21119800	+
chr17	22683700	+
chr17	22705100	+
chr17	23156800	+
chr17	23244400	-
chr17	23669900	+
chr17	23950300	+
chr17	24062900	-
chr17	24499900	+
chr17	24532200	-
chr17	25951800	-
chr17	26059100	+
chr17	26183000	+
chr17	26257400	+
chr17	26322200	-
chr17	26359700	+
chr17	26446300	+
chr17	26901100	-
chr17	27358700	+
chr17	27478900	-
chr17	27692900	+
chr17	28228000	+
chr17	30512700	+
chr17	30665000	-
chr17	30697600	-
chr17	30725000	-
chr17	30783900	-
chr17	30799600	-
chr17	30838600	+
chr17	30891500	+
chr17	30929700	-
chr17	31230800	-
chr17	31282100	-
chr17	31965100	-
chr17	32152500	+
chr17	32840900	+
chr17	33043200	-
chr17	34263400	-
chr17	34467100	-
chr17	34563300	-
chr17	34648800	-
chr17	35270800	+
chr17	35277700	-
chr17	35463800	+
chr17	35697800	-
chr17	35813100	-
chr17	35827500	+
chr17	35853500	+
chr17	36229200	-
chr17	37058000	+
chr17	37076300	-
chr17	37089100	-
chr17	37245600	-
chr17	37340300	-
chr17	37455200	+
chr17	37828400	-
chr17	38064400	+
chr17	38178700	+
chr17	38719300	+
chr17	38748200	+
chr17	38793700	+
chr17	38801600	-
chr17	39095400	-
chr17	39153300	+
chr17	39212100	-
chr17	39350700	-
chr17	39360800	-
chr17	39503500	+
chr17	40122300	+
chr17	40141400	+
chr17	40380600	+
chr17	40484400	-
chr17	40606000	-
chr17	41017900	-
chr17	41328000	+
chr17	41700500	-
chr17	42532600	-
chr17	42637000	+
chr17	42756400	+
chr17	43082100	-
chr17	43381700	+
chr17	43457400	-
chr17	44324800	+
chr17	44625400	-
chr17	44662900	+
chr17	44692800	-
chr17	45110100	-
chr17	45120600	+
chr17	45283700	-
chr17	45601500	+
chr17	45858700	-
chr17	45940900	+
chr17	46376100	+
chr17	46462000	+
chr17	50671000	-
chr17	50853600	-
chr17	51009900	-
chr17	51183500	+
chr17	52212300	+
chr17	52410600	-
chr17	53378900	+
chr17	53589800	-
chr17	53651600	+
chr17	54064600	-
chr17	54091100	+
chr17	54125000	+
chr17	54587200	-
chr17	54652600	+
chr17	54806000	-
chr17	55219200	-
chr17	55396800	-
chr17	55510700	-
chr17	56110100	-
chr17	56654300	+
chr17	56844300	-
chr17	57359900	-
chr17	58156400	-
chr17	58397300	+
chr17	58877700	+
chr17	59173000	-
chr17	60088400	-
chr17	60402400	-
chr17	60412100	+
chr17	60527400	-
chr17	60549800	+
chr17	62867700	+
chr17	62905100	-
chr17	63252000	+
chr17	63419300	+
chr17	63446700	+
chr17	63462200	+
chr17	63707800	-
chr17	63713800	+
chr17	63746000	+
chr17	64834700	-
chr17	64922700	+
chr17	68228000	-
chr17	68600300	-
chr17	69974700	-
chr17	70245500	+
chr17	70542100	-
chr17	70584900	+
chr17	70595500	+
chr17	70617500	+
chr17	70690200	-
chr17	70964400	+
chr17	71292500	+
chr17	71487000	-
chr17	71891200	+
chr17	72150400	+
chr17	72179400	+
chr17	72476600	+
chr17	73117700	+
chr17	73153200	+
chr17	74042600	+
chr17	75625000	-
chr17	76043100	+
chr17	76607200	-
chr17	77848900	+
chr17	78001400	-
chr18	802200	+
chr18	2580400	-
chr18	3052200	-
chr18	3287300	-
chr18	3592800	+
chr18	5300600	-
chr18	5446500	+
chr18	7936800	+
chr18	9073800	-
chr18	9085200	+
chr18	9395100	-
chr18	11898400	-
chr18	11936900	+
chr18	11971200	+
chr18	12765700	-
chr18	12938000	-
chr18	13127700	-
chr18	13166600	+
chr18	13256900	-
chr18	13264300	-
chr18	13269500	-
chr18	13427500	+
chr18	17517100	-
chr18	18767400	+
chr18	19072400	-
chr18	19271900	-
chr18	19496500	-
chr18	20231800	-
chr18	20494600	-
chr18	20998900	-
chr18	21788100	-
chr18	22024100	+
chr18	22060700	+
chr18	27852700	+
chr18	30863900	-
chr18	31101000	+
chr18	31211000	-
chr18	31901400	-
chr18	32663000	-
chr18	40594000	-
chr18	41832500	-
chr18	41938200	+
chr18	42084500	-
chr18	44319800	+
chr18	44615200	-
chr18	46079200	-
chr18	46088000	+
chr18	46155600	-
chr18	46601000	-
chr18	46748400	+
chr18	46932300	+
chr18	50049700	+
chr18	50138300	+
chr18	50646600	+
chr18	50764000	+
chr18	51295400	+
chr18	52469400	+
chr18	53404800	-
chr18	53440200	-
chr18	53448900	-
chr18	53651500	+
chr18	53862300	+
chr18	54433400	+
chr18	55718200	+
chr18	58324200	+
chr18	58663900	-
chr18	59185100	-
chr18	63062100	+
chr18	63334900	+
chr18	64539900	+
chr18	65716700	-
chr18	65753800	+
chr18	66024200	-
chr18	69901500	-
chr18	70317800	-
chr18	70472900	+
chr18	70835500	+
chr18	72292500	-
chr18	73049200	+
chr18	76006500	+
chr19	1441500	+
chr19	2204700	-
chr19	2446300	-
chr19	2928400	-
chr19	2986700	-
chr19	4279700	-
chr19	4818400	-
chr19	5779000	+
chr19	6037800	+
chr19	6061200	-
chr19	6411100	+
chr19	6440900	-
chr19	6468000	-
chr19	7148600	+
chr19	7459500	+
chr19	8239900	+
chr19	8334600	-
chr19	8804200	-
chr19	9112000	+
chr19	9740300	-
chr19	9764100	+
chr19	10673000	+
chr19	11127400	+
chr19	11317900	+
chr19	11710500	-
chr19	11738900	+
chr19	11770000	+
chr19	11786300	-
chr19	11859600	+
chr19	11896400	+
chr19	11937100	-
chr19	12024100	+
chr19	12063700	+
chr19	12128200	-
chr19	12305100	-
chr19	12337400	-
chr19	12372900	-
chr19	12456700	-
chr19	12467600	-
chr19	12749900	+
chr19	12929200	-
chr19	13134700	+
chr19	13144700	-
chr19	13703000	+
chr19	13767000	+
chr19	13878100	+
chr19	14046400	-
chr19	14053400	+
chr19	14108500	+
chr19	15523400	-
chr19	15808900	-
chr19	15973500	+
chr19	16310400	+
chr19	16468200	-
chr19	17097200	+
chr19	17155600	+
chr19	17362900	+
chr19	17391700	+
chr19	17904600	+
chr19	19838200	+
chr19	19872900	+
chr19	20011000	-
chr19	20050500	-
chr19	20209600	+
chr19	20897800	+
chr19	20975000	-
chr19	21303900	-
chr19	21480200	+
chr19	21579300	-
chr19	21742400	-
chr19	21826900	-
chr19	23224300	+
chr19	23248300	-
chr19	23369900	-
chr19	23662100	-
chr19	23738100	-
chr19	23889100	+
chr19	24061600	+
chr19	35014000	+
chr19	35027800	-
chr19	37588700	-
chr19	39003300	-
chr19	39317600	-
chr19	39355100	+
chr19	39452500	+
chr19	39542300	-
chr19	39587000	+
chr19	39916600	+
chr19	40110200	-
chr19	40307000	+
chr19	40431000	+
chr19	40885100	+
chr19	41196700	+
chr19	41237200	-
chr19	41311200	-
chr19	42838200	-
chr19	42961700	+
chr19	43498800	+
chr19	43544500	+
chr19	43578200	-
chr19	43585400	+
chr19	43600200	+
chr19	44061100	-
chr19	44213700	+
chr19	44740800	+
chr19	44861700	+
chr19	45623300	-
chr19	45860900	+
chr19	46424100	+
chr19	46744900	-
chr19	48771500	-
chr19	48995100	-
chr19	49023000	+
chr19	49131100	+
chr19	49180000	+
chr19	49337700	-
chr19	50646000	-
chr19	50779700	-
chr19	50863500	+
chr19	52149400	+
chr19	52230800	+
chr19	52679600	-
chr19	53444200	+
chr19	53558800	+
chr19	53708400	-
chr19	53840400	+
chr19	54128700	-
chr19	55553100	+
chr19	55563800	+
chr19	55627100	-
chr19	56589700	-
chr19	56636400	+
chr19	56898800	+
chr19	57366700	-
chr19	57731300	-
chr19	57796200	+
chr19	57833100	+
chr19	57885900	-
chr19	57930000	-
chr19	58118500	-
chr19	58560800	-
chr19	59291600	-
chr19	59591800	+
chr19	61518100	-
chr19	63022900	+
chr19	63566000	-
chr19	63654300	+
chr2	165600	+
chr2	420400	-
chr2	427700	+
chr2	3676700	+
chr2	6839000	-
chr2	6935100	+
chr2	7488600	-
chr2	8514300	-
chr2	8546300	-
chr2	9061200	-
chr2	10086700	-
chr2	11162000	-
chr2	11523700	-
chr2	11887200	+
chr2	12185200	+
chr2	12329300	-
chr2	12514700	-
chr2	17798700	-
chr2	19932300	+
chr2	20052900	+
chr2	23608900	+
chr2	24016700	+
chr2	24123100	+
chr2	24200000	-
chr2	24869400	+
chr2	25003400	-
chr2	25048100	+
chr2	25238100	-
chr2	25371800	-
chr2	25438700	+
chr2	25870500	+
chr2	26375900	-
chr2	26579700	+
chr2	26862400	+
chr2	27047300	-
chr2	27200200	-
chr2	27566300	-
chr2	27811900	+
chr2	27966900	+
chr2	28643500	-
chr2	30302500	-
chr2	30343400	-
chr2	30498400	-
chr2	31310400	+
chr2	32118200	-
chr2	32706400	+
chr2	32834800	+
chr2	33554900	+
chr2	33677800	-
chr2	36437800	-
chr2	37237600	-
chr2	37425900	-
chr2	37666800	-
chr2	38006100	-
chr2	38746800	+
chr2	38859400	-
chr2	38956600	-
chr2	39205200	-
chr2	39859500	+
chr2	40570200	-
chr2	41922000	-
chr2	42186300	+
chr2	42213500	+
chr2	42575700	-
chr2	42649600	+
chr2	42891500	-
chr2	43239100	+
chr2	43718100	-
chr2	43854500	+
chr2	44168700	-
chr2	45648100	+
chr2	45941200	+
chr2	46580200	+
chr2	46622400	+
chr2	46779700	-
chr2	47352600	+
chr2	47601600	-
chr2	48192300	+
chr2	54804300	+
chr2	54978800	-
chr2	55214200	+
chr2	55313200	+
chr2	55349900	+
chr2	55362700	+
chr2	55500400	+
chr2	55600300	+
chr2	58127600	-
chr2	58322100	-
chr2	61146700	+
chr2	61226100	-
chr2	61775600	-
chr2	61935000	-
chr2	61986000	+
chr2	62390700	-
chr2	62786400	+
chr2	64099800	-
chr2	64285400	-
chr2	64309500	-
chr2	64687500	+
chr2	64811900	-
chr2	64848200	+
chr2	64900700	-
chr2	64999200	-
chr2	65027700	-
chr2	65112700	-
chr2	65512500	-
chr2	68547900	-
chr2	69517800	+
chr2	69822600	+
chr2	69995700	+
chr2	70175700	-
chr2	70205500	-
chr2	70212900	+
chr2	70223100	-
chr2	70271500	-
chr2	70634400	-
chr2	70848400	-
chr2	71029700	-
chr2	71058100	+
chr2	71307600	-
chr2	72905900	-
chr2	73034800	+
chr2	73152400	-
chr2	73909500	+
chr2	74059600	+
chr2	74201100	-
chr2	74286400	+
chr2	74930100	+
chr2	75727300	+
chr2	75791400	-
chr2	84596600	+
chr2	84969900	-
chr2	85007100	-
chr2	85019900	+
chr2	85499100	+
chr2	85518300	+
chr2	85934400	-
chr2	86703700	-
chr2	88097100	+
chr2	95555800	+
chr2	96355000	+
chr2	96365500	-
chr2	96516100	-
chr2	96953800	+
chr2	99124300	-
chr2	99137800	+
chr2	100400400	-
chr2	100564100	-
chr2	100924200	+
chr2	101134700	-
chr2	101345300	-
chr2	101457300	+
chr2	102065200	+
chr2	102087000	+
chr2	102233800	+
chr2	102339100	+
chr2	104362200	-
chr2	105020800	+
chr2	105357200	-
chr2	108517300	+
chr2	108596000	-
chr2	108604000	+
chr2	108701800	+
chr2	108769800	-
chr2	108971700	-
chr2	109015200	-
chr2	109315400	-
chr2	110319700	+
chr2	110327100	-
chr2	111332600	+
chr2	112358000	+
chr2	112372700	-
chr2	112630100	-
chr2	112749800	+
chr2	113101300	-
chr2	113239000	-
chr2	118562700	+
chr2	118659900	+
chr2	118784200	-
chr2	119357200	-
chr2	120168700	+
chr2	120234000	+
chr2	120403900	+
chr2	120487300	-
chr2	120651400	+
chr2	120706200	-
chr2	120787600	+
chr2	122174400	-
chr2	122449700	-
chr2	127250800	+
chr2	127499900	-
chr2	127581200	-
chr2	127889900	+
chr2	130351700	-
chr2	131200800	+
chr2	131272200	-
chr2	132156800	-
chr2	136005200	+
chr2	136459300	+
chr2	136856000	+
chr2	144806300	-
chr2	148982800	+
chr2	149027200	+
chr2	151826500	-
chr2	152202700	+
chr2	152740600	-
chr2	156906800	-
chr2	156965400	-
chr2	158193500	-
chr2	158464700	-
chr2	159022100	+
chr2	159851600	-
chr2	160834900	-
chr2	160976800	-
chr2	161057600	-
chr2	161701100	+
chr2	161808400	+
chr2	162628300	+
chr2	162638700	-
chr2	162883500	-
chr2	162908700	+
chr2	169021500	+
chr2	169055500	-
chr2	170044000	+
chr2	170138500	-
chr2	170259500	-
chr2	171538000	+
chr2	172459100	-
chr2	172572700	+
chr2	172913200	-
chr2	173838100	-
chr2	174585500	+
chr2	174612100	+
chr2	175059700	-
chr2	175066500	+
chr2	176575100	-
chr2	176842400	+
chr2	178125700	+
chr2	178191600	+
chr2	178987200	-
chr2	179604800	+
chr2	179622400	+
chr2	179837300	-
chr2	181553700	+
chr2	182465100	+
chr2	183289200	+
chr2	183611100	+
chr2	183651500	+
chr2	187163000	+
chr2	190153800	-
chr2	190247400	+
chr2	190335900	-
chr2	190916900	+
chr2	190932900	+
chr2	191107300	-
chr2	191201700	-
chr2	191222300	+
chr2	191780000	-
chr2	196641600	-
chr2	196773600	+
chr2	196833400	+
chr2	197212600	+
chr2	197372600	-
chr2	197499600	-
chr2	197829100	-
chr2	197896800	-
chr2	200879800	-
chr2	200946700	+
chr2	201040900	-
chr2	201082700	+
chr2	201098900	+
chr2	201462100	+
chr2	202215700	+
chr2	202354100	+
chr2	202483600	-
chr2	203208300	+
chr2	203484900	-
chr2	203587500	+
chr2	203901400	+
chr2	204108000	-
chr2	204426800	+
chr2	204682700	+
chr2	206656800	+
chr2	207432100	+
chr2	208198200	+
chr2	208598700	-
chr2	208827800	-
chr2	210744000	-
chr2	211049400	-
chr2	213857300	+
chr2	215382700	-
chr2	216654700	+
chr2	216985300	+
chr2	218363000	-
chr2	218608200	-
chr2	218889700	+
chr2	218969700	+
chr2	219283700	+
chr2	219482300	-
chr2	222090900	+
chr2	223362300	+
chr2	224410500	-
chr2	224612000	-
chr2	227364600	-
chr2	228356700	-
chr2	230641100	-
chr2	230798200	+
chr2	231498200	-
chr2	231771600	-
chr2	232056400	-
chr2	232087700	-
chr2	232186900	-
chr2	232936600	+
chr2	233992400	+
chr2	234147400	+
chr2	234427700	-
chr2	234999600	+
chr2	237543500	-
chr2	238805100	-
chr2	242322800	+
chr2	242392400	-
chr20	167000	-
chr20	195700	-
chr20	219400	-
chr20	581800	-
chr20	1585700	+
chr20	2437300	+
chr20	2453500	+
chr20	3102400	-
chr20	3336300	-
chr20	3706600	-
chr20	3749100	-
chr20	3908900	+
chr20	3966200	+
chr20	4101100	-
chr20	4655000	+
chr20	4689400	+
chr20	5006700	+
chr20	5041700	-
chr20	5879200	+
chr20	8061300	+
chr20	8143400	-
chr20	10430200	+
chr20	12938400	-
chr20	13149900	-
chr20	13176800	-
chr20	13567800	-
chr20	16503800	-
chr20	17433300	+
chr20	18425200	+
chr20	18496000	+
chr20	18516600	+
chr20	21054700	-
chr20	21477600	-
chr20	21486200	+
chr20	23995700	-
chr20	24667800	-
chr20	25077800	-
chr20	25239800	-
chr20	25336200	+
chr20	25679700	+
chr20	29624600	-
chr20	29656500	+
chr20	29713000	+
chr20	29790900	-
chr20	29931500	-
chr20	30794800	-
chr20	30813900	+
chr20	31737500	-
chr20	31783600	+
chr20	32354600	-
chr20	33007200	-
chr20	33463400	-
chr20	33653300	-
chr20	34102500	-
chr20	34116200	-
chr20	34667700	+
chr20	34835400	+
chr20	35474700	-
chr20	36736600	-
chr20	37112500	-
chr20	39065400	+
chr20	39379500	-
chr20	39680200	-
chr20	41576600	+
chr20	41652900	-
chr20	41719800	-
chr20	41784500	-
chr20	42280700	-
chr20	42538000	+
chr20	42594300	-
chr20	43410400	-
chr20	43874900	-
chr20	43973700	-
chr20	44180500	-
chr20	45564400	+
chr20	45789400	+
chr20	46810900	-
chr20	47645000	-
chr20	48121000	+
chr20	48388700	+
chr20	48533800	-
chr20	48845000	+
chr20	50241600	-
chr20	50543800	+
chr20	51051500	+
chr20	51673100	-
chr20	51711000	-
chr20	51757500	+
chr20	51879600	-
chr20	51965200	+
chr20	54420500	+
chr20	57131800	-
chr20	57155000	+
chr20	58064700	+
chr20	58146400	+
chr20	60610300	+
chr20	61134700	+
chr20	61639500	+
chr21	14897300	-
chr21	15360400	-
chr21	17807300	+
chr21	18078500	-
chr21	25751000	+
chr21	25856100	+
chr21	25933900	+
chr21	26365400	-
chr21	26464400	-
chr21	29178800	+
chr21	29296500	+
chr21	29313300	-
chr21	29491300	-
chr21	29730900	-
chr21	31853200	-
chr21	32573200	+
chr21	32864100	-
chr21	33181000	-
chr21	33656100	-
chr21	33785900	-
chr21	33882800	-
chr21	34188800	+
chr21	34226100	+
chr21	34496500	-
chr21	34909100	-
chr21	34934200	+
chr21	35637000	+
chr21	36407500	+
chr21	36429500	+
chr21	36592700	-
chr21	37283900	+
chr21	37515100	-
chr21	37858000	+
chr21	39061800	-
chr21	39739100	+
chr21	41407300	-
chr21	41713900	+
chr21	42219200	+
chr21	42356800	-
chr21	42789300	+
chr21	42821300	-
chr21	42977700	-
chr21	43040500	-
chr21	43077700	-
chr21	43125600	+
chr21	43338900	+
chr21	44452000	-
chr22	16032800	-
chr22	16080500	-
chr22	16097700	+
chr22	16863900	-
chr22	17012300	+
chr22	17273800	+
chr22	17659500	-
chr22	19698400	+
chr22	20341600	+
chr22	20419800	-
chr22	21192700	-
chr22	22422900	+
chr22	22529900	+
chr22	22586200	-
chr22	22714100	+
chr22	23532400	-
chr22	23837100	+
chr22	24290500	+
chr22	25384100	-
chr22	26528100	-
chr22	28947000	+
chr22	29332400	+
chr22	29420800	-
chr22	29886200	+
chr22	30403000	-
chr22	30479800	+
chr22	30696400	+
chr22	30929500	-
chr22	31370300	+
chr22	34126000	+
chr22	34353900	-
chr22	34792300	-
chr22	34978900	+
chr22	35255200	-
chr22	35587000	+
chr22	35704100	-
chr22	35924900	-
chr22	36783000	+
chr22	37296400	+
chr22	37740100	+
chr22	39258200	+
chr22	39748100	-
chr22	39923200	+
chr22	40672400	+
chr22	40796600	-
chr22	40805700	-
chr22	41095400	-
chr22	41163300	+
chr22	41170300	+
chr22	41869400	-
chr22	41989900	-
chr22	42207800	-
chr22	42539500	+
chr22	43230200	-
chr22	44810600	+
chr22	45071000	+
chr22	46249800	-
chr22	49348900	-
chr22	49406400	+
chr3	3209100	-
chr3	4483300	+
chr3	4534900	+
chr3	4885200	-
chr3	8980200	-
chr3	9265500	+
chr3	9826700	-
chr3	9932800	-
chr3	10181900	-
chr3	10568700	-
chr3	11736100	-
chr3	12500700	+
chr3	12827000	-
chr3	13468600	-
chr3	13896300	-
chr3	16858700	+
chr3	17759100	-
chr3	17766400	-
chr3	20202600	+
chr3	23219700	-
chr3	25806400	+
chr3	27649800	-
chr3	27898500	-
chr3	30451900	+
chr3	31478900	+
chr3	32255500	+
chr3	32444400	+
chr3	32798300	-
chr3	33675600	+
chr3	36893400	-
chr3	37009500	-
chr3	37876900	+
chr3	38047200	-
chr3	38056200	-
chr3	38363100	+
chr3	39197000	-
chr3	40326200	+
chr3	40493600	+
chr3	40522700	-
chr3	40541500	-
chr3	41978300	-
chr3	43179700	-
chr3	43638400	-
chr3	43707400	+
chr3	44354900	+
chr3	44778300	-
chr3	45405300	-
chr3	45858600	-
chr3	46229600	+
chr3	46424100	-
chr3	46506100	-
chr3	46575100	-
chr3	46646000	+
chr3	46829500	-
chr3	46862700	-
chr3	48204700	-
chr3	48257200	+
chr3	49106200	-
chr3	49288900	+
chr3	49441900	-
chr3	49552200	+
chr3	49868900	+
chr3	49942400	-
chr3	51037300	+
chr3	52163400	+
chr3	52780000	-
chr3	53053400	+
chr3	53139400	-
chr3	56924700	+
chr3	57960100	-
chr3	58139300	+
chr3	58198500	+
chr3	59010400	+
chr3	59405200	-
chr3	61211700	+
chr3	62406700	-
chr3	63992200	-
chr3	67105000	+
chr3	67131600	+
chr3	69145200	+
chr3	71437500	-
chr3	71588300	-
chr3	71713900	+
chr3	71916300	+
chr3	72870800	+
chr3	81893500	-
chr3	87358900	+
chr3	95181600	+
chr3	95264200	+
chr3	98965900	+
chr3	99073600	+
chr3	100103100	-
chr3	101411300	+
chr3	101536500	-
chr3	102926400	+
chr3	103029900	-
chr3	107378500	-
chr3	107408300	-
chr3	108632900	-
chr3	108800400	+
chr3	109129400	-
chr3	109326700	+
chr3	109423600	+
chr3	109791400	-
chr3	109805200	+
chr3	112272400	+
chr3	112525800	+
chr3	113335200	-
chr3	113505100	+
chr3	113535000	+
chr3	113559700	+
chr3	113700300	-
chr3	114176200	-
chr3	114716600	-
chr3	115149500	+
chr3	115354700	-
chr3	115415700	+
chr3	116301600	+
chr3	116714800	-
chr3	116859900	+
chr3	116985900	+
chr3	120495900	-
chr3	120699800	+
chr3	120781200	+
chr3	120904900	-
chr3	121011200	+
chr3	121618500	+
chr3	121760900	-
chr3	122634700	-
chr3	122747500	-
chr3	123778800	+
chr3	124269000	+
chr3	124786800	+
chr3	125162800	-
chr3	125822900	-
chr3	126323200	-
chr3	126512900	-
chr3	126796900	-
chr3	127192200	-
chr3	127905700	+
chr3	128749200	-
chr3	128799800	+
chr3	129354400	+
chr3	129882900	-
chr3	129989900	-
chr3	130047700	-
chr3	130125200	-
chr3	130195700	+
chr3	130228800	-
chr3	130829000	+
chr3	132095300	+
chr3	132704400	-
chr3	133861700	-
chr3	134707600	+
chr3	135128800	+
chr3	135565800	+
chr3	137452300	-
chr3	137954200	-
chr3	139531500	-
chr3	139796000	-
chr3	139810600	-
chr3	140117200	-
chr3	140530200	+
chr3	140879400	-
chr3	142143600	+
chr3	142279000	-
chr3	142469000	-
chr3	142513500	-
chr3	142604000	+
chr3	142888900	-
chr3	143077700	+
chr3	143350800	-
chr3	143427000	-
chr3	143797700	+
chr3	143925900	+
chr3	144953100	+
chr3	145049700	+
chr3	147670400	-
chr3	150286900	-
chr3	150425200	+
chr3	150953300	-
chr3	151171500	-
chr3	151585900	-
chr3	151904000	+
chr3	153404500	+
chr3	157006300	+
chr3	157071300	+
chr3	157684400	-
chr3	158017500	+
chr3	158027100	-
chr3	158289700	+
chr3	159311400	-
chr3	159873100	-
chr3	161189600	-
chr3	161650000	-
chr3	162305800	-
chr3	170964900	+
chr3	171013100	+
chr3	171238800	+
chr3	171423100	+
chr3	173677200	+
chr3	173723600	+
chr3	173911300	-
chr3	173951000	+
chr3	178561000	-
chr3	178627300	+
chr3	180238500	-
chr3	180461800	-
chr3	180854000	-
chr3	184362900	-
chr3	184424600	+
chr3	184647800	+
chr3	186483700	-
chr3	187973200	+
chr3	188007100	-
chr3	188226100	+
chr3	188339700	+
chr3	189003000	+
chr3	189120000	-
chr3	189136200	+
chr3	189559200	+
chr3	191714400	+
chr3	195338400	-
chr3	195456500	-
chr3	195507300	-
chr3	195599900	-
chr3	195758300	+
chr3	195785900	+
chr3	195835300	-
chr3	195971400	-
chr3	196331200	+
chr3	196350000	+
chr3	197391000	+
chr3	197430200	+
chr3	197835100	+
chr3	197843900	-
chr3	197923600	-
chr3	198180300	-
chr3	198667700	+
chr3	199161200	+
chr4	1833700	+
chr4	2012700	+
chr4	3174300	-
chr4	4008300	-
chr4	6322500	-
chr4	6742500	-
chr4	6804000	+
chr4	7155100	+
chr4	7811900	+
chr4	7929600	-
chr4	7992100	-
chr4	8211900	-
chr4	9215500	-
chr4	9302500	-
chr4	9629600	+
chr4	14844300	+
chr4	15012300	-
chr4	15313800	+
chr4	17122800	-
chr4	23501300	-
chr4	24771600	+
chr4	24923700	-
chr4	24941900	+
chr4	25524700	+
chr4	26194600	+
chr4	26494600	+
chr4	37997600	-
chr4	38483100	-
chr4	38708600	+
chr4	38860400	+
chr4	39031400	+
chr4	39137100	-
chr4	39316600	-
chr4	40013500	+
chr4	40248400	-
chr4	44375100	+
chr4	44423200	-
chr4	47160500	-
chr4	47181800	+
chr4	47533900	-
chr4	47611100	-
chr4	47713300	+
chr4	47966800	-
chr4	52599000	+
chr4	53220600	-
chr4	53282900	+
chr4	53926900	-
chr4	55907100	+
chr4	56107500	+
chr4	56948800	-
chr4	57091300	+
chr4	57214500	+
chr4	57318900	-
chr4	57382700	-
chr4	68093900	-
chr4	70660300	-
chr4	71819100	+
chr4	71987000	+
chr4	74154000	-
chr4	75242700	+
chr4	76774600	+
chr4	77446500	-
chr4	78297700	-
chr4	78334200	-
chr4	78994100	-
chr4	79002600	+
chr4	79916400	+
chr4	80079700	-
chr4	81212900	-
chr4	81313300	-
chr4	81603500	-
chr4	83425100	-
chr4	84041000	-
chr4	84153300	-
chr4	84360200	-
chr4	84424800	-
chr4	84475300	-
chr4	84595600	-
chr4	84676200	-
chr4	87500800	-
chr4	88360500	-
chr4	88531400	-
chr4	88563100	-
chr4	89299100	-
chr4	89963700	-
chr4	90439200	-
chr4	91035400	-
chr4	95348400	+
chr4	95592200	+
chr4	96136800	-
chr4	99283200	+
chr4	99344500	-
chr4	99779600	+
chr4	100228800	+
chr4	100956600	+
chr4	103770400	+
chr4	104009500	+
chr4	104160100	+
chr4	104216900	-
chr4	104240400	-
chr4	104339000	-
chr4	106287300	+
chr4	109072200	+
chr4	109130300	+
chr4	109312900	+
chr4	113372300	+
chr4	119976500	-
chr4	120353000	+
chr4	120768500	+
chr4	121207400	+
chr4	122298200	-
chr4	122322500	-
chr4	122341100	-
chr4	122368300	-
chr4	122383900	+
chr4	122837800	-
chr4	122941400	+
chr4	122964300	-
chr4	123011100	-
chr4	123599700	-
chr4	123873200	+
chr4	129021500	+
chr4	139382900	-
chr4	140156300	-
chr4	140406900	+
chr4	140442200	+
chr4	140807000	-
chr4	141190300	-
chr4	141212000	-
chr4	141236600	-
chr4	141664500	+
chr4	143518000	+
chr4	143697300	+
chr4	143987400	-
chr4	144699900	+
chr4	146759700	+
chr4	147383500	+
chr4	148086900	-
chr4	148932900	+
chr4	149357400	+
chr4	152901600	-
chr4	153387100	+
chr4	153820300	-
chr4	154294000	+
chr4	154363200	+
chr4	154389900	-
chr4	154485500	+
chr4	154573900	+
chr4	154825000	+
chr4	156517700	-
chr4	157094000	+
chr4	159812300	+
chr4	159951700	+
chr4	164635000	+
chr4	164698700	+
chr4	166118400	-
chr4	166253400	-
chr4	169476200	-
chr4	170167900	-
chr4	170770300	-
chr4	171184500	+
chr4	174326400	+
chr4	175441400	+
chr4	178467700	+
chr4	178600500	-
chr4	184075700	+
chr4	184257800	-
chr4	184556900	-
chr4	184817400	+
chr4	184880900	+
chr4	185426300	-
chr4	185502500	-
chr4	185695800	-
chr4	185892200	-
chr4	186300900	+
chr4	186629600	-
chr4	187227000	-
chr4	187319300	+
chr4	187349800	-
chr5	446300	-
chr5	596700	+
chr5	1307900	+
chr5	1589100	+
chr5	1778400	-
chr5	1846100	+
chr5	7905200	-
chr5	8510800	+
chr5	10494800	-
chr5	10680400	+
chr5	14196100	+
chr5	14468000	-
chr5	18781600	-
chr5	31675300	-
chr5	32053600	+
chr5	32210100	-
chr5	32567800	+
chr5	34043800	-
chr5	34874900	+
chr5	35653400	+
chr5	35816900	+
chr5	35961000	-
chr5	37285200	-
chr5	39245300	-
chr5	39252900	+
chr5	41961000	+
chr5	43141100	+
chr5	43433100	-
chr5	43519500	+
chr5	43550900	-
chr5	49772800	-
chr5	52119100	+
chr5	52892000	+
chr5	53978800	-
chr5	54866300	-
chr5	55043800	-
chr5	55492200	-
chr5	55812700	-
chr5	56241300	+
chr5	57791800	-
chr5	58091300	+
chr5	58459200	-
chr5	60031900	-
chr5	60494200	-
chr5	60662200	+
chr5	64367300	+
chr5	64434900	+
chr5	64894600	+
chr5	65053800	-
chr5	65198400	+
chr5	65475900	+
chr5	66498200	-
chr5	66869600	-
chr5	67765600	+
chr5	68043400	-
chr5	68498600	+
chr5	68521200	-
chr5	68566400	+
chr5	68663900	+
chr5	68747200	-
chr5	72148400	-
chr5	73908200	+
chr5	74197800	-
chr5	74266600	+
chr5	74568200	+
chr5	75001200	-
chr5	76047400	+
chr5	76150600	+
chr5	76823900	-
chr5	77626600	-
chr5	78316600	-
chr5	78845800	-
chr5	79739500	+
chr5	79819400	+
chr5	80087300	-
chr5	80338600	+
chr5	81110900	-
chr5	81183600	-
chr5	81303300	+
chr5	87834200	-
chr5	89741400	-
chr5	90611900	+
chr5	92982800	-
chr5	95008400	+
chr5	95122500	-
chr5	95196700	+
chr5	96178200	+
chr5	96238300	+
chr5	98137500	+
chr5	99899400	-
chr5	101659600	+
chr5	102119000	+
chr5	102622700	+
chr5	102925900	+
chr5	108091600	+
chr5	109285500	+
chr5	111121200	+
chr5	114660100	-
chr5	114966000	-
chr5	115448600	-
chr5	118351800	+
chr5	118434500	+
chr5	118816000	+
chr5	122208700	-
chr5	122786800	-
chr5	125787600	-
chr5	125813300	+
chr5	125958500	+
chr5	126215700	+
chr5	126232200	+
chr5	126336300	-
chr5	126437100	-
chr5	126593400	-
chr5	130573100	+
chr5	131338200	+
chr5	131860400	-
chr5	132194000	-
chr5	133293700	+
chr5	133435500	-
chr5	133829700	-
chr5	134268600	+
chr5	137252600	+
chr5	137577000	-
chr5	137695600	-
chr5	138562100	-
chr5	138748800	-
chr5	138802900	-
chr5	138877600	+
chr5	138903000	+
chr5	139030300	-
chr5	139070900	+
chr5	139107300	-
chr5	139467600	+
chr5	139535100	-
chr5	139662600	+
chr5	139717000	+
chr5	139992600	-
chr5	140070600	+
chr5	141042700	-
chr5	141052400	+
chr5	141209300	+
chr5	141328700	+
chr5	141375500	-
chr5	141384300	+
chr5	141448700	-
chr5	142072700	+
chr5	142144300	-
chr5	142794300	-
chr5	143188000	+
chr5	147142000	-
chr5	148169500	-
chr5	148941300	-
chr5	149090800	+
chr5	150016400	+
chr5	150138500	+
chr5	150612600	+
chr5	150807500	+
chr5	156409300	-
chr5	156469200	-
chr5	156900300	+
chr5	157090800	+
chr5	157103700	+
chr5	158235500	-
chr5	158459900	-
chr5	158622700	+
chr5	159478800	-
chr5	159759300	+
chr5	159782200	+
chr5	159798300	+
chr5	159827100	+
chr5	162863300	+
chr5	167628400	+
chr5	167768700	+
chr5	168043800	-
chr5	168943300	+
chr5	169752600	+
chr5	171643500	-
chr5	172056600	+
chr5	172318800	+
chr5	172395700	-
chr5	172504100	+
chr5	173277500	+
chr5	173333500	-
chr5	176382100	+
chr5	177490700	+
chr5	178090600	-
chr5	180009500	-
chr6	154600	+
chr6	169800	+
chr6	444600	-
chr6	656000	+
chr6	1500400	-
chr6	2168000	-
chr6	2821100	-
chr6	3102900	-
chr6	3301600	-
chr6	3688100	-
chr6	4080800	-
chr6	4949500	-
chr6	5120000	-
chr6	5206100	+
chr6	5796800	-
chr6	6559700	-
chr6	6997400	+
chr6	7032300	+
chr6	7052600	+
chr6	7855700	-
chr6	10637100	+
chr6	10831100	+
chr6	11333300	-
chr6	11646000	-
chr6	11836700	+
chr6	11894800	-
chr6	13436900	-
chr6	13463600	+
chr6	13536600	-
chr6	13563200	-
chr6	13595500	-
chr6	13982400	-
chr6	14319200	+
chr6	14892800	+
chr6	14966000	-
chr6	16346600	+
chr6	16521700	+
chr6	16529900	-
chr6	16546100	-
chr6	16808800	-
chr6	16820500	+
chr6	16945100	+
chr6	17124100	-
chr6	18263600	+
chr6	18495800	-
chr6	20320800	-
chr6	20427900	+
chr6	20443900	+
chr6	20474900	+
chr6	20509000	+
chr6	20517700	+
chr6	20642900	+
chr6	24465700	+
chr6	24603200	-
chr6	24753900	+
chr6	25149800	+
chr6	25387400	+
chr6	25478000	+
chr6	25512600	+
chr6	26100900	-
chr6	26129400	+
chr6	26141500	-
chr6	26163400	+
chr6	26195500	+
chr6	26280500	-
chr6	26296500	+
chr6	26313300	+
chr6	26324900	-
chr6	26332400	+
chr6	26342600	-
chr6	26349300	-
chr6	26393700	-
chr6	26421800	-
chr6	26491400	+
chr6	26529300	+
chr6	26629800	-
chr6	26662300	-
chr6	26768100	-
chr6	27208600	+
chr6	27215100	+
chr6	27233800	-
chr6	27264400	-
chr6	27450400	+
chr6	27554800	-
chr6	27578600	+
chr6	27595100	-
chr6	27763700	-
chr6	27769800	+
chr6	27833200	-
chr6	27867900	+
chr6	27883600	-
chr6	27907000	-
chr6	27942900	-
chr6	27948400	+
chr6	27971000	-
chr6	28156500	+
chr6	28180900	-
chr6	28200100	+
chr6	28237900	-
chr6	28342700	+
chr6	28411700	+
chr6	28425600	+
chr6	28475600	-
chr6	28914100	+
chr6	28939700	+
chr6	28972000	+
chr6	29016800	+
chr6	29057600	-
chr6	29093000	-
chr6	29868600	-
chr6	29963700	+
chr6	30002300	+
chr6	30289400	-
chr6	30335400	+
chr6	30617600	+
chr6	30954000	-
chr6	31031200	+
chr6	31066700	-
chr6	31170200	+
chr6	31234300	+
chr6	31384500	-
chr6	31570300	+
chr6	31696400	+
chr6	31756900	-
chr6	31871000	+
chr6	31890900	-
chr6	31903100	-
chr6	32271800	-
chr6	32664800	+
chr6	32741700	-
chr6	33028600	+
chr6	33094500	+
chr6	33151700	+
chr6	33467500	+
chr6	33668700	+
chr6	34271900	+
chr6	34300100	-
chr6	34602700	+
chr6	34732700	+
chr6	34867900	-
chr6	36463100	-
chr6	36499100	+
chr6	36950700	-
chr6	36988300	+
chr6	37082500	-
chr6	37178800	-
chr6	37298400	-
chr6	37430000	+
chr6	37578600	+
chr6	37781700	-
chr6	39190800	-
chr6	39304800	-
chr6	41104000	-
chr6	41276400	+
chr6	41394300	+
chr6	41607600	+
chr6	41758800	+
chr6	41970900	+
chr6	42179800	+
chr6	42293600	-
chr6	42623800	-
chr6	42802300	+
chr6	42955300	+
chr6	43005300	+
chr6	43129300	-
chr6	43304600	+
chr6	43323300	-
chr6	43651600	+
chr6	43720700	+
chr6	44313700	-
chr6	45007800	+
chr6	45453500	+
chr6	45906900	-
chr6	46091200	-
chr6	46205800	-
chr6	47490300	+
chr6	47553500	+
chr6	49538700	+
chr6	52362600	+
chr6	52392700	+
chr6	52637100	+
chr6	53332100	-
chr6	53521000	-
chr6	53624600	+
chr6	56515800	-
chr6	57145400	+
chr6	57290000	+
chr6	64366500	-
chr6	70582700	-
chr6	71179900	+
chr6	71333600	-
chr6	74029500	+
chr6	74076600	-
chr6	74155100	+
chr6	74218400	-
chr6	74346700	+
chr6	74420600	-
chr6	74462300	+
chr6	79633900	+
chr6	80000400	-
chr6	80770800	-
chr6	80873700	-
chr6	83831900	+
chr6	84994100	-
chr6	88089200	-
chr6	88174600	+
chr6	88239400	+
chr6	88779400	-
chr6	89912600	+
chr6	90123500	+
chr6	90178600	+
chr6	90199700	+
chr6	90405200	-
chr6	90985600	-
chr6	91192300	-
chr6	96132000	+
chr6	97076300	+
chr6	97479100	+
chr6	97837800	-
chr6	99948800	-
chr6	100070200	-
chr6	100075500	+
chr6	101566600	-
chr6	105413800	+
chr6	106546700	+
chr6	106641400	+
chr6	106670100	-
chr6	108085400	+
chr6	108251800	-
chr6	108276700	-
chr6	108688900	-
chr6	108722800	+
chr6	109276100	+
chr6	109302700	-
chr6	109437300	-
chr6	109944000	-
chr6	110118900	+
chr6	110468200	-
chr6	111386400	+
chr6	111515700	+
chr6	112470800	+
chr6	112515300	-
chr6	116798900	+
chr6	117043900	+
chr6	117096600	-
chr6	117976300	-
chr6	119237800	+
chr6	119297500	+
chr6	125664400	-
chr6	126144000	+
chr6	126349200	+
chr6	126702600	+
chr6	127846900	+
chr6	127878700	-
chr6	128263400	+
chr6	128280400	-
chr6	128314300	-
chr6	128335300	+
chr6	128622800	-
chr6	129853900	-
chr6	130035500	-
chr6	130073000	-
chr6	130112200	-
chr6	130223500	+
chr6	130585700	+
chr6	130728800	-
chr6	131425600	-
chr6	131498500	+
chr6	131563200	-
chr6	131806300	-
chr6	133126000	-
chr6	133161500	-
chr6	134541600	-
chr6	134714500	+
chr6	134799800	-
chr6	135449700	+
chr6	135492600	-
chr6	135572900	+
chr6	135860800	-
chr6	136243000	+
chr6	137777700	+
chr6	138470100	+
chr6	139654300	+
chr6	141846000	+
chr6	141924800	+
chr6	143208700	-
chr6	143220700	-
chr6	143270500	-
chr6	143423300	+
chr6	143813600	-
chr6	143917400	+
chr6	144417800	+
chr6	144427000	-
chr6	144579000	-
chr6	144945800	+
chr6	146098000	+
chr6	146327100	-
chr6	149124300	-
chr6	149193000	+
chr6	149596600	+
chr6	150226900	-
chr6	150305300	-
chr6	150431600	+
chr6	150996400	-
chr6	151350700	-
chr6	151752100	+
chr6	152053900	-
chr6	152548600	-
chr6	154519700	-
chr6	155506000	+
chr6	155534300	+
chr6	155677400	-
chr6	155780900	+
chr6	156336600	+
chr6	156632600	-
chr6	156759100	+
chr6	157049900	+
chr6	157082600	+
chr6	157664600	-
chr6	158105100	+
chr6	158508900	+
chr6	159210500	+
chr6	159280300	-
chr6	159340900	-
chr6	159448300	+
chr6	160161000	+
chr6	160861500	-
chr6	163068400	+
chr6	163746700	+
chr6	163757500	-
chr6	166874300	+
chr6	166961100	+
chr6	167380700	+
chr6	167427900	-
chr6	167684600	-
chr6	169026400	-
chr6	169796500	+
chr6	169932200	+
chr7	244500	+
chr7	1175700	+
chr7	2704900	+
chr7	2901100	-
chr7	3069100	+
chr7	3101500	-
chr7	4619700	-
chr7	4648200	-
chr7	5052000	+
chr7	5569000	-
chr7	5576200	-
chr7	6065200	-
chr7	6087700	-
chr7	6277900	+
chr7	6510200	+
chr7	6583200	+
chr7	7431100	-
chr7	7573100	+
chr7	7646700	+
chr7	7768200	+
chr7	7950600	+
chr7	8182200	+
chr7	10980000	+
chr7	12263900	-
chr7	16427400	-
chr7	17240000	+
chr7	17305500	+
chr7	21904300	+
chr7	21951700	-
chr7	22089100	+
chr7	23112200	+
chr7	23545700	-
chr7	23603600	+
chr7	23686500	+
chr7	24579500	+
chr7	25186200	+
chr7	25901000	-
chr7	26298400	+
chr7	26671600	-
chr7	27669000	-
chr7	27979000	-
chr7	28336800	-
chr7	28932700	+
chr7	30328000	+
chr7	30733300	-
chr7	30744600	+
chr7	30777900	+
chr7	32948900	-
chr7	33135600	+
chr7	35043500	-
chr7	36373300	+
chr7	36396100	+
chr7	37414600	+
chr7	38184200	+
chr7	38239200	-
chr7	38469500	+
chr7	39739600	-
chr7	42894300	+
chr7	43765000	-
chr7	47998200	-
chr7	50079200	+
chr7	50103500	-
chr7	50236800	+
chr7	50451300	+
chr7	50485600	-
chr7	50502700	+
chr7	55401100	+
chr7	56069700	-
chr7	63404800	+
chr7	63661100	-
chr7	63667900	-
chr7	63763900	+
chr7	64001000	+
chr7	64350200	-
chr7	64476300	+
chr7	65178200	+
chr7	65307900	+
chr7	65515700	+
chr7	65595800	+
chr7	65947200	+
chr7	66405100	-
chr7	69785000	+
chr7	69933000	-
chr7	72618300	+
chr7	72894400	+
chr7	73146700	-
chr7	73505900	-
chr7	74127600	-
chr7	75206300	-
chr7	75860100	+
chr7	76667100	-
chr7	76818400	+
chr7	76883500	-
chr7	77004400	+
chr7	86526600	-
chr7	86812600	+
chr7	87095800	-
chr7	87401600	+
chr7	88471000	-
chr7	89870700	+
chr7	91348000	-
chr7	91646700	-
chr7	91915200	+
chr7	92185800	-
chr7	92615200	-
chr7	93471500	-
chr7	94124700	-
chr7	94902000	+
chr7	95788900	+
chr7	96584800	+
chr7	99016100	-
chr7	99365300	-
chr7	99433000	+
chr7	99485200	+
chr7	99584400	+
chr7	99602800	+
chr7	99980800	+
chr7	100062100	+
chr7	100069100	-
chr7	100272400	+
chr7	100447500	-
chr7	100752100	-
chr7	101244700	+
chr7	101737600	-
chr7	101853800	-
chr7	102577200	-
chr7	102707900	+
chr7	104372100	+
chr7	104411500	+
chr7	104816200	-
chr7	105498900	+
chr7	105712700	-
chr7	106195700	+
chr7	106554200	+
chr7	107008000	+
chr7	110989500	-
chr7	112546100	-
chr7	113475400	+
chr7	115638000	+
chr7	116380900	+
chr7	120378000	+
chr7	120823400	-
chr7	122961700	+
chr7	124357300	-
chr7	128218900	-
chr7	128365300	+
chr7	128597000	-
chr7	128795700	-
chr7	128861300	+
chr7	128929700	-
chr7	129497400	+
chr7	129569000	-
chr7	129632500	-
chr7	129868100	+
chr7	129913500	+
chr7	130003900	-
chr7	132001500	+
chr7	132588500	+
chr7	133463000	-
chr7	133652100	-
chr7	133767600	+
chr7	133981900	+
chr7	134004900	+
chr7	134200900	-
chr7	134321800	+
chr7	134546700	-
chr7	134845200	-
chr7	137271000	-
chr7	137337400	-
chr7	138454900	-
chr7	138469300	-
chr7	138566800	-
chr7	138676500	+
chr7	138970800	+
chr7	139175800	+
chr7	139524100	-
chr7	139556000	+
chr7	139576800	+
chr7	139722200	+
chr7	139750600	-
chr7	139825500	-
chr7	140003200	-
chr7	141721200	+
chr7	141831500	+
chr7	142409900	+
chr7	142687000	+
chr7	144163400	-
chr7	148242600	-
chr7	148269400	-
chr7	148291500	-
chr7	148316400	-
chr7	148393800	-
chr7	148419000	-
chr7	148454400	+
chr7	148750700	-
chr7	149651200	+
chr7	149835700	+
chr7	151097800	-
chr7	151263800	+
chr7	151792100	-
chr7	152003300	+
chr7	152088100	+
chr7	154351000	+
chr7	156125900	+
chr7	157054600	-
chr7	157337600	-
chr7	158342100	+
chr7	158443800	-
chr7	158683500	-
chr8	1224300	+
chr8	1681700	-
chr8	1724000	-
chr8	1753100	-
chr8	1908100	+
chr8	6251500	+
chr8	8242100	-
chr8	8443100	+
chr8	8764300	+
chr8	9030700	+
chr8	9949400	+
chr8	10229200	-
chr8	10405800	-
chr8	10910900	+
chr8	11362000	-
chr8	11393600	-
chr8	11631800	-
chr8	13035200	-
chr8	17058400	+
chr8	17599300	-
chr8	18111500	+
chr8	19659300	-
chr8	21961900	-
chr8	21974100	-
chr8	22280800	+
chr8	22618400	+
chr8	24855700	-
chr8	26354200	+
chr8	26362500	+
chr8	27461400	-
chr8	27505500	+
chr8	27751300	-
chr8	28315200	-
chr8	28325400	+
chr8	28407800	-
chr8	28536000	-
chr8	28615000	+
chr8	29280700	-
chr8	29413100	-
chr8	29442900	+
chr8	30721400	-
chr8	32846300	-
chr8	33449800	-
chr8	37557700	+
chr8	37671100	+
chr8	37713700	+
chr8	38007100	+
chr8	38067700	-
chr8	38363300	-
chr8	38443300	+
chr8	41506400	-
chr8	41769500	-
chr8	41805100	-
chr8	42117100	-
chr8	42156900	+
chr8	48336100	+
chr8	49035900	+
chr8	49394700	-
chr8	49589700	-
chr8	53368800	-
chr8	54732200	+
chr8	55210600	-
chr8	56919900	-
chr8	57081200	+
chr8	57149400	-
chr8	59734500	-
chr8	59876900	-
chr8	60066400	+
chr8	61940600	+
chr8	61984900	+
chr8	62002000	-
chr8	62789700	-
chr8	64466400	+
chr8	66719200	+
chr8	67188400	-
chr8	67610900	-
chr8	67754700	+
chr8	67787800	+
chr8	67850000	+
chr8	67945300	+
chr8	71216500	-
chr8	71727300	-
chr8	71743900	-
chr8	75066600	+
chr8	75425600	-
chr8	79591100	+
chr8	81178300	-
chr8	81196900	+
chr8	81246300	-
chr8	81993800	-
chr8	82355400	+
chr8	82761300	-
chr8	82916700	+
chr8	86206800	-
chr8	86276900	+
chr8	86345200	-
chr8	87589900	-
chr8	91065800	-
chr8	91709300	-
chr8	92066500	+
chr8	92122300	-
chr8	94836400	-
chr8	95072600	+
chr8	95201500	-
chr8	95556800	-
chr8	95801100	+
chr8	95904900	+
chr8	96042400	-
chr8	96181600	+
chr8	96350600	+
chr8	97726500	+
chr8	99198500	+
chr8	99446700	-
chr8	99907000	+
chr8	100784700	+
chr8	101231900	+
chr8	101576900	-
chr8	101890800	+
chr8	101989600	+
chr8	102237800	-
chr8	103610600	-
chr8	109549700	+
chr8	110621200	+
chr8	116730100	-
chr8	117554000	+
chr8	119171700	-
chr8	120720900	-
chr8	120914400	-
chr8	120937500	-
chr8	120955700	-
chr8	121813500	+
chr8	124001200	-
chr8	124242600	-
chr8	124477500	-
chr8	124498300	+
chr8	125506900	+
chr8	125645600	-
chr8	125769300	-
chr8	126080000	+
chr8	126730100	-
chr8	129637200	-
chr8	131127500	+
chr8	133757100	-
chr8	133841700	+
chr8	135401900	-
chr8	135766500	+
chr8	135913700	-
chr8	141536800	+
chr8	141543800	-
chr8	142285300	+
chr8	142324700	-
chr8	143531200	-
chr8	144343900	-
chr8	144894500	-
chr9	204900	+
chr9	263900	-
chr9	459800	+
chr9	494800	-
chr9	639900	+
chr9	5440200	+
chr9	5500200	+
chr9	6005800	-
chr9	6671200	+
chr9	6693800	+
chr9	6706400	-
chr9	14683100	-
chr9	14983400	+
chr9	15286600	-
chr9	15543100	-
chr9	19221200	+
chr9	19370200	-
chr9	19432400	-
chr9	19455300	-
chr9	20033600	-
chr9	20232400	+
chr9	21021300	-
chr9	21086900	-
chr9	21347500	+
chr9	21549800	-
chr9	21984800	+
chr9	26882700	-
chr9	26946200	+
chr9	27519500	-
chr9	32374400	+
chr9	34513000	-
chr9	34691300	+
chr9	35152000	+
chr9	35480200	-
chr9	35610000	-
chr9	35818800	+
chr9	36460500	+
chr9	36563200	-
chr9	37069700	+
chr9	37097300	+
chr9	37110400	+
chr9	37566400	-
chr9	38059800	-
chr9	38217900	-
chr9	38382600	+
chr9	38611400	-
chr9	70926500	+
chr9	70973200	+
chr9	72224200	+
chr9	72470400	-
chr9	74332100	-
chr9	76757200	-
chr9	78264200	+
chr9	78456200	-
chr9	79259400	-
chr9	79834600	+
chr9	79937100	+
chr9	80101900	+
chr9	81375400	+
chr9	85428000	+
chr9	85726000	-
chr9	85761500	-
chr9	88753300	-
chr9	89779600	-
chr9	91802300	-
chr9	92995600	+
chr9	93010100	+
chr9	94209100	-
chr9	94680300	-
chr9	95832700	-
chr9	96441500	-
chr9	96458500	-
chr9	96528500	+
chr9	97119700	-
chr9	97318300	+
chr9	97823700	-
chr9	97952100	+
chr9	98185700	-
chr9	98368900	-
chr9	98457100	+
chr9	99040300	+
chr9	99724800	-
chr9	99920700	+
chr9	100609900	+
chr9	101522300	+
chr9	102214100	-
chr9	102229300	+
chr9	102400700	+
chr9	105896300	-
chr9	106549700	+
chr9	106566300	-
chr9	106729300	+
chr9	106770200	+
chr9	106793600	+
chr9	107046800	+
chr9	107187400	+
chr9	107250400	-
chr9	107360000	+
chr9	110645300	-
chr9	111950600	+
chr9	113327500	+
chr9	113740800	-
chr9	114182100	-
chr9	114289000	-
chr9	114953500	-
chr9	115141900	+
chr9	115688500	+
chr9	116492900	+
chr9	119585900	-
chr9	122382100	-
chr9	122876900	+
chr9	122924400	+
chr9	124618600	-
chr9	124637100	+
chr9	124707200	-
chr9	125125100	-
chr9	125141900	-
chr9	126060200	+
chr9	126460900	+
chr9	126655500	-
chr9	126743200	+
chr9	126945300	-
chr9	127002600	+
chr9	127550900	-
chr9	129047100	+
chr9	129414500	-
chr9	130005000	+
chr9	130052600	+
chr9	130164600	-
chr9	130504600	+
chr9	130704500	+
chr9	130977100	+
chr9	131185400	+
chr9	131540600	-
chr9	131931300	-
chr9	132547100	+
chr9	133104200	-
chr9	133238700	-
chr9	134027200	+
chr9	134634700	+
chr9	134744000	-
chr9	135558300	-
chr9	135848400	-
chr9	136487900	+
chr9	138298500	+
chr9	138964400	-
chrX	7076200	-
chrX	7855600	-
chrX	7965500	+
chrX	10026800	+
chrX	10039300	-
chrX	11686700	+
chrX	12719500	+
chrX	12878800	-
chrX	13957700	-
chrX	14801500	+
chrX	15086600	-
chrX	15263700	-
chrX	15718300	+
chrX	16647600	+
chrX	16714500	+
chrX	16810500	+
chrX	17665600	+
chrX	18353400	-
chrX	18814800	-
chrX	18912400	-
chrX	19301200	-
chrX	19919000	-
chrX	21302500	+
chrX	21767800	+
chrX	21901800	+
chrX	23595800	+
chrX	23670900	-
chrX	23982700	+
chrX	24393400	+
chrX	24574900	+
chrX	24621700	+
chrX	30581300	+
chrX	30817200	-
chrX	37591700	-
chrX	38071900	-
chrX	38305800	-
chrX	38919100	+
chrX	39518700	-
chrX	39681700	-
chrX	39757800	-
chrX	39919200	-
chrX	40696700	+
chrX	40754900	-
chrX	41020500	+
chrX	41075400	+
chrX	43399400	+
chrX	44088600	-
chrX	44287300	-
chrX	44931000	+
chrX	45514900	-
chrX	45551000	+
chrX	46191600	+
chrX	46332500	-
chrX	46503200	-
chrX	46581300	+
chrX	47227300	-
chrX	47747800	-
chrX	47816000	-
chrX	48419600	+
chrX	48480800	+
chrX	48545000	+
chrX	48844800	+
chrX	49053000	-
chrX	51155400	+
chrX	51562700	+
chrX	51653800	-
chrX	53041700	-
chrX	53118400	-
chrX	53140000	+
chrX	53333900	-
chrX	53478100	-
chrX	53485100	+
chrX	53758100	+
chrX	54087400	-
chrX	54401400	+
chrX	54573600	+
chrX	54964900	-
chrX	55043100	+
chrX	55204400	-
chrX	55495300	+
chrX	55532000	-
chrX	55760800	+
chrX	55952000	-
chrX	56276100	-
chrX	56856300	-
chrX	57038600	-
chrX	62487100	+
chrX	62697600	+
chrX	62891500	-
chrX	63531900	-
chrX	64112700	-
chrX	64171600	+
chrX	67635300	+
chrX	67965700	+
chrX	69426700	-
chrX	69571000	-
chrX	69592500	-
chrX	70204900	-
chrX	70232700	+
chrX	70318900	-
chrX	70333900	-
chrX	70420100	+
chrX	71709500	-
chrX	72699700	+
chrX	73673000	-
chrX	74061500	-
chrX	74292700	-
chrX	74410600	+
chrX	74659400	+
chrX	75309300	+
chrX	76927800	-
chrX	77041800	+
chrX	79951900	-
chrX	84145500	+
chrX	95826900	-
chrX	99961700	+
chrX	100070600	+
chrX	100193600	-
chrX	100490200	-
chrX	100532500	+
chrX	100549800	-
chrX	100559800	+
chrX	100758900	+
chrX	101740900	+
chrX	101793300	+
chrX	101862500	-
chrX	102396700	-
chrX	102727400	+
chrX	103060200	+
chrX	103103900	+
chrX	103243900	-
chrX	103288300	-
chrX	106248700	-
chrX	106906200	-
chrX	106956300	-
chrX	107221600	-
chrX	108863000	-
chrX	110811400	+
chrX	111810400	-
chrX	111971000	-
chrX	117364400	+
chrX	117514300	+
chrX	118417200	+
chrX	118486700	+
chrX	118889200	-
chrX	119263000	-
chrX	119269000	+
chrX	119503500	+
chrX	119648000	-
chrX	122821900	+
chrX	128805200	-
chrX	128943400	+
chrX	129229900	-
chrX	129301500	+
chrX	129363600	+
chrX	131180800	-
chrX	131441900	+
chrX	131451300	+
chrX	133134400	+
chrX	133422100	+
chrX	133876800	-
chrX	133966200	-
chrX	133994200	-
chrX	134482100	+
chrX	134895500	-
chrX	134981100	-
chrX	135161000	-
chrX	135691100	-
chrX	135819400	+
chrX	135962100	+
chrX	146801000	+
chrX	147270900	-
chrX	149398300	-
chrX	149466700	-
chrX	151861000	+
chrX	153208400	+
chrX	153428800	-
chrX	153632400	-
chrX	153686800	-
chrX	153908700	+
chrX	154146500	-
chrX	154495600	-
chrY	2931300	+
chrY	13526400	+
chrY	14100900	-
chrY	14325300	+
chrY	14372200	+
chrY	14966200	-
chrY	16045900	+
chrY	19697800	+
chrY	21562200	+

Cluster GW8 (in hg18)
chr1	8008900	+
chr1	9164900	-
chr1	15123600	+
chr1	15355900	-
chr1	27211500	+
chr1	31908100	+
chr1	31953200	+
chr1	32703600	-
chr1	32777700	+
chr1	36721000	-
chr1	38003900	+
chr1	38765400	+
chr1	40140700	-
chr1	41083000	+
chr1	44644100	-
chr1	46632400	+
chr1	46705400	-
chr1	46727600	+
chr1	47418300	-
chr1	47469400	+
chr1	47746300	+
chr1	50347900	+
chr1	50572100	-
chr1	50662100	+
chr1	53301200	-
chr1	53977200	-
chr1	55235000	-
chr1	56817000	+
chr1	56883300	+
chr1	58815100	-
chr1	61288700	-
chr1	61295900	-
chr1	64869600	-
chr1	65386900	+
chr1	65503700	+
chr1	66771800	+
chr1	66990600	+
chr1	67546200	-
chr1	68071000	-
chr1	68734800	+
chr1	71285500	-
chr1	75853000	+
chr1	77106600	-
chr1	78284100	+
chr1	83931000	+
chr1	84098500	+
chr1	84237800	-
chr1	85702500	+
chr1	91073800	+
chr1	91089600	-
chr1	92268500	-
chr1	93686500	+
chr1	94283600	+
chr1	98283600	+
chr1	99243000	-
chr1	100777400	+
chr1	107485100	-
chr1	110084300	+
chr1	110428300	+
chr1	110900000	+
chr1	111614900	+
chr1	115986400	+
chr1	116320700	-
chr1	118102600	+
chr1	119672000	-
chr1	145657000	-
chr1	147481700	+
chr1	147490600	-
chr1	149960500	-
chr1	151584500	-
chr1	151918100	+
chr1	156349700	+
chr1	156418100	-
chr1	158307500	-
chr1	159494700	+
chr1	160260100	+
chr1	163680200	+
chr1	164402500	-
chr1	165120900	-
chr1	169487700	-
chr1	175417700	-
chr1	176961300	+
chr1	177979300	-
chr1	178465600	+
chr1	180850900	+
chr1	182040700	+
chr1	182272700	+
chr1	182623000	+
chr1	183970300	-
chr1	196138800	-
chr1	200125300	-
chr1	200182000	+
chr1	201097000	+
chr1	202310000	-
chr1	205694000	+
chr1	207228300	+
chr1	207915600	-
chr1	208551100	+
chr1	209373500	+
chr1	209846500	+
chr1	211190500	+
chr1	211291500	-
chr1	212228700	-
chr1	216405100	-
chr1	219050800	-
chr1	227610300	-
chr1	227634400	-
chr1	230832500	+
chr1	231103500	+
chr1	231817000	+
chr1	232878100	+
chr1	234294500	+
chr1	234625200	+
chr1	237687500	+
chr1	238842100	-
chr1	242147600	-
chr1	243385900	-
chr10	6165200	+
chr10	13521800	+
chr10	13973500	-
chr10	14256200	-
chr10	23257200	-
chr10	26149500	+
chr10	27587600	+
chr10	28327200	+
chr10	29006700	+
chr10	29051100	+
chr10	32036600	+
chr10	33663600	-
chr10	42569700	-
chr10	42749300	-
chr10	44679800	+
chr10	45189600	-
chr10	47974700	+
chr10	50065900	-
chr10	52504000	+
chr10	53744100	+
chr10	60792200	+
chr10	62373700	-
chr10	62882000	+
chr10	63698800	-
chr10	65296600	-
chr10	69661500	-
chr10	71002600	-
chr10	71685000	+
chr10	71871200	-
chr10	73393900	+
chr10	76724500	+
chr10	76831100	+
chr10	79140700	+
chr10	79837200	-
chr10	81654400	+
chr10	89409400	+
chr10	90957300	-
chr10	92607500	-
chr10	93382500	+
chr10	93637000	+
chr10	94439500	-
chr10	95502400	-
chr10	96152900	+
chr10	98124500	+
chr10	100017900	+
chr10	101079600	-
chr10	102231700	+
chr10	102577700	+
chr10	102816800	+
chr10	102870600	+
chr10	102896400	+
chr10	103980600	-
chr10	112393800	-
chr10	115850600	+
chr10	118598900	-
chr10	118754800	-
chr10	118917100	+
chr10	120209400	-
chr10	120344900	+
chr10	122699500	-
chr10	122728500	+
chr10	124211600	+
chr10	125744600	+
chr10	127721200	-
chr10	129595900	-
chr10	130724700	-
chr10	133141800	+
chr10	134905500	-
chr11	4165600	-
chr11	4615000	-
chr11	5797200	-
chr11	6237400	-
chr11	6904200	+
chr11	7651100	+
chr11	7997500	+
chr11	8147200	-
chr11	8241100	+
chr11	8246500	-
chr11	8571900	+
chr11	10909500	-
chr11	11599000	+
chr11	12652400	-
chr11	13941400	+
chr11	14359000	+
chr11	14952400	-
chr11	15052200	+
chr11	16591500	+
chr11	16992500	+
chr11	19219400	-
chr11	20110900	-
chr11	22410800	-
chr11	27678800	+
chr11	33807100	+
chr11	33847800	+
chr11	33871900	+
chr11	35597100	+
chr11	36572200	+
chr11	43846900	+
chr11	43920900	+
chr11	44283600	+
chr11	45317700	+
chr11	46274200	-
chr11	46895900	-
chr11	47164600	+
chr11	60819000	-
chr11	63972900	+
chr11	73699800	+
chr11	75056800	+
chr11	75157700	-
chr11	76062100	-
chr11	76172100	-
chr11	82122000	+
chr11	85071100	+
chr11	86344200	+
chr11	87881900	-
chr11	93885800	-
chr11	94114600	-
chr11	101485700	+
chr11	106967100	+
chr11	109550000	+
chr11	110888500	+
chr11	110916800	+
chr11	112337900	+
chr11	112618600	+
chr11	115136300	-
chr11	117252500	-
chr11	119544600	+
chr11	122571100	+
chr11	122733800	+
chr11	123029900	+
chr11	124244100	-
chr11	124438600	-
chr11	124540900	-
chr11	124870600	-
chr11	128655800	-
chr11	131501700	-
chr12	1513500	-
chr12	2014200	+
chr12	3279500	+
chr12	4838000	-
chr12	4889600	+
chr12	6054300	+
chr12	10766600	-
chr12	12935800	-
chr12	19174300	+
chr12	24606100	+
chr12	24883100	+
chr12	24947400	-
chr12	24992600	+
chr12	26166600	+
chr12	26344000	-
chr12	27377300	-
chr12	31407300	-
chr12	31635100	+
chr12	32443500	+
chr12	32940700	+
chr12	34170700	-
chr12	36818200	+
chr12	40612200	+
chr12	40704600	-
chr12	41163600	-
chr12	46863700	-
chr12	46977300	-
chr12	48228800	+
chr12	48647900	-
chr12	48736700	+
chr12	50271000	-
chr12	52419500	-
chr12	52431100	+
chr12	52675500	+
chr12	52697600	+
chr12	52853500	-
chr12	52868900	+
chr12	60871900	+
chr12	61115300	+
chr12	61312600	-
chr12	61321800	-
chr12	61614600	-
chr12	61830800	+
chr12	62524500	-
chr12	63461200	+
chr12	70119900	-
chr12	75983100	+
chr12	78608300	+
chr12	92509800	-
chr12	92951900	-
chr12	94466600	-
chr12	94708700	+
chr12	95903400	+
chr12	97663500	+
chr12	99492000	-
chr12	101876300	-
chr12	101883500	-
chr12	102413200	+
chr12	104002200	-
chr12	113606500	-
chr12	116758900	+
chr12	116891100	-
chr12	126196400	+
chr12	129827300	-
chr13	19600500	-
chr13	23452400	-
chr13	24219200	+
chr13	26258000	-
chr13	26833900	+
chr13	27425800	+
chr13	28004400	-
chr13	32284200	-
chr13	32758400	-
chr13	34414700	+
chr13	35769600	+
chr13	39075300	-
chr13	43908300	-
chr13	45859100	+
chr13	48203400	+
chr13	48221400	+
chr13	48692200	+
chr13	50694300	+
chr13	52072500	-
chr13	52211600	-
chr13	71338500	+
chr13	74954000	+
chr13	77391300	-
chr13	79814600	+
chr13	97861600	+
chr13	99345900	-
chr13	101851000	-
chr13	105942400	-
chr13	106368600	-
chr13	107946400	+
chr13	108365900	+
chr13	109791700	-
chr13	110564800	+
chr13	111128400	+
chr13	112632900	+
chr14	22891300	+
chr14	24148700	-
chr14	30413500	+
chr14	32478300	+
chr14	33489200	+
chr14	35811300	+
chr14	36195700	+
chr14	36736400	+
chr14	37794600	+
chr14	50097300	-
chr14	50480600	+
chr14	51605400	-
chr14	52487200	-
chr14	53490100	+
chr14	53756100	+
chr14	57688300	+
chr14	58174500	-
chr14	59113500	-
chr14	59407400	-
chr14	59456300	-
chr14	60259400	+
chr14	60725500	+
chr14	61653400	+
chr14	63831500	-
chr14	64079000	-
chr14	64416700	+
chr14	68891500	-
chr14	69825300	+
chr14	70358700	-
chr14	77034600	+
chr14	80972300	-
chr14	88087800	-
chr14	88577400	-
chr14	95156700	+
chr14	96568900	+
chr14	100656500	+
chr15	19111100	+
chr15	19249600	-
chr15	20055300	-
chr15	20444400	+
chr15	32594700	-
chr15	37999900	+
chr15	38332200	+
chr15	38515900	-
chr15	38590700	+
chr15	41597500	+
chr15	42274500	+
chr15	43457500	+
chr15	47043300	-
chr15	47055800	-
chr15	48262000	+
chr15	49156400	+
chr15	53821700	-
chr15	54445200	-
chr15	57451900	+
chr15	60423700	+
chr15	64332500	-
chr15	66584600	-
chr15	66657900	-
chr15	67153900	-
chr15	68554800	-
chr15	68842700	-
chr15	68971700	+
chr15	74139200	-
chr15	76720300	+
chr15	79345200	+
chr15	81113100	+
chr15	81567900	-
chr15	81667000	-
chr15	81907400	-
chr15	87723100	-
chr15	87999700	-
chr15	88158800	+
chr15	89444500	+
chr15	96340300	-
chr15	96908700	+
chr15	99276700	-
chr16	4304700	+
chr16	10182300	+
chr16	13958700	-
chr16	19005100	+
chr16	24529400	-
chr16	55508800	-
chr16	56394400	-
chr16	56827300	+
chr16	65985700	-
chr16	69949700	-
chr16	78619100	-
chr16	79131800	+
chr16	80370500	+
chr16	83926600	-
chr16	85110400	+
chr16	88676600	-
chr17	4434500	-
chr17	5913000	+
chr17	6556800	+
chr17	6675500	+
chr17	7561500	-
chr17	7954500	-
chr17	8960100	-
chr17	9489600	-
chr17	9958600	+
chr17	10688500	+
chr17	15788800	+
chr17	15810200	+
chr17	27618700	-
chr17	29316400	-
chr17	29930600	+
chr17	31146900	-
chr17	33277900	+
chr17	34575200	-
chr17	38190200	+
chr17	39340100	+
chr17	39416200	+
chr17	39990200	-
chr17	41217500	+
chr17	43445000	-
chr17	44010700	+
chr17	44018500	-
chr17	44079400	+
chr17	45401700	-
chr17	45426300	-
chr17	46797200	+
chr17	52111100	+
chr17	52478700	-
chr17	53306400	+
chr17	53682400	-
chr17	53964600	-
chr17	54188600	-
chr17	58084200	-
chr17	58225800	-
chr17	64005800	+
chr17	67537700	+
chr17	68099700	+
chr17	68125500	+
chr17	69410100	-
chr17	70401000	+
chr17	72093400	-
chr17	76953700	+
chr18	648000	+
chr18	2837200	-
chr18	3761200	+
chr18	5881300	-
chr18	6404000	+
chr18	7557000	-
chr18	9007100	+
chr18	11679400	+
chr18	18003900	+
chr18	18969600	-
chr18	19453100	+
chr18	20260600	+
chr18	27492600	+
chr18	27519200	+
chr18	28304900	+
chr18	28604600	+
chr18	32021100	+
chr18	40513900	-
chr18	41609100	+
chr18	42167900	-
chr18	42780600	+
chr18	45342100	-
chr18	52902500	+
chr18	53028600	-
chr18	54013300	+
chr18	55083200	+
chr18	57711900	-
chr18	58143700	+
chr18	59755100	-
chr18	60025900	+
chr18	71003200	-
chr19	6538500	-
chr19	10489700	+
chr19	20023800	+
chr19	21449700	-
chr19	33920200	-
chr19	38247100	-
chr19	38484900	+
chr19	40088100	+
chr19	40741300	-
chr19	44266600	-
chr19	44697800	-
chr19	46729300	+
chr19	47521500	+
chr19	48896900	+
chr19	49016000	+
chr19	51147900	+
chr19	51621300	+
chr19	52642100	-
chr19	54251900	-
chr19	54558100	+
chr19	55343500	-
chr19	55751100	+
chr19	56012800	+
chr19	56031200	-
chr19	59620700	+
chr19	59996800	-
chr2	36400	+
chr2	574900	+
chr2	730000	+
chr2	3805500	+
chr2	17923400	-
chr2	19411300	-
chr2	20730100	-
chr2	23456800	+
chr2	25118700	-
chr2	25208700	-
chr2	25245200	-
chr2	29996700	+
chr2	31492400	+
chr2	32974700	-
chr2	33025800	-
chr2	39041300	-
chr2	40532500	-
chr2	42090300	+
chr2	45023100	-
chr2	54411500	-
chr2	54789900	-
chr2	60635500	+
chr2	62538500	-
chr2	62555700	+
chr2	63139800	-
chr2	69387400	+
chr2	70870300	+
chr2	71357500	-
chr2	71534600	-
chr2	72230400	+
chr2	72943500	-
chr2	73257300	+
chr2	75280900	-
chr2	75498100	-
chr2	85214300	-
chr2	86888600	+
chr2	95375600	-
chr2	101369900	+
chr2	104836300	+
chr2	106253200	-
chr2	106565600	-
chr2	111222300	+
chr2	118698500	-
chr2	119248200	+
chr2	127445600	+
chr2	128148800	+
chr2	130865100	+
chr2	145058100	-
chr2	152569900	-
chr2	152663500	+
chr2	154043000	+
chr2	156886300	+
chr2	162189800	-
chr2	165406300	+
chr2	166359000	+
chr2	167858100	+
chr2	169455500	-
chr2	172087800	+
chr2	172745400	+
chr2	172808000	+
chr2	173649000	+
chr2	173928200	-
chr2	175302700	+
chr2	175577900	+
chr2	176761300	-
chr2	177045600	+
chr2	177126700	+
chr2	178645500	+
chr2	178767300	+
chr2	182230400	-
chr2	183440100	-
chr2	185171200	-
chr2	187267500	-
chr2	191818100	+
chr2	192768100	+
chr2	201158700	+
chr2	205118600	-
chr2	205598900	-
chr2	206847900	-
chr2	207016400	-
chr2	209996900	+
chr2	213724900	-
chr2	217266300	-
chr2	218328900	+
chr2	219614600	-
chr2	220258600	-
chr2	222878900	-
chr2	222891900	-
chr2	222997200	-
chr2	223245400	-
chr2	224974300	+
chr2	227222900	+
chr2	231985000	+
chr2	232498900	-
chr2	233449300	-
chr2	235964400	-
chr2	238048700	+
chr20	2729100	-
chr20	3168100	+
chr20	10602000	-
chr20	11819900	+
chr20	19865900	+
chr20	25797300	-
chr20	29921900	-
chr20	34144200	+
chr20	34206800	-
chr20	35977100	-
chr20	38751300	+
chr20	41569900	+
chr20	43532800	-
chr20	44370000	-
chr20	45057200	-
chr20	47369200	-
chr20	49659800	+
chr20	49817700	-
chr20	49850300	+
chr20	57412200	-
chr20	57580700	-
chr21	14510400	+
chr21	21292100	-
chr21	31637700	+
chr21	33365300	+
chr21	42061300	+
chr21	44955000	+
chr21	46218000	+
chr22	16230500	-
chr22	16887200	-
chr22	23678900	-
chr22	27169000	-
chr22	31607700	+
chr22	32580200	-
chr22	32648200	-
chr22	34640900	+
chr22	40702700	+
chr3	3126500	+
chr3	12813400	-
chr3	19163900	-
chr3	23778500	+
chr3	29298100	-
chr3	31997600	-
chr3	37468600	-
chr3	38666100	+
chr3	40403800	-
chr3	42361900	+
chr3	42923500	-
chr3	42995500	+
chr3	44012900	+
chr3	44075700	-
chr3	45813100	-
chr3	46908300	+
chr3	57088100	-
chr3	57173500	+
chr3	58545700	+
chr3	69517800	+
chr3	72233200	-
chr3	72420600	+
chr3	87220900	+
chr3	89239600	-
chr3	95174800	+
chr3	99023500	-
chr3	99734000	+
chr3	101693900	+
chr3	113061500	+
chr3	113288100	-
chr3	119198500	+
chr3	120236400	-
chr3	124123200	+
chr3	124229200	+
chr3	124650900	-
chr3	125786100	+
chr3	126036400	+
chr3	126088600	+
chr3	127558700	-
chr3	131176400	+
chr3	132564000	-
chr3	134239700	+
chr3	138020800	+
chr3	138233800	+
chr3	139636000	+
chr3	140138500	-
chr3	142590600	-
chr3	142861000	+
chr3	144146300	-
chr3	144165100	+
chr3	147361700	-
chr3	150858700	-
chr3	154035900	-
chr3	155322200	-
chr3	156945300	-
chr3	157806300	+
chr3	159772000	-
chr3	160964300	-
chr3	161426200	+
chr3	169296200	+
chr3	170861500	+
chr3	172228600	+
chr3	173010800	-
chr3	173648900	-
chr3	180651500	+
chr3	184454500	-
chr3	185476600	-
chr3	185581300	+
chr3	185713800	+
chr3	185725500	+
chr3	185784600	-
chr3	186353700	-
chr3	186454900	-
chr3	187268000	+
chr3	187562100	+
chr3	187614100	+
chr3	187703600	-
chr3	191523100	-
chr3	192529800	-
chr3	194118000	-
chr3	195403900	+
chr3	198640100	-
chr4	4906200	-
chr4	4913000	+
chr4	4996800	-
chr4	5104200	-
chr4	5764300	+
chr4	10072600	-
chr4	11039400	+
chr4	15081100	-
chr4	15693900	-
chr4	22126300	-
chr4	24083100	-
chr4	24590600	+
chr4	24699900	-
chr4	26695300	+
chr4	37132000	-
chr4	40326900	-
chr4	40912800	+
chr4	41848800	+
chr4	42094100	-
chr4	44145300	+
chr4	46690100	+
chr4	46728700	-
chr4	48180800	+
chr4	52613200	-
chr4	53312400	-
chr4	53423200	-
chr4	54264100	-
chr4	54467500	-
chr4	54652600	+
chr4	54788200	+
chr4	55219300	-
chr4	56610600	-
chr4	56851500	-
chr4	57241900	+
chr4	57671300	+
chr4	57724300	+
chr4	72180600	-
chr4	72271800	+
chr4	75124000	-
chr4	75184500	-
chr4	75450000	-
chr4	77392000	-
chr4	78038200	-
chr4	78727800	-
chr4	78733600	-
chr4	79691500	+
chr4	81406400	-
chr4	81476600	-
chr4	83938900	+
chr4	85723300	-
chr4	87734900	-
chr4	88032000	+
chr4	90977300	+
chr4	91268200	-
chr4	96689400	+
chr4	101330400	+
chr4	108177100	-
chr4	109903000	+
chr4	110443000	+
chr4	113655700	+
chr4	113846700	+
chr4	114496700	-
chr4	115120600	-
chr4	115739600	-
chr4	122063100	-
chr4	122521300	-
chr4	123091800	-
chr4	123829900	-
chr4	123967800	+
chr4	124645900	+
chr4	125853100	+
chr4	126456300	-
chr4	128922800	+
chr4	129694400	-
chr4	140420000	+
chr4	140747700	-
chr4	141568300	-
chr4	141639100	-
chr4	141896800	+
chr4	142273600	+
chr4	142777100	+
chr4	144477500	+
chr4	146622700	+
chr4	148690900	+
chr4	151219100	+
chr4	152549300	-
chr4	154932500	+
chr4	155474100	-
chr4	155557000	+
chr4	155884400	+
chr4	156808400	-
chr4	156900200	+
chr4	169990300	-
chr4	170428000	+
chr4	174666900	+
chr4	174696000	+
chr4	175375000	+
chr4	175680400	-
chr4	177223600	+
chr4	183298500	-
chr4	185063200	+
chr4	186286400	+
chr4	187303300	+
chr5	10387000	-
chr5	10590700	-
chr5	17270300	-
chr5	31890800	-
chr5	34692300	-
chr5	35265400	+
chr5	36107000	+
chr5	36725900	+
chr5	36761200	-
chr5	36780600	+
chr5	37874000	-
chr5	39460700	+
chr5	43228200	+
chr5	50298800	+
chr5	52320700	+
chr5	52814600	-
chr5	57914200	+
chr5	60609700	-
chr5	65928300	-
chr5	68824700	-
chr5	71887900	+
chr5	72287700	-
chr5	76419000	-
chr5	77183700	-
chr5	77304100	+
chr5	77841900	-
chr5	77979400	+
chr5	79901200	+
chr5	82805600	-
chr5	88021900	+
chr5	89890600	-
chr5	92941900	+
chr5	94646200	+
chr5	95092800	+
chr5	102229200	+
chr5	106935700	-
chr5	108112400	-
chr5	110437400	-
chr5	111782300	-
chr5	112657700	+
chr5	114533800	-
chr5	114543300	+
chr5	115179600	+
chr5	115325900	-
chr5	119828200	+
chr5	124111600	-
chr5	129268700	-
chr5	131375200	+
chr5	131657700	-
chr5	132140900	+
chr5	132177300	-
chr5	132982100	-
chr5	135496600	+
chr5	137605500	+
chr5	139115700	-
chr5	139402700	+
chr5	139722600	+
chr5	140836300	-
chr5	140851400	+
chr5	141062200	+
chr5	141273700	+
chr5	143172700	-
chr5	145195300	-
chr5	145296900	-
chr5	146594000	+
chr5	146813500	+
chr5	149984100	+
chr5	150031500	+
chr5	151046300	+
chr5	152713000	-
chr5	154007500	-
chr5	155089100	-
chr5	157031000	-
chr5	158172400	-
chr5	158465800	+
chr5	158669800	+
chr5	159558600	+
chr5	159730000	-
chr5	162926200	-
chr5	170696000	+
chr5	172654200	-
chr5	174419300	+
chr5	175493500	-
chr5	175990100	-
chr5	179712700	+
chr5	180412700	+
chr6	1261300	+
chr6	6491700	+
chr6	6802500	-
chr6	7206800	-
chr6	7487100	+
chr6	10534300	+
chr6	11152200	-
chr6	12398400	-
chr6	13065400	+
chr6	14033300	-
chr6	15019500	-
chr6	18096100	+
chr6	27173000	-
chr6	27327500	-
chr6	27388000	+
chr6	27465000	-
chr6	27668600	+
chr6	28300800	+
chr6	28519400	-
chr6	28619100	+
chr6	28662600	+
chr6	28734000	-
chr6	29029000	+
chr6	29629400	-
chr6	30052200	+
chr6	36810400	+
chr6	37859200	+
chr6	38790200	-
chr6	39378400	+
chr6	40662700	+
chr6	40716300	+
chr6	41448500	-
chr6	44078200	+
chr6	46246700	-
chr6	46566500	+
chr6	46729000	-
chr6	47384800	+
chr6	49626700	+
chr6	52037000	-
chr6	56824500	+
chr6	56927400	-
chr6	57194600	+
chr6	69401700	-
chr6	70633900	-
chr6	71722800	-
chr6	75971600	+
chr6	76515800	-
chr6	76701000	+
chr6	78232800	+
chr6	80303600	+
chr6	80544200	+
chr6	80636000	+
chr6	83131000	+
chr6	86216500	+
chr6	86632700	-
chr6	88734500	+
chr6	91377800	-
chr6	94185400	+
chr6	97391700	+
chr6	99379400	-
chr6	100143200	+
chr6	105691200	+
chr6	106156600	-
chr6	108546200	-
chr6	108561900	-
chr6	108603600	+
chr6	110843800	-
chr6	110903700	-
chr6	110972500	-
chr6	112445200	+
chr6	112681500	+
chr6	116889800	+
chr6	121799900	+
chr6	122973400	-
chr6	123152600	-
chr6	125517300	-
chr6	125725800	+
chr6	132170800	-
chr6	132313700	-
chr6	134253400	+
chr6	134680300	+
chr6	135237400	-
chr6	136888700	+
chr6	137860100	+
chr6	139055700	-
chr6	149063600	+
chr6	149110300	+
chr6	150286400	-
chr6	150353200	+
chr6	150781400	+
chr6	150963000	+
chr6	152170900	-
chr6	152999800	-
chr6	156531000	-
chr6	164737900	-
chr6	167970400	-
chr6	168994100	-
chr6	169395300	+
chr7	1296900	-
chr7	12409600	+
chr7	12576900	-
chr7	16760400	-
chr7	20336800	-
chr7	22128700	-
chr7	22570300	-
chr7	24290900	-
chr7	24763400	-
chr7	25974300	+
chr7	26382800	+
chr7	26404600	+
chr7	26870800	-
chr7	27167200	-
chr7	28416600	+
chr7	29097800	-
chr7	29201300	+
chr7	29571400	-
chr7	29995600	-
chr7	35542900	+
chr7	37663400	+
chr7	37839700	-
chr7	39420900	-
chr7	41711400	+
chr7	43119200	-
chr7	43320300	+
chr7	48042300	-
chr7	55290200	+
chr7	63986700	-
chr7	68927100	+
chr7	72699900	+
chr7	79602500	+
chr7	87067400	+
chr7	89712600	-
chr7	90518500	-
chr7	90732100	-
chr7	91892300	-
chr7	93862600	-
chr7	94792400	-
chr7	94863900	-
chr7	96494400	+
chr7	100395900	-
chr7	107088800	+
chr7	107430200	-
chr7	113513500	-
chr7	115927000	+
chr7	115938900	+
chr7	115953300	+
chr7	117641400	+
chr7	120284900	-
chr7	120756900	+
chr7	121571200	+
chr7	124192400	+
chr7	127562900	+
chr7	127629500	-
chr7	128572200	-
chr7	130891700	-
chr7	140417800	+
chr7	140858700	-
chr7	147590300	+
chr7	150737500	-
chr7	154337200	-
chr7	154968600	-
chr7	154976100	+
chr7	157071400	-
chr7	157497200	-
chr8	1980300	-
chr8	2578500	+
chr8	6645300	+
chr8	6686300	-
chr8	8596900	-
chr8	11881900	-
chr8	13468900	-
chr8	16929200	-
chr8	17478600	+
chr8	20204900	-
chr8	23595900	+
chr8	24828300	+
chr8	24869700	-
chr8	26427200	+
chr8	31010700	+
chr8	33576600	+
chr8	38505300	-
chr8	38530600	+
chr8	38733700	-
chr8	41286000	-
chr8	41630200	+
chr8	49810300	+
chr8	54016000	-
chr8	56955000	-
chr8	57188100	+
chr8	62214100	+
chr8	68037400	-
chr8	68103800	-
chr8	70908800	-
chr8	75396000	-
chr8	76482400	-
chr8	79741100	-
chr8	79880100	-
chr8	80859100	-
chr8	80966200	-
chr8	82705200	+
chr8	95723400	+
chr8	97226600	+
chr8	99146500	-
chr8	99375500	-
chr8	101240000	-
chr8	103204900	-
chr8	104380400	-
chr8	105670500	+
chr8	106400400	+
chr8	107352100	+
chr8	108578600	+
chr8	110726200	+
chr8	119703100	+
chr8	120498500	-
chr8	125809000	-
chr8	135538100	+
chr8	136538800	-
chr9	2232400	-
chr9	2612300	+
chr9	4480700	-
chr9	14312400	+
chr9	17125300	-
chr9	17568700	+
chr9	19924900	+
chr9	21998800	+
chr9	23581400	-
chr9	38353300	-
chr9	70979200	+
chr9	71476900	+
chr9	74110500	-
chr9	76302100	+
chr9	83494300	+
chr9	87904000	+
chr9	91764600	-
chr9	93707800	+
chr9	94986200	+
chr9	95070500	+
chr9	98677500	+
chr9	99606100	-
chr9	101098500	+
chr9	103507900	-
chr9	108665800	-
chr9	111123100	-
chr9	111721200	-
chr9	112118500	+
chr9	113400800	-
chr9	123301300	-
chr9	124148700	+
chr9	125799300	+
chr9	126573200	-
chr9	127209800	+
chr9	128319000	+
chr9	131239700	-
chrX	67569800	+
chrX	73557100	-
chrX	84385900	+
chrX	102973800	-
chrX	119328000	-
chrX	124166800	+
chrX	133511400	+
chrX	135941700	+
chrX	136338200	-
chrY	15146000	-

Cluster GW9 (in hg18)
chr1	831000	+
chr1	978400	+
chr1	988600	-
chr1	1017000	+
chr1	1048500	+
chr1	1312400	+
chr1	1362300	+
chr1	1443200	-
chr1	1820200	+
chr1	1892100	-
chr1	1967100	+
chr1	2134800	+
chr1	2348000	-
chr1	2420700	-
chr1	2552400	+
chr1	2605500	-
chr1	2619600	-
chr1	2821900	-
chr1	3243500	+
chr1	3314800	-
chr1	3723700	-
chr1	5851000	-
chr1	6427300	-
chr1	6455400	+
chr1	11356200	-
chr1	16892900	+
chr1	19261100	-
chr1	24521800	+
chr1	26560600	+
chr1	30901100	+
chr1	31152700	-
chr1	32003500	+
chr1	38284000	-
chr1	42000000	+
chr1	42100800	-
chr1	42119400	+
chr1	42198600	+
chr1	43797700	-
chr1	44099000	+
chr1	44512300	-
chr1	53333200	-
chr1	109595600	+
chr1	110464400	-
chr1	152952900	-
chr1	158065500	+
chr1	179197600	-
chr1	196448100	-
chr1	199110200	+
chr1	200860900	+
chr1	221564600	+
chr1	226171500	+
chr1	226260300	+
chr1	226566600	+
chr1	226613000	+
chr1	230538300	+
chr10	341200	+
chr10	481500	-
chr10	703500	-
chr10	1206500	-
chr10	1287900	+
chr10	3452100	-
chr10	6303300	-
chr10	22117100	-
chr10	32333700	-
chr10	35351100	+
chr10	43074500	+
chr10	50045200	-
chr10	50620400	+
chr10	70655600	+
chr10	73117600	-
chr10	75945400	-
chr10	80626700	+
chr10	80807800	+
chr10	99515400	-
chr10	115795100	+
chr10	125521400	-
chr10	128949300	-
chr10	131341800	-
chr10	131378500	-
chr10	131418600	+
chr10	132865300	+
chr10	133912600	+
chr10	134091700	-
chr10	134102700	-
chr10	134488700	-
chr10	134500600	-
chr10	134784800	-
chr10	134848100	+
chr10	135013400	+
chr10	135106900	+
chr10	135122200	+
chr10	135373500	-
chr11	359300	+
chr11	539900	-
chr11	712600	+
chr11	1078900	-
chr11	1407000	+
chr11	2411600	-
chr11	3070300	+
chr11	7559500	+
chr11	12099400	+
chr11	12223500	+
chr11	17771200	-
chr11	36406200	+
chr11	47334200	-
chr11	58729400	-
chr11	61103500	-
chr11	65948100	+
chr11	66976400	+
chr11	117307200	+
chr11	122959500	+
chr11	133774000	+
chr11	134033100	+
chr11	134114200	-
chr12	1554400	+
chr12	31763400	-
chr12	50049800	+
chr12	55158100	+
chr12	66260100	+
chr12	88412900	+
chr12	107281500	+
chr12	107513100	-
chr12	115298100	+
chr12	120674000	+
chr12	122563600	+
chr12	124000300	+
chr12	131250500	-
chr12	131370400	-
chr12	131430100	+
chr12	131520800	+
chr12	131542800	+
chr12	131565100	+
chr13	19865200	-
chr13	23829300	+
chr13	27687200	+
chr13	111071400	-
chr13	111677300	-
chr13	112577100	+
chr13	112588200	+
chr13	113148900	-
chr13	113535000	+
chr13	113551900	+
chr13	113636500	+
chr13	113984600	+
chr14	20631000	+
chr14	22597200	+
chr14	68464300	-
chr14	70318600	+
chr14	74783400	-
chr14	92076400	+
chr14	94425900	-
chr14	102641800	+
chr14	103621700	+
chr14	104203700	+
chr14	104543900	-
chr14	104704500	-
chr15	18823300	+
chr15	19963400	+
chr15	20004100	-
chr15	23141400	-
chr15	26023800	-
chr15	47391900	-
chr15	72212100	+
chr15	77172000	+
chr15	88723200	+
chr15	99900300	-
chr16	552500	+
chr16	892600	+
chr16	1003800	+
chr16	1081200	+
chr16	1255200	+
chr16	1320900	-
chr16	1357500	+
chr16	1439200	+
chr16	2026300	-
chr16	2061200	-
chr16	2160900	-
chr16	3078900	+
chr16	4645400	-
chr16	29221700	-
chr16	29930200	+
chr16	46124100	-
chr16	55561600	-
chr16	66341100	+
chr16	67037000	-
chr16	73828300	+
chr16	79183300	+
chr16	85778400	-
chr16	86896500	-
chr16	86974800	+
chr16	87037700	+
chr16	87110000	+
chr16	87411900	+
chr16	87616600	-
chr16	87628800	+
chr16	87788400	+
chr16	88626500	+
chr17	94200	-
chr17	1076100	-
chr17	2544500	+
chr17	2877100	+
chr17	8867700	+
chr17	18090400	-
chr17	24421900	-
chr17	30501700	-
chr17	30584100	+
chr17	34957600	-
chr17	39600500	-
chr17	42175500	-
chr17	53022000	+
chr17	59773800	-
chr17	62885300	+
chr17	70467200	-
chr17	71066400	+
chr17	71129200	+
chr17	72864900	+
chr17	73740700	+
chr17	74550400	-
chr17	74645900	-
chr17	75335500	-
chr17	75403600	+
chr17	75613500	+
chr17	76392100	+
chr17	76628400	+
chr17	76635500	-
chr17	76689700	-
chr17	76726600	-
chr17	77002100	+
chr17	77013600	+
chr17	77705700	+
chr17	78510500	+
chr17	78556500	-
chr18	12420200	-
chr18	13224000	+
chr18	42231900	+
chr18	44237200	+
chr18	45085000	+
chr18	58909500	-
chr18	70087300	-
chr18	70927900	+
chr18	73368500	+
chr18	75658300	-
chr19	353800	-
chr19	449900	+
chr19	1403800	+
chr19	1698500	-
chr19	1704400	-
chr19	2302300	-
chr19	2475200	-
chr19	2742200	+
chr19	3195300	-
chr19	3349400	+
chr19	3733100	-
chr19	3758400	-
chr19	4453200	-
chr19	6021100	+
chr19	6668300	-
chr19	7308900	+
chr19	7662900	+
chr19	7843200	+
chr19	9980700	-
chr19	10110300	+
chr19	10887400	+
chr19	11328200	+
chr19	13036900	-
chr19	13907100	+
chr19	19513800	+
chr19	32988700	+
chr19	38619500	+
chr19	38958200	-
chr19	41073200	+
chr19	42523600	-
chr19	43744800	+
chr19	43896400	+
chr19	45414500	-
chr19	45533900	-
chr19	47115700	+
chr19	50460500	-
chr19	52876900	+
chr19	55891200	-
chr19	57873900	-
chr19	58756500	+
chr19	60231900	+
chr19	60383500	+
chr19	60730600	+
chr19	60915900	-
chr2	595400	+
chr2	2556000	-
chr2	2626500	-
chr2	8456500	+
chr2	8497200	+
chr2	9835300	-
chr2	10550900	-
chr2	10832300	+
chr2	11011000	+
chr2	12913600	-
chr2	23498000	-
chr2	23572600	-
chr2	23749500	-
chr2	43320500	-
chr2	55047800	+
chr2	88845700	-
chr2	109180000	+
chr2	109249600	+
chr2	127517800	-
chr2	217049100	+
chr2	219871000	+
chr2	220126500	+
chr2	232924200	+
chr2	233609000	+
chr2	235088400	+
chr2	240735400	-
chr2	241052700	+
chr2	241231200	-
chr2	241550200	+
chr2	242337500	-
chr20	24996600	+
chr20	25614400	-
chr20	33036400	-
chr20	33371900	+
chr20	35890400	-
chr20	41988400	+
chr20	60230300	+
chr20	60324100	-
chr20	60429400	-
chr20	60599900	-
chr20	61278300	-
chr20	61287400	-
chr20	61427900	-
chr20	62152400	+
chr20	62191100	-
chr20	62198100	-
chr21	32801300	-
chr21	34279900	+
chr21	42098200	-
chr21	42248300	+
chr21	42319800	-
chr21	42586400	+
chr21	42963200	-
chr21	43849500	+
chr21	44030900	-
chr21	44370500	+
chr21	44651200	+
chr21	44754900	-
chr21	45549100	+
chr21	45697600	+
chr21	45743800	-
chr21	45879900	-
chr21	46089300	-
chr21	46313800	+
chr21	46426600	-
chr21	46590400	-
chr21	46598600	+
chr22	18084800	-
chr22	18092500	+
chr22	29529700	+
chr22	36292700	-
chr22	36753700	-
chr22	38290500	+
chr22	38349700	+
chr22	40380600	+
chr22	41931800	-
chr22	43391800	-
chr22	44754800	+
chr22	45062200	-
chr22	47738100	-
chr22	48238700	+
chr22	48802600	+
chr22	49056300	-
chr22	49268400	+
chr22	49469200	+
chr22	49514800	-
chr3	12909100	+
chr3	43110600	-
chr3	43383200	+
chr3	45918600	+
chr3	46925300	-
chr3	49166300	+
chr3	49668800	+
chr3	50503200	-
chr3	51966000	+
chr3	52154100	+
chr3	52335200	-
chr3	52457000	+
chr3	52870900	+
chr3	67519100	+
chr3	128208000	+
chr3	130788100	+
chr3	162514200	+
chr3	162595800	+
chr3	189536800	-
chr3	196311600	-
chr4	807700	-
chr4	1598600	+
chr4	1623900	-
chr4	2276900	+
chr4	3439700	-
chr4	4480900	-
chr4	6214400	-
chr4	6224700	+
chr4	6242800	+
chr4	6330600	-
chr4	6718300	+
chr4	6777900	-
chr4	7917300	+
chr4	8088700	+
chr4	8122800	+
chr4	10575200	+
chr4	37665200	-
chr4	38411600	-
chr4	102557200	-
chr4	109140700	+
chr4	114696500	-
chr4	151391200	-
chr4	177501600	+
chr5	1265200	+
chr5	1274900	-
chr5	1510900	-
chr5	10602500	+
chr5	132132200	+
chr5	139505900	+
chr5	168106200	-
chr5	175245100	+
chr5	175281900	+
chr5	175955600	-
chr5	175978500	-
chr5	176229600	+
chr5	176449500	-
chr6	466800	-
chr6	15750400	+
chr6	16290100	+
chr6	34148400	+
chr6	35504400	+
chr6	37723500	-
chr6	38235100	+
chr6	39282100	+
chr6	105853100	+
chr6	150626900	-
chr6	155263700	-
chr6	158114800	-
chr6	158596600	-
chr6	160457000	-
chr6	161559000	+
chr6	166782100	-
chr6	167235100	+
chr6	169389400	-
chr6	169514600	+
chr7	197200	+
chr7	602600	+
chr7	662500	+
chr7	1072200	+
chr7	1098200	-
chr7	1748500	+
chr7	1817200	-
chr7	2261000	+
chr7	2322900	+
chr7	5304100	+
chr7	36303100	-
chr7	44252400	-
chr7	55628000	-
chr7	64011900	+
chr7	72486900	+
chr7	73548900	+
chr7	98855300	+
chr7	100332400	+
chr7	128318400	-
chr7	132289500	+
chr7	150319200	-
chr7	151150000	+
chr7	154494500	+
chr7	156765600	+
chr7	156919000	+
chr7	157030000	-
chr8	6461700	+
chr8	41872700	-
chr8	48838400	-
chr8	57271800	-
chr8	100428700	+
chr8	129123400	+
chr8	134275900	-
chr8	135726300	+
chr8	140974100	-
chr8	142620700	+
chr8	143375700	+
chr8	143505200	+
chr8	143513300	-
chr8	143547300	-
chr8	143580600	-
chr8	143608600	+
chr8	144142800	-
chr8	144335800	+
chr8	144370300	-
chr8	144560400	-
chr8	145184800	-
chr8	145587600	-
chr8	145816500	+
chr9	34360900	-
chr9	87857800	-
chr9	91058800	+
chr9	96885000	-
chr9	97829400	+
chr9	125313000	+
chr9	129002500	+
chr9	129306400	+
chr9	129325200	-
chr9	131212300	-
chr9	131270600	-
chr9	135350400	-
chr9	135367500	+
chr9	135565200	+
chr9	135770200	-
chr9	136394400	-
chr9	136791800	-
chr9	137257200	-
chr9	137318400	+
chr9	137806300	+
chr9	138011600	+
chr9	138235300	-
chr9	138583600	-
chr9	138600400	-
chr9	138988800	-
chr9	139023500	-
chr9	139167000	-
chr9	139177900	+
chr9	139320400	+
chr9	139371300	+
chr9	139516700	+
chrX	20045400	+
chrX	41645600	-
chrX	48343300	+
chrX	67790100	+
chrX	100631500	-
chrX	152659300	+

Cluster GW10 (in hg18)
chr1	2070000	+
chr1	2120000	+
chr1	2747200	-
chr1	3504500	-
chr1	5406300	-
chr1	6342100	-
chr1	8152400	+
chr1	8586400	+
chr1	8646300	+
chr1	9400000	-
chr1	10499300	+
chr1	11712500	+
chr1	12071200	-
chr1	12133700	-
chr1	12327900	-
chr1	12418400	+
chr1	12455900	-
chr1	12461900	-
chr1	15778100	+
chr1	16165700	-
chr1	16356500	+
chr1	16367800	-
chr1	17385300	+
chr1	19887500	-
chr1	20258700	+
chr1	21478900	+
chr1	22155900	+
chr1	23621900	-
chr1	23727200	+
chr1	23791700	-
chr1	23800800	+
chr1	23811700	-
chr1	24550300	+
chr1	24682700	+
chr1	25162900	-
chr1	25170300	+
chr1	25184100	+
chr1	25876000	-
chr1	26402000	-
chr1	26573600	+
chr1	26999700	-
chr1	27311700	-
chr1	27755700	-
chr1	27808900	-
chr1	27817000	+
chr1	27822800	-
chr1	27832500	+
chr1	31006600	+
chr1	31938200	-
chr1	32087200	-
chr1	32223800	-
chr1	33540500	+
chr1	35777600	-
chr1	36604800	-
chr1	36762300	+
chr1	38269100	+
chr1	38798100	-
chr1	40083800	+
chr1	40272800	+
chr1	41035500	+
chr1	41911800	+
chr1	42030200	+
chr1	42045100	+
chr1	42136200	+
chr1	43273200	-
chr1	44091600	+
chr1	44593500	+
chr1	44935500	-
chr1	45782600	-
chr1	51808000	-
chr1	53532400	+
chr1	53651400	+
chr1	54698500	-
chr1	54719400	+
chr1	54842600	-
chr1	58957000	-
chr1	58994100	-
chr1	59096700	-
chr1	59180400	-
chr1	59206100	+
chr1	59284300	-
chr1	62510600	-
chr1	63890400	-
chr1	66513800	-
chr1	66683700	+
chr1	85092500	+
chr1	85100900	+
chr1	89841900	-
chr1	90092200	+
chr1	91794700	+
chr1	91970400	-
chr1	92110000	+
chr1	92648700	+
chr1	92713600	+
chr1	93229600	-
chr1	94056000	+
chr1	101529500	+
chr1	101550200	+
chr1	108241200	+
chr1	108426900	+
chr1	108990600	+
chr1	109876400	+
chr1	110135100	-
chr1	110147000	-
chr1	111557700	-
chr1	111847800	-
chr1	111937300	-
chr1	111945100	-
chr1	113049300	-
chr1	115407300	-
chr1	115992500	+
chr1	116848300	+
chr1	116859000	+
chr1	117171200	+
chr1	117393300	+
chr1	144455800	-
chr1	145466600	+
chr1	150054600	+
chr1	150064700	+
chr1	150072600	+
chr1	150184500	+
chr1	152704300	+
chr1	153023600	+
chr1	158147300	-
chr1	158665200	+
chr1	158697100	-
chr1	158804100	+
chr1	158910200	-
chr1	159455000	+
chr1	165838400	+
chr1	166040500	+
chr1	166644900	-
chr1	166768000	-
chr1	167280200	-
chr1	170594400	+
chr1	170875000	+
chr1	170982200	+
chr1	171000500	+
chr1	171441800	-
chr1	179385000	+
chr1	179395200	+
chr1	179633600	+
chr1	180323400	-
chr1	180495500	-
chr1	181825300	-
chr1	181888100	-
chr1	182192500	-
chr1	183575200	-
chr1	183796400	-
chr1	196402500	-
chr1	197409000	-
chr1	198389600	-
chr1	201072100	+
chr1	201507300	-
chr1	201513800	+
chr1	201526100	-
chr1	202697400	+
chr1	202814800	+
chr1	204495300	+
chr1	204977400	+
chr1	205009600	-
chr1	205024500	+
chr1	205069200	+
chr1	205311200	-
chr1	205576300	-
chr1	206113300	-
chr1	210495900	+
chr1	212668500	-
chr1	220019000	-
chr1	221430200	+
chr1	221469900	+
chr1	221488400	+
chr1	222424100	-
chr1	222752100	+
chr1	222878300	+
chr1	223699900	-
chr1	224151900	-
chr1	224699900	-
chr1	225053700	-
chr1	226139600	+
chr1	226996400	-
chr1	227013200	+
chr1	227066500	+
chr1	227141300	-
chr1	227260400	+
chr1	227332600	-
chr1	227528500	-
chr1	229815100	+
chr1	230121300	-
chr1	231127100	-
chr1	232927000	-
chr1	232933500	+
chr1	232970900	+
chr1	233045700	-
chr1	233129400	-
chr1	233157400	+
chr1	233167100	+
chr1	233181800	-
chr1	234184400	+
chr1	245637100	+
chr10	506100	+
chr10	3699500	-
chr10	3789900	-
chr10	3835700	+
chr10	3842500	-
chr10	4113000	+
chr10	4868300	-
chr10	5350000	-
chr10	6120100	-
chr10	6134900	-
chr10	6150600	+
chr10	6353000	-
chr10	6682900	-
chr10	7275200	+
chr10	11223900	-
chr10	11483200	+
chr10	13869700	-
chr10	13877400	+
chr10	14588800	+
chr10	15267000	+
chr10	16522800	+
chr10	17108300	-
chr10	22066400	-
chr10	22959700	-
chr10	22979000	-
chr10	23012300	-
chr10	30010300	+
chr10	30785100	-
chr10	30822400	+
chr10	31121200	-
chr10	33466000	-
chr10	42937100	-
chr10	43109200	+
chr10	44729500	-
chr10	44784000	-
chr10	47140000	+
chr10	48047500	+
chr10	48081800	+
chr10	48098300	-
chr10	50187800	+
chr10	51942200	+
chr10	62003400	-
chr10	63539400	-
chr10	64120500	+
chr10	64163200	-
chr10	70474600	-
chr10	70487000	+
chr10	70757500	+
chr10	70856200	-
chr10	70937200	+
chr10	71562000	+
chr10	71862700	-
chr10	72006200	-
chr10	73097800	-
chr10	73144600	-
chr10	73165700	+
chr10	73564600	+
chr10	73745900	-
chr10	75464000	+
chr10	76444300	+
chr10	80455600	-
chr10	80478000	+
chr10	82194400	-
chr10	85927600	-
chr10	85940600	+
chr10	87362200	+
chr10	88161300	-
chr10	89815200	-
chr10	89842200	-
chr10	89867200	-
chr10	90020900	+
chr10	90575800	-
chr10	91082900	-
chr10	95162500	+
chr10	99163500	-
chr10	99531300	+
chr10	99619000	+
chr10	102260500	+
chr10	103308200	+
chr10	104223200	+
chr10	104362600	+
chr10	104384800	+
chr10	105497900	-
chr10	105514000	-
chr10	105550200	+
chr10	112164700	+
chr10	112367900	-
chr10	112489600	-
chr10	112533500	-
chr10	112553000	-
chr10	112584100	-
chr10	112600000	+
chr10	112613900	+
chr10	114139100	+
chr10	116294000	-
chr10	120881300	-
chr10	121228300	+
chr10	121244500	-
chr10	121251400	+
chr10	121266700	-
chr10	124265500	-
chr10	124300400	+
chr10	126143800	+
chr10	129784200	-
chr10	129791600	+
chr10	130746000	-
chr10	134111000	+
chr10	134927300	+
chr11	1822800	-
chr11	2876500	+
chr11	2992400	-
chr11	6416600	-
chr11	6624400	+
chr11	10388000	-
chr11	10602100	-
chr11	11127900	+
chr11	12064700	-
chr11	12202000	+
chr11	12214500	-
chr11	13918500	+
chr11	14249200	+
chr11	16964700	-
chr11	19701600	-
chr11	34426600	-
chr11	35061400	-
chr11	35084500	+
chr11	35104500	-
chr11	36387800	-
chr11	36429200	-
chr11	44587500	+
chr11	44725300	+
chr11	44956000	-
chr11	45052000	-
chr11	45058500	-
chr11	46296500	+
chr11	46308800	-
chr11	47133200	+
chr11	47983700	+
chr11	47998000	+
chr11	48086300	-
chr11	56998300	+
chr11	57316300	-
chr11	58796900	+
chr11	60585600	-
chr11	60596100	-
chr11	61208400	-
chr11	61358300	+
chr11	62080600	+
chr11	63084400	+
chr11	63476500	-
chr11	65841900	+
chr11	65852200	+
chr11	66869400	+
chr11	67170900	-
chr11	67855000	+
chr11	68604000	+
chr11	68646700	+
chr11	68835900	+
chr11	68859100	+
chr11	72009700	+
chr11	72542200	-
chr11	73354200	+
chr11	73419900	-
chr11	74531900	-
chr11	74564000	-
chr11	74649700	+
chr11	74717800	-
chr11	74728500	+
chr11	74864400	+
chr11	74896800	+
chr11	75190600	-
chr11	75677100	+
chr11	76053600	+
chr11	76156900	+
chr11	76471700	-
chr11	87788100	-
chr11	87797300	+
chr11	92902500	-
chr11	94297800	-
chr11	94307100	-
chr11	95385600	-
chr11	95448600	+
chr11	95513500	-
chr11	95631500	-
chr11	95679800	+
chr11	104252300	-
chr11	107216400	+
chr11	110284500	-
chr11	111673800	-
chr11	113479700	+
chr11	113536400	+
chr11	113558800	+
chr11	113592300	-
chr11	116229000	+
chr11	117191300	-
chr11	117199300	+
chr11	117346200	-
chr11	117426600	-
chr11	117695400	-
chr11	118068800	-
chr11	118081500	+
chr11	118252500	-
chr11	118316200	+
chr11	118642900	-
chr11	118682300	-
chr11	120333700	-
chr11	120704500	-
chr11	120794200	+
chr11	122219800	-
chr11	122459800	-
chr11	122758100	+
chr11	125458800	+
chr11	125840900	-
chr11	127717600	-
chr11	127986800	-
chr11	128003500	+
chr11	128220200	+
chr11	129098300	-
chr11	131733400	-
chr11	132094400	+
chr12	519100	-
chr12	653100	+
chr12	665100	+
chr12	706200	+
chr12	1468600	-
chr12	1895400	-
chr12	3872500	-
chr12	3930600	-
chr12	3942100	-
chr12	3953300	+
chr12	4098000	+
chr12	4132800	+
chr12	4195400	-
chr12	4808900	+
chr12	6120400	+
chr12	6210600	-
chr12	6406000	+
chr12	6592000	+
chr12	9752000	-
chr12	11776000	+
chr12	11810300	-
chr12	11856700	+
chr12	12486300	+
chr12	12511800	+
chr12	12519800	-
chr12	12571800	+
chr12	13210200	-
chr12	14299700	-
chr12	15038900	+
chr12	19526900	-
chr12	28267100	+
chr12	31973900	-
chr12	45140200	+
chr12	45957700	-
chr12	46088500	-
chr12	46512800	-
chr12	48317300	-
chr12	48342400	+
chr12	48351300	-
chr12	48868700	+
chr12	49195200	-
chr12	50163700	-
chr12	50836100	-
chr12	51266100	-
chr12	51296200	-
chr12	51783300	-
chr12	55691600	-
chr12	55727900	-
chr12	56578500	+
chr12	66129600	+
chr12	66163500	-
chr12	66196200	-
chr12	66310100	-
chr12	66817200	+
chr12	66856400	-
chr12	67054700	-
chr12	74659000	-
chr12	87957900	-
chr12	88798900	+
chr12	90928800	+
chr12	91296800	+
chr12	92366200	-
chr12	93200300	+
chr12	94965000	+
chr12	100589500	+
chr12	105671700	+
chr12	105677800	+
chr12	106291400	+
chr12	106411000	-
chr12	107563800	+
chr12	108786500	-
chr12	108811000	-
chr12	109512900	+
chr12	109599500	+
chr12	112130700	-
chr12	115517000	-
chr12	115595900	-
chr12	115985800	-
chr12	120146100	-
chr12	122157400	+
chr12	122170600	+
chr12	122182800	-
chr12	123057100	-
chr12	123659200	+
chr12	130561600	+
chr12	131230400	-
chr13	19884600	+
chr13	20816400	+
chr13	21578900	+
chr13	24090600	+
chr13	24098200	-
chr13	24157100	+
chr13	25319600	-
chr13	25347000	-
chr13	25378000	+
chr13	25446300	-
chr13	25955700	-
chr13	26766200	-
chr13	29861400	+
chr13	29881000	-
chr13	30518000	-
chr13	31880400	-
chr13	39618200	+
chr13	39662700	-
chr13	39694600	+
chr13	39956500	-
chr13	41827300	-
chr13	41867800	+
chr13	44769700	+
chr13	45656300	+
chr13	45768800	+
chr13	45813500	-
chr13	47977700	+
chr13	48277900	-
chr13	49579300	-
chr13	50893800	-
chr13	51116700	-
chr13	93773900	+
chr13	96789300	+
chr13	98817600	-
chr13	98831600	-
chr13	98883300	-
chr13	109975400	-
chr13	113945500	+
chr14	19979200	-
chr14	20028200	-
chr14	20206200	-
chr14	23150000	-
chr14	24022700	+
chr14	24213000	-
chr14	31483800	+
chr14	34825400	-
chr14	34872600	+
chr14	34906300	+
chr14	34955300	+
chr14	49482800	-
chr14	49576700	+
chr14	50321500	+
chr14	50329000	-
chr14	54309900	+
chr14	54639600	-
chr14	54971000	-
chr14	61207800	-
chr14	63981100	+
chr14	64261300	-
chr14	64308200	-
chr14	65387800	-
chr14	65465200	-
chr14	67798000	-
chr14	67819300	+
chr14	68020800	+
chr14	68032300	-
chr14	68221700	+
chr14	68248700	-
chr14	68270500	-
chr14	68308100	-
chr14	70182900	-
chr14	70188900	-
chr14	70782900	+
chr14	71977300	-
chr14	72055000	+
chr14	72253100	+
chr14	74110500	-
chr14	74329300	+
chr14	74403600	+
chr14	74766600	-
chr14	75078700	+
chr14	76304600	-
chr14	76318700	+
chr14	76403600	+
chr14	76440700	+
chr14	76463900	-
chr14	87564000	-
chr14	88136000	-
chr14	89218000	-
chr14	90604300	+
chr14	90789900	-
chr14	91426400	-
chr14	92123100	+
chr14	93694100	+
chr14	95563800	-
chr14	95662700	+
chr14	96657200	-
chr14	96944600	+
chr14	97155000	-
chr14	97243200	+
chr14	97260300	+
chr14	97530700	-
chr14	97712900	+
chr14	97745900	+
chr14	97980700	-
chr14	98520300	+
chr14	98972600	-
chr14	99061500	-
chr14	99594800	+
chr14	99942800	-
chr14	100084000	+
chr14	100153600	-
chr14	100176500	+
chr14	101132300	+
chr14	101243300	+
chr14	101268100	-
chr14	102485300	+
chr14	104614400	+
chr14	106226000	-
chr14	106240800	+
chr14	106250800	-
chr15	19968500	-
chr15	19985000	-
chr15	19992100	+
chr15	27169200	+
chr15	29347100	+
chr15	36691300	-
chr15	36751600	+
chr15	36768000	-
chr15	37708100	+
chr15	38127700	-
chr15	38177800	+
chr15	38572900	+
chr15	39973700	+
chr15	40014200	+
chr15	40687700	+
chr15	50216200	+
chr15	50314500	+
chr15	50341600	-
chr15	50543700	+
chr15	53320100	-
chr15	53362800	-
chr15	54287600	-
chr15	56411400	-
chr15	56437700	-
chr15	56553100	-
chr15	56564800	-
chr15	56597600	-
chr15	58879500	+
chr15	59249400	-
chr15	61516100	+
chr15	61553000	-
chr15	61939400	+
chr15	61971700	+
chr15	61985500	-
chr15	62018400	+
chr15	62050300	+
chr15	62089600	+
chr15	62962600	+
chr15	63912700	-
chr15	65320200	+
chr15	65863500	-
chr15	67139300	-
chr15	67548200	-
chr15	67748200	-
chr15	68338000	-
chr15	68504000	+
chr15	68515400	+
chr15	68531200	+
chr15	68608600	-
chr15	72021800	-
chr15	72061700	-
chr15	72463800	-
chr15	72482300	+
chr15	73123100	-
chr15	73686500	-
chr15	75067900	+
chr15	75259100	-
chr15	76182900	-
chr15	76841400	-
chr15	78112300	+
chr15	83332800	-
chr15	83375200	+
chr15	83660300	+
chr15	84051600	-
chr15	84998200	-
chr15	87248300	+
chr15	88403500	+
chr15	88536600	-
chr15	88942100	+
chr15	88964300	+
chr15	89201400	-
chr15	91184600	-
chr15	94078800	+
chr15	94086200	-
chr15	97313100	+
chr15	97782800	+
chr15	98078400	+
chr15	98469500	+
chr15	98587300	+
chr15	98688500	-
chr15	98877900	+
chr15	99485000	-
chr15	99539000	-
chr16	28500	+
chr16	1516000	-
chr16	1983400	+
chr16	2998100	+
chr16	3090400	-
chr16	3500200	-
chr16	4072100	+
chr16	4654300	+
chr16	10872000	+
chr16	11053200	+
chr16	11095300	+
chr16	11106500	+
chr16	11115700	+
chr16	11265500	-
chr16	11313700	+
chr16	11497900	+
chr16	12082900	+
chr16	12460600	+
chr16	17304400	-
chr16	17361500	-
chr16	17418200	-
chr16	19616100	-
chr16	20763300	-
chr16	21566300	+
chr16	23745000	+
chr16	23783200	-
chr16	23869000	+
chr16	23878600	-
chr16	23933400	+
chr16	27113900	+
chr16	27156600	-
chr16	27286200	+
chr16	27297300	-
chr16	28229100	-
chr16	28927900	+
chr16	28948000	+
chr16	30377300	+
chr16	45608500	-
chr16	48033100	-
chr16	48054900	-
chr16	48987000	+
chr16	49218200	+
chr16	49275700	-
chr16	52110000	+
chr16	52336600	-
chr16	54818600	+
chr16	54854000	-
chr16	55452000	-
chr16	55717800	+
chr16	55733500	-
chr16	56067900	-
chr16	56121900	-
chr16	56183800	+
chr16	56768300	+
chr16	65576200	+
chr16	66139400	-
chr16	66655500	+
chr16	66661700	-
chr16	66864800	-
chr16	66961800	-
chr16	66971100	+
chr16	68999700	+
chr16	73666600	+
chr16	73690900	+
chr16	73702000	+
chr16	77681900	-
chr16	77972400	+
chr16	78008100	-
chr16	78209900	+
chr16	79156200	-
chr16	79172100	-
chr16	81246400	+
chr16	83105800	+
chr16	83695900	+
chr16	83901500	+
chr16	84039900	+
chr16	84527500	+
chr16	86487800	-
chr16	87286000	+
chr16	87385300	-
chr16	88379200	-
chr17	1051700	-
chr17	1613700	+
chr17	1721300	+
chr17	2113300	+
chr17	3588800	+
chr17	3601100	+
chr17	3646100	+
chr17	3766600	+
chr17	4026600	-
chr17	7712200	+
chr17	8237400	+
chr17	14041000	+
chr17	14049500	+
chr17	16756000	+
chr17	16832800	-
chr17	17025900	+
chr17	17149300	+
chr17	17248700	-
chr17	17693300	+
chr17	18765500	+
chr17	18799200	+
chr17	19128800	-
chr17	19308100	-
chr17	19615500	+
chr17	20078600	-
chr17	22825500	-
chr17	24334200	-
chr17	24506200	+
chr17	25714700	+
chr17	27871100	+
chr17	30192800	-
chr17	30374400	-
chr17	30528000	+
chr17	30819000	+
chr17	32105900	+
chr17	32520900	+
chr17	34504800	-
chr17	35089400	+
chr17	35117400	-
chr17	35204100	+
chr17	35372900	-
chr17	35521100	+
chr17	35717200	-
chr17	35836300	-
chr17	35889900	+
chr17	35900500	+
chr17	35925600	-
chr17	35943800	+
chr17	35991900	+
chr17	36818600	+
chr17	39089500	-
chr17	43153700	+
chr17	43873000	-
chr17	44649700	-
chr17	44820600	-
chr17	45128400	+
chr17	45616100	+
chr17	46342100	+
chr17	46351400	+
chr17	46862800	+
chr17	53037800	+
chr17	53602300	+
chr17	54798300	-
chr17	59137700	+
chr17	59385800	-
chr17	60135400	+
chr17	60600800	-
chr17	60608800	-
chr17	60950000	-
chr17	61011300	-
chr17	62684900	-
chr17	62816900	+
chr17	63594000	-
chr17	63854900	-
chr17	63864800	-
chr17	63891800	-
chr17	63906300	+
chr17	68234700	+
chr17	69099800	-
chr17	69230700	-
chr17	70185500	+
chr17	70299100	-
chr17	70994900	+
chr17	71043000	+
chr17	71760200	-
chr17	72167400	+
chr17	72490500	+
chr17	72506100	+
chr17	72850800	-
chr17	72872300	-
chr17	73354800	-
chr17	73766100	+
chr17	73822500	-
chr17	73851700	-
chr17	75525100	+
chr17	76256000	+
chr17	76292100	-
chr17	76371600	+
chr17	76378700	-
chr17	76436100	+
chr17	76683500	-
chr17	77854100	+
chr18	2873100	+
chr18	2881400	+
chr18	10550000	+
chr18	12830600	+
chr18	18905300	+
chr18	19093900	+
chr18	19572300	+
chr18	19705800	-
chr18	19801600	-
chr18	19837700	+
chr18	27919200	-
chr18	31451000	-
chr18	40559200	+
chr18	41500100	-
chr18	41990400	-
chr18	42457500	+
chr18	44620100	-
chr18	44649300	-
chr18	44949900	-
chr18	54128200	+
chr18	58917100	-
chr18	59024900	+
chr18	65728600	+
chr18	72981500	+
chr19	1983900	-
chr19	3106300	-
chr19	3764800	+
chr19	6629400	-
chr19	7677400	-
chr19	8165600	+
chr19	11021200	-
chr19	11508300	-
chr19	13212800	-
chr19	13852800	-
chr19	14121300	+
chr19	14478100	-
chr19	15643900	+
chr19	16042100	+
chr19	16233600	+
chr19	16973600	+
chr19	17767400	-
chr19	17775000	+
chr19	17993400	+
chr19	19179300	+
chr19	19562400	-
chr19	34804700	+
chr19	36523100	+
chr19	38343100	+
chr19	40387100	-
chr19	43231100	+
chr19	43916900	+
chr19	44420700	-
chr19	45132800	-
chr19	46025000	-
chr19	47073400	-
chr19	47319000	+
chr19	48634800	+
chr19	48758100	+
chr19	51807000	+
chr19	52290400	-
chr19	52341200	+
chr19	52372000	-
chr19	54604400	+
chr19	55092000	+
chr19	60245300	-
chr2	308500	-
chr2	1148400	+
chr2	1501500	-
chr2	3206400	-
chr2	6968300	+
chr2	7114900	+
chr2	7509500	+
chr2	7625100	+
chr2	7729600	+
chr2	8369100	-
chr2	8539500	-
chr2	8580200	+
chr2	8601500	-
chr2	9760700	+
chr2	9793500	-
chr2	9856600	+
chr2	10199100	+
chr2	12352500	-
chr2	12360600	+
chr2	12874900	-
chr2	15200100	-
chr2	16023200	+
chr2	16668500	-
chr2	16695100	-
chr2	25362100	+
chr2	27172300	+
chr2	28424500	-
chr2	28436100	-
chr2	28455100	+
chr2	28500000	+
chr2	28622000	+
chr2	28670600	+
chr2	28697600	+
chr2	28757900	-
chr2	30391900	-
chr2	30410800	+
chr2	30438300	-
chr2	31395400	+
chr2	37471000	+
chr2	37537400	-
chr2	37696400	+
chr2	37706900	-
chr2	37716500	-
chr2	37763500	+
chr2	37972600	-
chr2	38734900	-
chr2	42180300	-
chr2	43140300	-
chr2	43211300	+
chr2	43250400	-
chr2	43275200	+
chr2	45972100	-
chr2	46260200	-
chr2	46320000	+
chr2	46432400	+
chr2	46616200	-
chr2	60888400	-
chr2	60900000	+
chr2	64748500	-
chr2	65382000	-
chr2	68469000	-
chr2	68802600	-
chr2	68989000	+
chr2	69773900	+
chr2	69856100	-
chr2	70027100	+
chr2	70149500	+
chr2	70189800	-
chr2	70604900	-
chr2	70745200	+
chr2	71082000	-
chr2	73977700	-
chr2	74051600	-
chr2	74371300	+
chr2	84562600	-
chr2	84822000	+
chr2	84940300	-
chr2	84945500	-
chr2	84975700	-
chr2	85947100	-
chr2	86079500	+
chr2	86117100	-
chr2	86160900	+
chr2	86306000	+
chr2	95086100	-
chr2	95091400	+
chr2	96187700	-
chr2	96193600	+
chr2	96271100	+
chr2	96437300	-
chr2	96831300	+
chr2	96925200	+
chr2	96970100	+
chr2	96990600	-
chr2	98811900	+
chr2	100218400	-
chr2	101313100	+
chr2	101469100	+
chr2	101606000	+
chr2	105910200	+
chr2	106040800	-
chr2	109331500	+
chr2	111321600	-
chr2	113108600	-
chr2	113641800	-
chr2	113726400	-
chr2	114351300	-
chr2	114361300	-
chr2	128670400	+
chr2	131630800	-
chr2	134708200	-
chr2	135144500	+
chr2	136524000	-
chr2	136531000	+
chr2	136712100	+
chr2	136755700	-
chr2	144203900	+
chr2	149001800	+
chr2	149014900	-
chr2	149020200	-
chr2	157892700	-
chr2	158348600	+
chr2	161217100	-
chr2	162560100	-
chr2	169035300	+
chr2	169147300	-
chr2	169321800	+
chr2	169671600	-
chr2	171813400	-
chr2	175181500	-
chr2	175290400	-
chr2	175326300	+
chr2	175351500	+
chr2	191231400	-
chr2	192210900	+
chr2	196847800	-
chr2	196862000	-
chr2	197779200	-
chr2	197786000	+
chr2	198466300	+
chr2	201029200	-
chr2	201986800	+
chr2	204314200	+
chr2	204376600	+
chr2	204385200	-
chr2	204579400	-
chr2	216631100	-
chr2	216923200	-
chr2	218183600	-
chr2	218740300	-
chr2	224599400	-
chr2	228037300	+
chr2	230891800	+
chr2	230908500	+
chr2	231175500	+
chr2	231216800	+
chr2	231231400	-
chr2	231236900	-
chr2	231546900	+
chr2	232118800	-
chr2	232883000	+
chr2	232895300	+
chr2	233813400	+
chr2	233973500	-
chr2	234563300	+
chr2	234675100	+
chr2	234790400	-
chr2	234865400	+
chr2	234874400	-
chr2	234881900	+
chr2	234945800	-
chr2	235011100	+
chr2	235038800	+
chr2	238006200	-
chr2	238190600	+
chr2	239068000	-
chr2	239909100	-
chr2	239914200	-
chr2	240189300	-
chr2	240212800	-
chr2	240338600	-
chr2	240499800	-
chr20	784700	+
chr20	1215800	-
chr20	1229400	+
chr20	1548300	+
chr20	1593300	+
chr20	1602400	-
chr20	2671100	+
chr20	3030700	+
chr20	3050300	-
chr20	3861900	-
chr20	4011500	-
chr20	16637400	+
chr20	17540100	-
chr20	17887600	+
chr20	18666600	+
chr20	20381700	-
chr20	23070700	+
chr20	23849000	-
chr20	23974000	+
chr20	25019600	-
chr20	30336800	+
chr20	30580200	+
chr20	30847700	+
chr20	31730500	+
chr20	31910600	+
chr20	34918400	+
chr20	35377700	+
chr20	35406600	+
chr20	36902800	-
chr20	39053400	-
chr20	41814200	+
chr20	42091500	-
chr20	42679700	+
chr20	42705800	+
chr20	44267700	-
chr20	48431600	+
chr20	48868700	-
chr20	49455300	-
chr20	49474400	-
chr20	51257900	+
chr20	51801400	-
chr20	51917000	+
chr20	51984900	+
chr20	51998800	+
chr20	54427000	-
chr20	54437800	+
chr20	55460000	+
chr20	55666600	-
chr20	56642900	+
chr20	57185800	+
chr20	57262100	-
chr20	57394300	-
chr20	60818900	-
chr20	61852500	+
chr21	15345300	-
chr21	24262900	+
chr21	29126900	-
chr21	29374000	+
chr21	31471300	-
chr21	33226300	+
chr21	33480000	+
chr21	33499000	+
chr21	33587400	+
chr21	33677200	+
chr21	34270700	-
chr21	35002300	-
chr21	35521300	+
chr21	38618300	-
chr21	39298900	-
chr21	42382400	-
chr21	42486200	+
chr21	42754000	-
chr21	43643800	+
chr21	44137700	+
chr21	44398600	-
chr21	45795400	-
chr21	45860500	-
chr21	46013000	+
chr21	46795700	-
chr22	16118900	+
chr22	16127800	-
chr22	16141800	+
chr22	16165300	-
chr22	16843500	-
chr22	17945100	-
chr22	18241600	+
chr22	18259200	+
chr22	18610100	+
chr22	19148400	-
chr22	21393500	-
chr22	21426100	-
chr22	21462100	+
chr22	21538600	-
chr22	21791900	-
chr22	23803000	-
chr22	28922400	+
chr22	29001600	+
chr22	29956200	-
chr22	29996800	-
chr22	30868800	-
chr22	31449300	+
chr22	34056000	+
chr22	35131500	+
chr22	35161900	+
chr22	35534200	+
chr22	35806200	-
chr22	35848400	-
chr22	35888600	-
chr22	35912200	+
chr22	37816800	-
chr22	37823000	+
chr22	37900500	+
chr22	38173500	-
chr22	39016200	-
chr22	39050800	+
chr22	39218900	+
chr22	39454700	+
chr22	40588500	+
chr22	41027000	-
chr22	43438900	-
chr22	45014600	-
chr22	45524100	-
chr22	48710400	+
chr3	4757500	+
chr3	4842700	-
chr3	4851200	-
chr3	4925300	-
chr3	9187000	+
chr3	9196800	+
chr3	9796300	+
chr3	9919300	-
chr3	10211700	-
chr3	10216100	-
chr3	10241400	+
chr3	10412900	-
chr3	10454100	-
chr3	10620300	-
chr3	11307900	+
chr3	11786900	+
chr3	13086000	+
chr3	13363500	-
chr3	13670700	+
chr3	13883000	-
chr3	14161300	-
chr3	14394200	-
chr3	14439800	-
chr3	14897400	+
chr3	14975400	+
chr3	15378300	+
chr3	16498900	+
chr3	16865900	+
chr3	18743700	-
chr3	27655800	+
chr3	31239900	+
chr3	31247500	-
chr3	32352100	-
chr3	32388800	-
chr3	32431400	+
chr3	32449300	+
chr3	32456200	-
chr3	33080200	-
chr3	38391100	+
chr3	38536400	-
chr3	39144800	-
chr3	39284900	-
chr3	39378400	-
chr3	41134700	-
chr3	42098100	+
chr3	42230700	-
chr3	42404600	-
chr3	42764100	-
chr3	42952300	+
chr3	43196800	+
chr3	43212200	+
chr3	43406700	-
chr3	43772700	-
chr3	45126700	+
chr3	45135300	-
chr3	45183700	+
chr3	45924300	+
chr3	45931900	+
chr3	46114200	+
chr3	46315300	+
chr3	46385700	-
chr3	48285600	-
chr3	48531400	+
chr3	49615200	+
chr3	49919000	-
chr3	50462400	+
chr3	50586100	+
chr3	50603900	-
chr3	56934200	+
chr3	57775700	+
chr3	57944400	+
chr3	58077100	-
chr3	58432300	-
chr3	59385500	-
chr3	66622900	+
chr3	67871700	+
chr3	71554500	-
chr3	72442200	+
chr3	72470000	-
chr3	72477600	+
chr3	73097700	-
chr3	103138700	-
chr3	103260300	-
chr3	109177800	-
chr3	109335100	+
chr3	112512700	+
chr3	113204200	+
chr3	113674500	+
chr3	113683000	-
chr3	113877900	+
chr3	115433200	-
chr3	116366400	+
chr3	120407500	-
chr3	124292700	+
chr3	124725000	-
chr3	129315500	+
chr3	129498500	-
chr3	129515400	-
chr3	130217800	-
chr3	130779000	+
chr3	134692900	-
chr3	142440000	-
chr3	142615500	-
chr3	144371600	+
chr3	152371500	+
chr3	152410700	+
chr3	154485700	-
chr3	158329800	-
chr3	159924200	-
chr3	172467400	-
chr3	172695500	-
chr3	173308300	+
chr3	173717800	-
chr3	173737800	-
chr3	178258300	-
chr3	178798200	+
chr3	178842700	+
chr3	179145400	-
chr3	184486600	-
chr3	187455700	-
chr3	187719500	+
chr3	189175800	-
chr3	189187900	+
chr3	190333200	-
chr3	190556100	+
chr3	191786900	+
chr3	195112300	-
chr3	196429600	+
chr3	197404200	-
chr3	197485500	+
chr3	197823800	-
chr4	2253500	-
chr4	2759100	-
chr4	2769700	+
chr4	3017000	-
chr4	7824000	-
chr4	7881200	-
chr4	9734400	+
chr4	9772400	-
chr4	11092000	-
chr4	15367200	+
chr4	25470600	-
chr4	25815200	+
chr4	26614700	-
chr4	37458100	+
chr4	37809700	-
chr4	37921200	+
chr4	38168100	-
chr4	38208200	-
chr4	39932600	-
chr4	39943900	-
chr4	39962100	-
chr4	40370000	-
chr4	42368400	-
chr4	47229000	-
chr4	47834900	-
chr4	53386800	+
chr4	55502900	-
chr4	78134100	+
chr4	82645900	-
chr4	84130200	-
chr4	84223700	+
chr4	90457800	-
chr4	99587300	-
chr4	99742900	-
chr4	109170300	-
chr4	109500300	-
chr4	109513500	+
chr4	109574900	+
chr4	111305800	+
chr4	113442500	+
chr4	114824900	+
chr4	115043300	-
chr4	122306500	-
chr4	123718900	+
chr4	143615000	+
chr4	149191800	-
chr4	153155100	-
chr4	153257100	-
chr4	153396900	-
chr4	153422300	+
chr4	153694800	+
chr4	153784400	-
chr4	154256000	+
chr4	154638900	+
chr4	154792000	+
chr4	169669500	+
chr4	178551200	+
chr4	183967400	-
chr4	184589500	-
chr4	185440400	+
chr4	185468400	-
chr4	185579600	-
chr4	185635700	-
chr4	185662500	-
chr4	185672200	+
chr4	185827200	-
chr4	185921200	-
chr4	186141900	+
chr4	187362400	+
chr5	1016800	+
chr5	1369000	+
chr5	6413600	-
chr5	10688100	+
chr5	14613900	-
chr5	14979200	-
chr5	35839300	-
chr5	35847200	-
chr5	35858800	-
chr5	40419900	+
chr5	40477800	-
chr5	54087800	+
chr5	54426400	+
chr5	54433800	-
chr5	54772600	+
chr5	55344400	-
chr5	55387800	+
chr5	55426500	+
chr5	55474900	-
chr5	55659100	-
chr5	55920800	+
chr5	56001700	-
chr5	56031100	-
chr5	66520800	-
chr5	66535600	+
chr5	67757200	+
chr5	75657900	-
chr5	75663600	-
chr5	76181900	-
chr5	79456800	+
chr5	79524300	-
chr5	81688700	+
chr5	86351300	+
chr5	90643500	-
chr5	94154900	+
chr5	94993200	+
chr5	95170300	-
chr5	95205100	-
chr5	95899400	-
chr5	100192000	-
chr5	109221400	-
chr5	112293600	-
chr5	112382000	+
chr5	118667000	-
chr5	131364600	-
chr5	131383500	+
chr5	131446000	+
chr5	131467200	-
chr5	133417600	-
chr5	133443200	+
chr5	133454800	+
chr5	133468700	+
chr5	133800800	-
chr5	133867600	-
chr5	134689300	-
chr5	135400400	+
chr5	137059500	+
chr5	137823200	+
chr5	138316600	+
chr5	138344300	-
chr5	138778600	-
chr5	138818800	+
chr5	139702100	+
chr5	140963500	+
chr5	141318800	-
chr5	141792700	-
chr5	142158300	-
chr5	142205400	-
chr5	142362300	-
chr5	142390700	-
chr5	142582800	-
chr5	142590000	-
chr5	146111600	-
chr5	148658100	-
chr5	150594300	+
chr5	150974700	+
chr5	153557400	+
chr5	153632600	-
chr5	154190000	-
chr5	156518900	+
chr5	156904600	-
chr5	156962900	-
chr5	156973100	+
chr5	158701300	-
chr5	158786700	+
chr5	159865300	+
chr5	169662500	+
chr5	169671800	+
chr5	169693800	-
chr5	172137200	+
chr5	174880900	-
chr5	174999700	-
chr5	175012300	-
chr5	177598100	+
chr6	140400	+
chr6	180800	+
chr6	349300	+
chr6	419500	+
chr6	435600	+
chr6	2738700	+
chr6	3263300	-
chr6	5248200	-
chr6	5565000	-
chr6	5571600	+
chr6	5651400	+
chr6	5777200	-
chr6	6600400	-
chr6	7082300	+
chr6	7227200	+
chr6	11219500	+
chr6	11386000	+
chr6	11820200	+
chr6	12341500	+
chr6	14378500	+
chr6	14385200	+
chr6	14562100	-
chr6	14743600	+
chr6	14841900	-
chr6	16582700	-
chr6	16595100	-
chr6	20525000	-
chr6	25077700	-
chr6	25105500	-
chr6	25165000	-
chr6	29785500	-
chr6	30584300	+
chr6	30845300	-
chr6	31852600	+
chr6	33020300	-
chr6	33661200	+
chr6	33745100	-
chr6	33980700	+
chr6	33990100	+
chr6	34561200	-
chr6	35387500	+
chr6	35795600	+
chr6	35802600	-
chr6	36831600	-
chr6	36845400	-
chr6	37039400	-
chr6	37094300	+
chr6	37126500	+
chr6	37285100	+
chr6	37365900	-
chr6	40865000	-
chr6	41409500	+
chr6	42465800	+
chr6	43299500	+
chr6	43460500	+
chr6	43800800	+
chr6	43999400	+
chr6	45662400	+
chr6	45695600	+
chr6	52269800	-
chr6	52278400	-
chr6	90065500	+
chr6	90921300	-
chr6	90944100	+
chr6	91000600	-
chr6	91169200	-
chr6	91179800	+
chr6	106696800	+
chr6	106718500	+
chr6	107489700	-
chr6	108047300	-
chr6	109226900	-
chr6	109918900	+
chr6	110988900	-
chr6	111116900	+
chr6	112193400	+
chr6	112407300	+
chr6	112420800	+
chr6	119196500	+
chr6	119676800	+
chr6	119703900	-
chr6	128256100	+
chr6	130049900	+
chr6	130501000	+
chr6	130887600	-
chr6	130939200	-
chr6	137124800	-
chr6	137594600	+
chr6	138131600	-
chr6	138162800	-
chr6	138183400	-
chr6	138338800	-
chr6	139897800	-
chr6	139903000	+
chr6	143275900	+
chr6	144697500	-
chr6	144831300	+
chr6	146229000	+
chr6	147133300	+
chr6	149429100	-
chr6	149576200	-
chr6	149635000	-
chr6	154594200	+
chr6	154969500	+
chr6	156997500	+
chr6	157418000	-
chr6	157462600	+
chr6	157876500	+
chr6	159061400	+
chr6	159397700	+
chr6	159405900	+
chr6	160271400	-
chr6	160356700	+
chr6	163799300	-
chr6	166592500	+
chr6	166753000	-
chr7	1993700	-
chr7	2072300	+
chr7	2515300	-
chr7	2651200	+
chr7	2870300	-
chr7	3119300	-
chr7	3468100	-
chr7	6627400	+
chr7	7265100	-
chr7	8111700	+
chr7	8132300	-
chr7	12227900	+
chr7	21359100	-
chr7	24233800	-
chr7	24832800	+
chr7	25034700	+
chr7	25527000	-
chr7	26108000	-
chr7	29344100	-
chr7	36215200	+
chr7	36643700	-
chr7	36732700	+
chr7	36795900	-
chr7	37448100	-
chr7	38373800	-
chr7	39639900	+
chr7	44629400	-
chr7	44645500	+
chr7	44775400	+
chr7	44898000	-
chr7	45129200	+
chr7	50177300	+
chr7	50223200	+
chr7	50277200	-
chr7	55344600	-
chr7	69560000	+
chr7	73669500	+
chr7	75085100	+
chr7	76795000	+
chr7	90551300	-
chr7	92151300	+
chr7	92265400	-
chr7	95753000	-
chr7	104782300	-
chr7	105277300	-
chr7	105450300	-
chr7	105468800	-
chr7	111849200	+
chr7	114436200	-
chr7	127535900	+
chr7	128561800	+
chr7	129795900	+
chr7	131770200	+
chr7	131990300	+
chr7	134046600	-
chr7	138088000	+
chr7	138982900	-
chr7	139114100	+
chr7	139201300	+
chr7	139569900	+
chr7	139872700	-
chr7	142782700	+
chr7	144148400	+
chr7	148101700	-
chr7	149685800	-
chr7	150270300	-
chr7	150535700	-
chr7	150761100	+
chr7	151472400	+
chr7	151664200	+
chr7	156988200	+
chr7	157313100	-
chr7	157360500	+
chr8	1607400	+
chr8	2037600	-
chr8	2070100	-
chr8	2136900	-
chr8	8692900	-
chr8	8814900	+
chr8	11678400	-
chr8	11773100	-
chr8	19362700	-
chr8	19374200	-
chr8	19397900	+
chr8	19417100	+
chr8	20338600	+
chr8	20395500	-
chr8	20939600	+
chr8	21480500	-
chr8	21772000	-
chr8	21829000	+
chr8	22454000	-
chr8	22540200	-
chr8	22628800	+
chr8	22988500	-
chr8	25298600	+
chr8	28281500	-
chr8	28297700	+
chr8	28974200	+
chr8	29404300	-
chr8	33531700	-
chr8	37238700	+
chr8	41443400	-
chr8	41491400	+
chr8	50023800	+
chr8	53858500	-
chr8	57047700	+
chr8	57066800	-
chr8	59727000	+
chr8	61978700	+
chr8	62073700	-
chr8	62087900	-
chr8	67035100	+
chr8	67107400	-
chr8	67597700	+
chr8	67615700	-
chr8	67991800	-
chr8	68468400	+
chr8	71292100	-
chr8	71599300	-
chr8	91087100	-
chr8	95944400	-
chr8	96293700	+
chr8	100944000	-
chr8	101492600	+
chr8	101513000	-
chr8	101525000	-
chr8	101545200	-
chr8	101583600	+
chr8	102190100	+
chr8	102219200	-
chr8	103178400	+
chr8	103665800	-
chr8	103743800	+
chr8	105747200	+
chr8	121806000	+
chr8	121939300	-
chr8	125278800	+
chr8	125676400	+
chr8	125742100	-
chr8	126590300	-
chr8	126609900	-
chr8	126690000	-
chr8	127581700	-
chr8	129186100	+
chr8	129740300	+
chr8	131145800	-
chr8	134063400	-
chr8	134163000	+
chr8	134205000	+
chr8	134299200	-
chr8	134698900	-
chr8	141352400	+
chr8	141870900	-
chr8	142318200	+
chr9	5342400	+
chr9	5548200	-
chr9	5576400	+
chr9	5834000	+
chr9	7140100	+
chr9	7926300	-
chr9	7967100	-
chr9	20199100	+
chr9	20987000	+
chr9	33071700	-
chr9	33407000	+
chr9	33443100	-
chr9	33449200	-
chr9	34652700	+
chr9	35898100	+
chr9	35935200	+
chr9	36141400	+
chr9	38268700	+
chr9	70700200	-
chr9	72368900	-
chr9	76956700	+
chr9	79782300	+
chr9	79815400	+
chr9	79879200	+
chr9	80016300	-
chr9	81429300	-
chr9	81689200	+
chr9	86098100	-
chr9	89490400	+
chr9	89514200	+
chr9	89597600	+
chr9	90728000	+
chr9	91313400	+
chr9	91324000	-
chr9	91333000	+
chr9	91585100	-
chr9	92644000	-
chr9	92965600	-
chr9	94946000	+
chr9	95700100	+
chr9	96672900	+
chr9	96754100	+
chr9	99906900	-
chr9	100816000	-
chr9	100860700	-
chr9	103375100	+
chr9	106916900	-
chr9	106930200	-
chr9	110903000	+
chr9	111770900	-
chr9	111928500	-
chr9	111959600	-
chr9	115350600	+
chr9	116254100	-
chr9	116609000	-
chr9	116696900	+
chr9	116709800	-
chr9	116792000	-
chr9	122705700	-
chr9	122737600	+
chr9	125107300	-
chr9	126005200	-
chr9	126018700	+
chr9	126088300	+
chr9	126297600	+
chr9	127449200	-
chr9	128213800	+
chr9	128272200	-
chr9	129360100	-
chr9	129664200	+
chr9	130871200	+
chr9	131010400	+
chr9	131260600	-
chr9	131386300	+
chr9	131671200	+
chr9	132042400	+
chr9	133118500	+
chr9	133175200	+
chr9	133609200	-
chr9	135513500	+
chr9	139402000	-
chrX	10011500	-
chrX	17701300	+
chrX	37499000	+
chrX	39053700	+
chrX	39599400	-
chrX	49001700	-
chrX	56841500	-
chrX	118593200	+
chrX	128566400	-
chrX	128722300	-
chrX	128734700	+
chrX	135530600	-
chrX	153600500	+

Cluster GW11 (in hg18)
chr1	3685700	-
chr1	3732300	-
chr1	6515300	+
chr1	6634800	+
chr1	11051200	+
chr1	11064700	-
chr1	11112600	-
chr1	11129600	+
chr1	11139100	-
chr1	11151800	-
chr1	11187500	+
chr1	11195600	-
chr1	11235800	+
chr1	11799300	-
chr1	11979300	+
chr1	11987700	+
chr1	12234100	-
chr1	12259600	-
chr1	12266600	-
chr1	12315600	+
chr1	15765600	-
chr1	15860400	-
chr1	16129100	+
chr1	19109300	+
chr1	19292600	+
chr1	19305400	-
chr1	19344500	+
chr1	19350900	+
chr1	19359800	-
chr1	19372800	+
chr1	19382200	+
chr1	19397600	-
chr1	19442100	+
chr1	19943100	-
chr1	19949800	+
chr1	19969500	-
chr1	19978600	-
chr1	19984000	-
chr1	20092000	-
chr1	20112600	+
chr1	20699900	-
chr1	21886000	-
chr1	21892000	+
chr1	21913300	+
chr1	22713800	+
chr1	22726200	-
chr1	23266500	-
chr1	23278100	+
chr1	23286100	+
chr1	24016200	-
chr1	24644100	-
chr1	24658700	-
chr1	25658800	+
chr1	25691000	-
chr1	26028500	+
chr1	26161300	+
chr1	26182300	+
chr1	26643200	-
chr1	26664600	+
chr1	27497100	+
chr1	27612700	-
chr1	31503200	-
chr1	31564400	-
chr1	31610200	+
chr1	31866200	-
chr1	32333600	+
chr1	32399600	-
chr1	32408800	+
chr1	32605300	-
chr1	32616400	+
chr1	33100000	+
chr1	33176900	+
chr1	33246400	+
chr1	33252700	-
chr1	33257800	-
chr1	33395800	-
chr1	33565600	-
chr1	35678500	+
chr1	35687700	-
chr1	35828700	-
chr1	36132400	-
chr1	36375600	-
chr1	37804800	+
chr1	37810700	+
chr1	39530600	+
chr1	39548400	+
chr1	39556000	+
chr1	39571200	+
chr1	39578400	-
chr1	39680300	+
chr1	39692000	+
chr1	39707700	+
chr1	39719500	-
chr1	39767100	+
chr1	39786400	+
chr1	40993200	-
chr1	41007600	-
chr1	41245400	+
chr1	41265300	+
chr1	42904900	+
chr1	42913200	-
chr1	42990800	+
chr1	43644400	-
chr1	43670200	-
chr1	43678500	+
chr1	43684700	+
chr1	43921400	+
chr1	44054400	-
chr1	44458300	-
chr1	44482600	+
chr1	44491100	-
chr1	44535800	-
chr1	44545000	+
chr1	44551900	+
chr1	44566600	+
chr1	44575800	-
chr1	44885900	+
chr1	45584900	+
chr1	45855100	-
chr1	46600400	+
chr1	46828200	+
chr1	46895000	-
chr1	51019500	+
chr1	51610500	+
chr1	52652900	+
chr1	52887900	-
chr1	52906300	-
chr1	53009100	-
chr1	53152600	+
chr1	53287200	+
chr1	53448700	-
chr1	53500300	+
chr1	54116000	-
chr1	54416200	+
chr1	54450600	-
chr1	54978400	+
chr1	55105200	-
chr1	55304800	-
chr1	55323600	-
chr1	55345900	-
chr1	55359200	+
chr1	55374900	+
chr1	63881500	-
chr1	65074700	+
chr1	67278900	+
chr1	74954400	+
chr1	74990700	-
chr1	78194500	-
chr1	85893300	+
chr1	89206600	-
chr1	89244600	-
chr1	89253200	-
chr1	89296700	-
chr1	89345500	-
chr1	89351600	+
chr1	89797600	-
chr1	89820900	+
chr1	89951800	+
chr1	91976800	-
chr1	91987300	-
chr1	93139900	-
chr1	96991100	-
chr1	97471700	+
chr1	97543000	+
chr1	97876200	-
chr1	100310700	+
chr1	100726900	+
chr1	101204400	+
chr1	109580200	+
chr1	110362600	-
chr1	110401100	+
chr1	111803000	+
chr1	113447400	-
chr1	114240200	-
chr1	114316800	+
chr1	116752300	-
chr1	117421700	-
chr1	117761100	+
chr1	118283600	-
chr1	119504100	+
chr1	120259400	-
chr1	120266900	-
chr1	120298500	+
chr1	120310500	+
chr1	144312200	-
chr1	145597600	+
chr1	148710000	+
chr1	149189700	-
chr1	149414300	+
chr1	149645000	+
chr1	151990600	+
chr1	152007500	+
chr1	152494200	-
chr1	152500300	-
chr1	152825000	+
chr1	153165100	-
chr1	154489800	-
chr1	154496900	-
chr1	154504300	-
chr1	154510800	-
chr1	155032600	-
chr1	155182200	-
chr1	155193000	-
chr1	157212600	-
chr1	157229200	-
chr1	157291200	+
chr1	158454800	+
chr1	158460900	-
chr1	158543600	+
chr1	158549200	+
chr1	158592000	+
chr1	158726200	+
chr1	158783900	+
chr1	158790000	-
chr1	158846900	-
chr1	159059700	-
chr1	160757800	+
chr1	165292000	-
chr1	168088500	+
chr1	168096700	+
chr1	168104800	-
chr1	168787600	-
chr1	169794100	-
chr1	169818300	+
chr1	169827200	-
chr1	170031700	+
chr1	170825800	-
chr1	171970100	+
chr1	171993500	+
chr1	177329800	+
chr1	177345600	-
chr1	178279100	+
chr1	179226800	-
chr1	181122000	-
chr1	181787800	+
chr1	182942500	+
chr1	183535000	-
chr1	191317900	-
chr1	195424200	-
chr1	196519100	-
chr1	196953200	-
chr1	196970000	+
chr1	199372800	+
chr1	199379000	-
chr1	200225100	+
chr1	200239400	+
chr1	200964800	-
chr1	201935800	+
chr1	201947300	+
chr1	201956600	+
chr1	202009700	-
chr1	202670600	-
chr1	202679300	-
chr1	202686000	+
chr1	203939900	+
chr1	208082900	+
chr1	208094500	-
chr1	209544800	-
chr1	209614900	-
chr1	210199700	+
chr1	210626600	-
chr1	212571000	-
chr1	218417400	+
chr1	218492400	-
chr1	220869400	-
chr1	220902200	+
chr1	220919500	+
chr1	222006700	+
chr1	222025900	-
chr1	224407500	+
chr1	224612600	-
chr1	224622400	-
chr1	226547800	-
chr1	226594100	+
chr1	227501700	+
chr1	227666200	-
chr1	227672500	-
chr1	227681900	+
chr1	227796800	-
chr1	227803300	-
chr1	227814300	-
chr1	227851600	-
chr1	228467100	-
chr1	228868300	+
chr1	228874900	-
chr1	229140400	-
chr1	229147900	-
chr1	229202800	-
chr1	229428600	-
chr1	229459800	-
chr1	229974400	-
chr1	230078500	+
chr1	231221400	+
chr1	231255000	+
chr1	231452800	+
chr1	231460900	-
chr1	233348600	+
chr1	233366400	+
chr1	234436900	+
chr1	234769000	+
chr1	234778200	-
chr1	234795400	+
chr1	234802200	-
chr1	235060700	-
chr1	235067800	-
chr1	235074700	-
chr1	235084200	+
chr1	235092600	+
chr1	235125600	-
chr1	241740700	-
chr1	242642900	+
chr1	242928300	+
chr1	244877900	+
chr1	245103700	-
chr1	245126600	+
chr1	245538800	+
chr1	245655600	+
chr1	247178200	-
chr10	1143800	+
chr10	3137100	-
chr10	3150300	-
chr10	3157300	+
chr10	3165000	+
chr10	3171700	-
chr10	3186800	-
chr10	3195100	+
chr10	5829100	+
chr10	5841700	+
chr10	5846900	+
chr10	5867300	+
chr10	5998300	+
chr10	6018800	+
chr10	6044200	+
chr10	6196100	-
chr10	6690300	-
chr10	7266500	-
chr10	7856600	-
chr10	8046300	-
chr10	12003600	-
chr10	12059900	+
chr10	12851600	-
chr10	12897600	-
chr10	12905100	+
chr10	13214000	+
chr10	13223400	+
chr10	13691700	-
chr10	13701900	+
chr10	13720300	-
chr10	15010000	-
chr10	15201300	+
chr10	15206600	-
chr10	16681400	-
chr10	16803400	-
chr10	16809200	+
chr10	16839500	+
chr10	17796100	-
chr10	22866400	+
chr10	26841400	-
chr10	26873100	-
chr10	26893900	+
chr10	27064600	-
chr10	27401600	-
chr10	28405500	+
chr10	29750400	+
chr10	29793700	-
chr10	32818100	-
chr10	35872300	+
chr10	35889400	+
chr10	38356700	+
chr10	42613000	+
chr10	45275100	+
chr10	45303600	+
chr10	45442500	-
chr10	50351000	-
chr10	51740200	-
chr10	51773300	-
chr10	52021500	+
chr10	61234200	+
chr10	61241800	+
chr10	61247400	-
chr10	61256100	-
chr10	61505900	+
chr10	61602800	+
chr10	61626200	+
chr10	61692900	-
chr10	69373800	+
chr10	69407400	+
chr10	70188600	+
chr10	70393000	-
chr10	70534800	-
chr10	71767000	-
chr10	75528500	-
chr10	75534300	-
chr10	76405600	-
chr10	76459900	-
chr10	79416700	-
chr10	79435000	+
chr10	88200200	+
chr10	88803500	+
chr10	90664600	-
chr10	90989500	+
chr10	93005000	-
chr10	93026700	+
chr10	93733500	+
chr10	97366900	+
chr10	97395400	+
chr10	97807400	+
chr10	98283100	+
chr10	98705400	+
chr10	98731400	+
chr10	99139800	-
chr10	99219800	+
chr10	99226600	+
chr10	99506600	-
chr10	99958800	-
chr10	99985200	-
chr10	99999700	-
chr10	101169700	+
chr10	101359500	+
chr10	101467800	-
chr10	101901200	-
chr10	102239900	-
chr10	102246600	+
chr10	103359300	+
chr10	104108500	+
chr10	104129800	+
chr10	104349900	+
chr10	104565000	-
chr10	104832300	-
chr10	105155700	-
chr10	105174100	-
chr10	105646700	-
chr10	105769600	-
chr10	111621700	-
chr10	111631000	+
chr10	111636600	+
chr10	111656500	+
chr10	111868300	-
chr10	114289100	-
chr10	114465900	+
chr10	115478800	+
chr10	116180500	-
chr10	116188400	+
chr10	116212700	-
chr10	116593500	+
chr10	116712800	+
chr10	119034800	-
chr10	119782000	+
chr10	120083700	-
chr10	120435100	+
chr10	121591400	+
chr10	121602600	-
chr10	121671000	+
chr10	121691600	+
chr10	123530100	-
chr10	126168100	-
chr10	126645000	-
chr10	126662000	-
chr10	127408200	+
chr10	127415100	+
chr10	127423700	-
chr10	127431100	-
chr10	127441500	+
chr10	127473100	-
chr10	127486800	-
chr10	129767200	+
chr10	131763800	-
chr10	131872600	+
chr10	133611900	-
chr11	673000	+
chr11	3654000	+
chr11	3700200	-
chr11	4061200	+
chr11	5657300	-
chr11	6434400	-
chr11	6541300	+
chr11	7610100	-
chr11	10742600	+
chr11	13342400	-
chr11	13348300	+
chr11	13358100	+
chr11	13379900	+
chr11	14458400	-
chr11	17807600	-
chr11	17839700	+
chr11	17853100	+
chr11	17872400	-
chr11	17905200	+
chr11	17928200	+
chr11	17932400	+
chr11	17943400	-
chr11	17951600	+
chr11	17963000	+
chr11	17983500	+
chr11	18073800	-
chr11	18459900	-
chr11	18469200	+
chr11	20416600	-
chr11	33687700	-
chr11	34090000	-
chr11	34116000	+
chr11	34125600	-
chr11	35200900	+
chr11	36395000	-
chr11	36468800	-
chr11	43385000	+
chr11	43876900	-
chr11	43897400	+
chr11	44092200	-
chr11	44204700	-
chr11	44848600	-
chr11	45183200	+
chr11	45841400	-
chr11	45846400	+
chr11	45859500	+
chr11	45926100	+
chr11	46411600	+
chr11	46520500	+
chr11	47267300	+
chr11	47302500	+
chr11	47448700	-
chr11	47456900	-
chr11	47813000	-
chr11	48107300	-
chr11	48115000	+
chr11	48122300	-
chr11	56941400	-
chr11	57076600	+
chr11	57213300	+
chr11	58141500	-
chr11	59100600	+
chr11	59118100	-
chr11	59124000	+
chr11	60375800	+
chr11	60827300	-
chr11	60833700	-
chr11	60847100	-
chr11	60928200	+
chr11	62347600	+
chr11	63069600	+
chr11	64730100	-
chr11	64819200	-
chr11	65744200	+
chr11	66016800	+
chr11	67687900	-
chr11	67694600	+
chr11	67784400	+
chr11	68101200	+
chr11	68115900	-
chr11	68125600	+
chr11	68139800	-
chr11	68259500	+
chr11	68297700	+
chr11	69862000	-
chr11	69901900	-
chr11	70885500	-
chr11	71695700	-
chr11	71718900	-
chr11	72400400	+
chr11	72808800	+
chr11	73641900	-
chr11	74001400	+
chr11	74206900	+
chr11	74226100	-
chr11	74233700	-
chr11	74241200	-
chr11	74296400	+
chr11	74324700	-
chr11	74369500	+
chr11	74394800	+
chr11	74401700	+
chr11	75372400	-
chr11	75395200	+
chr11	76335200	+
chr11	77350100	-
chr11	77458700	-
chr11	85651000	+
chr11	85727100	-
chr11	87665900	+
chr11	93181900	-
chr11	93550800	-
chr11	94337700	-
chr11	94481900	+
chr11	95352900	+
chr11	101705700	-
chr11	104329600	+
chr11	107545600	-
chr11	107731200	-
chr11	108052200	+
chr11	111096000	-
chr11	111114400	-
chr11	111123400	-
chr11	112720400	+
chr11	112727500	-
chr11	112734800	+
chr11	116139000	+
chr11	116222500	-
chr11	116251000	+
chr11	116664800	+
chr11	116720900	-
chr11	116731700	-
chr11	116738400	+
chr11	116757100	+
chr11	117608300	-
chr11	117898100	+
chr11	118453300	+
chr11	120683100	+
chr11	120965400	+
chr11	120985400	+
chr11	120997000	-
chr11	122165000	-
chr11	122172700	-
chr11	122180100	-
chr11	122989900	-
chr11	124005900	+
chr11	124476200	+
chr11	124953600	+
chr11	125573100	+
chr11	125668500	+
chr11	129254400	+
chr11	129498500	-
chr11	129636100	-
chr11	130258100	-
chr11	130270100	+
chr11	130278900	-
chr11	133543700	+
chr11	133617400	+
chr11	133634700	+
chr12	313100	-
chr12	1945800	+
chr12	2802800	+
chr12	3594100	-
chr12	3606200	-
chr12	3635700	+
chr12	3647500	+
chr12	3658700	-
chr12	3694000	-
chr12	4639700	+
chr12	4650200	-
chr12	4664500	-
chr12	6496600	-
chr12	6561500	+
chr12	6652700	+
chr12	7180300	-
chr12	8092400	+
chr12	8987400	-
chr12	10264700	+
chr12	11886500	+
chr12	11904900	+
chr12	11929400	+
chr12	12681300	-
chr12	12868300	+
chr12	14504100	-
chr12	14527900	-
chr12	14534600	-
chr12	21560000	-
chr12	21679200	-
chr12	21685900	+
chr12	22577800	+
chr12	22723800	-
chr12	25113400	+
chr12	27835700	-
chr12	30675600	+
chr12	38238500	+
chr12	41065700	-
chr12	43994900	-
chr12	44031000	-
chr12	44049600	-
chr12	44531900	-
chr12	44555800	+
chr12	44905200	-
chr12	46823600	+
chr12	46830200	+
chr12	47023600	-
chr12	47335000	+
chr12	47621100	+
chr12	48779100	+
chr12	48809600	+
chr12	49388000	-
chr12	49675400	+
chr12	49739600	-
chr12	49870800	+
chr12	50663300	-
chr12	51995100	-
chr12	52395500	-
chr12	53205600	+
chr12	53220100	-
chr12	54671600	+
chr12	54893100	+
chr12	55290500	+
chr12	55393700	+
chr12	55779400	+
chr12	55949200	-
chr12	56216200	+
chr12	61040400	-
chr12	63401400	+
chr12	65986000	-
chr12	68254700	+
chr12	69019200	-
chr12	75292200	-
chr12	81392300	-
chr12	93214400	-
chr12	93889700	+
chr12	93980400	-
chr12	94945900	-
chr12	95190300	-
chr12	100938800	+
chr12	101025500	-
chr12	103239000	-
chr12	104123700	+
chr12	105260200	-
chr12	106504000	-
chr12	106537200	-
chr12	106577600	-
chr12	106609100	-
chr12	106621900	-
chr12	106629200	+
chr12	106656500	-
chr12	107454000	+
chr12	107733300	+
chr12	108370200	+
chr12	108408900	-
chr12	108430100	+
chr12	108443300	+
chr12	108829500	+
chr12	108836600	+
chr12	108852900	+
chr12	108874300	-
chr12	109249200	-
chr12	109297500	-
chr12	109641000	+
chr12	110377300	-
chr12	110577800	-
chr12	110586900	+
chr12	110615600	+
chr12	110811300	+
chr12	110822300	-
chr12	110974800	-
chr12	111075600	-
chr12	111219100	+
chr12	111405100	+
chr12	111411200	-
chr12	111840800	-
chr12	111884600	-
chr12	112093000	+
chr12	112099700	+
chr12	112199800	-
chr12	112294000	+
chr12	112751400	-
chr12	112762400	-
chr12	112772500	-
chr12	112780600	-
chr12	112786500	-
chr12	112801200	-
chr12	112826100	+
chr12	112832500	-
chr12	112839700	+
chr12	112869300	-
chr12	114895600	-
chr12	114905000	-
chr12	114913100	-
chr12	114931500	+
chr12	115869300	-
chr12	115938400	-
chr12	116097800	-
chr12	117073800	-
chr12	117082700	+
chr12	117094500	-
chr12	117122000	+
chr12	118601600	+
chr12	118988100	+
chr12	118994300	+
chr12	119059500	+
chr12	119069800	-
chr12	119076100	+
chr12	119385600	+
chr12	119487400	-
chr12	119688800	+
chr12	119933500	-
chr12	120152000	+
chr12	120250200	-
chr12	120345900	-
chr12	120825300	+
chr12	121385600	+
chr12	122454500	-
chr12	122664900	-
chr12	122784700	+
chr12	123063800	-
chr12	123398800	-
chr12	123409100	+
chr12	127848400	+
chr12	129851300	+
chr12	129856900	+
chr12	130769700	+
chr12	130807700	-
chr12	131030900	+
chr12	131057200	-
chr12	131128600	-
chr12	131177300	+
chr12	131718600	-
chr12	131762800	+
chr12	131827200	+
chr12	131867300	+
chr13	19478000	-
chr13	20280900	-
chr13	22838800	+
chr13	23926200	-
chr13	23965100	-
chr13	25686500	+
chr13	27041300	-
chr13	27051400	+
chr13	28988900	-
chr13	28996000	+
chr13	29001100	-
chr13	30121000	-
chr13	30230000	+
chr13	36436900	+
chr13	36463600	+
chr13	38486000	-
chr13	41083000	+
chr13	41602200	-
chr13	41781800	-
chr13	42369000	-
chr13	42418600	+
chr13	42425800	+
chr13	44475300	+
chr13	44610000	+
chr13	44880300	-
chr13	44975800	-
chr13	44990800	-
chr13	45007100	-
chr13	45440600	+
chr13	45622400	+
chr13	45627000	+
chr13	47545500	-
chr13	48604200	+
chr13	48670200	-
chr13	48958600	-
chr13	48987100	+
chr13	49032400	-
chr13	50448700	-
chr13	51214100	+
chr13	72188300	-
chr13	72210000	+
chr13	72266000	+
chr13	73167400	+
chr13	73208000	+
chr13	76551800	-
chr13	76616400	+
chr13	76645500	+
chr13	76744000	+
chr13	76752000	-
chr13	78839000	-
chr13	78849300	+
chr13	94023000	-
chr13	95231100	+
chr13	96915100	-
chr13	97443100	-
chr13	97469600	-
chr13	98247600	+
chr13	98252600	+
chr13	98280600	+
chr13	98296500	-
chr13	98309900	+
chr13	98332300	-
chr13	98352000	+
chr13	98373000	+
chr13	98996200	-
chr13	100093300	+
chr13	100113100	+
chr13	102077600	-
chr13	102096200	-
chr13	110729200	+
chr13	110743400	+
chr13	110754200	-
chr13	112200500	+
chr13	112249200	-
chr13	112256200	-
chr13	112519900	-
chr13	112527600	-
chr13	112534300	+
chr13	112881400	+
chr13	112899400	-
chr13	112948500	+
chr13	112958100	+
chr13	113160900	+
chr13	113202300	+
chr13	113239900	-
chr13	114025600	+
chr14	19836900	+
chr14	19910800	-
chr14	19918800	+
chr14	19945300	+
chr14	19986000	+
chr14	20932400	-
chr14	20937900	+
chr14	20952900	+
chr14	22150200	+
chr14	22438000	+
chr14	22444700	-
chr14	22618300	+
chr14	23643000	+
chr14	23662400	-
chr14	23793900	+
chr14	30265800	+
chr14	30667800	+
chr14	35144400	-
chr14	38579300	-
chr14	38624500	+
chr14	38937800	-
chr14	50274200	-
chr14	50298100	-
chr14	51484000	-
chr14	54377100	-
chr14	54396200	+
chr14	54888300	-
chr14	55157000	-
chr14	56174600	-
chr14	59663300	-
chr14	63547600	-
chr14	63596800	+
chr14	63602900	+
chr14	63635200	-
chr14	63683200	-
chr14	63707000	-
chr14	63727100	+
chr14	63754600	+
chr14	64549800	-
chr14	66914200	+
chr14	67186700	-
chr14	67341200	+
chr14	68408400	+
chr14	68627500	-
chr14	68912600	-
chr14	70514500	+
chr14	70567800	+
chr14	70581100	+
chr14	70597200	+
chr14	70628400	+
chr14	71221100	+
chr14	72479900	-
chr14	72511200	+
chr14	72743700	-
chr14	72756300	-
chr14	72819900	-
chr14	73445700	-
chr14	74199600	-
chr14	74317800	+
chr14	74429900	-
chr14	74437500	-
chr14	74620200	-
chr14	75187700	-
chr14	75719900	+
chr14	75733100	+
chr14	76650100	+
chr14	76878700	+
chr14	76978700	-
chr14	77001000	-
chr14	77355800	+
chr14	89504200	-
chr14	89533200	+
chr14	89540100	-
chr14	89561500	-
chr14	89577400	-
chr14	89809000	+
chr14	89828200	-
chr14	89839900	-
chr14	90739300	+
chr14	91523700	-
chr14	92354900	-
chr14	92498500	+
chr14	92787700	+
chr14	93582100	+
chr14	94649800	-
chr14	94661500	-
chr14	94957900	-
chr14	94966800	-
chr14	94972900	+
chr14	98935600	-
chr14	98945300	+
chr14	98964700	-
chr14	98983800	-
chr14	99890300	-
chr14	99896500	+
chr14	100027300	-
chr14	100063700	+
chr14	101438500	-
chr14	101516000	+
chr14	101530200	-
chr14	101541100	-
chr14	101577900	-
chr14	102414100	-
chr14	103198200	+
chr15	20401800	-
chr15	20410200	-
chr15	20558000	-
chr15	23480100	-
chr15	23488000	-
chr15	23518100	-
chr15	26186500	+
chr15	27188400	+
chr15	29003300	-
chr15	29033100	-
chr15	29038000	+
chr15	32435800	+
chr15	36579900	-
chr15	36591600	+
chr15	38047700	-
chr15	39057400	+
chr15	39606800	+
chr15	39897700	-
chr15	40003100	+
chr15	40240600	-
chr15	40246300	+
chr15	40266100	+
chr15	40316900	+
chr15	40530500	-
chr15	40804700	+
chr15	40832300	-
chr15	41558100	+
chr15	42645300	+
chr15	43755100	-
chr15	43761600	+
chr15	43771000	+
chr15	47189800	-
chr15	47363900	-
chr15	47369000	-
chr15	47383700	+
chr15	49878700	+
chr15	49949200	-
chr15	50030500	+
chr15	50139500	+
chr15	50430700	-
chr15	50689800	+
chr15	54175500	-
chr15	55342500	+
chr15	55353700	+
chr15	56967300	+
chr15	58426800	-
chr15	58433900	+
chr15	60087500	+
chr15	61140500	-
chr15	61640900	+
chr15	61708800	-
chr15	61724500	-
chr15	61743300	-
chr15	61792300	-
chr15	61825100	-
chr15	62216300	+
chr15	62251500	-
chr15	63178300	+
chr15	63198500	+
chr15	63344100	-
chr15	63638000	-
chr15	63770000	-
chr15	63794900	+
chr15	64388000	+
chr15	64561000	+
chr15	64569000	-
chr15	65272500	+
chr15	66263800	-
chr15	66299100	-
chr15	70321100	+
chr15	70333300	+
chr15	70429100	+
chr15	70816000	-
chr15	70835400	-
chr15	71648900	-
chr15	71662400	-
chr15	71997700	+
chr15	72121400	+
chr15	72530200	+
chr15	72716100	-
chr15	72722700	+
chr15	72975900	+
chr15	73459400	+
chr15	73468800	-
chr15	73548600	-
chr15	73697100	+
chr15	74013000	-
chr15	74294300	+
chr15	74340300	-
chr15	76251200	-
chr15	76354200	+
chr15	76359600	+
chr15	76628900	+
chr15	79020500	-
chr15	79027300	-
chr15	80242200	+
chr15	83482500	+
chr15	86874600	+
chr15	87500200	+
chr15	87507600	+
chr15	87512100	+
chr15	87519600	-
chr15	87535500	-
chr15	88180100	+
chr15	88793200	-
chr15	88799600	-
chr15	88818200	-
chr15	88973700	+
chr15	89262000	-
chr15	89351300	-
chr15	89357900	-
chr15	90469600	-
chr15	90480000	-
chr15	90493800	+
chr15	91326300	+
chr15	91341900	-
chr15	91356400	-
chr15	91365200	+
chr15	97272500	+
chr15	100003900	-
chr15	100031500	+
chr15	100058300	-
chr16	3732900	+
chr16	3747900	-
chr16	3764800	-
chr16	4431900	+
chr16	4849600	+
chr16	4864800	-
chr16	4870700	-
chr16	8643700	-
chr16	8813200	+
chr16	8907300	+
chr16	10762200	+
chr16	11062400	-
chr16	11125500	+
chr16	11551400	+
chr16	11694100	-
chr16	11775300	-
chr16	12026100	+
chr16	12290600	+
chr16	12346700	+
chr16	12405000	+
chr16	12445500	+
chr16	14255200	-
chr16	14583800	+
chr16	14668600	-
chr16	15035200	+
chr16	15618100	+
chr16	15626600	+
chr16	15726600	-
chr16	16084500	-
chr16	16113500	+
chr16	16126300	-
chr16	16138900	+
chr16	17118400	+
chr16	17127500	+
chr16	17138300	+
chr16	17161200	-
chr16	17222600	-
chr16	18761100	+
chr16	19572400	+
chr16	19603700	-
chr16	19624100	-
chr16	20779600	+
chr16	21883400	+
chr16	22176300	+
chr16	22268700	-
chr16	23398200	+
chr16	23448400	-
chr16	23453700	+
chr16	23586300	+
chr16	23607000	+
chr16	24012400	+
chr16	24042300	-
chr16	24092500	+
chr16	24097600	-
chr16	24104700	+
chr16	24115400	+
chr16	24708800	+
chr16	24718900	-
chr16	24840000	-
chr16	24858800	+
chr16	24874300	+
chr16	25063400	+
chr16	27175300	+
chr16	27270300	+
chr16	27389300	+
chr16	27401100	+
chr16	27435400	+
chr16	27449800	+
chr16	27501500	+
chr16	28788700	+
chr16	28882700	-
chr16	29717800	-
chr16	30629900	+
chr16	46235500	-
chr16	46854100	+
chr16	46933900	+
chr16	48819400	+
chr16	48907700	+
chr16	52061900	+
chr16	52087900	-
chr16	52406000	+
chr16	52418400	+
chr16	54959100	+
chr16	54975600	+
chr16	55675200	-
chr16	55796600	+
chr16	56049000	-
chr16	56060900	+
chr16	56607800	+
chr16	56706200	+
chr16	57129100	+
chr16	65322600	-
chr16	65726300	+
chr16	65732300	+
chr16	66322900	-
chr16	66938200	-
chr16	67625700	+
chr16	67734500	+
chr16	67959000	-
chr16	68390800	-
chr16	68436100	-
chr16	68465200	+
chr16	68501400	+
chr16	68517200	-
chr16	68750700	-
chr16	68850000	+
chr16	69088400	-
chr16	69140100	+
chr16	69146700	+
chr16	70068000	+
chr16	70350100	+
chr16	70605600	+
chr16	70700500	+
chr16	73047600	+
chr16	73061000	+
chr16	73081600	-
chr16	73267000	+
chr16	73283300	-
chr16	73533800	+
chr16	74204600	+
chr16	74226800	-
chr16	74248800	-
chr16	79220200	+
chr16	79968900	+
chr16	80246300	-
chr16	80255800	-
chr16	80590400	-
chr16	82659800	-
chr16	83369200	-
chr16	83567100	+
chr16	84255600	-
chr16	85129800	-
chr16	86665200	-
chr17	386200	-
chr17	410100	+
chr17	450600	+
chr17	610000	+
chr17	918000	-
chr17	1637300	-
chr17	1747000	+
chr17	1931800	+
chr17	2870100	-
chr17	3866800	-
chr17	3882600	+
chr17	3907200	-
chr17	3925000	+
chr17	3938500	-
chr17	3954900	+
chr17	4006400	+
chr17	4012000	+
chr17	4057400	-
chr17	4526300	-
chr17	4848600	-
chr17	5206300	-
chr17	5235800	-
chr17	5249800	+
chr17	5321900	+
chr17	5376100	-
chr17	7355100	-
chr17	7748600	+
chr17	7788400	+
chr17	8109100	+
chr17	8325400	-
chr17	9110100	+
chr17	10555400	+
chr17	11827200	-
chr17	11960400	-
chr17	11972700	-
chr17	12836100	+
chr17	13878300	+
chr17	15298800	+
chr17	15360800	+
chr17	15870900	-
chr17	15907000	-
chr17	15941200	+
chr17	17721600	+
chr17	18125300	+
chr17	18134800	+
chr17	18144400	+
chr17	18721300	-
chr17	19499400	+
chr17	19509300	+
chr17	19515600	+
chr17	19749500	-
chr17	20851800	+
chr17	21017800	+
chr17	21066100	+
chr17	22943300	+
chr17	23702300	-
chr17	23907200	-
chr17	23936400	-
chr17	23970800	+
chr17	24026800	-
chr17	24037600	-
chr17	24051200	+
chr17	24110100	-
chr17	24262000	+
chr17	24269400	-
chr17	24443900	+
chr17	24894600	+
chr17	25023200	-
chr17	25600200	-
chr17	25610500	+
chr17	26882600	-
chr17	30343900	+
chr17	30365100	+
chr17	30457600	-
chr17	30485300	+
chr17	31068300	-
chr17	31076400	-
chr17	31944800	-
chr17	31954500	+
chr17	32482200	+
chr17	32487600	-
chr17	32975100	+
chr17	34188600	-
chr17	34819400	-
chr17	34934900	+
chr17	34967500	-
chr17	35441800	+
chr17	35580900	+
chr17	35689000	+
chr17	37295500	-
chr17	37315100	+
chr17	37381900	-
chr17	37394800	-
chr17	38123800	-
chr17	38223900	+
chr17	38249300	+
chr17	38361100	-
chr17	38926300	-
chr17	38940400	+
chr17	38957200	+
chr17	40287100	+
chr17	40297500	-
chr17	40535700	+
chr17	41063600	+
chr17	41466800	+
chr17	41472300	-
chr17	42188300	+
chr17	43089600	-
chr17	43242500	+
chr17	43586200	-
chr17	43610500	-
chr17	43617600	+
chr17	44295700	+
chr17	45030100	-
chr17	45053700	+
chr17	45254000	+
chr17	45815400	-
chr17	46129500	-
chr17	46711200	+
chr17	46719700	+
chr17	52323600	+
chr17	52550700	-
chr17	52999100	-
chr17	53052400	+
chr17	53062300	-
chr17	53936800	-
chr17	54830700	-
chr17	57414200	-
chr17	57462600	-
chr17	57476100	+
chr17	58018200	-
chr17	58042400	-
chr17	59020700	+
chr17	59111100	+
chr17	62074100	-
chr17	62134300	+
chr17	62169300	+
chr17	62180500	+
chr17	62218600	-
chr17	62534500	+
chr17	63177400	-
chr17	63371500	+
chr17	64044900	-
chr17	65013300	-
chr17	68336300	+
chr17	68538900	+
chr17	68579500	+
chr17	70733400	+
chr17	71208600	+
chr17	71320800	+
chr17	71548300	+
chr17	72704500	+
chr17	72713900	-
chr17	73581200	+
chr17	73600600	-
chr17	73605900	-
chr17	74321100	-
chr17	75726400	+
chr17	76496400	-
chr17	76513200	-
chr17	76517900	+
chr17	76535100	+
chr17	77143400	+
chr17	77202900	+
chr18	2729200	-
chr18	2764900	+
chr18	8626100	-
chr18	9524800	-
chr18	9540100	+
chr18	9548900	-
chr18	10530300	+
chr18	10542300	+
chr18	11874500	-
chr18	11892400	-
chr18	12324600	+
chr18	13004300	-
chr18	13032900	+
chr18	13046900	+
chr18	13086500	-
chr18	13096000	+
chr18	13481900	+
chr18	13735700	+
chr18	13746400	-
chr18	13752400	+
chr18	19369800	-
chr18	19389600	+
chr18	21891100	+
chr18	30973800	+
chr18	31168200	-
chr18	31498000	+
chr18	31947500	-
chr18	31991600	-
chr18	32009900	+
chr18	32638100	+
chr18	41700400	-
chr18	41751000	-
chr18	41774500	-
chr18	44825700	-
chr18	44892800	+
chr18	45066100	-
chr18	45113900	-
chr18	46756700	-
chr18	49939100	+
chr18	49950200	-
chr18	52751200	+
chr18	52839600	+
chr18	53372800	-
chr18	53389000	-
chr18	53421400	+
chr18	54557600	+
chr18	55157700	+
chr18	58088000	-
chr18	58364000	-
chr18	58393300	+
chr18	59221800	-
chr18	59800500	-
chr18	65721600	-
chr18	72730600	+
chr18	72747000	-
chr18	72769000	-
chr18	72779600	-
chr18	72800900	-
chr18	74974700	-
chr18	75026900	+
chr18	75056400	-
chr18	75176800	-
chr18	75222000	-
chr18	75995400	+
chr19	5064900	+
chr19	8227000	-
chr19	8439700	-
chr19	10126900	-
chr19	10985300	+
chr19	11000300	-
chr19	12868800	+
chr19	13893300	+
chr19	14129700	+
chr19	14672900	-
chr19	16375300	-
chr19	17030500	+
chr19	17134900	+
chr19	17539900	-
chr19	19250100	+
chr19	19325200	+
chr19	21127600	-
chr19	24028100	-
chr19	24042700	-
chr19	39524800	-
chr19	40936200	-
chr19	43282700	-
chr19	43293800	-
chr19	44552800	+
chr19	44559000	-
chr19	45521400	-
chr19	45867800	+
chr19	46479100	-
chr19	46585500	-
chr19	47290300	+
chr19	48933100	+
chr19	49280900	+
chr19	51033900	+
chr19	51043200	-
chr19	51583200	+
chr19	52278100	+
chr19	52401000	+
chr19	54991200	-
chr19	57416200	-
chr19	57822000	-
chr19	59558500	+
chr19	63515600	+
chr19	63545200	+
chr19	63620900	+
chr2	269300	+
chr2	3158100	+
chr2	3175200	+
chr2	3239900	-
chr2	3336200	-
chr2	3449500	+
chr2	3494700	-
chr2	7057100	-
chr2	8835500	-
chr2	8859600	-
chr2	9915100	-
chr2	10498300	+
chr2	10632500	+
chr2	10639800	+
chr2	10843500	-
chr2	11862200	+
chr2	11872900	-
chr2	11881200	+
chr2	15344000	-
chr2	15386800	+
chr2	15662200	-
chr2	20097200	-
chr2	20345600	-
chr2	20685900	+
chr2	20750700	-
chr2	20757500	-
chr2	24114600	-
chr2	24296700	+
chr2	24346300	+
chr2	25309000	+
chr2	25826200	+
chr2	26298800	-
chr2	27280600	-
chr2	27303100	+
chr2	27310800	-
chr2	27410800	+
chr2	27716600	+
chr2	29220100	-
chr2	30595900	+
chr2	32542400	-
chr2	32564100	+
chr2	32593500	-
chr2	32760200	-
chr2	39066000	-
chr2	39093200	+
chr2	39816800	+
chr2	43412400	-
chr2	43478800	+
chr2	43637700	-
chr2	44418800	+
chr2	45680900	+
chr2	47109900	+
chr2	47120600	+
chr2	48548000	-
chr2	48567500	+
chr2	48580300	+
chr2	48596800	+
chr2	53934600	+
chr2	54735800	-
chr2	54749400	+
chr2	63685500	-
chr2	65162900	+
chr2	68871100	+
chr2	70017100	-
chr2	70144600	-
chr2	71219800	+
chr2	71468500	-
chr2	71475000	+
chr2	73306000	+
chr2	73594500	+
chr2	74180900	-
chr2	74245600	+
chr2	74441300	+
chr2	74571400	-
chr2	74756300	-
chr2	85729500	-
chr2	85925200	-
chr2	86108900	-
chr2	86175200	+
chr2	86208700	+
chr2	86215800	-
chr2	86291600	+
chr2	86545000	+
chr2	86563200	+
chr2	86569700	-
chr2	86588800	+
chr2	95129400	-
chr2	95136100	+
chr2	96216000	-
chr2	96221800	-
chr2	96313100	-
chr2	96320300	+
chr2	96640700	+
chr2	96735200	-
chr2	96741100	+
chr2	96763300	+
chr2	97889500	+
chr2	98521600	-
chr2	98566200	-
chr2	98657400	-
chr2	99380800	-
chr2	101854200	+
chr2	101859200	-
chr2	102702100	+
chr2	105257800	+
chr2	105264000	+
chr2	105280600	-
chr2	105326700	+
chr2	105332800	+
chr2	108747100	+
chr2	109383500	+
chr2	112561100	-
chr2	112996300	+
chr2	113026900	-
chr2	113050000	+
chr2	114448100	+
chr2	118300400	-
chr2	118407400	-
chr2	118419200	+
chr2	118432000	-
chr2	118442800	-
chr2	118449200	-
chr2	118471300	+
chr2	120377400	+
chr2	120428300	-
chr2	120440000	-
chr2	121820200	+
chr2	121837000	+
chr2	121855100	-
chr2	121922400	-
chr2	121988100	+
chr2	127733600	+
chr2	127745600	+
chr2	127756700	+
chr2	127788300	+
chr2	127795600	-
chr2	128190700	-
chr2	128238600	-
chr2	128338300	+
chr2	128424900	-
chr2	128474000	-
chr2	128488300	-
chr2	128613700	-
chr2	128641400	-
chr2	128650100	+
chr2	134902700	+
chr2	134910600	-
chr2	135428200	+
chr2	135645100	+
chr2	136154800	-
chr2	136258500	-
chr2	139011400	+
chr2	143676500	+
chr2	143986200	-
chr2	143993000	-
chr2	144034400	+
chr2	144086800	+
chr2	144115000	+
chr2	144243300	+
chr2	149258800	+
chr2	151985300	+
chr2	158302500	+
chr2	158312400	-
chr2	158355900	+
chr2	159927700	+
chr2	160842100	+
chr2	161795300	-
chr2	162596800	+
chr2	162610300	-
chr2	168570700	+
chr2	168591400	-
chr2	168658900	-
chr2	168714100	+
chr2	168721500	+
chr2	169134400	+
chr2	169261500	-
chr2	171531100	+
chr2	172034400	-
chr2	172288900	-
chr2	172295000	-
chr2	173064700	-
chr2	173144700	+
chr2	173169500	+
chr2	174951500	-
chr2	175136700	-
chr2	175153000	+
chr2	179068900	-
chr2	179112400	-
chr2	179121500	+
chr2	180524600	-
chr2	182072100	+
chr2	191062300	-
chr2	191076300	+
chr2	191495500	-
chr2	191503900	+
chr2	191544200	+
chr2	196300500	-
chr2	197566400	-
chr2	197615200	+
chr2	197652100	+
chr2	198053300	-
chr2	198719400	-
chr2	201064300	-
chr2	201737100	-
chr2	201849000	-
chr2	201961800	-
chr2	203498500	+
chr2	203505900	-
chr2	203825600	-
chr2	206577700	-
chr2	206719800	-
chr2	207351700	+
chr2	207652600	-
chr2	208474400	-
chr2	208573500	+
chr2	208850600	-
chr2	208878200	+
chr2	208919800	+
chr2	211034300	-
chr2	215919200	-
chr2	216572500	+
chr2	216710500	+
chr2	216720800	+
chr2	216729600	+
chr2	216744200	+
chr2	216779400	+
chr2	216997800	-
chr2	217011400	-
chr2	219168300	+
chr2	219211500	-
chr2	219310900	-
chr2	223482500	-
chr2	223493900	+
chr2	224467600	+
chr2	225347300	-
chr2	225392700	-
chr2	225402200	-
chr2	225424200	-
chr2	225448900	-
chr2	227437900	-
chr2	227482300	+
chr2	228110800	+
chr2	230745800	+
chr2	230756200	+
chr2	230810600	+
chr2	230859200	-
chr2	230931600	-
chr2	230961700	-
chr2	231040600	+
chr2	231072400	+
chr2	231083800	-
chr2	231391400	-
chr2	232369500	+
chr2	232383200	-
chr2	232594900	-
chr2	232601800	+
chr2	232788400	-
chr2	232813200	+
chr2	232836200	-
chr2	232849400	+
chr2	232872600	-
chr2	233142300	-
chr2	233154600	+
chr2	233388800	+
chr2	233407300	+
chr2	233430400	+
chr2	233744800	+
chr2	233848300	+
chr2	233864100	-
chr2	234029400	-
chr2	237673800	-
chr2	238348200	+
chr2	238355500	-
chr2	238761400	+
chr2	238849600	-
chr2	239712200	-
chr2	240576500	-
chr2	240588800	-
chr2	240714500	+
chr2	241757800	+
chr2	241827700	-
chr2	241841100	-
chr2	241939200	+
chr2	242003400	-
chr20	416700	-
chr20	1373300	+
chr20	1380600	+
chr20	2940400	+
chr20	2950100	+
chr20	3120200	+
chr20	3505600	-
chr20	3800900	+
chr20	4716400	-
chr20	4726600	+
chr20	4816600	+
chr20	13661200	-
chr20	13720300	-
chr20	16445100	-
chr20	17562100	-
chr20	17878200	+
chr20	18116400	-
chr20	18245300	-
chr20	18457600	-
chr20	18475300	-
chr20	18482000	-
chr20	18553900	-
chr20	20336600	-
chr20	20442800	-
chr20	20556600	+
chr20	20567600	+
chr20	23322700	+
chr20	24901100	+
chr20	25143600	+
chr20	25252700	+
chr20	25439000	-
chr20	25604500	-
chr20	30188600	-
chr20	30197200	-
chr20	30268800	+
chr20	30274300	-
chr20	31410900	+
chr20	31682400	-
chr20	32618100	+
chr20	32794200	-
chr20	33063600	-
chr20	33100700	-
chr20	33169700	+
chr20	33357800	-
chr20	33411100	+
chr20	33512300	+
chr20	33605800	-
chr20	33725200	-
chr20	33894200	+
chr20	33969300	+
chr20	35266500	+
chr20	35289200	+
chr20	35293400	-
chr20	35800200	+
chr20	35819800	+
chr20	35922800	-
chr20	36049200	+
chr20	36063100	+
chr20	36107400	-
chr20	36128900	-
chr20	36588500	-
chr20	36603400	-
chr20	36607600	-
chr20	36825500	-
chr20	37069000	-
chr20	39238200	-
chr20	39477900	-
chr20	39483900	+
chr20	39518900	+
chr20	39532500	-
chr20	39560600	-
chr20	39572400	+
chr20	42557400	-
chr20	42563700	-
chr20	42696700	+
chr20	43004800	-
chr20	43013000	+
chr20	43866900	-
chr20	43939900	-
chr20	44021400	+
chr20	44438000	-
chr20	44450600	-
chr20	45274600	-
chr20	45698100	-
chr20	45715100	-
chr20	47005200	-
chr20	47151800	+
chr20	47285800	+
chr20	47298200	+
chr20	47313100	+
chr20	47686100	+
chr20	47703400	-
chr20	47915100	-
chr20	48001800	-
chr20	49445200	+
chr20	50209000	+
chr20	54460600	+
chr20	54503100	+
chr20	55378400	+
chr20	56254300	+
chr20	56678400	+
chr20	57001500	-
chr20	60011100	+
chr20	60173800	-
chr21	14791400	-
chr21	14797300	-
chr21	14804200	-
chr21	15261500	-
chr21	16089500	+
chr21	31995100	+
chr21	32613600	-
chr21	32653100	+
chr21	32660900	-
chr21	32666400	-
chr21	32677600	-
chr21	32897800	-
chr21	33036400	+
chr21	33805100	-
chr21	34196600	+
chr21	34263400	+
chr21	35093100	-
chr21	35121100	-
chr21	36329600	+
chr21	36339200	-
chr21	37393300	+
chr21	37471300	+
chr21	37495400	+
chr21	39113000	+
chr21	39465900	+
chr21	39585700	-
chr21	41690600	-
chr21	41702600	+
chr21	41740200	-
chr21	41752100	-
chr21	42150800	+
chr21	42195800	-
chr21	42212000	-
chr21	42286300	-
chr21	42575600	-
chr21	42829200	-
chr21	43062900	+
chr21	43310200	+
chr21	43318200	+
chr21	43929900	+
chr21	44346600	+
chr21	45465400	-
chr21	46438100	-
chr21	46755700	-
chr21	46782900	-
chr21	46790200	+
chr22	16460800	-
chr22	16475300	-
chr22	16590500	+
chr22	16596500	-
chr22	17717800	+
chr22	17728200	-
chr22	17743800	-
chr22	17753800	+
chr22	17764900	-
chr22	17821600	+
chr22	18281100	-
chr22	19431900	+
chr22	19449200	+
chr22	19483600	-
chr22	19567100	-
chr22	20456500	-
chr22	20614900	-
chr22	20651900	+
chr22	21002600	-
chr22	22416500	-
chr22	22497500	+
chr22	22642400	+
chr22	22767300	-
chr22	22777500	-
chr22	22802800	+
chr22	22811700	+
chr22	22815900	+
chr22	22841900	+
chr22	23058100	+
chr22	23064700	-
chr22	23076600	-
chr22	23123700	+
chr22	25190300	-
chr22	25196700	+
chr22	25220500	+
chr22	25509300	+
chr22	25516600	-
chr22	28061400	+
chr22	28068500	-
chr22	28079700	-
chr22	28084700	+
chr22	28255000	-
chr22	28262200	-
chr22	28394500	+
chr22	28401100	-
chr22	28515100	-
chr22	28531300	-
chr22	28728500	+
chr22	28751600	+
chr22	29143200	-
chr22	29653800	-
chr22	30163000	+
chr22	30276600	-
chr22	30329100	+
chr22	30439100	+
chr22	30548700	-
chr22	31224600	+
chr22	33990800	+
chr22	34068900	-
chr22	34136400	+
chr22	34145900	+
chr22	34159600	-
chr22	34867200	+
chr22	35243100	+
chr22	36559400	-
chr22	36595900	+
chr22	36603200	-
chr22	36961100	+
chr22	38408500	-
chr22	38695100	+
chr22	39895400	+
chr22	39949900	+
chr22	39990600	+
chr22	40061100	-
chr22	40544700	-
chr22	40611600	+
chr22	40619800	+
chr22	40786400	-
chr22	40882100	-
chr22	41532900	-
chr22	42704200	-
chr22	44106200	-
chr22	44110700	-
chr22	44477700	+
chr22	44516600	+
chr22	44582100	-
chr22	45948500	-
chr22	49208700	-
chr3	4657600	-
chr3	4670800	+
chr3	4703500	+
chr3	4750400	+
chr3	4763000	+
chr3	4788400	+
chr3	4799800	-
chr3	4834400	-
chr3	5234100	-
chr3	9459300	+
chr3	9465300	+
chr3	9711600	-
chr3	10319600	-
chr3	11617400	+
chr3	11807100	-
chr3	11815300	-
chr3	11824500	+
chr3	11836500	+
chr3	12590700	-
chr3	12925500	+
chr3	14474900	-
chr3	15021400	-
chr3	15029800	-
chr3	15040300	+
chr3	15048800	-
chr3	15063200	-
chr3	15456800	-
chr3	15702300	-
chr3	17183100	+
chr3	18368700	+
chr3	20112000	+
chr3	20140200	-
chr3	25640500	+
chr3	25743200	+
chr3	31648700	-
chr3	32164100	+
chr3	32172100	-
chr3	32552200	-
chr3	33029800	-
chr3	33074400	+
chr3	33426100	-
chr3	36843300	-
chr3	36868500	+
chr3	36999100	-
chr3	37030700	+
chr3	37057100	+
chr3	37372000	+
chr3	37401200	+
chr3	38270200	+
chr3	38499100	+
chr3	39083900	-
chr3	39091000	-
chr3	39103000	+
chr3	39112000	+
chr3	41250400	+
chr3	41262100	+
chr3	42104400	+
chr3	42239600	-
chr3	42566100	+
chr3	44468200	-
chr3	44771000	+
chr3	44945700	+
chr3	45412000	-
chr3	45430700	-
chr3	45653000	+
chr3	45688400	+
chr3	45698700	-
chr3	45761100	+
chr3	45940000	-
chr3	45975400	+
chr3	45998300	+
chr3	46691100	+
chr3	47350800	-
chr3	47361100	+
chr3	47512700	+
chr3	47635300	-
chr3	47860200	+
chr3	48476200	+
chr3	48705200	-
chr3	48764500	+
chr3	48870100	+
chr3	48991900	-
chr3	49297900	-
chr3	49313100	+
chr3	49337300	-
chr3	49924500	+
chr3	50114500	-
chr3	51639800	-
chr3	51649100	-
chr3	52214700	-
chr3	52357800	+
chr3	52487300	+
chr3	52567900	-
chr3	52588200	-
chr3	52618000	-
chr3	52659000	+
chr3	52861100	+
chr3	53099400	+
chr3	53132400	-
chr3	53236900	-
chr3	53884400	+
chr3	56577900	+
chr3	56650500	-
chr3	57878000	+
chr3	58092700	-
chr3	58345400	+
chr3	63973400	+
chr3	67510400	+
chr3	67649100	+
chr3	67661000	+
chr3	67741300	-
chr3	69193200	+
chr3	71096200	+
chr3	71103600	-
chr3	106860700	+
chr3	106920700	-
chr3	106964600	+
chr3	107026500	-
chr3	109235200	+
chr3	110055300	-
chr3	112788300	-
chr3	112856400	+
chr3	114829200	-
chr3	114861400	-
chr3	115277900	-
chr3	115288100	+
chr3	115553100	+
chr3	120414200	+
chr3	120581900	-
chr3	120719700	-
chr3	121805100	+
chr3	122834100	+
chr3	122845900	-
chr3	122881900	+
chr3	122926000	+
chr3	123734900	-
chr3	123899200	-
chr3	123921600	-
chr3	123941200	+
chr3	126171000	-
chr3	126221700	+
chr3	126434100	+
chr3	127209900	-
chr3	127218800	+
chr3	127934900	+
chr3	128771200	+
chr3	128810000	-
chr3	129263500	-
chr3	129301900	+
chr3	129347700	-
chr3	129448100	+
chr3	129464500	+
chr3	129476100	-
chr3	129485200	+
chr3	129553100	-
chr3	129568400	-
chr3	129999700	+
chr3	130014700	+
chr3	130097500	+
chr3	130103100	+
chr3	130333300	-
chr3	130470300	+
chr3	130633700	-
chr3	133911400	-
chr3	135024300	+
chr3	135738300	-
chr3	137499800	+
chr3	137537100	-
chr3	139461300	-
chr3	139467400	-
chr3	139970600	-
chr3	140573000	+
chr3	142718800	+
chr3	143534000	-
chr3	143589300	+
chr3	143596900	+
chr3	144853100	+
chr3	150211100	+
chr3	150366200	+
chr3	155476000	+
chr3	157138200	+
chr3	159537100	+
chr3	159862600	+
chr3	159906100	-
chr3	172265400	+
chr3	172289600	-
chr3	172301100	+
chr3	172326300	+
chr3	172336200	-
chr3	172341800	-
chr3	172349600	-
chr3	172378000	+
chr3	172406100	-
chr3	173707500	-
chr3	180775700	-
chr3	184282400	-
chr3	184708100	-
chr3	185034200	+
chr3	185366600	-
chr3	185526200	+
chr3	186166700	-
chr3	186229100	-
chr3	186690200	-
chr3	187786000	+
chr3	188932100	+
chr3	189810900	+
chr3	195847300	-
chr3	195853500	+
chr3	196507400	+
chr3	196521800	+
chr3	196905200	+
chr3	196911300	-
chr3	196921500	-
chr3	196927500	-
chr3	197682900	-
chr3	198116000	-
chr3	198819100	-
chr3	198894600	-
chr3	198902800	+
chr3	199052900	+
chr3	199066900	-
chr4	499000	-
chr4	1359000	-
chr4	1872900	-
chr4	2632200	-
chr4	2642900	-
chr4	2669100	-
chr4	2871100	+
chr4	2918600	-
chr4	3086700	+
chr4	3106400	-
chr4	3125100	+
chr4	3131400	+
chr4	3154200	+
chr4	3160500	+
chr4	3184800	+
chr4	3191300	+
chr4	4366200	+
chr4	4509700	-
chr4	4522500	+
chr4	6353700	+
chr4	6669200	-
chr4	6904600	-
chr4	6914000	-
chr4	6920500	+
chr4	7062400	+
chr4	8507200	+
chr4	8517300	-
chr4	9677400	-
chr4	13211200	-
chr4	13224000	+
chr4	14676500	-
chr4	15774700	-
chr4	15804100	-
chr4	17100700	+
chr4	17208300	-
chr4	24786400	+
chr4	25007300	+
chr4	25022100	-
chr4	25395000	-
chr4	25422100	-
chr4	25428800	-
chr4	26037800	-
chr4	26530900	-
chr4	26628100	-
chr4	35801700	+
chr4	37312500	+
chr4	37580700	+
chr4	37694900	-
chr4	37733600	+
chr4	38375800	+
chr4	38989900	-
chr4	41720200	+
chr4	48209600	+
chr4	48218600	-
chr4	52433900	+
chr4	52470100	-
chr4	53907800	-
chr4	54014300	+
chr4	56440800	+
chr4	68013200	-
chr4	68040800	+
chr4	68269200	+
chr4	74224800	+
chr4	74254600	-
chr4	76674300	+
chr4	77060900	+
chr4	77096800	+
chr4	77117800	+
chr4	78160100	+
chr4	81046700	-
chr4	81117600	+
chr4	83599500	-
chr4	88227200	+
chr4	88261000	-
chr4	88274700	+
chr4	88480700	-
chr4	89531500	+
chr4	89555300	-
chr4	89796400	+
chr4	89850900	-
chr4	89868900	+
chr4	100976500	+
chr4	103449100	+
chr4	103688300	-
chr4	106375100	+
chr4	107478000	+
chr4	110635500	-
chr4	113497300	+
chr4	119846100	-
chr4	119952100	-
chr4	124094200	-
chr4	129061900	+
chr4	130012800	+
chr4	142374500	+
chr4	146290000	-
chr4	147331600	+
chr4	149231500	-
chr4	149250100	-
chr4	149260500	+
chr4	151421900	-
chr4	152879900	+
chr4	154763400	-
chr4	164655900	-
chr4	169405700	-
chr4	169544100	-
chr4	169556700	-
chr4	169569800	-
chr4	169577000	+
chr4	169594400	-
chr4	169628500	+
chr4	174409400	-
chr4	184049400	-
chr4	184062200	-
chr5	688300	-
chr5	922400	+
chr5	5497300	+
chr5	5513300	+
chr5	6657700	-
chr5	7947800	+
chr5	7953600	+
chr5	10449500	+
chr5	14668500	-
chr5	14741500	-
chr5	14751800	+
chr5	14798100	-
chr5	14804000	+
chr5	16503200	+
chr5	16622700	+
chr5	31474100	+
chr5	31508800	+
chr5	31518300	+
chr5	31538500	-
chr5	31549400	+
chr5	32269300	+
chr5	32295300	+
chr5	32426900	-
chr5	36195400	+
chr5	37666900	+
chr5	41775100	+
chr5	41784400	-
chr5	41793600	+
chr5	41831200	+
chr5	41836400	-
chr5	41842000	-
chr5	41887600	-
chr5	43210200	-
chr5	43496800	-
chr5	43686000	-
chr5	43739000	+
chr5	50120700	+
[truncated: 422,580 more chars]
